# NeoMProbe: a new class of fluorescent cellular and tissue membrane probe†

**DOI:** 10.1039/d4sc06225f

**Published:** 2024-10-30

**Authors:** Saurabh Anand, Preeti Ravindra Bhoge, Rakesh Raigawali, Srinivas Vinod Saladi, Raghavendra Kikkeri

**Affiliations:** a Department of Chemistry, Indian Institute of Science Education and Research Pune 411008 India rkikkeri@iiserpune.ac.in; b Department of Cell and Cancer Biology, University of Toledo, College of Medicine and Life Sciences Toledo OH 43614 USA srinivas.saladi@utoledo.edu; c Department of CPAS, Jackson State University Jackson Mississippi 39217 USA

## Abstract

The development of long-lasting plasma membrane (PM) and basement membrane (BM) probes is in high demand to advance our understanding of membrane dynamics during differentiation and disease conditions. Herein, we report that the microheterogeneity of heparan sulfate (HS) on fluorescent neo-proteoglycans backbone offers a facile platform for designing membrane probes. Confocal live-cell imaging studies of cancer and normal cell lines with a panel of Cy5 fluorescently tagged neo-proteoglycans confirmed that highly sulfated HS ligands with an l-iduronic acid component (PG@ID-6) induce a prolonged and brighter expression on the PM compared to low-sulfated and uronic acid counterparts. Mono- and multi-photon microscopic imaging of tissue sections with NeoMProbe (PG@ID-6) allowed mapping BM and demonstrated staining efficacy equivalent to antibodies against the BM components. Finally, *in vivo*, whole-body imaging of NeoMProbe and subsequent tissue section imaging confirmed versatile and efficient membrane mapping by the probe. Overall, NeoMProbe offers a novel toolkit for cell biology and tissue biomembrane imaging.

## Abstract 2

A new class of plasma membrane and basement membrane probe derived from heparan sulfate proteoglycan is reported.

## Graphical abstract

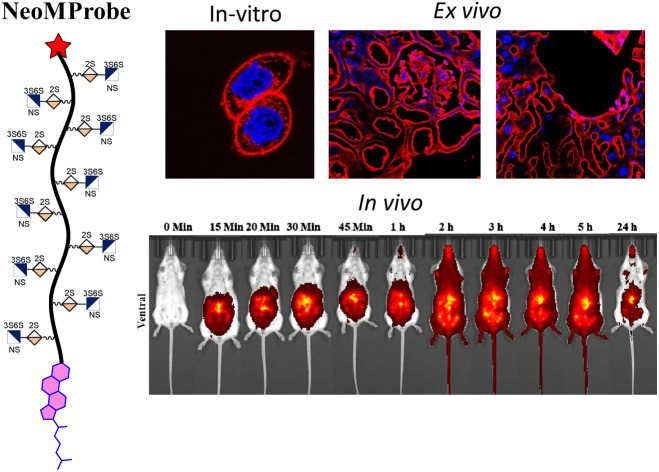

Plasma membrane (PM) and basement membrane (BM) are constituents of cells and tissues. They play a central role in a broad spectrum of physiological and pathological processes.^1–6^ Visualizing these membranes using biomarkers offers a comprehensive overview of the local conditions of tissues and cells, including their health and activity.^7^ In recent years, there has been a significant expansion in the design of fluorescence markers that aim to target the PM to elucidate cellular events.^8–14^ Despite relevant progress, critical challenges in PM imaging remain, particularly in distinguishing the PM of various cell types under disease conditions and maintaining marker longevity to explore PM dynamics.^8^ Chromophores, such as chromone, Nile red, BODIPY, perylene, and fluorescein isothiocyanate, have been modified with zwitter ionic and amphiphilic groups to develop methods of PM staining.^15–20^ However, these probes are internalized within short intervals, revealing poor information on PM dynamics.^21^ Conversely, visualization of BM using a synthetic probe is extremely challenging as its environment is more complex and heterogeneous, resulting in non-specific staining. Hence, fluorescent-tagged antibodies directed at BM components have been extensively used.^22,23^ Overall, a new model is required to design synthetic membrane probes that are effective, not only at the cellular level but also for *ex vivo* and *in vivo* membrane mapping. Herein, we report the design of a novel class of membrane probes that exploit the function of proteoglycan to promote membrane anchoring and imaging (Fig. 1).

**Fig. 1:**
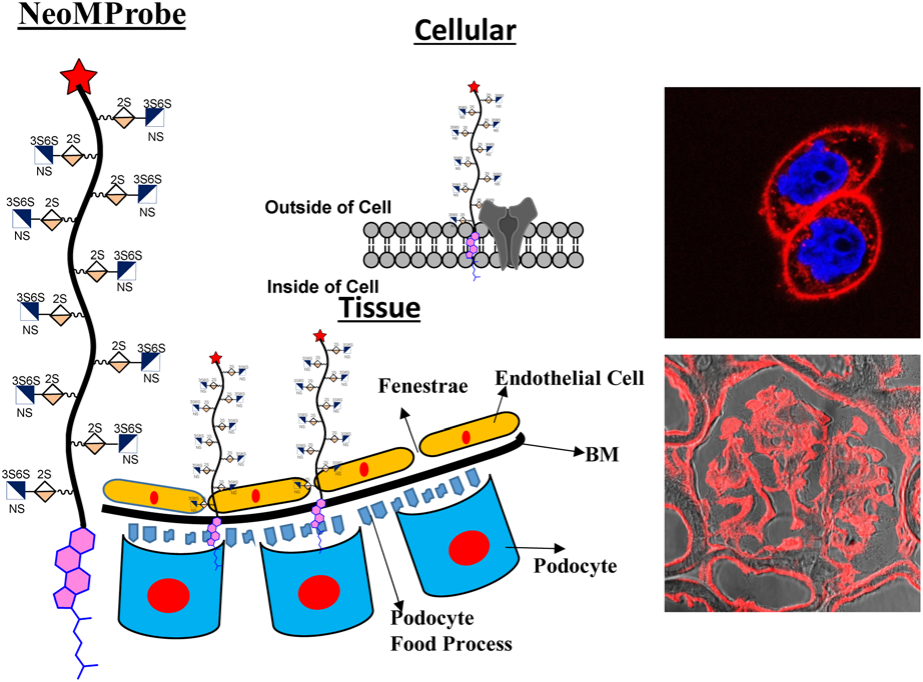
NeoMProbe as a biomarker on the cell membrane and liver basement membrane.

Proteoglycans (PG) are a combination of proteins and glycosaminoglycans (GAGs), where the ectodomains of proteins are anchored to highly-sulfated GAGs, including heparan sulfate (HS). They are ubiquitously expressed on the cell membrane and extracellular matrix.^24^ Consequently, HS-based neo-proteoglycans (neoPGs) are promising targets for glycocalyx remodeling and membrane probe development.^25^ Pioneering advances in glycopolymer and glycopeptide-based neoPGs have demonstrated their PM retention for a longer time scale, ensuring active growth factor signaling.^26–29^ A thorough understanding of the key elements controlling the membrane dynamics of neoPGs could inspire new strategies for effective glycocalyx remodeling and the development of novel membrane probes. To this end, we synthesized a panel of Cy5-tagged neoPGs to study the structural heterogeneity of HS altering the membrane persistence and differentiating the cell types at tissue slices. We employed various imaging techniques and whole-body studies to show the compatibility of these membrane probes.

## Results and discussions

**Scheme 1:**
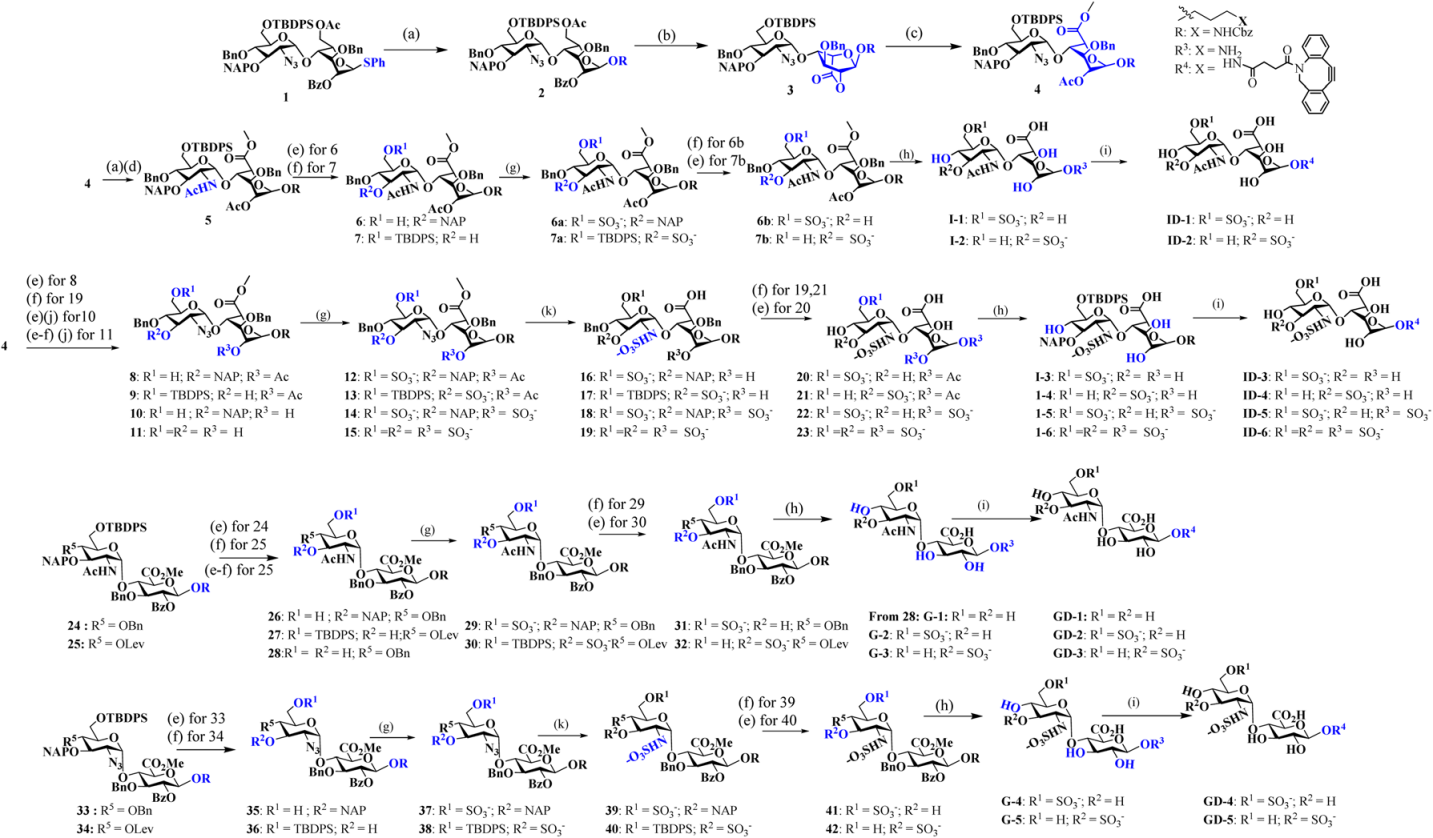
Synthesis of heparan sulfate (HS) disaccharide analogs: reagents and conditions: (a) R = Linker, NIS, TMSOTf, DCM, −10 °C, 15 min; (b) NaOMe, MeOH, RT, 12 h; TEMPO, BAIB, DCM : H_2_O (1 : 1), RT; (c) 1 M LiOH, THF : H_2_O (1 : 1), RT, 2 h; MeI, K_2_CO_3_, DMF, 12 h; Ac_2_O, DCM : Py (1 : 1), 0 °C, 12 h; (d) Zn dust, THF : AcOH : Ac_2_O (3 : 2 : 1), RT, 12 h; (e) HF·Py, Py, 0 °C, 12 h; (f) DDQ, DCM : H_2_O (18 : 1), RT, 1 h; (g) SO_3_. Et_3_N, DMF, 60 °C, 24 h; (h) (i) LiOH·H_2_O, H_2_O, 12 h; (ii) H_2_, Pd(OH)_2_, MeOH. 48 h; (i) DBCO-NHS linker, Et_3_N, DMF, 24 h. (j) LiOH·H_2_O, H_2_O; (k) PMe_3_. THF, 24 h; (ii) SO_3_·Py. MeOH, 1N NaOH, 48 h.

**Scheme 2:**
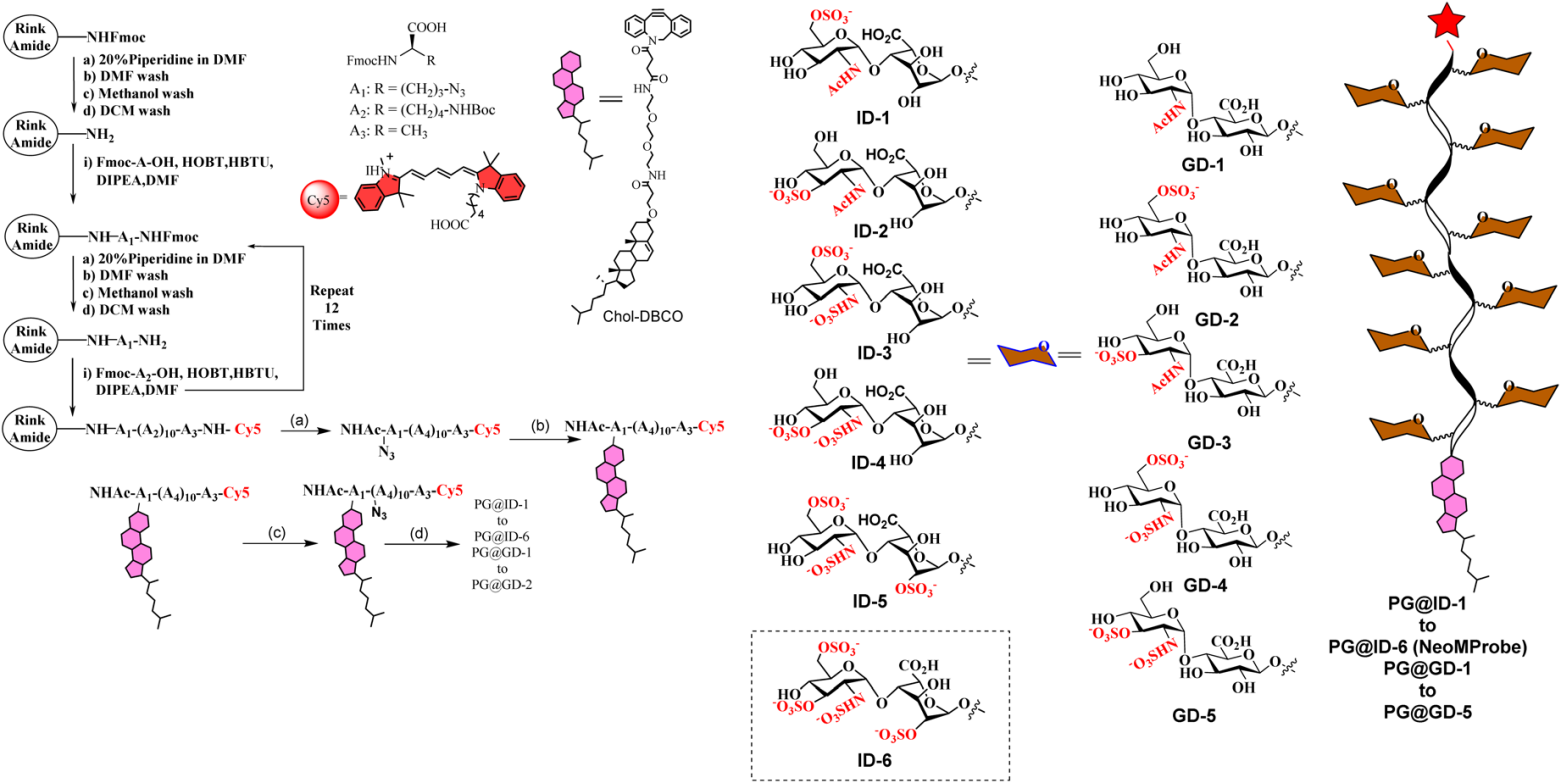
Synthesis of proteoglycan mimetics: reagents and conditions: (a) TFA : H_2_O : TIPS : Phenol (18 : 1 : 1 : 0.5); (b) MeOH : H_2_O (1 : 1), RT, 12 h, chol-DBCO, 92%; (c) 3-azido-propanyl-NHS, DIPEA, DMF, RT, 16 h, 60%; (d) ID-1 to ID-6 or GD-1 to GD-5, H_2_O, 6 days 24–35%.

Like natural proteoglycans, the synthetic neo-proteoglycan is composed of a peptide chain comprising an azide functional group to which HS disaccharide ligands are orthogonally functionalized *via* a copper-free click reaction. These glycopeptides are also functionalized with a Cy5 fluorescent tag for live imaging of the probe and cholesterol moiety to anchor them on the cell membrane. Previously, it has been shown that cholesterol moieties significantly improve the mucin mimetic cell surface expression compared to the phospholipid counterparts.^30^ A library of HS disaccharides was synthesized by modifying the previously documented protocols.^31–38^ In the HS disaccharide library, ID-1 to -6 represent l-iduronic acid domain HS ligands, while compounds GD-1 to -5 correspond to d-glucuronic acid-based HS ligands. Compounds, ID-1, ID-2, and GD-1 to -3, are composed of either 6-*O* or 3-*O*-sulfated or non-sulfated *N*-acetate glucosamine derivatives. For comparison, we synthesized highly sulfated *N*- sulfated ID-3 to -6 and GD-4 to -5 ligands. These disaccharides were synthesized from thiodonors, 1, 24, and 33, that were orthogonally protected by TBDPS (*tert*-butyldiphenylsilyl), 2-NAP (2-naphthylmethyl), and (N_3_) azide at the 6th, 3rd, and 2nd positions of glucosamine, respectively, for selective sulfation and *N*-acetylation/*N*-sulfation (Scheme 1). Thiodonor 1 was glycosylated with a CBZ-protected propanol linker utilizing *N*-iodosuccinimide (NIS), and the trimethylsilyl trifluoromethanesulfonate (TMSOTf) promoter yielded 85% of product 2. Subsequently, deacetylation, oxidation of the primary hydroxyl of 2 with a catalytic 2,2,6,6-tetramethyl-1-piperidinyloxyl free radical (TEMPO) and [bis(acetoxy)iodo]benzene (BAIB) yielded 79% of the lactonized product 3, followed by lithium hydroxide-based lactone ring opening and methyl esterification to yield product 4. Azide to amine conversion in the presence of zinc dust and acetic acid, followed by acetylation, yielded *N*-acetylation HS disaccharide precursor 5. While *N*-sulfation of 12–15 in the presence of sulphur trioxide pyridine complex yielded 16–19 respectively. Selective TBDPS or 2-NAP deprotection using hydrogen fluoride·pyridine complex (HF·py) or 2,3-dichloro-5,6-dicyano-1,4-benzoquinone (DDQ), and global deprotection yielded I-1 to I-6, respectively. Similar reaction conditions with 24 and 33 donors yielded G-1 to G-5 ligands, respectively (Scheme 1). These ligands were conjugated to the DBCO linker, purified by flash column, and used as such in the next step. To validate sulfation pattern-dependent proteoglycan activity, we functionalised disaccharide ligands on a peptide-based proteoglycan scaffold carrying a Cy5 fluorescent tag for imaging studies. Solid-state peptide chemistry and rink amide resin were utilised to synthesise a 12-mer peptide, carrying 10 lysine residues positioned between 5-azido-pentanoic acid and l-alanine at the N- and C-terminals of the peptide, facilitating the conjugation of Cy5 and the cholesterol linker, respectively (Scheme 2).^39^ The peptide was purified using HPLC and characterized through NMR, mass-spectra, UV, and fluorescent spectroscopy. The photophysical properties of neoPGs were investigated in water at room temperature. All complexes showed characteristic peaks of Cy5 with 5–6 nm bathochromic shift compared to the Cy5 as such, indicating the glycoconjugation (Tables S1 & S2†).

**Fig. 2:**
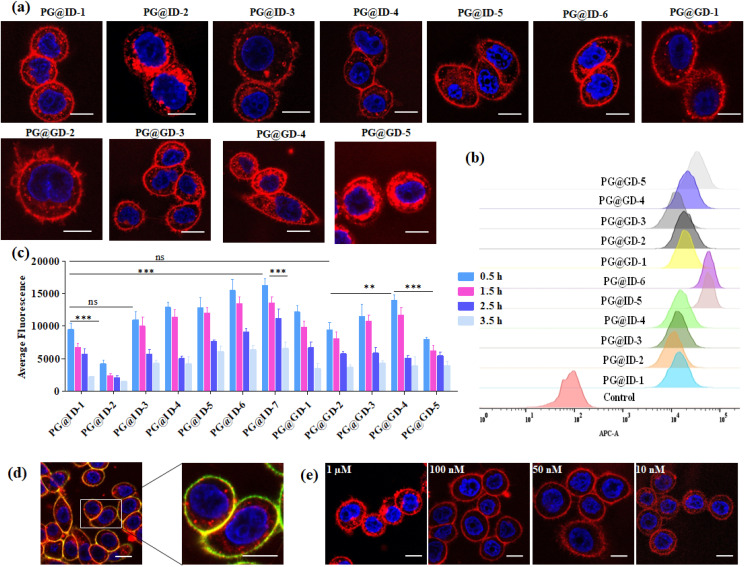
(a) Confocal imaging of proteoglycan mimetics in MDA-MB468 cells after 30 min of incubation; (b) FACS analysis of proteoglycan mimetics uptake after 1 h incubation in MDA-MB 468 cells; (c) average surface fluorescence quantification of proteoglycan mimetics in MDA-MB 468 cells at different time interval *n* = 50. The asterisk indicates statistically significant differences (**p < 0.001; ***p < 0.0001; ns, not significant). Statistical analysis is performed using a two-tailed Student's *t*-test; (d) colocalization of PG@ID-6 with CD44 Monoclonal antibody-FITC cell membrane marker in MDA-MB 468 cell line after 30 min; (e) confocal images of PG@ID-6 at different concentrations (scale bar = 10 μm).

Following the synthesis and characterization of the 11 neoPGs, we assessed their relative toxicity in both cancer and normal cells using the MTT assay (Fig. S2a & b†). It was observed that neither cell type showed significant cytotoxicity at concentrations up to 5 μM, even after 48 hours. Next, we investigated the PM dynamics of the neoPGs using MDA-MB-468 and NIH-3T3 cells (purchased from the National Centre for Cell Science, Pune, India), as they have different compositions of anionic glycans and sugar densities.^40^ This disparity was hypothesized to cause electrostatic repulsion with the glycocalyx glycans, thereby altering their assembly on the PM, akin to DNA–lipid conjugates.^41^ A solution of neoPGs (2 μM) was added to the cells and washed after 30 min of incubation; confocal images of all neoPG mimetics revealed clear decoration on the PM, where some analogs showed internalization. To our surprise, PG@ID-2 showed maximum internalization within 30 min (Fig. 2a), whereas other sulfate mimetics displayed intense fluorescence on the PM and slowly underwent endocytosis. Among the different sulfation patterns, 6-*O*-sulfated neoPGs exhibited significantly more PM assembly, compared to their 3-*O*-sulfated counterparts. Contrarily, uronic acid compositions in neoPGs did not show any significant differences. Notably, heavily sulfated l-iduronic acid-based neoPGs (PG@ID-6) showed intense PM fluorescence compared to the non-sulfated or low-sulfated neoPGs (Fig. 2c). To confirm the PM decoration of the PG@ID-6, a FITC-conjugated anti-CD44, and E-cadherin co-staining experiment was performed (Fig. 2d). A strong Pearson correlation coefficient confirmed the PM assembly of PG@ID-6 (Fig. 2d & S8†). FACS measurement after 1 h, at which most of the neoPGs were expected to decorate the cell membrane, except for the 3-*O*-sulfated analogs, clearly reiterated that the degree of sulfation significantly improves the overall cellular expression (Fig. 2b). Next, we determined the PM dynamics of the neoPG through live imaging studies at different time intervals. All compounds underwent 50–90% endocytosis after 3.5 h of incubation in MDA-MB-468 (quantified based on Fig. 2c). Interestingly, PG@ID-6, named NeoMProbe, displayed a slow internalization process compared to other analogs (Fig. 2c and S3†). Unlike cancer cells, NIH-3T3 normal cells exhibited a faster internalization process (Fig. S5 and S6†) within 2 h, illustrating that the composition and dense glycocalyx layer significantly altered the PM dynamics of neoPGs. Contrarily, NeoMProbe once again demonstrated maximum PM expression as compared to all other analogs in NIH-3T3. Next, to validate the mechanism of PM expression and endocytosis of neoPGs, we performed PM engineering studies under glycocalyx-modified conditions. Here, we treated the MDA-MB-468 cells with heparinase and sialidase enzymes to cleave sialic acid and HS glycans from the cell membrane. The aim was to evaluate whether the electrostatic interaction with glycocalyx modulates the PM expression of NeoMProbe. As expected, cleavage of negatively charged glycans significantly reduces the PM decoration of NeoMProbe (Fig. S9†). Further, we also evaluated the stabilization of NeoMProbe by the cell surface receptors. To this end, we treated MDA-MB-468 cells with an anti-EGFR antibody followed by NeoMProbe addition, as cancer cells ubiquitously express the EGFR receptor (Fig. S10†).^31^ After 1 h, we observed a substantial decrease in NeoMProbe expression on the cell membrane, indicating that the cell surface receptors are another important factor influencing the stability of the NeoMProbe expression on the cell membrane. All these studies confirm that the degree of sulfation and sulfation patterns of HS synergistically fine-tune the PM dynamics of neoPGs. Considering that 6-*O* sulfation and *N*-sulfation are essential binding patterns of HS sulfation for most growth factors receptors in comparison to 3-*O*-sulfated and *N*-acetate HS,^42^ it is hypothesized that 6-*O*-sulfated *N*-sulfated ligands are essential for PM expression. Altogether, our results confirmed that strong sulfation is essential for cell membrane expression and NeoMProbe is the best PM probe among the reported 11 neoPGs. Similar to commercial PM makers,^43^NeoMProbe stained the PM of cancer cells at concentrations of up to 10 nM (Fig. 2e & S7†); additionally, the co-staining of NeoMProbe with DiO (Fig. S11†) demonstrates long-lasting membrane staining, indicating its potential for commercial use.

**Fig. 3:**
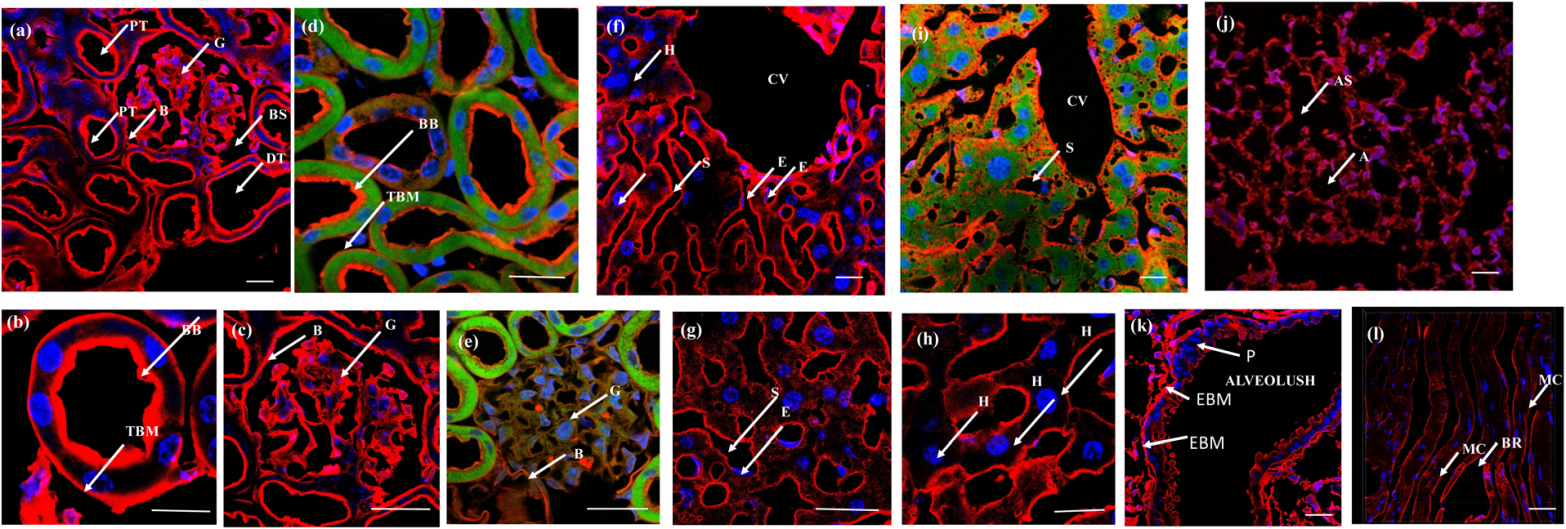
Tissue slides, 1 mm-thick, were incubated with NeoMProbe and DAPI: (a) kidney sections display the staining of the BM of distal (DT) and proximal tubules (PT), glomerulus (G), and the Bowman's capsule region. The yellow box corresponds to the zoomed region. (b) and (c) Zoomed kidney section displaying BM and brush border staining of NeoMProbe (brush border (BB) and tubular basement membrane (TBM) bowman's space (BS)); (d) and (e) double immunofluorescent images kidney slices with NeoMProbe (red) and FITC anti-E-cadherin (green); (f)–(h) liver sections exhibit selective staining at the sinusoidal (S) and central vein (CV) membranes over the hepatocyte cell membrane (H) sinusoidal endothelial cells (E); (i) double immunofluorescent images of liver slices with NeoMProbe (red) and FITC anti-E-cadherin (green); (j) lung section shows the marking of the BM of alveolar sacs (AS), alveoli (A) region (k) zoomed region of alveolus, NeoMProbe shows the staining of the epithelial basement membrane (EBM) close to the pneumocyte type I and type II cells (P); (l) heart sections demonstrate the staining of NeoMProbe at the BM close to the muscle endothelial cells (MC) and also highlight the branching (BR) region (scale bar = 100 μm).

**Fig. 4:**
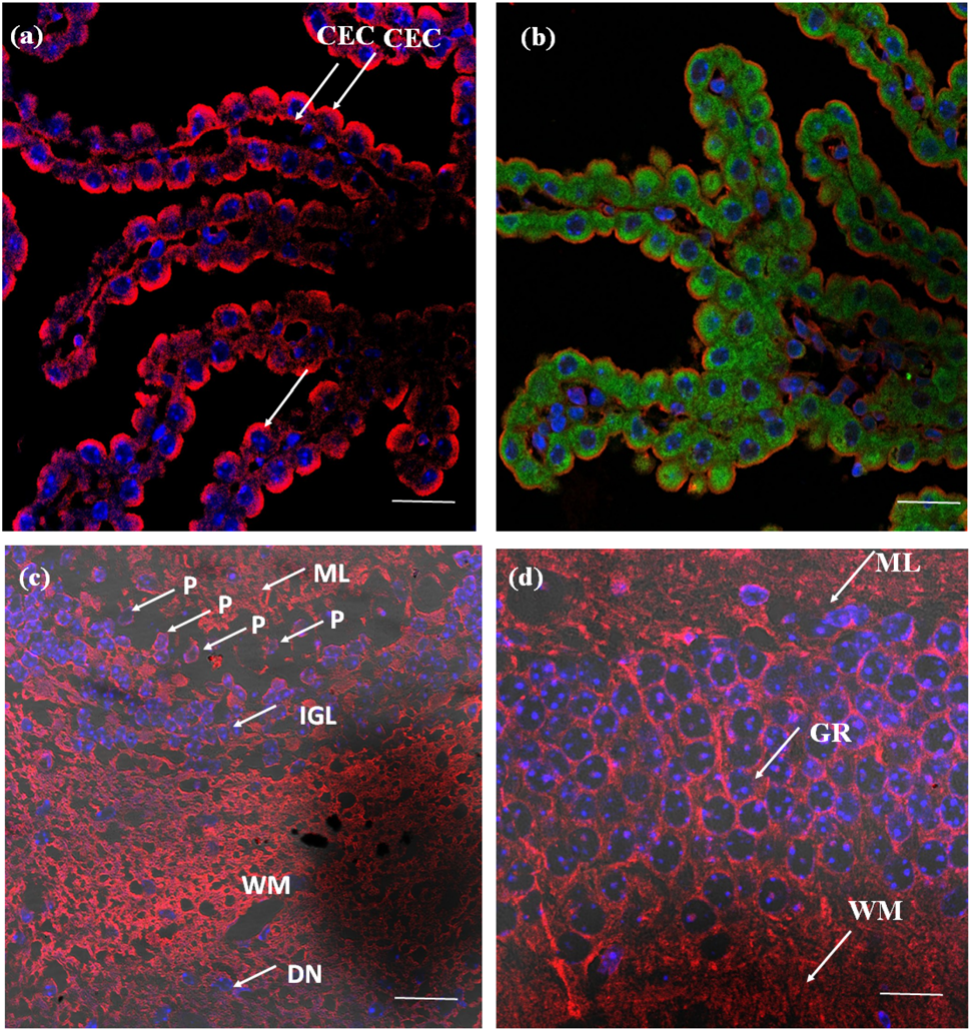
Brain slides, 1 mm thick, were incubated with NeoMProbe and DAPI: (a) 100× confocal images of the 3rd ventricle region show selective staining of the cell membrane of cuboidal epithelial cells (CEC) in the choroid plexus; (b) immunofluorescent double labelling analysis of the 3rd ventricle with NeoMProbe and FITC anti-E-cadherin monoclonal antibody; (c) cerebellum and (d) hippocampal region shows differentiation among the white matter (WM) granular region (GR), Purkinje layer (P), inner granular layer (IGL), and the molecular layer (ML). The hippocampal region shows staining of neurite axon networks of different layers (scale bar = 100 μm).

The efficacy of the PM probe at the cellular level inspired us to extend our testing to tissue membranes using NeoMProbe. Accordingly, we prepared 1 mm-thick tissue sections from mouse liver, kidney, heart, lung, and brains obtained from the National Facility for Gene Function in Health and Disease (NFGFHD) at IISER Pune, India. Tissues were stained with NeoMProbe for 1 hour and washed before imaging (Fig. S12–S15,†3 & 4). Previous studies have indicated that lipophilic PM markers, such as DiO, DiD, and PKH26, often suffer from poor water solubility, resulting in uneven labelling of tissue membranes.^43^ In contrast, the zwitterion-based probes, such as the MemBright series, have successfully stained liver hepatocyte membranes and primary neuronal cultures.^44^ However, probing BM and PM in tissues and animals necessitates a specific modification for appropriate distribution across tissue membranes. We hypothesized that the neoPG backbone could modify the properties of chromophores and enhance BM/PM staining in tissues and animals. To our surprise, the kidney slices exhibited exceptional staining of BM in both the tubular and glomerulus regions (Fig. 3a–c). In the glomerulus region, NeoMProbe distinctively marked the Bowman's capsule and glomeruli, differentiating them from the Bowman's space (Fig. 3c). Within the Bowman's capsule, the NeoMProbe notably stained the BM closely associated with the epithelial cell membrane. In the glomerular region, the double immunostaining with FITC anti-E-cadherin and NeoMProbe displayed staining of BM between the podocytes and endothelial cell lines (Fig. 3e). In the tubular region, NeoMProbe provided strong staining of the BM of distal and proximal tubules, which was further confirmed by E-cadherin co-staining (Fig. 3d). Upon closer inspection of multi-photon 3D images, staining was primarily observed on the BM close to the proximal tubular epithelial cell membrane and brush border (Videos 1 & 2†). These results demonstrate that the proteoglycan backbone significantly modulates the fluorescent staining properties at the tissue level. Moreover, these results are comparable to the kidney section staining of BM with immunostaining of α3 subunit of collagen VI in the literature.^45^ Overall, NeoMProbe serves as a potential marker for kidney BM, and it has future prospective applications in the study of anti-glomerular BM diseases. Similarly, liver tissue staining with NeoMProbe exhibited preferential staining of sinusoidal walls within the sinusoidal lumen and did not stain the PM of hepatocyte cell line, suggesting its potential to differentiate the membrane in a heterogeneous liver system (Fig. 3f–h). As the endothelial cell membrane and blood vessels of the sinusoidal microvesicle are closely associated, NeoMProbe behaves similarly to the cell maskTM and VE-cadherin antibodies staining the BM of sinusoidal microvesicles (Fig. 3i, Videos 3 & 4†).^46,47^ In heart sections, NeoMProbe effectively stained the BM of cardiac muscle fibers, allowing differentiation of branching and striation junctions (Fig. 3l, Video 5†).^48^ Additionally, NeoMProbe labelled the epithelial BM of pneumocyte type I and type II cells in the alveolar regions of the lung sections (Fig. 3l and k, Video 6†).^49^ Next, we carried out an immunofluorescent analysis of the sections of the brain while focusing on the cerebellum, hippocampal, and third ventricle regions (Fig. 4). In the third ventricle section, we observed notable BM staining of the choroid plexus epithelial cell wall, distinguishing it from the stromal capillary. When immunofluorescent double labelling analyses were conducted using FITC anti-E-cadherin and NeoMProbe, E-cadherin was observed in the PM layer beneath the NeoMProbe, showing no co-localization (Fig. 4a and b, Video 7†). This demonstrates that the BM and PM of epithelial cells in the third ventricle are differentiated. In cerebellar regions, NeoMProbe exhibited more prominent staining networks in the white matter and molecular layer compared to the granular region. It is hypothesized that NeoMProbe marked the myelinated climbing fibres and mossy fibres, which form extensive networks in these areas (Fig. 4c). In the Purkinje region, NeoMProbe stained the outer layer of the Purkinje cells but did not stain the axons or dendrites emanating from them. Further efforts with confocal and multiphoton imaging did not result in better images, indicating the need for a more detailed investigation. A similar trend was also observed in the hippocampal region, warranting further investigation of brain sections (Fig. 4d). Overall, NeoMProbe is a potential BM marker. To investigate the component involved in BM staining over another tissue compartment, we performed the immunostaining assay of PG@ID-2 and the peptide backbone as such. We observe that PG@ID-2 also stained the BM in a similar way as NeoMProbe. However, the intensity was very poor compared to NeoMProbe. While the peptide backbone as such showed no staining, indicating that the peptide backbone and the HS ligands synergistically influenced the BM staining.

**Fig. 5:**
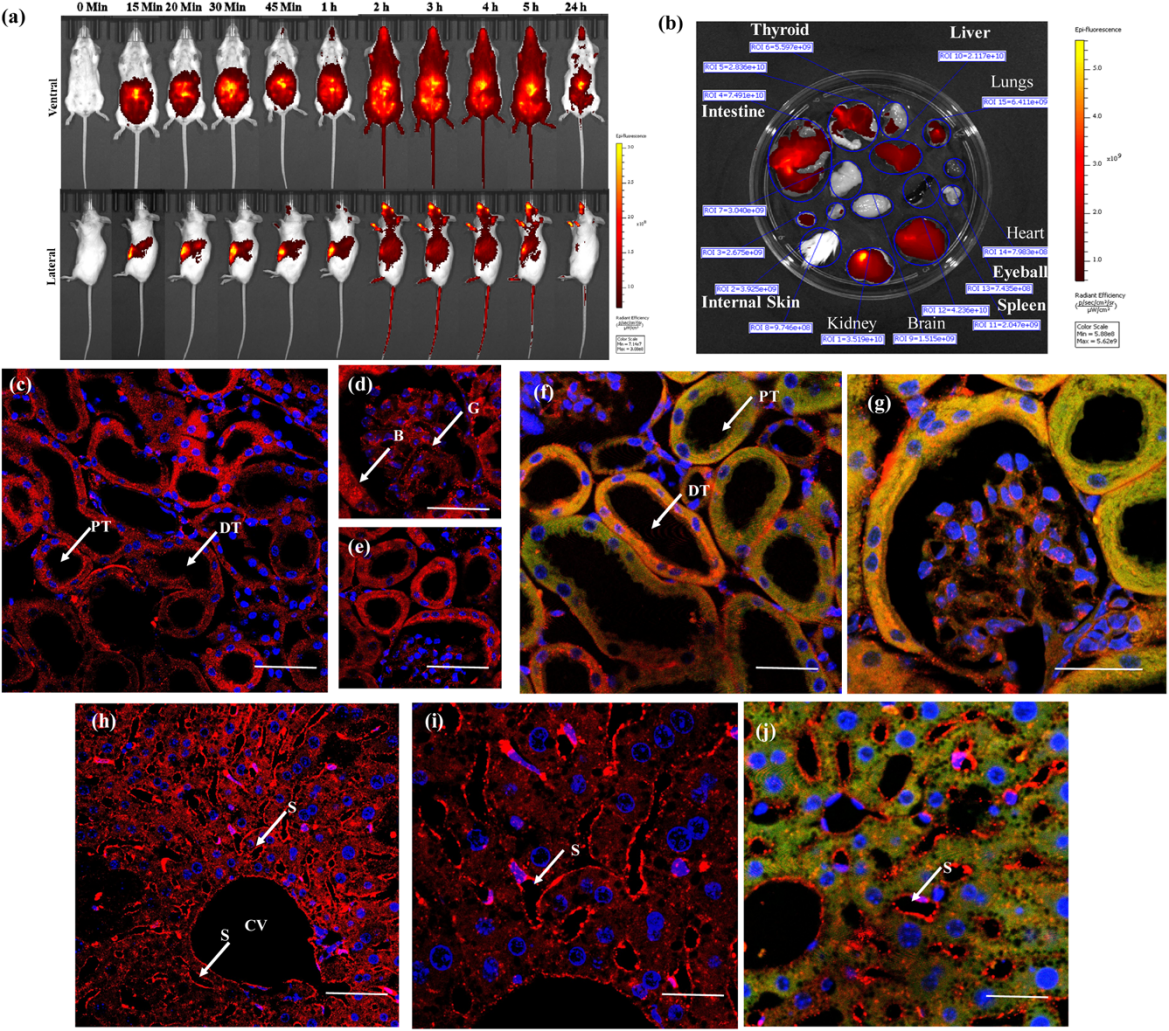
(a) Live animal imaging for different time intervals after injecting 2 μM of NeoMProbe (b) photograph of major organs collected after 3 h of intraperitoneal injection of the NeoMProbe. (c)–(e) mono-photon images of kidney slices after 3 h post-injection of NeoMProbe; (f) and (g) double immunofluorescent staining (anti E-cadherin (green)) and NeoMProbe (red) of kidney slices; (h) and (i) liver section processed after 2 h of intraperitoneal injection of NeoMProbe; (j) double immunofluorescent staining of liver slices (scale bar = 100 μm).

Inspired by the successful *ex vivo* imaging studies, we subsequently conducted *in vivo* live imaging to assess the compatibility of the probe with animals and explore its real-time applications for membrane probing. To this end, we performed BALB/c mice live imaging studies. NeoMProbe was injected intraperitoneally (i.p.), and whole-body images were collected at different time intervals (Fig. 5a and b). The advantage of intraperitoneal (i.p.) injection over intravenous (i.v.) injection is that it ensures slower absorption of the probe, allowing a better understanding of biodistribution. As anticipated, NeoMProbe demonstrated slow uptake. Within 15 minutes to 1 hour, the majority of the probe was localized in the lower part of the body, particularly in the kidneys, liver, intestines, and gastrointestinal tract (Fig. 5a). After 2 hours, the entire body appeared to be stained with the probe, including the brain; the stain persisted for nearly 5 hours, and slowly cleared in the next 24 hours, clearly showcasing its potential as a biomarker for whole-body imaging. When we examined tissue sections of the liver, kidney, and brain after 3 h post-injection, the kidney slices showed persistent NeoMProbe presence in the tubular and glomerular regions (Fig. 5c–e). However, their biodistribution and sequestration are quite different from the Cy5 probe as such,^50^ indicating the neoPG backbone modulates Cy5 *in vivo* activity. Unlike *ex vivo* staining, the NeoMProbe exhibited wide bio-distribution. The double immunofluorescent staining with anti-E-cadherin confirmed that the NeoMProbe was not only used to stain the BM, but also the PM and other cellular compartments (Fig. 5f and g). Similarly, liver sections also showed significant persistence of the sinusoidal membrane, as well as the hepatocyte cellular region (Fig. 5h–j). However, to our surprise, we did not observe any fluorescence in the brain sections, indicating that the molecules had very poor permeability for the brain tissue. This suggests that the HS composition of NeoMProbe may have interacted with various active biological systems, including growth factors, chemokines, and morphogens, leading to different behaviour *in vivo* compared to *in vitro* and *ex vivo* levels, where the biological complexity is limited. It is expected that further modification of the NeoMProbe with HS mimetics and peptide backbone may increase BM specificity *in vivo*, which is currently under investigation.

## Conclusions

We introduced neoPGs-based membrane probes that are compatible across cellular, tissue, and animal levels. Unlike amphiphilic and zwitter ion-based fluorescent probes, these probes are designed by using a proteoglycan mimetic backbone. They were synthesized by using peptides and heparan sulfate ligands. Confocal imaging studies of Cy5 neoPG mimetics demonstrated that the cell membrane persistence depends on the sulfation patterns. Our results indicated that proteoglycan mimetics carrying *N*-sulfated and l-iduronic acid-based HS ligands (PG@ID-6 or NeoMProbe) exhibited greater cell membrane persistence compared to those with *N*-acetylated derivatives. Systematic mechanism studies showed that electrostatic interaction with glycocalyx and membrane receptor binding stabilizes the NeoMProbe on PM for a long time. *Ex vivo* imaging studies of the NeoMProbe displayed BM staining of tissues. Notably, BM in the tubular and granular region of kidney sections was selectively mapped over cell membrane decoration, while, in the liver section, NeoMProbe stained sinusoidal over the hepatocyte region. In the heart and lung section, BM on muscle cells and pneumocyte type I/II cells were stained. These results align with the BM component antibody staining as published in the literature.^41–43^*In vivo* live imaging, followed by tissue section imaging, underscores the potential of these probes to work as membrane biomarkers. However, we failed to demonstrate membrane specificity. The system's flexible architecture permits the use of membrane probes to explore various fluorescent probes, with Cy5 being interchangeable with any other fluorescent labels. This simple exchange of fluorescent or carbohydrate epitopes will greatly streamline the process of generating collections of NeoMProbes and glycocalyx remodeling systems.

## Data availability

The data supporting this article have been included as part of the ESI.†

## Author contributions

R. K. and S. V. S. planned the project. S. A., R. R., and P. R. B., synthesized, purified, and characterized the proteoglycan mimetics and performed all bioassays. R. K., S. A., and P. R. B. analysed the data and wrote the manuscript with some assistance from other co-authors.

## Conflicts of interest

R. K. and S. V. S. are cofounders of Reinwik Inc., and also have a patent for NeoMProbe pending.

## Supplementary Material

SC-015-D4SC06225F-s001

SC-015-D4SC06225F-s002

SC-015-D4SC06225F-s003

SC-015-D4SC06225F-s004

SC-015-D4SC06225F-s005

SC-015-D4SC06225F-s006

SC-015-D4SC06225F-s007

SC-015-D4SC06225F-s008
