# Correction: NeoMProbe: a new class of fluorescent cellular and tissue membrane probe

**DOI:** 10.1039/d6sc90033j

**Published:** 2026-02-13

**Authors:** Saurabh Anand, Preeti Ravindra Bhoge, Rakesh Raigawali, Srinivas Vinod Saladi, Raghavendra Kikkeri

**Affiliations:** a Department of Chemistry, Indian Institute of Science Education and Research Pune 411008 India rkikkeri@iiserpune.ac.in; b Department of Cell and Cancer Biology, University of Toledo, College of Medicine and Life Sciences Toledo OH 43614 USA srinivas.saladi@utoledo.edu; c Department of CPAS, Jackson State University Jackson Mississippi 39217 USA

## Abstract

Correction for ‘NeoMProbe: a new class of fluorescent cellular and tissue membrane probe’ by Saurabh Anand *et al.*, *Chem. Sci.*, 2024, **15**, 19962–19969, https://doi.org/10.1039/D4SC06225F.

The authors regret that in Fig. 5a, due to an oversight during the final assembly of the figure panels, the image for the lateral 2-hour time point was inadvertently duplicated and placed in the panel representing the 3-hour and 4-hour time points. The corrected figure is shown below. This correction does not affect any of the conclusions of the article.

**Fig. 5:**
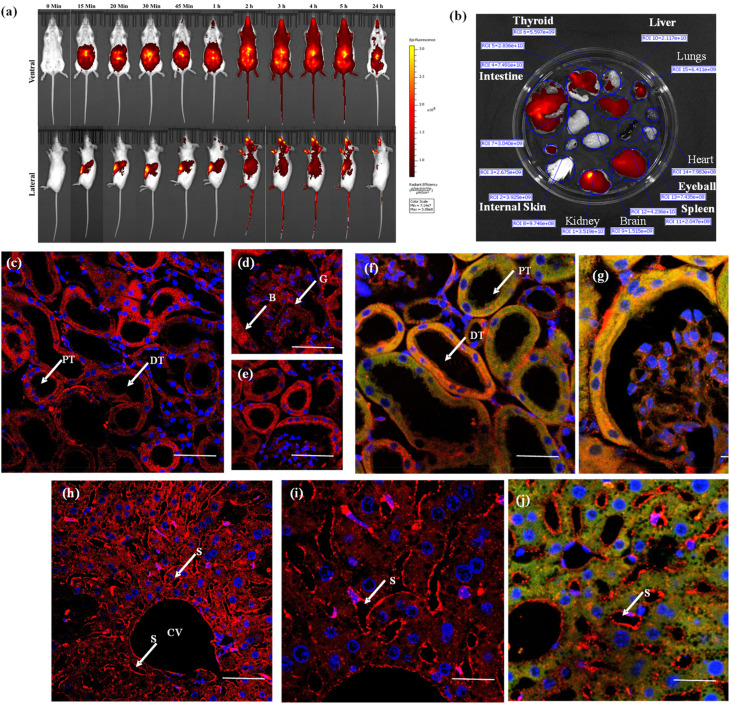
(a) Live animal imaging for different time intervals after injecting 2 µM of NeoMProbe; (b) photograph of major organs collected after 3 h of intraperitoneal injection of the NeoMProbe. (c)–(e) Mono-photon images of kidney slices after 3 h post-injection of **NeoMProbe**; (f) and (g) double immunofluorescent staining (anti E-cadherin (green)) and NeoMProbe (red) of kidney slices; (h) and (i) liver section processed after 2 h of intraperitoneal injection of NeoMProbe; (j) double immunofluorescent staining of liver slices (scale bar = 100 µm).

The Royal Society of Chemistry apologises for these errors and any consequent inconvenience to authors and readers.

