# Supplementary material for: NeoMProbe: a new class of fluorescent cellular and tissue membrane probe
Source: Chem Sci. 2024 Oct 30;15(47):19962–9. doi: 10.1039/d4sc06225f (PMC11575582; doi:10.1039/d4sc06225f)
Supplement: SC-015-D4SC06225F-s001 [file SC-015-D4SC06225F-s001.pdf]

Supplementary Information (SI) for Chemical Science.
This journal is © The Royal Society of Chemistry 2024

SUPPORTING INFORMATION

NeoMProbe: A New Class of Fluorescent Cellular and Tissue Membrane Probe

Saurabh Anand,[†] Preeti Ravindra Bhoge,[†] Rakesh Raigawali,[†] Srinivas Vinod Saladi,[± *] Raghavendra Kikkeri.[*†¥]

[†]Indian Institute of Science Education and Research, Dr. Homi Bhabha Road, Pune 411008, India

[±]Department of Cell and Cancer Biology, University of Toledo, College of Medicine and Life Science, Toledo, OH 43614, USA

[¥]Department of CPAS, Jackson State University, Jackson, Mississippi, 39217, USA

Email. rkikkeri@iiserpune.ac.in; srinivas.saldi@utoledo.edu

1. General Information

All chemicals were reagent grade and used without further purification unless otherwise noted. Reactions were carried out in anhydrous solvents under a nitrogen atmosphere. Reaction progress was monitored by analytical thin-layer chromatography (TLC) on Merck silica gel 60 F_{254}. Spots on TLC plate were visualized under UV light or by dipping the TLC plate in CAM/ninhydrin solution followed by heating. Column chromatography was carried out using Fluka kiesel gel 60 (230-400 mesh). ^1H and ^{13}C NMR spectra of compounds were measured with Bruker 400 MHz, Bruker 600 MHz and Jeol 400 MHz using residual solvents as an internal reference ($CDCl_3$ δH 7.26 ppm, δC 77.3 ppm, CD_3OD δH 3.31 ppm, δC 49.0 ppm, and D_2O δH 4.79 ppm). The chemical shifts (δ) are reported in ppm and coupling constants (J) in Hz. UV-visible measurements were performed with Evolution 300 UV-visible spectrophotometer (Thermo Fisher Scientific, USA). Fluorescence spectra were measured with FluoroMax-4 spectrofluorometer (Horiba Scientific, U.S.A.). All microscopy images were captured using Leica SP8 confocal microscope and processed using Image J software.

2. Synthesis of Iduronic acid based Disaccharides

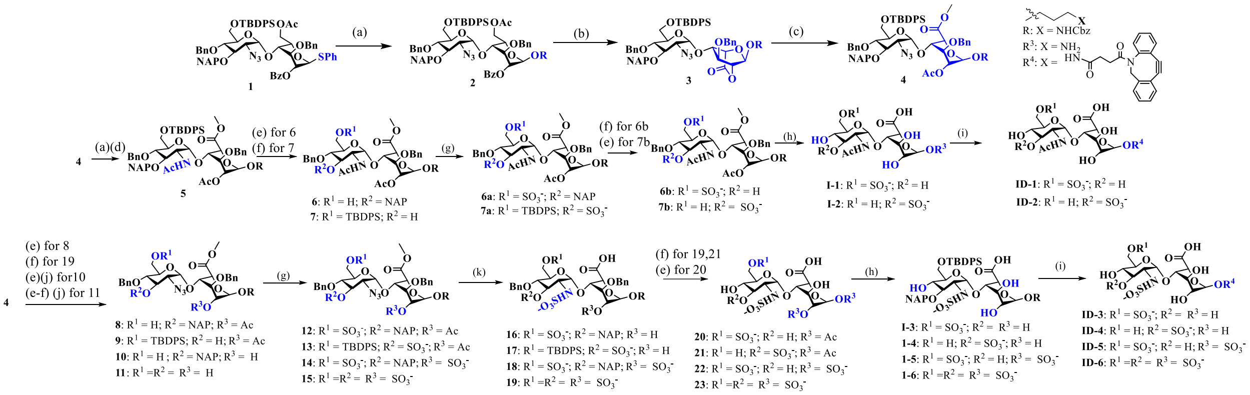

Scheme S1. Synthesis of Iduronic acid based HS disaccharides (a) R = Linker, NIS, TMSOTf, DCM, -10 °C, 15 min; (b) NaOMe, MeOH, RT, 12 h; TEMPO, BAIB, DCM:H_2O (1:1), RT; (c) 1 M LiOH, THF: H_2O (1:1), RT, 2 h; MeI, K_2CO_3, DMF, 12 h; Ac_2O, DCM:Py (1:1), 0 °C, 12 h; (d) Zn dust, THF:AcOH:Ac_2O (3:2:1), RT, 12 h; (e) HF.Py, Py, 0 °C, 12 h; (f)DDQ, DCM:H_2O (18:1), RT, 1 h; (g) SO_3. Et_3N, DMF, 60 °C, 24 h; (h) (i) LiOH.H_2O, H_2O, 12 h; (ii) H_2, $Pd(OH)_2$, MeOH. 48 h; (i) DBCO-NHS linker, Et_3N, DMF, 24 h. (j) LiOH.H_2O, H_2O; (k) PMe_3.THF, 24 h; (ii) SO_3.Py. MeOH, 1N NaOH, 48 h.

1,6-Anhydro-[2-azido-4-*O*-benzyl-6-*O*-*tert*-butyldiphenylsilyl-3-*O*-(2 naphthylmethyl)-2-deoxy-*α*-D-glucopyranosyl]-(1→4)-*O*-2-*O*-benzoyl-3-*O*-benzyl-*β*-L- idopyranose (1b)

To a solution of compound **1d** donor (1g, 1 eq) and acceptor (0.41 g, 0.8 eq) in CH_2Cl_2 (20 mL) was stirred under N_2 atmosphere in round bottom flask containing freshly dried 4 Å molecular sieves for 1 h. The mixture solution was cooled down to -78 ^{o} C followed by the addition of NIS (0.46 g, 2.08 mmol) and TMSOTf (71 µL, 0.39 mmol). The temperature was gradually increased to -20 ^{o} C and reaction mixture was left for stirring for another 15 minutes. After 15 minutes reaction completion was monitored by TLC and quenched using few drops of Et_3N. Molecular sieves were filtered using celite and organic layer was washed with aqueous $Na_2S_2O_3$ and brine. The collected organic layer was dried over Na_2SO_4, filtered, concentrated and purified through silica gel column chromatography (ethyl acetate/ hexane= 1/12, v/v) to obtain compound **1e** in

65 % yield. ^{1}H NMR (400 MHz, Chloroform-d) δ 8.08 – 8.04 (m, 2H), 7.83 (m, 4H), 7.70 – 7.67 (m, 4H), 7.62 – 7.54 (m, 2H), 7.51 – 7.42 (m, 7H), 7.42 – 7.35 (m, 5H), 7.31 – 7.27 (m, 3H), 7.24 – 7.18 (m, 3H), 7.17 – 7.13 (m, 2H), 5.52 (d, J = 1.8 Hz, 1H), 5.32 – 5.29 (m, 1H), 5.12 – 5.06 (m, 3H), 4.89 (t, J = 10.4 Hz, 2H), 4.76 (d, J = 10.8 Hz, 1H), 4.66 – 4.57 (m, 2H), 4.14 (d, J = 7.7 Hz, 1H), 4.10 – 3.99 (m, 3H), 3.88 (d, J = 1.8 Hz, 2H), 3.75 – 3.65 (m, 3H), 3.45 (dd, J = 10.3, 3.8 Hz, 1H), 1.07 (s, 9H). ^{13}C NMR (101 MHz, CDCl3) δ 165.8, 138.0, 137.5, 135.8, 135.7, 135.2, 133.4, 133.3, 133.2, 132.9, 129.9, 129.9, 129.8, 129.4, 128.6, 128.5, 128.4, 128.4, 128.1, 128.1, 128.0, 127.9, 127.8, 127.7, 127.0, 126.2, 126.1, 126.0, 99.7, 99.3, 80.0, 79.4, 78.9, 78.3, 77.4, 77.0, 76.7, 75.7, 75.5, 75.0, 74.2, 72.9, 65.7, 63.7, 62.6, 31.0, 26.9, 26.8, 25.3, 19.2. HR-ESI-MS *(m/z)*: $[M+Na]^+$ calcd for $C_{60}H_{61}N_3O_{10}SiNa$, 1034.4024; found, 1034.4010.

Phenyl-[2-azido-4-*O*-benzyl-6-*O*-*tert*-butyldiphenylsilyl-3-*O*-(2-naphthylmethyl)-2-deoxy-*α*-D-glucopyranosyl]-(1→4)-*O*-2-*O*-benzoyl-3-*O*-benzyl-6-*O*-acetyl-1-thio-*α*-L- idopyranoside (1)

A solution of compound **1e** (1 g, 0.98 mmol, 1 eq) in Ac_2O (10 mL) was stirred at ice cold temperature for 15 minutes before the addition of $Cu(OTf)_2$ (0.035 g, 0.098 mmol, 0.1 eq). After 16 h, the reaction mixture was concentrated under reduced pressure and the residue was extracted with ethyl acetate, $NaHCO_3$ and washed with brine. The combined organic layer was dried over Na_2SO_4, filtered, concentrated and preceded further without any purification. Next, the as obtained acetylated solution of compound (0.98 g, 0.88 mmol), ZnI_2 (0.59 g, 1.84 mmol) and phenyl trimethylsilyl sulphide (0.50 g, 2.72 mmol) in CH_2Cl_2 (15 mL) was stirred under N_2 atmosphere for 2 h. Upon completion, the reaction mixture was filtered through celite, evaporated and purified through silica gel column chromatography (ethyl acetate/ hexane= 1/5, v/v) to obtain compound **1** in 85 % yield. ^{1}H NMR (400 MHz, Chloroform-d) δ 8.20 – 8.16 (m, 2H), 7.81 – 7.74 (m, 3H), 7.70 (d, J = 1.5 Hz, 1H), 7.68 – 7.66 (m, 2H), 7.65 (d, J = 1.5 Hz, 1H), 7.58 – 7.55 (m, 3H), 7.51 – 7.46 (m, 4H), 7.43 – 7.37 (m, 5H), 7.36 – 7.27 (m, 14H), 7.19 – 7.16 (m, 2H), 5.62 (s, 1H), 5.40 (t, J = 2.5 Hz, 1H), 4.99 (d, J = 11.6 Hz, 1H), 4.90 - 4.88 (m, 1H), 4.82 – 4.68 (m, 3H), 4.63 (d, J = 3.7 Hz, 1H), 4.55 (d, J = 10.6 Hz, 1H), 4.41 (dd, J = 11.6, 8.1 Hz, 1H), 4.24 – 4.15 (m, 3H), 4.02 – 3.98 (m, 1H), 3.88 (d, J = 11.5, 1H), 3.81 – 3.65 (m,4H), 3.36 (dd, J = 9.9, 3.7 Hz, 1H), 1.91 (s, 3H), 1.05 (s, 9H). ^{13}C NMR (101 MHz, CHLOROFORM-D) δ 170.5, 165.9, 138.1, 137.4, 136.0, 135.7, 135.6, 135.2, 133.6, 133.4, 133.1, 133.1, 131.8,

130.0, 130.0, 129.8, 129.8, 129.0, 128.7, 128.6, 128.5, 128.3, 128.2, 128.2, 128.1, 127.9, 127.9, 127.8, 127.8, 127.7, 127.6, 127.1, 126.2, 126.2, 126.1, 99.1, 86.0, 80.9, 77.9, 77.5, 77.4, 77.2, 76.8, 75.4, 75.2, 75.1, 73.3, 72.9, 72.0, 69.8, 66.5, 64.2, 63.2, 62.3, 27.0, 20.8, 19.4. HR-ESI-MS *(m/z)*: $[M+Na]^+$ calcd for $C_{68}H_{69}N_3O_{11}SSiNa$, 1186.4320; found, 1186.4315.

N-benzyloxycarbonyl-3-aminopropyl-*O*-[2-azido-4-*O*-benzyl-6-*O*-*tert*-butyldiphenylsilyl-3-*O*-(2-naphthylmethyl)-2-deoxy-*α*-D-glucopyranosyl]-(1→4)-*O*-2-*O*-benzoyl-3-*O*-benzyl-6-*O*-acetyl-1-*α*-L- idopyranoside (2)

A solution of donor **1** (0.90 g, 0.42 mmol) and linker benzyl (3-hydroxypropyl) carbamate (0.097 g, 0.46 mmol) in CH_2Cl_2 (15 mL) was stirred under N_2 atmosphere in round bottom flask containing freshly dried 4 Å molecular sieves for 1 h. Next, NIS (0.15 g, 0.67 mmol) and TfOH (7.4 µL, 0.084 mmol) was added at room temperature and reaction completion was monitored by TLC, quenched using few drops of NEt_3. Molecular sieves were filtered using celite and organic layer was washed with aqueous $Na_2S_2O_3$ and brine. The collected organic layer was dried over Na_2SO_4, filtered, concentrated and purified through silica gel column chromatography (ethyl acetate/ hexane= 1/3, v/v) to obtain compound **2** in 87 % yield. ^{1}H NMR (400 MHz, Chloroform-d) δ 8.17 – 8.09 (m, 2H), 7.84 – 7.72 (m, 3H), 7.69 – 7.63 (m, 4H), 7.58 (s, 1H), 7.50 – 7.45 (m, 2H), 7.43 – 7.38 (m, 2H), 7.37 – 7.27 (m, 20H), 7.20 – 7.14 (m, 2H), 5.64 (t, J = 6.0 Hz, 1H), 5.07 (d, J = 6.9 Hz, 3H), 4.94 (d, J = 1.4 Hz, 1H), 4.81 (dd, J = 11.5, 8.4 Hz, 2H), 4.67 (t, J = 11.3 Hz, 2H), 4.57 (d, J = 10.6 Hz, 1H), 4.43 – 4.21 (m, 4H), 4.07 (dd, J = 10.9, 4.1 Hz, 1H), 4.04 – 3.95 (m, 2H), 3.84 (dd, J = 10.4, 5.7 Hz, 2H), 3.78 – 3.65 (m, 3H), 3.58 – 3.40 (m, 3H), 3.30 – 3.18 (m, 2H), 1.92 (s, 3H), 1.85 (p, J = 6.9, 5.8 Hz, 2H), 1.04 (s, 9H). ^{13}C NMR (101 MHz, CHLOROFORM-D) δ 170.6, 165.8, 156.7, 138.1, 137.6, 136.8, 136.0, 135.7, 135.2, 133.6, 133.4, 133.3, 133.1, 130.0, 129.9, 129.8, 128.7, 128.5, 128.4, 128.4, 128.2, 128.1, 128.1, 127.9, 127.8, 127.7, 127.7, 127.1, 126.2, 126.1, 98.8, 98.2, 80.8, 77.9, 77.4, 77.1, 76.8, 75.4, 75.2, 74.6, 73.1, 72.6, 72.0, 68.7, 67.1, 66.7, 65.3, 64.2, 63.4, 62.3, 39.8, 29.5, 26.9, 20.8, 19.4. HR-ESI-MS *(m/z)*: $[M+Na]^+$ calcd for $C_{73}H_{78}N_4O_{14}SSiNa$, 1285.5181; found, 1285.5174.

N-benzyloxycarbonyl-3-aminopropyl-*O*-[(methyl(2-azido-4-*O*-benzyl-6-*O*-*tert*-butyldiphenylsilyl-3-*O*-(2-naphthylmethyl)-2-deoxy-*α*-D-glucopyranosyl))]-(1→4)-*O*-3-*O*-benzyl-1-*α*- L-idopyranosyluronate (3)

A solution of compound **2** (1g, 1 eq) in DCM:MeOH (1:1, 14mL), NaOMe(1.2 eq) was added and kept it for stirring for 12 h at room temperature followed by quenching with IR Na^+ resin, filtered, evaporated and purified by silica gel column chromatography (ethyl acetate/ hexane= 1/2.5, v/v). After that compound was dissolved in DCM:H_2O (1:1) followed by addition by of TEMPO (0.2 eq) and BAIB (3 eq) and kept it stirring for 12 h at room temperature followed by extraction with saturated aqueous solution NH_4Cl. The organic layer was evaporated and purified by silica gel column chromatography. Then the compound was dissolved in THF:H_2O (1:1) followed by addition of lithium hydroxide (10 eq) and kept it on stirring for 2 h at room temperature followed by evaporation and dried over vacuum. After that reaction mixture was dissolved in dry DMF and potassium carbonate (3 eq), methyl iodide (9 eq) was added and kept it on stirring for at room temperature. After 12 h extraction was done using 10 % HCl solution and organic layer was evaporated, purified using silica gel chromatography using ethyl acetate/hexane (2/3) to obtain compound **3** 84%. ^{1}H NMR (400 MHz, Chloroform-*d*) δ 7.84 – 7.68 (m, 6H), 7.68 – 7.61 (m, 2H), 7.50 – 7.27 (m, 19H), 7.26 – 7.13 (m, 5H), 5.56 (d, *J* = 6.0 Hz, 1H), 5.04 (s, 2H), 5.00 (d, *J* = 10.6 Hz, 3H), 4.93 – 4.88 (m, 2H), 4.80 (s, 1H), 4.76 (d, *J* = 11.4 Hz, 1H), 4.60 – 4.56 (m, 2H), 4.12 4.11 (m, 1H), 3.97 (dd, *J* = 11.6, 2.9 Hz, 1H), 3.93 – 3.80 (m, 5H), 3.71 - 3.67(m, 1H), 3.63 (s, 3H), 3.61 – 3.54 (m, 3H), 3.54 – 3.49 (m, 1H), 3.44 (d, *J* = 5.5 Hz, 1H), 1.90 – 1.77 (m, 2H), 1.08 (s, 9H). ^{13}C NMR (101 MHz, $CDCl_3$) δ 169.8, 156.5, 138.3, 137.3, 136.6, 135.9, 135.6, 135.0, 133.4, 133.3, 133.1, 133.1, 129.8, 129.8, 128.7, 128.6, 128.4, 128.4, 128.3, 128.3, 128.1, 128.0, 127.9, 127.8, 127.7, 127.6, 127.6, 127.2, 127.2, 127.1, 126.2, 126.2, 126.1, 126.0, 101.5, 94.9, 81.1, 77.6, 77.4, 77.3, 77.1, 76.7, 76.1, 74.8, 72.8, 71.9, 70.8, 70.7, 68.0, 67.0, 66.9, 66.6, 63.9, 62.1, 53.4, 52.4, 39.9, 29.5, 26.9, 19.4. HR-ESI-MS *(m/z)*: $[M+Na]^+$ calcd for $C_{65}H_{72}N_4O_{13}SiNa$, 1167.4763; found, 1167.5478.

N-benzyloxycarbonyl-3-aminopropyl-*O*-[(methyl(2-azido-4-*O*-benzyl-6-*O*-*tert*-butyldiphenylsilyl-3-*O*-(2-naphthylmethyl)-2-deoxy-*α*-D-glucopyranosyl))]-(1→4)-*O*-2-*O*-acetyl-3-*O*-benzyl-1-*α*- L-idopyranosyluronate (4)

A solution of compound **3** (1g, 1 eq) in DCM:Py (1:1, 14mL), acetic anhydride (5 eq) and DMAP (0.2 eq) was added and kept it for stirring for 12 h at 0 ^{o}C followed by washing with 1N HCl 3 times and organic layer was evaporated and purified by silica gel column chromatography (ethyl acetate/ hexane= 1/4, v/v) to obtain **4**, 86 %. ^{1}H NMR (400 MHz, Chloroform-d) δ 7.87 –

7.72 (m, 4H), 7.70 - 7.68 (m, 2H), 7.64 – 7.62 (m, 2H), 7.52 – 7.43 (m, 3H), 7.42 – 7.26 (m, 17H), 7.26 – 7.14 (m, 3H), 5.44 (t, J = 5.7 Hz, 1H), 5.05 (s, 2H), 5.02 – 4.91 (m, 3H), 4.90 (s, 1H), 4.87 – 4.80 (m, 2H), 4.79 – 4.71 (m, 2H), 4.65 (m, 2H), 4.02 – 3.93 (m, 3H), 3.91 – 3.81(m, 4H), 3.76 – 3.68 (m, 1H), 3.60 – 3.52 (m, 4H), 3.43 – 3.31 (m, 2H), 3.28 – 3.20 (m, 1H), 2.07 (s, 3H), 1.86 – 1.73 (m, 2H), 1.06 (s, 8H). ^{13}C NMR (101 MHz, $CDCl_3$) δ 170.1, 169.4, 156.5, 138.3, 137.3, 136.6, 135.8, 135.6, 135.1, 133.4, 133.3, 133.1, 133.1, 129.7, 128.5, 128.4, 128.4, 128.4, 128.3, 128.2, 128.0, 128.0, 128.0, 127.8, 127.7, 127.7, 127.6, 127.6, 127.5, 127.2, 127.1, 127.0, 126.1, 126.0, 126.0, 98.3, 97.3, 79.9, 77.9, 77.3, 77.0, 76.7, 75.5, 74.7, 72.6, 72.4, 72.0, 71.4, 68.0, 67.8, 67.6, 66.6, 63.6, 62.2, 52.2, 39.5, 29.4, 26.9, 26.9, 20.8, 20.7, 19.4. HR-ESI-MS *(m/z)*: $[M+Na]^+$ calcd for $C_{67}H_{74}N_4O_{14}SiNa$, 1209.4868; found, 1209.4862.

N-benzyloxycarbonyl-3-aminopropyl-*O*-[(methyl(2-acetamido-4-*O*-benzyl-6-*O*-*tert*-butyldiphenylsilyl-3-*O*-(2-naphthylmethyl)-2-deoxy-*α*-D-glucopyranosyl))]-(1→4)-*O*-*2*-*O*-acetyl-3-*O*-benzyl-1-*α*- L-idopyranosyluronate (5)

A solution of compound **4** (1g, 1 eq) in THF:CH_3COOH:Ac_2O (3:2:1, 18mL), zinc dust (30 eq) was added and kept it for stirring for 12 h at room temperature followed by celite filtration and organic layer was evaporated. After that compound was re-dissolved in ethyl acetate and washed with 10 % sodium bicarbonate 3 times followed by evaporation of organic layer, purification by silica gel column chromatography (ethyl acetate/ hexane= 1/2, v/v) to obtain **5**, 81 %. ^{1}H NMR (400 MHz, Chloroform-d) δ 7.84 – 7.63 (m, 8H), 7.47 – 7.10 (m, 24H), 5.02 (s, 1H), 4.98 (d, J = 12.0 Hz, 1H), 4.92 (d, J = 11.2 Hz, 1H), 4.89 – 4.83 (m, 2H), 4.82 – 4.76 (m, 3H), 4.72 (d, J = 11.2 Hz, 1H), 4.48 (s, 1H), 4.37 (td, J = 10.1, 3.5 Hz, 1H), 4.05 (d, J = 2.9 Hz, 1H), 3.99 – 3.83 (m, 3H), 3.83 – 3.73 (m, 2H), 3.64 (s, 3H), 3.63 – 3.56 (m, 2H), 3.55 – 3.45 (m, 1H), 3.42 – 3.30 (m, 1H), 3.26 – 3.14 (m, 1H), 1.83 (s, 3H), 1.78 (m, 2H), 1.70 (s, 3H), 1.07 (s, 9H). ^{13}C NMR (101 MHz, $CDCl_3$) δ 170.2, 169.5, 169.4, 156.5, 138.5, 136.9, 136.6, 135.9, 135.8, 135.6, 135.6, 133.4, 133.3, 133.1, 133.0, 129.8, 128.6, 128.5, 128.5, 128.4, 128.4, 128.3, 128.3, 128.2, 128.1, 128.1, 128.0, 127.9, 127.9, 127.8, 127.7, 127.7, 127.6, 127.6, 127.4, 127.4, 127.4, 126.8, 126.2, 126.2, 126.1, 98.2, 95.3, 80.5, 77.9, 77.5, 77.2, 76.9, 75.2, 74.8, 73.1, 72.4, 68.0, 67.7, 67.7, 66.6, 62.6, 52.4, 52.2, 39.3, 29.4, 27.0, 26.9, 26.9, 23.3, 20.7, 19.4. HR-ESI-MS *(m/z)*: $[M+Na]^+$ calcd for $C_{69}H_{78}N_2O_{15}SiNa$, 1225.5069; found, 1225.5059.

N-benzyloxycarbonyl-3-aminopropyl-*O*-[(methyl(2-acetamido-4-*O*-benzyl-6-*O*-*tert*-butyldiphenylsilyl-3-*O*-sulfonato-2-deoxy-*α*-D-glucopyranosyl))]-(1→4)-*O-2-O*-acetyl-3-*O*-benzyl-1-*α*- L-idopyranosyluronate (7a)

To a solution of starting material **5** (1 mmol, 1 eq) in CH_2Cl_2 (6 mL) and H_2O (340 µL) was added DDQ (5 mmol, 5 eq) in 4 portions over the interval of 20 min. After 1 h, the reaction mixture was quenched using $NaHCO_3$, extracted with CH_2Cl_2 and washed with brine. The collected organic layer was dried over Na_2SO_4, filtered, concentrated and purified through silica gel column chromatography. After that, compound was dissolved to 1 mmol in DMF (2 mL) was added $SO_3.NEt_3$ complex (10 mmol per -OH) under N_2 atmosphere and stirred at 70 ^{o} C for 3 days. Upon completion of reaction, the reaction mixture was concentrated under reduced pressure and it was concentrated and purified using silica column chromatography (MeOH/DCM 1/10 v/v) to obtain product **7a** in 71 % yield. ^{1}H NMR (400 MHz, Methanol-d4) δ 7.82 – 7.66 (m, 4H), 7.50 – 7.24 (m, 21H), 5.39 – 5.22 (m, 2H), 5.14 – 5.02 (m, 3H), 5.00 – 4.89 (m, 2H), 4.82 – 4.59 (m, 4H), 4.23 – 4.09 (m, 2H), 4.04 – 3.81 (m, 6H), 3.71 (s, 3H), 3.57 (dd, J = 10.3, 5.4 Hz, 1H), 3.27 (dq, J = 19.7, 6.7 Hz, 2H), 2.17 (s, 3H), 2.03 (s, 3H), 1.89 – 1.73 (m, 2H), 1.12 (s, 9H). ^{13}C NMR (101 MHz, MeOD) δ 172.6, 170.6, 170.0, 157.3, 138.6, 137.6, 136.9, 135.6, 135.4, 133.3, 133.0, 129.6, 128.2, 128.1, 128.0, 128.0, 127.9, 127.7, 127.6, 127.5, 127.5, 127.5, 127.4, 127.2, 98.6, 96.4, 78.4, 76.1, 74.5, 73.0, 72.4, 72.2, 71.8, 68.3, 68.1, 66.5, 66.0, 62.7, 54.0, 53.8, 51.9, 48.5, 48.3, 48.1, 47.9, 47.7, 47.4, 47.2, 44.3, 38.0, 29.4, 29.3, 26.2, 22.0, 20.0, 18.8. HR-ESI-MS *(m/z)*: $[M]^-$ calcd for $C_{58}H_{69}N_2O_{18}SSi^-$, 1141.4041; found, 1141.4019.

N-benzyloxycarbonyl-3-aminopropyl-*O*-[(methyl(2-azido-4-*O*-benzyl-6-*O*-*tert*-butyldiphenylsilyl-3-*O*-sulfonato-2-deoxy-*α*-D-glucopyranosyl))]-(1→4)-*O-2-O*-acetyl-3-*O*-benzyl-1-*α*- L-idopyranosyluronate (13)

To a solution of starting material **4** (1 mmol, 1 eq) in CH_2Cl_2 (6 mL) and H_2O (340 µL) was added DDQ (5 mmol, 1 eq) in 4 portions over the interval of 20 min. After 1 h, the reaction mixture was quenched using $NaHCO_3$, extracted with CH_2Cl_2 and washed with brine. The collected organic layer was dried over Na_2SO_4, filtered, concentrated and purified through silica gel column chromatography. After that, compound was dissolved to 1 mmol in DMF (2 mL) was added $SO_3.NEt_3$ complex (10 mmol per -OH) under N_2 atmosphere and stirred at 70 ^{o} C for 3

days. Upon completion of reaction, the reaction mixture was concentrated under reduced pressure and it was concentrated and purified using silica column chromatography (MeOH/DCM 1/10 v/v) to obtain product **13** 77 % in yield. ^{1}H NMR (400 MHz, Methanol-d4) δ 7.79 – 7.73 (m, 4H), 7.47 – 7.28 (m, 4H), 5.35 (d, J = 10.7 Hz, 1H), 5.21 – 5.05 (m, 4H), 5.03 – 4.91 (m, 3H), 4.82 – 4.63 (m, 4H), 4.29 – 4.15 (m, 2H), 4.01 – 3.77 (m, 5H), 3.73 (s, 3H), 3.60 (m, 1H), 3.39 – 3.17 (m, 4H), 2.24 (s, 3H), 1.84 (q, J = 5.9 Hz, 2H). ^{13}C NMR (101 MHz, MeOD) δ 170.8, 170.3, 157.3, 138.5, 137.6, 136.9, 135.6, 135.4, 133.3, 133.1, 129.7, 128.3, 128.2, 128.2, 128.1, 128.0, 127.9, 127.8, 127.7, 127.6, 127.5, 127.5, 127.3, 98.5, 96.9, 78.7, 76.4, 74.6, 72.6, 72.3, 71.2, 68.1, 68.0, 66.6, 66.1, 62.8, 62.4, 51.9, 48.5, 48.3, 48.1, 47.9, 47.7, 47.5, 47.3, 46.6, 38.1, 29.4, 29.3, 26.3, 19.9, 18.9. HR-ESI-MS *(m/z)*: $[M]^-$ calcd for $C_{56}H_{65}N_4O_{17}SiS^-$, 1125.3840; found, 1125.3839.

N-benzyloxycarbonyl-3-aminopropyl-*O*-[(methyl(2-acetamido-4-*O*-benzyl-3-*O*-sulfonato-2-deoxy-*α*-D-glucopyranosyl))]-(1→4)-*O-2-O*-acetyl-3-*O*-benzyl-1-*α*- L-idopyranosyluronate (7b)

A solution of compound **7a** (1g, 1 eq) in dry pyridine (5 mL) 70% HF.Py complex (5 eq) was added at 0 ^{o}C and kept it for stirring for 12 h. After that in reaction mixture was evaporated followed by purification by silica gel column chromatography (MeOH/DCM = 1/10, v/v) to obtain **7b** in 85 % yield. ^{1}H NMR (400 MHz, Methanol-d4) δ 7.55 – 7.44 (m, 2H), 7.41 – 7.25 (m, 13H), 5.28 – 5.15 (m, 2H), 5.07 (d, J = 2.0 Hz, 2H), 5.00 (d, J = 2.7 Hz, 1H), 4.91 (d, J = 3.3 Hz, 1H), 4.77 – 4.66 (m, 3H), 4.59 (d, J = 10.5 Hz, 1H), 4.15 (t, J = 3.6 Hz, 1H), 4.04 – 3.90 (m, 2H), 3.84 (s, 4H), 3.81 (d, J = 2.1 Hz, 2H), 3.79 – 3.71 (m, 1H), 3.66 (t, J = 9.2 Hz, 1H), 3.60 – 3.50 (m, 1H), 3.23 (m, 2H), 2.13 (s, 3H), 1.94 (s, 3H), 1.87 – 1.72 (m, 2H). ^{13}C NMR (101 MHz, MeOD) δ 172.5, 170.7, 170.2, 157.4, 138.7, 137.8, 137.0, 128.4, 128.1, 127.8, 127.6, 127.5, 127.5, 127.5, 127.2, 98.7, 97.1, 77.8, 75.9, 74.5, 73.3, 72.9, 72.9, 72.2, 68.6, 68.3, 66.4, 66.0, 60.4, 53.9, 51.9, 48.6, 48.4, 48.2, 48.0, 47.8, 47.6, 47.3, 47.1, 37.9, 29.4, 29.3, 21.9, 21.8, 20.0. HR-ESI-MS *(m/z)*: $[M]^-$ calcd for $C_{42}H_{51}N_2O_{18}S^-$, 903.2863; found, 903.2857.

N-benzyloxycarbonyl-3-aminopropyl-*O*-[(methyl(2-azido-4-*O*-benzyl-3-*O*-sulfonato-2-deoxy-*α*-D-glucopyranosyl))]-(1→4)-*O-2-O*-acetyl-3-*O*-benzyl-1-*α*- L-idopyranosyluronate (17)

A solution of compound **13** (1g, 1eq) in dry pyridine (5 mL) 70% HF.Py complex (5 eq) was added at 0 ^{o}C and kept it for stirring for 12 h. After that in reaction mixture was evaporated followed by purification by silica gel column chromatography (MeOH/DCM = 1/10, v/v) to obtain **17** in 85 % yield. ^{1}H NMR (400 MHz, Methanol-d4) δ 7.51 – 7.45 (m, 2H), 7.40 – 7.25 (m, 13H), 5.24 (d, J = 10.4 Hz, 1H), 5.09 – 5.04 (m, 4H), 4.90 (d, J = 3.3 Hz, 1H), 4.84 (d, J = 1.8 Hz, 1H), 4.76 – 4.68 (m, 2H), 4.58 (d, J = 10.5 Hz, 2H), 4.20 (t, J = 3.6 Hz, 1H), 4.06 (t, J = 4.0 Hz, 1H), 3.81 (s, 3H), 3.81 – 3.76 (m, 3H), 3.74 – 3.50 (m, 3H), 3.30 – 3.16 (m, 3H), 2.17 (s, 3H), 1.84 – 1.73 (m, 2H). ^{13}C NMR (101 MHz, MeOD) δ 171.0, 170.4, 157.4, 138.6, 137.7, 136.9, 128.4, 128.3, 128.2, 128.2, 128.1, 127.9, 127.8, 127.7, 127.7, 127.6, 127.5, 127.5, 127.3, 98.7, 97.8, 78.6, 76.2, 74.5, 72.9, 72.4, 72.4, 68.4, 68.1, 66.4, 66.1, 62.3, 60.3, 51.9, 48.6, 48.4, 48.2, 48.0, 47.7, 47.5, 47.3, 47.1, 38.0, 29.3, 19.9. HR-ESI-MS *(m/z)*: $[M]^-$ calcd for $C_{40}H_{47}N_4O_{17}S^-$, 887.2668; found, 887.2657.

N-benzyloxycarbonyl-3-aminopropyl-*O*-[(methyl(2-acetamido-4-*O*-benzyl-3-*O*-(2-naphthylmethyl)-2-deoxy-*α*-D-glucopyranosyl))]-(1→4)-*O*-2-*O*-acetyl-3-*O*-benzyl-1-*α*- L-idopyranosyluronate (6)

A solution of compound **5** (1g, 1 eq) in dry pyridine (5 mL) 70% HF.Py complex (5 eq) was added at 0 ^{o}C and kept it for stirring for 12 h. After that in reaction mixture ethyl acetate was added and washed with 1N HCl 3 times followed by evaporation of organic layer, purification by silica gel column chromatography (ethyl acetate/ hexane= 2/3, v/v) to obtain **6**, 92 %. ^{1}H NMR (400 MHz, Chloroform-d) δ 7.84 – 7.67 (m, 4H), 7.49 – 7.42 (m, 2H), 7.40 – 7.26 (m, 12H), 7.26 – 7.16 (m, 4H), 5.91 – 5.80 (m, 1H), 5.46 (t, J = 5.8 Hz, 1H), 5.11 – 5.01 (m, 2H), 4.97 – 4.83 (m, 3H), 4.81 – 4.64 (m, 5H), 4.63 – 4.46 (m, 2H), 4.25 (td, J = 9.8, 3.6 Hz, 1H), 3.96 (q, J = 3.4, 2.9 Hz, 1H), 3.85 – 3.79 (m, 1H), 3.77 (s, 3H), 3.73 – 3.62 (m, 3H), 3.61 – 3.53 (m, 3H), 3.46 – 3.34 (m, 1H), 3.27 – 3.18 (m, 1H), 1.87 – 1.73 (m, 5H), 1.65 (s, 3H). ^{13}C NMR (101 MHz, $CDCl_3$) δ 169.9, 169.5, 169.4, 156.5, 138.1, 136.8, 136.6, 135.7, 133.2, 133.0, 128.7, 128.6, 128.5, 128.5, 128.5, 128.5, 128.4, 128.3, 128.3, 128.2, 128.1, 128.1, 128.0, 128.0, 127.9, 127.8, 127.7, 127.7, 127.6, 127.5, 126.6, 126.3, 126.1, 126.1, 126.0, 98.3, 95.7, 80.1, 77.8, 77.4, 77.1, 76.8, 75.0, 74.8, 72.6, 72.2, 70.3, 69.7, 68.0, 67.9, 67.5, 66.7, 61.5, 52.4, 52.3, 39.5, 29.4, 23.3, 20.7. HR-ESI-MS *(m/z)*: $[M+Na]^+$ calcd for $C_{53}H_{60}N_2O_{15}Na$, 987.3891; found, 987.3875.

N-benzyloxycarbonyl-3-aminopropyl-*O*-[(2-azido-4-*O*-benzyl-3-*O*-(2-naphthylmethyl)-2-deoxy-*α*-D-glucopyranosyl)]-(1→4)-*O*-3-*O*-benzyl-1-*α*- L-idopyranosyluronate (8)

A solution of compound **4** (1g, 1 eq) in dry pyridine (5 mL) 70% HF.Py complex (5 eq) was added at 0 °C and kept it for stirring for 12 h. After that reaction mixture was evaporated followed by purification by silica gel column chromatography (ethyl acetate/ hexane= 1/10, v/v) to obtain **10** in, 87 %. ^{1}H NMR (400 MHz, Chloroform-d) δ 7.84 – 7.70 (m, 4H), 7.48 – 7.41 (m, 3H), 7.39 – 7.26 (m, 11H), 7.25 (m, 3H), 5.57 (s, 1H), 5.05 (s, 1H), 5.01 (s, 1H), 4.97 (s, 2H), 4.87 – 4.82 (m, 2H), 4.69 – 4.59 (m, 2H), 4.50 (d, J = 12.3 Hz, 1H), 4.07 (s, 1H), 3.92 – 3.82 (m, 2H), 3.80 (s, 3H), 3.76 (d, J = 2.2 Hz, 1H), 3.74 – 3.64 (m, 3H), 3.64 – 3.57 (m, 2H), 3.54 – 3.52 (m, 2H), 3.49 – 3.43 (m, 2H), 3.30 – 3.20 (m, 1H), 1.90 – 1.77 (m, 2H). ^{13}C NMR (101 MHz, CHLOROFORM-D) δ 170.1, 156.6, 137.9, 137.3, 136.6, 135.0, 133.4, 133.1, 128.8, 128.6, 128.5, 128.4, 128.3, 128.2, 128.1, 128.1, 128.0, 128.0, 127.8, 127.7, 127.1, 126.2, 126.1, 126.1, 101.7, 95.0, 81.1, 77.4, 77.3, 77.1, 76.8, 76.0, 74.8, 72.3, 71.4, 71.1, 68.1, 66.9, 66.8, 63.8, 61.3, 52.8, 40.0, 29.5. HR-ESI-MS *(m/z)*: [M+Na] calcd for $C_{42}H_{48}N_4NaO_{13}$, 839.3116; found, 839.3110.

N-benzyloxycarbonyl-3-aminopropyl-*O*-[(methyl(2-acetamido-6-*O*-sulfonato-4-*O*-benzyl-3-*O*-(2-naphthylmethyl)-2-deoxy-*α*-D-glucopyranosyl))]-(1→4)-*O*-*2*-*O*-acetyl-3-*O*-benzyl-1-*α*-L-idopyranosyluronate (6a)

To a solution of starting material **6** (1 mmol, 1 eq) in DMF (2 mL) was added $SO_3.NEt_3$ complex (10 mmol per -OH, 10 eq) under N_2 atmosphere and stirred at 70 ° C for 3 days. Upon completion of reaction, the reaction mixture was concentrated under reduced pressure and it was concentrated and purified using silica column chromatography (MeOH/DCM 1/10 v/v) to obtain product **6a** 78 %. ^{1}H NMR (400 MHz, Methanol-d4) δ 7.86 – 7.78 (m, 2H), 7.78 – 7.71 (m, 1H), 7.52 – 7.45 (m, 2H), 7.44 – 7.26 (m, 16H), 5.08 (s, 2H), 4.99 – 4.96 (m, 3H), 4.94 – 4.93(m, 1H), 4.85 – 4.72 (m, 3H), 4.42 – 4.33 (m, 2H), 4.29 (dd, J = 10.2, 3.3 Hz, 1H), 4.24 (s, 1H), 4.01 (s, 1H), 3.92 (d, J = 8.7 Hz, 1H), 3.89 – 3.83 (m, 1H), 3.82 (s, 3H), 3.78 – 3.67 (m, 2H), 3.57 (m, 1H), 3.34 – 3.18 (m, 1H), 2.01 (s, 3H), 1.91 (s, 3H), 1.82 (m, 2 H). ^{13}C NMR (101 MHz, MeOD) δ 171.8, 170.2, 170.0, 157.4, 138.4, 137.6, 137.0, 135.9, 133.4, 133.1, 128.3, 128.1, 128.1, 127.9, 127.9, 127.7, 127.6, 127.6, 127.5, 127.5, 127.3, 127.3, 126.0, 125.8, 125.6, 125.5, 98.6, 94.8, 80.4, 77.8, 74.9, 74.6, 72.0, 70.8, 70.4, 69.2, 68.1, 67.7, 66.4, 66.0, 52.7, 51.7, 48.6, 48.4, 48.1,

47.9, 47.7, 47.5, 47.3, 47.1, 37.9, 29.3, 21.8, 19.7. HR-ESI-MS *(m/z)*: $[M+Na]^+$ calcd for $C_{53}H_{59}N_2O_{18}S^-$, 1043.3489; found, 1043.3485.

N-benzyloxycarbonyl-3-aminopropyl-*O*-[(methyl(2-azido-4-*O*-benzyl-6-*O*-sulfonato-3-*O*-(2-naphthylmethyl)-2-deoxy-*α*-D-glucopyranosyl))]-(1→4)-*O*-*2*-*O*-acetyl-3-*O*-benzyl-1-*α*- L-idopyranosyluronate (12)

A solution of compound **4** (1mmol, 1 eq) in dry pyridine (5 mL) 70% HF.Py complex (5 eq) was added at 0 °C and kept it for stirring for 12 h. After that reaction mixture was evaporated followed by purification by silica gel column chromatography (ethyl acetate/ hexane= 1/10, v/v). After that, compound was dissolved to (1 mmol, 1 eq) in DMF (2 mL) was added $SO_3.NEt_3$ complex (10 mmol per -OH) under N_2 atmosphere and stirred at 70 ° C for 3 days. Upon completion of reaction, the reaction mixture was concentrated under reduced pressure and it was concentrated and purified using silica column chromatography (MeOH/DCM 1/10 v/v) to obtain product **12** in 81% yield. ^{1}H NMR (400 MHz, Methanol-d4) δ 7.88 – 7.72 (m, 4H), 7.51 – 7.44 (m, 3H), 7.41 – 7.32 (m, 10H), 7.35 – 7.26 (m, 5H), 5.08 (s, 2H), 5.03 (d, J = 11.3 Hz, 1H), 5.01 – 4.93 (m, 3H), 4.95 – 4.86 (m, 2H), 4.82 (d, J = 10.8 Hz, 1H), 4.79 – 4.65 (m, 2H), 4.41 – 4.29 (m, 2H), 4.16 (t, J = 3.1 Hz, 1H), 3.98 (d, J = 3.2 Hz, 1H), 3.97 – 3.87 (m, 2H), 3.83 (m, 1H), 3.80 (s, 3H), 3.79 – 3.72 (m, 1H), 3.58 – 3.52 (m, 1H), 3.32 – 3.18 (m, 2H), 2.05 (s, 3H), 1.80 (m, 2H). ^{13}C NMR (101 MHz, MeOD) δ 170.3, 170.1, 157.4, 138.3, 137.6, 135.5, 133.3, 133.1, 128.1, 128.1, 127.9, 127.9, 127.8, 127.7, 127.6, 127.6, 127.5, 127.5, 127.4, 127.3, 127.3, 126.2, 125.8, 125.6, 125.6, 98.6, 97.5, 79.4, 77.7, 74.5, 74.5, 72.8, 72.0, 70.5, 68.0, 67.6, 66.3, 66.0, 65.5, 63.2, 51.7, 48.5, 48.3, 48.1, 47.9, 47.7, 47.4, 47.2, 47.0, 38.0, 29.2, 19.6. HR-ESI-MS *(m/z)*: $[M]^-$ calcd for $C_{51}H_{55}N_4O_{17}S^-$, 1027.3288; found, 1027.3275.

N-benzyloxycarbonyl-3-aminopropyl-*O*-[(methyl-(2-azido-4-*O*-benzyl-6-*O*-sulfonato-3-*O*-(2-naphthylmethyl)-2-deoxy-*α*-D-glucopyranosyl))]-(1→4)-*O*-2-*O*-sulfonato-3-*O*-benzyl-1-*α*-L-idopyranosyluronate (14)

The compound **4** was dissolved to (1 mmol, 1eq) in DMF (2 mL) was added $SO_3.NEt_3$ complex (10 mmol per -OH) under N_2 atmosphere and stirred at 70 ° C for 4 days. Upon completion of reaction, the reaction mixture was concentrated under reduced pressure and it was concentrated and purified using silica column chromatography (MeOH/DCM 1.3/10 v/v) to obtain product **14** in 76% yield. ^{1}H NMR (400 MHz, Methanol-d4) δ 7.91 – 7.73 (m, 4H), 7.54 – 7.19 (m, 18H),

5.22 (s, 1H), 5.15 – 4.98 (m, 5H), 4.80 (d, J = 10.9 Hz, 2H), 4.67 (d, J = 11.8 Hz, 2H), 4.52 (s, 1H), 4.37 – 4.25 (m, 3H), 4.14 (t, J = 2.8 Hz, 1H), 4.03 – 3.98 (m, 1H), 3.87 – 3.80 (m, 2H), 3.78 (s, 3H), 3.73 – 3.56 (m, 3H), 3.40 (dd, J = 10.3, 3.4 Hz, 1H), 3.33 – 3.17 (m, 2H), 1.89 – 1.77 (m, 2H). ^{13}C NMR (101 MHz, MeOD) δ 170.8, 157.5, 138.3, 137.7, 136.9, 135.8, 133.3, 133.1, 128.1, 128.1, 128.0, 127.9, 127.8, 127.7, 127.6, 127.6, 127.6, 127.5, 127.3, 127.2, 126.3, 125.8, 125.7, 125.6, 99.4, 96.4, 79.8, 77.6, 74.9, 74.5, 71.8, 71.3, 71.1, 71.0, 70.5, 67.1, 66.4, 66.1, 65.7, 63.5, 53.9, 51.9, 48.4, 48.2, 47.9, 47.7, 47.5, 47.3, 47.1, 38.1, 29.3, 29.2. HR-ESI-MS *(m/z)*: $[M]^{2-}$ calcd for $C_{42}H_{46}N_4O_{19}S_2^{2-}$, 487.1104; found, 487.1098.

N-benzyloxycarbonyl-3-aminopropyl-*O*-[(methyl-(2-azido-4-*O*-benzyl-6-*O*-sulfonato-3-*O*-sulfonato-2-deoxy-*α*-D-glucopyranosyl))]-(1→4)-*O*-2-*O*-sulfonato-3-*O*-benzyl-1-*α*- L-idopyranosyluronate (15)

To a solution of starting material **4** (1 mmol, 1 eq) in CH_2Cl_2 (6 mL) and H_2O (340 µL) was added DDQ (5 mmol) portion wise over the interval of 20 min. After 1 h, the reaction mixture was quenched using $NaHCO_3$, extracted with CH_2Cl_2 and washed with brine. The collected organic layer was dried over Na_2SO_4, filtered, concentrated and purified through silica gel column chromatography. After that, solution of product in dry pyridine (5 mL) 70% HF.Py complex (5 eq) was added at 0 ^{o}C and kept it for stirring for 12 h. After that in reaction mixture was evaporated followed by purification by silica gel column chromatography (ethyl acetate/hexane = 1/2, v/v). Then, compound was dissolved to 1 mmol in DMF (2 mL) was added SO_3.NEt_3 complex (10 mmol per -OH) under N_2 atmosphere and stirred at 70 ^{o} C for 4 days. Upon completion of reaction, the reaction mixture was concentrated under reduced pressure and it was concentrated and purified using silica column chromatography (MeOH/DCM 1.5/10 v/v) to obtain product **15** in 62% yield. ^{1}H NMR (400 MHz, Methanol-d4) δ 7.58 – 7.20 (m, 15H), 5.17 (s, 1H), 5.06 (dd, J = 6.5, 2.8 Hz, 2H), 4.92 (d, J = 10.6 Hz, 2H), 4.78 (d, J = 10.8 Hz, 2H), 4.72 – 4.60 (m, 2H), 4.46 (s, 1H), 4.27 – 4.24 (m, 2H), 4.17 – 4.08 (m, 1H), 4.03 – 3.86 (m, 1H), 3.89 – 3.72 (m, 2H), 3.75 (s, 3H), 3.32 – 3.11 (m, 3H), 1.85 – 1.80 (m, 2H). ^{13}C NMR (101 MHz, MeOD) δ 170.4, 157.4, 138.6, 138.4, 137.8, 136.9, 128.8, 128.2, 128.1, 128.1, 128.0, 128.0, 127.9, 127.8, 127.8, 127.8, 127.7, 127.5, 127.5, 127.3, 99.4, 96.3, 77.7, 74.6, 71.8, 71.5, 70.2, 67.2, 66.0, 63.5, 53.9, 51.7, 48.6, 48.4, 48.1, 47.9, 47.7, 47.5, 47.3, 47.1, 44.4, 44.3, 29.2. HR-ESI-MS *(m/z)*: $[M]^{2-}$ calcd for $C_{30}H_{37}N_4O_{20}S_3^{3-}$, 289.7060; found, 289.7047.

N-benzyloxycarbonyl-3-aminopropyl-*O*-[(2-sulfonatomido-4-*O*-benzyl-6-*O*-sulfonato-3-*O*-(2-naphthylmethyl)-2-deoxy-*α*-D-glucopyranosyl)]-(1→4)-*O*-3-*O*-benzyl-1-*α*- L-idopyranosyluronate (16)

A solution of compound **12** (0.6g, 1 eq) in THF:H_2O (1:1, 5 mL) LiOH.H_2O (20 eq) was added and kept it on stirring for room temperature. After 12 h reaction was quenched using IR H^+ resin and reaction fixture was evaporated and purified using reverse phase C-18 column chromatography using (MeOH/H_2O 1/9 v/v) to obtain compound **deacetylated product**. A solution of compound **deactylated product** (0.6g, 1 eq) in THF (3 mL) 1M PMe_3.THF (8 eq) and 0.1M aqueous NaOH (10eq) was added and kept it on stirring for room temperature. After 12 h reaction was quenched using glacial acetic acid and reaction mixture solvent was evaporated and purified using reverse phase C-18 column chromatography using (MeOH/H_2O 2/3 v/v), followed by passing through DOWEX 50WX8 Na^+ resin column to obtain compound **aminated product** in 76 % yield. A solution of compound **aminated product** (0.2g, 1 eq) in MeOH (3 mL) 0.1M NaOH (4 eq), triethylamine (8 eq), 10 eq of SO_3.Py was added at an interval of 1 h for 6 times by maintain 9-10 pH of reaction mixture and kept it on stirring for room temperature. The progress of reaction was monitored using TLC plate (ethyl acetate/pyridine/water/acetic acid 8/5/3/1 v/v/v/v). After completion, reaction mixture solvent was evaporated and purified using reverse phase C-18 column chromatography using (MeOH/H_2O 2/3 v/v), followed by passing through DOWEX 50WX8 Na^+ resin column to obtain compound **39** in 64 % yield. ^{1}H NMR (400 MHz, Methanol-d_4) δ 7.86 – 7.74 (m, 4H), 7.60 – 7.58 (m, 1H), 7.43 – 7.36 (m, 4H), 7.32 – 7.24 (m, 9H), 7.22 – 7.19 (m, 3H), 5.38 (d, J = 3.9 Hz, 1H), 5.27 (d, J = 11.3 Hz, 1H), 5.03 (s, 2H), 4.93 (d, J = 10.1 Hz, 2H), 4.83 – 4.73 (m, 3H), 4.65 (q, J = 11.6 Hz, 2H), 4.43 – 4.23 (m, 1H), 4.25 (d, J = 10.8, 1H), 4.17 (d, J = 6.7 Hz, 2H), 3.94 – 3.86 (m, 2H), 3.84 – 3.73 (m, 2H), 3.73 – 3.65 (m, 1H), 3.63 – 3.48 (m, 2H), 3.29 – 3.15 (m, 2H), 1.82 – 1.74 (m, 2H). ^{13}C NMR (101 MHz, MeOD) δ 172.9, 157.4, 138.5, 138.2, 136.9, 136.7, 133.4, 133.0, 128.1, 128.0, 128.0, 127.8, 127.7, 127.7, 127.6, 127.5, 127.4, 127.3, 127.2, 127.1, 126.2, 126.2, 125.5, 125.3, 101.2, 96.5, 80.6, 77.3, 75.0, 74.6, 73.1, 71.8, 69.9, 66.7, 66.1, 65.9, 58.2, 48.3, 48.1, 47.9, 47.7, 47.5, 47.3, 47.0, 38.3, 29.3. HR-ESI-MS *(m/z)*: $[M]^-$ calcd for $C_{48}H_{52}N_2O_{19}S^{2-}$, 512.1308; found, 512.1297.

N-benzyloxycarbonyl-3-aminopropyl-*O*-[((2-sulfonatomido-4-*O*-benzyl-3-*O*-sulfonato-2-deoxy-*α*-D-glucopyranosyl))]-(1→4)-*O*-3-*O*-benzyl-1-*α*- L-idopyranosyluronate (17)

A solution of compound **13** (0.6g, 1 eq) in THF:H_2O (1:1, 5 mL) LiOH.H_2O (20 eq) was added and kept it on stirring for room temperature. After 12 h reaction was quenched using IR H^+ resin and reaction fixture was evaporated and purified using reverse phase C-18 column chromatography using (MeOH/H_2O 1/9 v/v) to obtain deacetylated product. A solution of **deactylated product** in THF (3 mL) 1M PMe_3.THF (8 eq) and 0.1M aqueous NaOH (10eq) was added and kept it on stirring for room temperature. After 12 h reaction was quenched using glacial acetic acid and reaction mixture solvent was evaporated and purified using reverse phase C-18 column chromatography using (MeOH/H_2O 2/3 v/v), followed by passing through DOWEX 50WX8 Na^+ resin column to obtain **amine product** in 78 % yield. A solution of compound **amine product** (0.2g, 1 eq) in MeOH (3 mL) 0.1M NaOH (4 eq), triethylamine (8 eq), 10 eq of SO_3.Py was added at an interval of 1 h for 6 times by maintain 9-10 pH of reaction mixture and kept it on stirring for room temperature. The progress of reaction was monitored using TLC plate (ethyl acetate/pyridine/water/acetic acid 8/5/3/1 v/v/v/v). After completion, reaction mixture solvent was evaporated and purified using reverse phase C-18 column chromatography using (MeOH/H_2O 2/3 v/v), followed by passing through DOWEX 50WX8 Na^+ resin column to obtain compound **37** in 62 % yield. ^{1}H NMR (400 MHz, Methanol-d_4) δ 7.50 – 7.21 (m, 15H), 5.28 (d, J = 3.8 Hz, 1H), 5.04 (s, 2H), 4.96 (d, J = 11.0 Hz, 2H), 4.68 – 4.62 (m, 4H), 4.24 – 4.04 (m, 2H), 3.89 – 3.72 (m, 5H), 3.74 – 5.71 (m, 1H), 3.58 – 3.46 (m, 2H), 3.33 – 3.11 (m, 3H), 1.80 (p, J = 6.1 Hz, H). ^{13}C NMR (151 MHz, MeOD) δ 174.0, 157.4, 138.6, 138.2, 136.9, 128.4, 128.1, 127.9, 127.9, 127.9, 127.6, 127.6, 127.5, 127.5, 127.3, 127.2, 101.0, 95.9, 78.3, 74.3, 73.1, 72.2, 71.7, 71.6, 71.2, 67.2, 66.1, 60.7, 58.4, 53.9, 48.1, 48.0, 47.8, 47.7, 47.5, 47.4, 47.3, 38.4, 29.3. HR-ESI-MS *(m/z)*: $[M]^{2-}$ calcd for $C_{53}H_{62}N_2O_{19}SSi^{2-}$, 561.1584; found, 561.1579.

N-benzyloxycarbonyl-3-aminopropyl-*O*-[((2-sulfonatomido-4-*O*-benzyl-6-*O*-sulfonato-2-deoxy-*α*-D-glucopyranosyl))]-(1→4)-*O*-2-*O*-sulfonato-3-*O*-benzyl-1-*α*- L-idopyranosyluronate (18)

A solution of compound **14** (0.2g, 1 eq) in THF:H_2O (1:1, 5 mL) LiOH.H_2O (20 eq) was added and kept it on stirring for room temperature. After 12 h reaction was quenched using IR H^+ resin

and reaction fixture was evaporated and purified using reverse phase C-18 column chromatography using ($MeOH/H_2O$ 1/9 v/v). A solution of compound 46a in THF (3 mL) 1M PMe_3.THF (8 eq) and 0.1M aqueous NaOH (10eq) was added and kept it on stirring for room temperature. After 12 h reaction was quenched using glacial acetic acid and reaction mixture solvent was evaporated and purified using reverse phase C-18 column chromatography using ($MeOH/H_2O$ 2/3 v/v), followed by passing through DOWEX 50WX8 Na^+ resin column. Then, a solution of compound 46b in MeOH (3 mL) 0.1M NaOH (4 eq), triethylamine (8 eq), 10 eq of SO_3.Py was added at an interval of 1 h for 6 times by maintain 9-10 pH of reaction mixture and kept it on stirring for room temperature. The progress of reaction was monitored using TLC plate (ethyl acetate/pyridine/water/acetic acid 8/5/3/1 v/v/v/v). After completion, reaction mixture solvent was evaporated and purified using reverse phase C-18 column chromatography using ($MeOH/H_2O$ 2/3 v/v), followed by passing through DOWEX 50WX8 Na^+ resin column to obtain **40** in 58 % yield. ^1H NMR (400 MHz, Methanol-d_4) δ 7.94 – 7.74 (m, 4H), 7.64 (d, J = 8.4 Hz, 1H), 7.53 – 7.14 (m, 17H), 5.36 (d, J = 11.6 Hz, 2H), 5.03 (s, 3H), 4.61 – 4.54 (m, 5H),4.45 – 4.34 (m, 3H), 4.47 – 4.07 (m, 3H), 3.91 – 3.72 (m, 2H), 3.70 3.54(m, 4H), 3.24 – 3.15 (m, 2H), 1.80 – 1.74 (m, 2H). ^{13}C NMR (101 MHz, MeOD) δ 174.6, 157.4, 138.3, 138.2, 136.9, 133.4, 133.0, 128.3, 128.1, 128.0, 127.9, 127.8, 127.6, 127.6, 127.5, 127.4, 127.3, 127.3, 127.2, 126.3, 126.3, 126.3, 125.5, 125.4, 98.8, 98.7, 80.5, 77.3, 75.0, 74.4, 73.8, 71.7, 70.8, 69.7, 67.2, 66.0, 65.8, 53.9, 48.4, 48.2, 48.0, 47.7, 47.5, 47.3, 47.1, 38.0, 29.3. HR-ESI-MS *(m/z)*: $[M]^{3-}$ calcd for $C_{42}H_{47}N_2O_{22}S_3^{3-}$, 321.0495; found, 321.0484.

N-benzyloxycarbonyl-3-aminopropyl-*O*-[((2-sulfonatomido-4-*O*-benzyl-6-*O*-sulfonato-3-*O*-sulfonato-2-deoxy-*α*-D-glucopyranosyl))]-(1→4)-*O*-2-*O*-sulfonato-3-*O*-benzyl-1-*α*- L-idopyranosyluronate (19)

A solution of compound **15** (0.2g, 1eq) in THF:H_2O (1:1, 5 mL) $LiOH.H_2O$ (20 eq) was added and kept it on stirring for room temperature. After 12 h reaction was quenched using IR H^+ resin and reaction fixture was evaporated and purified using reverse phase C-18 column chromatography using ($MeOH/H_2O$ 1/9 v/v). A solution of compound **41a** in THF (3 mL) 1M PMe_3.THF (8 eq) and 0.1M aqueous NaOH (10eq) was added and kept it on stirring for room temperature. After 12 h reaction was quenched using glacial acetic acid and reaction mixture solvent was evaporated and purified using reverse phase C-18 column chromatography using

($MeOH/H_2O$ 2/3 v/v), followed by passing through DOWEX 50WX8 Na^+ resin column. Then, a solution of compound **41b** in MeOH (3 mL) 0.1M NaOH (4 eq), triethylamine (8 eq), 10 eq of SO_3.Py was added at an interval of 1 h for 6 times by maintain 9-10 pH of reaction mixture and kept it on stirring for room temperature. The progress of reaction was monitored using TLC plate (ethyl acetate/pyridine/water/acetic acid 8/5/3/1 v/v/v/v). After completion, reaction mixture solvent was evaporated and purified using reverse phase C-18 column chromatography using ($MeOH/H_2O$ 2/3 v/v), followed by passing through DOWEX 50WX8 Na^+ resin column to obtain compound **41** in 55 % yield. ^1H NMR (400 MHz, Methanol-d4) δ 7.55 (d, J = 7.4 Hz, 2H), 7.46 (d, J = 7.6 Hz, 2H), 7.43 – 7.26 (m, 11H), 5.34 (d, J = 15.4 Hz, 2H), 5.10 (s, 2H), 4.77 (d, J = 6.3 Hz, 2H), 4.71 – 4.60 (m, 3H), 4.57 (s, 1H), 4.49 (d, J = 10.9 Hz, 1H), 4.44 – 4.18 (m, 4H), 4.10 (d, J = 10.1 Hz, 1H), 3.93 – 3.77 (m, 2H), 3.72 – 3.61 (m, 2H), 3.31 – 3.17 (m, 2H), 1.89 – 1.80 (m, 2H). ^{13}C NMR (101 MHz, MeOD) δ 157.5, 138.4, 138.1, 136.9, 129.6, 129.5, 128.5, 128.1, 128.1, 127.9, 127.7, 127.5, 127.5, 127.4, 127.2, 98.6, 97.7, 78.1, 74.8, 73.4, 72.0, 71.7, 70.9, 69.5, 66.1, 65.9, 58.5, 48.4, 48.2, 47.9, 47.7, 47.5, 47.3, 47.1, 46.3, 38.1, 29.2. HR-ESI-MS *(m/z)*: $[M]^{2-}$ calcd for $C_{37}H_{42}N_2O_{55}S_4^{4-}$, 380.4864; found, 380.4858.

3-aminopropyl-*O*-[(2-acetamido-3-*O*-sulfonato-*α*-D-glucopyranosyl)-(1→4)-*O*-*α*-L-idopyranosiduronate (I-2)

A solution of compound **7b** (60 mg, 1 eq) in H_2O (2 mL) $LiOH.H_2O$ (20 eq) was added and kept it on stirring for room temperature. After 12 h reaction was quenched using IR H^+ resin and reaction fixture was evaporated and purified using reverse phase C-18 column chromatography using ($MeOH/H_2O$ 1/9 v/v). The reaction mixture was dissolved in water and $Pd(OH)_2$ was added and stirred under H_2 atm. After 48 h, the mixture was filtered, concentrated and eluted through Bond Elute C-18 column using H_2O as eluent. The combined H_2O fraction were pooled and lyophilized to yield fully deprotected 3-O-sulfated NAc-disaccharide. ^1H NMR (400 MHz, Deuterium Oxide) δ 5.17 (d, J = 3.7 Hz, 1H), 4.91 (d, J = 2.7 Hz, 1H), 4.51 (d, J = 2.6 Hz, 1H), 4.44 (dd, J = 10.5, 9.0 Hz, 1H), 4.13 – 4.05 (m, 2H), 4.02 (t, J = 4.1 Hz, 1H), 3.95 – 3.89 (m, 1H), 3.89 – 3.82 (m, 3H), 3.73 (dt, J = 10.6, 5.6 Hz, 1H), 3.69 – 3.62 (m, 2H), 3.15 (td, J = 6.7, 1.4 Hz, 2H), 2.00 (s, 5H). ^{13}C NMR (151 MHz, D_2O) δ 175.1, 174.3, 100.4, 94.9, 79.8, 73.7, 71.8, 68.7, 68.3, 68.2, 67.5, 66.4, 59.9, 52.1, 38.1, 26.3, 22.0. HR-ESI-MS *(m/z)*: $[M]^-$ calcd for $C_{17}H_{29}N_2O_{15}S^-$, 533.1294; found, 533.1262.

3-aminopropyl-*O*-[(2-acetamido-6-*O*-sulfonato-*α*-D-glucopyranosyl)-(1→4)-*O*-*α*-L-idopyranosiduronate (I-1)

A solution of compound **19** (50 mg, 1 eq) in THF:H_2O (1:1, 5 mL) LiOH.H_2O (20 eq) was added and kept it on stirring for room temperature. After 12 h reaction was quenched using IR H^+ resin and reaction fixture was evaporated and purified using reverse phase C-18 column chromatography using (MeOH/H_2O 1/9 v/v). The reaction mixture was dissolved in water and $Pd(OH)_2$ was added and stirred under H_2 atm. After 48 h, the mixture was filtered, concentrated and eluted through Bond Elute C-18 column using H_2O as eluent. The combined H_2O fraction was pooled and lyophilized to yield fully deprotected 6-O-sulfated NAc-disaccharide. ^{1}H NMR (400 MHz, Deuterium Oxide) δ 5.06 (d, J = 3.7 Hz, 1H), 4.81 (d, J = 2.6 Hz, 1H), 4.44 (d, J = 2.6 Hz, 1H), 4.25 (dd, J = 11.1, 3.4 Hz, 1H), 4.12 (dd, J = 11.0, 2.1 Hz, 1H), 3.98 (t, J = 2.9 Hz, 1H), 3.92 (t, J = 4.0 Hz, 1H), 3.89 (d, J = 3.5 Hz, 1H), 3.86 (d, J = 3.6 Hz, 1H), 3.84 – 3.78 (m, 1H), 3.66 – 3.60 (m, 2H), 3.58 (dd, J = 4.7, 2.6 Hz, 1H), 3.48 (t, J = 9.6 Hz, 1H), 3.10 – 3.02 (m, 2H), 1.95 – 1.87 (m, 5H). ^{13}C NMR (101 MHz, D_2O) δ 175.0, 174.3, 100.4, 94.3, 72.9, 71.0, 70.1, 69.1, 68.5, 68.2, 67.0, 66.5, 53.3, 38.3, 26.3, 21.9. HR-ESI-MS *(m/z)*: $[M]^-$ calcd for $C_{17}H_{29}N_2O_{15}S^-$, 533.1294; found, 533.1291.

3-aminopropyl-*O*-[(2-sulfonatomido-3-*O*-sulfonato-*α*-D-glucopyranosyl)-(1→4)-*O*-*α*-L-idopyranosiduronate (I-4)

A solution of compound **38** in H_2O and $Pd(OH)_2$ was added and stirred under H_2 atm. After 48 h, the mixture was filtered, concentrated and eluted through Bond Elute C-18 column using H_2O as eluent. The combined H_2O fraction was pooled and lyophilized to yield fully deprotected NS-disaccharide. ^{1}H NMR (600 MHz, Deuterium Oxide) δ 5.33 (d, J = 3.7 Hz, 1H), 4.93 (s, 1H), 4.66 (d, J = 2.5 Hz, 1H), 4.20 (t, J = 3.5 Hz, 1H), 4.07 (d, J = 2.6 Hz, 1H), 3.95 – 3.88 (m, 1H), 3.84 – 3.74 (3, 2H), 3.75 (d, J = 3.7 Hz, 1H), 3.74 – 3.65 (m, 2H), 3.63 – 3.60 (m, 1H), 3.51 (t, J = 9.6 Hz, 1H), 3.24 – 3.12 (m, 3H), 2.03 – 1.96 (m, 2H). ^{13}C NMR (151 MHz, D_2O) δ 174.2, 100.5, 96.1, 74.1, 72.0, 71.2, 69.4, 67.8, 67.2, 66.6, 66.6, 60.0, 57.7, 55.4, 38.3, 26.2. HR-ESI-MS *(m/z)*: $[M]^{2-}$ calcd for $C_{15}H_{26}N_2O_{17}S^{2-}$, 285.0684; found, 285.0673.

3-aminopropyl-*O*-[(2-sulfonatomido-6-*O*-sulfonato-*α*-D-glucopyranosyl)-(1→4)-*O*-*α*-L-idopyranosiduronate (I-3)

A solution of compound **39** in H_2O and $Pd(OH)_2$ was added and stirred under H2 atm. After 48 h, the mixture was filtered, concentrated and eluted through Bond Elute C-18 column using H2O as eluent. The combined H2O fraction was pooled and lyophilized to yield fully deprotected NS-disaccharide. ^1H NMR (400 MHz, Deuterium Oxide) δ 5.34 (d, J = 3.7 Hz, 1H), 4.97 – 4.85 (m, 1H), 4.60 (d, J = 2.4 Hz, 1H), 4.34 (dd, J = 11.0, 3.1 Hz, 1H), 4.27 – 4.11 (m, 2H), 4.08 (t, J = 2.8 Hz, 1H), 3.97 – 3.83 (m, 2H), 3.78 – 3.68 (m, 2H), 3.66 – 3.54 (m, 2H), 3.25 (dd, J = 10.0, 3.6 Hz, 1H), 3.21 – 3.11 (m, 2H), 2.04 – 1.98 (m, 2H). ^{13}C NMR (151 MHz, D_2O) δ 174.6, 100.4, 95.8, 74.2, 74.1, 71.1, 70.1, 69.1, 68.1, 68.0, 67.5, 67.5, 66.9, 66.8, 66.6, 66.5, 66.3, 57.6, 50.8, 43.2, 38.3, 26.2, 25.6. HR-ESI-MS (m/z): [M]2$^-$ calcd for C15H26N2O17S2-, 285.0684; found, 285.0671.

3-aminopropyl-*O*-[(2-sulfonatomido-6-*O*-sulfonato-*α*-D-glucopyranosyl)-(1→4)-*O*-2-*O*-sulfonato *O*-*α*-L-idopyranosiduronate (I-5)

A solution of compound **40** in H_2O and $Pd(OH)_2$ was added and stirred under H_2 atm. After 48 h, the mixture was filtered, concentrated and eluted through Bond Elute C-18 column using H_2O as eluent. The combined H_2O fraction was pooled and lyophilized to yield fully deprotected NS-disaccharide. ^1H NMR (400 MHz, Deuterium Oxide) δ 5.32 (d, J = 3.5 Hz, 1H), 5.08 (d, J = 2.6 Hz, 1H), 4.48 (d, J = 2.7 Hz, 1H), 4.28 (dd, J = 11.0, 2.8 Hz, 1H), 4.24 – 4.11 (m, 3H), 4.03 (t, J = 3.1 Hz, 1H), 3.91 – 3.84 (m, 2H), 3.67 – 3.60 (m, 1H), 3.59 – 3.46 (m, 2H), 3.19 (dd, J = 10.2, 3.6 Hz, 1H), 3.10 (t, J = 6.5 Hz, 2H), 1.97 – 1.90 (m, 2H). ^{13}C NMR (101 MHz, D_2O) δ 176.9, 101.2, 99.7, 78.4, 78.2, 73.5, 72.6, 71.7, 70.9, 70.8, 69.2, 69.0, 60.4, 40.9, 28.7.HR-ESI-MS *(m/z)*: $[M]^{2-}$ calcd for $C_{16}H_{27}N_2O_{20}S_3^{3-}$, 221.0112; found, 221.0195.

3-aminopropyl-*O*-[(2-sulfonatomido-6-*O*-sulfonato-3-*O*-sulfonato-*α*-D-glucopyranosyl)-(1→4)-*O*-2-*O*-sulfonato *O*-*α*-L-idopyranosiduronate (I-6)

A solution of compound **41** in H_2O and $Pd(OH)_2$ was added and stirred under H_2 atm. After 48 h, the mixture was filtered, concentrated and eluted through Bond Elute C-18 column using H_2O as eluent. The combined H_2O fraction was pooled and lyophilized to yield fully deprotected NS-disaccharide. ^1H NMR (400 MHz, Deuterium Oxide) δ 5.34 (d, J = 3.7 Hz, 1H), 4.97 – 4.85 (m, 1H), 4.60 (d, J = 2.4 Hz, 1H), 4.34 (dd, J = 11.0, 3.1 Hz, 1H), 4.27 – 4.11 (m, 2H), 4.08 (t, J = 2.8 Hz, 1H), 3.97 – 3.83 (m, 2H), 3.78 – 3.68 (m, 2H), 3.66 – 3.54 (m, 2H), 3.25 (dd, J = 10.0, 3.6 Hz, 1H), 3.21 – 3.11 (m, 2H), 2.04 – 1.98 (m, 2H). ^{13}C NMR (151 MHz, D_2O) δ 174.6,

100.4, 95.8, 74.2, 74.1, 71.1, 70.1, 69.1, 68.1, 68.0, 67.5, 67.5, 66.9, 66.8, 66.6, 66.5, 66.3, 57.6, 50.8, 43.2, 38.3, 26.2, 25.6. HR-ESI-MS *(m/z)*: $[M]^{2-}$ calcd for $C_{15}H_{26}N_2O_{17}S^{2-}$, 285.0684; found, 285.0671.

3. Synthesis of Glucuronic acid based Disaccharides

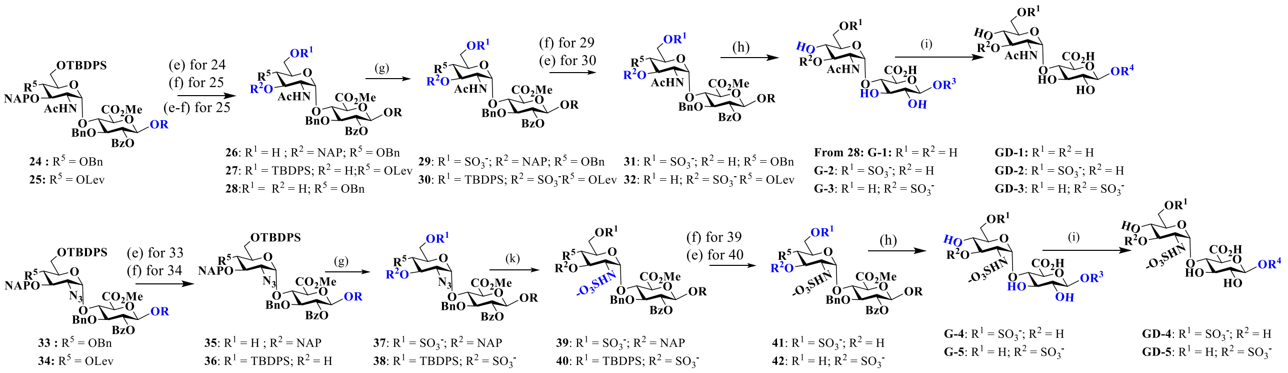

Scheme S2. Synthesis of glucuronic acid based HS disaccharides (a) R = Linker, NIS, TMSOTf, DCM, -10 °C, 15 min; (b) NaOMe, MeOH, RT, 12 h; TEMPO, BAIB, DCM:H_2O (1:1), RT; (c) 1 M LiOH, THF: H_2O (1:1), RT, 2 h; MeI, K_2CO_3, DMF, 12 h; Ac_2O, DCM:Py (1:1), 0 °C, 12 h; (d) Zn dust, THF:AcOH:Ac_2O (3:2:1), RT, 12 h; (e) HF.Py, Py, 0 °C, 12 h; (f)DDQ, DCM:H_2O (18:1), RT, 1 h; (g) SO_3. Et_3N, DMF, 60 °C, 24 h; (h) (i) LiOH.H_2O, H_2O, 12 h; (ii) H_2, $Pd(OH)_2$, MeOH. 48 h; (i) DBCO-NHS linker, Et_3N, DMF, 24 h. (j) LiOH.H_2O, H_2O; (k) PMe_3.THF, 24 h; (ii) SO_3.Py. MeOH, 1N NaOH, 48 h.

N-benzyloxycarbonyl-3-aminopropyl-*O*-[(2-acetamido-6-*O*-sulfonato-4-*O*-benzyl-3-*O*-(2-naphthylmethyl)-2-deoxy-*α*-D-glucopyranosyl)]-(1→4)-*O*-3-*O*-benzyl-1-β-D-glucopyranosiduronate (29)

Compound **24** (1g, 1 eq) was dissolved in dry pyridine (5 mL), and a 70 % HF.Py complex (5 eq) was added at 0°C. The mixture was stirred for 12 hours. Subsequently, ethyl acetate was added to the reaction mixture and washed with 1 N HCl three times. The organic layer was then evaporated, and purification was carried out using silica gel column chromatography. Then sulfation reaction was performed as mentioned for compound **6** followed by ester hydrolysis was

performed using LiOH.H_2O (20 eq) in MeOH:H_2O (1:1) for 12 h to obtain compound **29**. ^{1}H NMR (400 MHz, Methanol-d4) δ 7.18 – 6.88 (m, 22H), 5.26 (d, J = 3.5 Hz, 1H), 4.81 (s, 1H), 4.77 (s, 2H), 4.72 (d, J = 11.5 Hz, 1H), 4.66 – 4.58 (m, 2H), 4.49 (d, J = 10.2 Hz, 1H), 4.37 (d, J = 10.5 Hz, 1H), 4.24 (d, J = 10.1 Hz, 1H), 4.13 – 3.94 (m, 4H), 3.74 (q, J = 10.6, 10.0 Hz, 2H), 3.67 – 3.52 (m, 3H), 3.52 – 3.42 (m, 1H), 3.37 – 3.17 (m, 2H), 3.01 – 2.89 (m, 2H), 1.62 (s, 3H), 1.53 – 1.42 (m, 2H). ^{13}C NMR (101 MHz, MeOD) δ 175.1, 171.8, 157.8, 138.4, 138.4, 137.2, 136.9, 136.3, 133.4, 133.0, 130.3, 129.1, 128.3, 128.2, 128.1, 128.0, 127.8, 127.7, 127.6, 127.6, 127.5, 127.4, 126.0, 125.9, 125.7, 125.6, 102.9, 97.5, 84.9, 80.7, 78.1, 76.9, 75.3, 74.7, 74.3, 74.0, 69.9, 67.0, 66.3, 66.0, 53.1, 48.8, 48.6, 48.4, 48.2, 48.0, 47.8, 47.6, 47.3, 37.4, 29.7, 22.0. HR-ESI-MS *(m/z)*: $[M]^-$ calcd for $C_{58}H_{61}N_2O_{18}S^-$, 1105.3646; found, 1105.3639.

N-benzyloxycarbonyl-3-aminopropyl-*O*-[(2-acetamido-6-*O*-*tert*-butyldiphenylsilyl-3-*O*-sulfonato-2-deoxy-*α*-D-glucopyranosyl)]-(1→4)-*O*-3-*O*-benzyl-1-β-D-glucopyranosiduronate (28)

A solution compound **24** containing (1 g, 1 eq) of starting material in 6 mL of CH_2Cl_2 and 340 µL of H_2O was prepared, to which DDQ (5 mmol, 5 eq) was gradually added over a 20-minute period. After 1 hour, the reaction mixture was quenched with $NaHCO_3$, extracted with CH_2Cl_2, and washed with brine. The organic layer obtained was dried over Na_2SO_4, filtered, concentrated, and subjected to purification through silica gel column chromatography. Subsequently, the compound was dissolved in 2 mL of DMF, and SO_3.NEt_3 complex (10 mmol per -OH, 10 eq) was added under a N_2 atmosphere. The mixture was stirred at 70 °C for 3 days. After the reaction completion, the mixture was concentrated under reduced pressure, and the resulting product was purified using silica column chromatography (MeOH/DCM 1/10 v/v) to get product. Then, TBDPS was deprotected when the reaction mixture was stirred for 12 h in presence of 70 % HF.Py, in pyridine followed by ester hydrolysis was performed using LiOH.H_2O (20 eq) in MeOH:H_2O (1:1) for 12 h to obtain compound **28**. ^{1}H NMR (400 MHz, Methanol-d4) δ 7.38 – 7.18 (m, 10H), 5.44 (d, J = 3.9 Hz, 1H), 5.04 (d, J = 15.7 Hz, 3H), 4.65 (d, J = 10.6 Hz, 1H), 4.50 (dd, J = 10.7, 8.9 Hz, 1H), 4.31 (d, J = 7.7 Hz, 1H), 4.06 (ddd, J = 10.6, 6.5, 3.8 Hz, 1H), 4.00 – 3.87 (m, 2H), 3.80 (dd, J = 21.6, 10.5 Hz, 3H), 3.68 (d, J = 8.8 Hz, 2H), 3.61 – 3.52 (m, 2H), 3.48 (dd, J = 8.9, 7.8 Hz, 1H), 3.24 (dt, J = 8.9, 6.6 Hz, 2H), 1.83 (s, 3H), 1.77 (q, J = 6.2 Hz, 2H). ^{13}C NMR (101 MHz, MeOD) δ 172.0, 157.6, 138.5, 128.1, 127.9,

127.6, 127.6, 127.4, 127.1, 103.0, 97.4, 84.5, 78.7, 75.5, 74.6, 74.0, 72.2, 69.7, 66.7, 66.0, 60.9, 52.1, 48.3, 48.0, 47.8, 47.6, 47.4, 47.2, 47.0, 37.3, 29.4, 21.5. HR-ESI-MS *(m/z)*: $[M]^-$ calcd for $C_{39}H_{47}N_2O_{17}S^-$, 847.2601; found, 847.2598.

%

N-benzyloxycarbonyl-3-aminopropyl-*O*-[(methyl(2-azido-6-*O*-sulfonato-4-*O*-levulinoyl-3-*O*-(2-naphthylmethyl)-2-deoxy-*α*-D-glucopyranosyl))]-(1→4)-*O*-3-*O*-benzyl-1-β-D-glucopyranosiduronate (37)

The compound **33** was dissolved to (1 mmol, 1 eq) in DMF (2 mL) was added $SO_3.NEt_3$ complex (10 mmol per -OH, 10 eq) under N_2 atmosphere and stirred at 70 ^{o} C for 4 days. Upon completion of reaction, the reaction mixture was concentrated under reduced pressure and it was concentrated and purified using silica column chromatography (MeOH/DCM 1.3/10 v/v) to obtain product **37** in 78% yield. ^{1}H NMR (400 MHz, Chloroform-d) δ 7.99 (dd, J = 8.3, 1.4 Hz, 2H), 7.83 – 7.72 (m, 4H), 7.54 (t, J = 7.5 Hz, 1H), 7.46 – 7.36 (m, 5H), 7.29 (t, J = 7.7 Hz, 4H), 7.11 (d, J = 3.7 Hz, 4H), 5.38 (d, J = 3.3 Hz, 1H), 5.27 (t, J = 7.6 Hz, 1H), 5.19 – 5.12 (m, 1H), 5.05 (d, J = 27.3 Hz, 3H), 4.86 (d, J = 11.5 Hz, 1H), 4.81 – 4.71 (m, 2H), 4.67 – 4.57 (m, 2H), 4.24 (t, J = 8.7 Hz, 1H), 4.04 (dq, J = 14.5, 8.4, 7.5 Hz, 2H), 3.89 (dt, J = 20.2, 9.4 Hz, 3H), 3.75 (s, 3H), 3.64 (d, J = 9.8 Hz, 1H), 3.45 (dd, J = 10.8, 6.1 Hz, 1H), 3.37 – 3.27 (m, 1H), 3.07 (hept, J = 7.9 Hz, 2H), 2.53 (t, J = 11.4 Hz, 3H), 2.35 – 2.23 (m, 1H), 1.99 (s, 3H), 1.62 (dp, J = 13.9, 8.9, 8.1 Hz, 2H). ^{13}C NMR (101 MHz, $CDCl_3$) δ 209.0, 172.7, 169.2, 165.2, 156.6, 137.2, 136.9, 135.2, 133.5, 133.2, 133.0, 129.8, 129.4, 128.6, 128.5, 128.4, 128.1, 128.0, 127.9, 127.8, 127.7, 126.8, 126.1, 126.0, 100.8, 97.5, 82.0, 77.4, 77.3, 77.0, 76.7, 74.5, 73.4, 67.7, 66.4, 62.5, 53.5, 38.0, 37.5, 29.7, 29.4, 27.9. HR-ESI-MS *(m/z)*: $[M]^-$ calcd for $C_{54}H_{57}N_4O_{19}S^-$, 1097.3343; found, 1097.3329.

N-benzyloxycarbonyl-3-aminopropyl-*O*-[(methyl(2-azido-6-*tert*-butyldiphenylsilyl-4-*O*-levulinoyl-3-*O*-sulfonato-2-deoxy-*α*-D-glucopyranosyl))]-(1→4)-*O*-3-*O*-benzyl-1-β-D-glucopyranosiduronate (38)

A solution compound **34** containing (1 g, 1 eq) of starting material in 6 mL of CH_2Cl_2 and 340 µL of H_2O was prepared, to which DDQ (5 mmol) was gradually added over a 20-minute period. After 1 hour, the reaction mixture was quenched with $NaHCO_3$, extracted with CH_2Cl_2, and washed with brine. The organic layer obtained was dried over Na_2SO_4, filtered, concentrated,

and subjected to purification through silica gel column chromatography. Subsequently, the compound was dissolved in 2 mL of DMF, and $SO_3.NEt_3$ complex (10 mmol per -OH) was added under a N_2 atmosphere. The mixture was stirred at 70 °C for 3 days. After the reaction completion, the mixture was concentrated under reduced pressure, and the resulting product was purified using silica column chromatography (MeOH/DCM 1/10 v/v) to obtain product **38** yielding 69 %. ^{1}H NMR (400 MHz, Chloroform-d) δ 8.00 (d, J = 7.5 Hz, 2H), 7.67 – 7.58 (m, 4H), 7.52 (t, J = 7.4 Hz, 1H), 7.42 – 7.25 (m, 14H), 7.17 – 7.06 (m, 5H), 5.57 (d, J = 3.3 Hz, 1H), 5.37 – 5.29 (m, 1H), 5.22 (t, J = 9.6 Hz, 1H), 5.06 (d, J = 6.1 Hz, 1H), 5.02 (s, 2H), 4.80 (t, J = 9.8 Hz, 1H), 4.70 (q, J = 10.7 Hz, 2H), 4.61 (d, J = 7.1 Hz, 1H), 4.25 (t, J = 9.0 Hz, 1H), 4.13 – 3.99 (m, 2H), 3.85 (dt, J = 9.3, 5.8 Hz, 1H), 3.75 – 3.64 (m, 2H), 3.63 (d, J = 4.4 Hz, 1H), 3.60 (s, 3H), 3.49 (dq, J = 12.7, 6.3, 5.8 Hz, 1H), 3.26 (d, J = 10.4 Hz, 1H), 3.12 (ddt, J = 25.4, 12.5, 6.7 Hz, 2H), 2.88 – 2.68 (m, 4H), 2.58 (dt, J = 17.9, 5.9 Hz, 1H), 2.35 (dt, J = 18.6, 5.3 Hz, 1H), 2.15 (s, 3H), 1.66 (dt, J = 12.1, 7.0 Hz, 2H), 1.01 (s, 9H). ^{13}C NMR (101 MHz, $CDCl_3$) δ 209.6, 172.8, 169.0, 165.2, 156.5, 137.2, 136.8, 135.7, 135.6, 133.5, 133.3, 132.9, 129.8, 129.3, 128.6, 128.5, 128.4, 128.0, 127.9, 127.8, 127.8, 127.7, 100.8, 97.3, 82.2, 77.4, 77.3, 77.1, 76.7, 76.3, 74.2, 73.5, 73.2, 70.7, 68.8, 67.3, 66.4, 61.8, 61.6, 52.8, 37.9, 37.6, 30.3, 30.1, 29.7, 29.4, 29.4, 28.1, 26.8, 19.3. HR-ESI-MS *(m/z)*: $[M]^-$ calcd for $C_{59}H_{67}N_4O_{19}SiS^-$, 1195.3895; found, 1195.3881.

N-benzyloxycarbonyl-3-aminopropyl-*O*-[(2-sulfonatomido-6-*O*-sulfonato-3-*O*-(2-naphthylmethyl)-2-deoxy-*α*-D-glucopyranosyl)]-(1→4)-*O*-1-β-D-glucopyranosiduronate (39)

A solution of compound **37** (0.2g, 1 eq) in THF:H_2O (1:1, 5 mL) LiOH.H_2O (20 eq) was added and kept it on stirring for room temperature. After 12 h reaction was quenched using IR H^+ resin and reaction fixture was evaporated and purified using reverse phase C-18 column chromatography using (MeOH/H_2O 1/9 v/v). A solution of compound 53a in THF (3 mL) 1M PMe_3.THF (8 eq) and 0.1M aqueous NaOH (10eq) was added and kept it on stirring for room temperature. After 12 h reaction was quenched using glacial acetic acid and reaction mixture solvent was evaporated and purified using reverse phase C-18 column chromatography using (MeOH/H_2O 2/3 v/v), followed by passing through DOWEX 50WX8 Na^+ resin column. Then, a

solution of compound 53b in MeOH (3 mL) 0.1M NaOH (4 eq), triethylamine (8 eq), 10 eq of SO_3.Py was added at an interval of 1 h for 6 times by maintain 9-10 pH of reaction mixture and kept it on stirring for room temperature. The progress of reaction was monitored using TLC plate (ethyl acetate/pyridine/water/acetic acid 8/5/3/1 v/v/v/v). After completion, reaction mixture solvent was evaporated and purified using reverse phase C-18 column chromatography using ($MeOH/H_2O$ 2/3 v/v), followed by passing through DOWEX 50WX8 Na^+ resin column to obtain compound **39** in 61 % yield. ^{1}H NMR (400 MHz, Deuterium Oxide) δ 8.05 – 7.92 (m, 4H), 7.71 (dd, J = 8.5, 1.5 Hz, 1H), 7.56 (qd, J = 6.9, 3.4 Hz, 2H), 7.50 – 7.35 (m, 10H), 5.73 (d, J = 3.4 Hz, 1H), 5.10 (d, J = 11.3 Hz, 3H), 4.98 (d, J = 11.1 Hz, 1H), 4.92 – 4.84 (m, 3H), 4.46 (d, J = 8.1 Hz, 1H), 4.33 (dd, J = 11.0, 3.0 Hz, 1H), 4.20 (dd, J = 10.9, 1.8 Hz, 1H), 3.95 (dd, J = 10.3, 7.2 Hz, 2H), 3.89 – 3.80 (m, 3H), 3.73 – 3.63 (m, 3H), 3.53 (t, J = 8.4 Hz, 1H), 3.42 (dt, J = 6.3, 3.5 Hz, 1H), 3.25 (q, J = 6.3 Hz, 2H), 1.81 (p, J = 6.4 Hz, 2H). ^{13}C NMR (101 MHz, D_2O) δ 175.0, 137.5, 135.7, 133.0, 132.8, 128.8, 128.8, 128.7, 128.4, 128.2, 128.1, 127.7, 127.6, 127.0, 126.4, 102.2, 96.3, 83.7, 79.3, 76.9, 74.8, 73.5, 73.1, 70.3, 68.9, 67.8, 66.9, 66.5, 57.4, 37.4, 28.8. HR-ESI-MS *(m/z)*: $[M]^{2-}$ calcd for $C_{41}H_{46}N_2O_{19}S_2^{2-}$, 467.1073; found, 467.1068.

N-benzyloxycarbonyl-3-aminopropyl-*O*-[(2-sulfonatoimido-3-*O*-sulfonato-2-deoxy-*α*-D-glucopyranosyl)]-(1→4)-*O*-3-*O*-benzyl-1-β-D-glucopyranosiduronate (40)

A solution of compound **38** (0.15g, 1eq) in THF:H_2O (1:1, 5 mL) LiOH.H_2O (20 eq) was added and kept it on stirring for room temperature. After 12 h reaction was quenched using IR H^+ resin and reaction fixture was evaporated and purified using reverse phase C-18 column chromatography using ($MeOH/H_2O$ 1/9 v/v). A solution of compound 54a in THF (3 mL) 1M PMe_3.THF (8 eq) and 0.1M aqueous NaOH (10eq) was added and kept it on stirring for room temperature. After 12 h reaction was quenched using glacial acetic acid and reaction mixture solvent was evaporated and purified using reverse phase C-18 column chromatography using ($MeOH/H_2O$ 2/3 v/v), followed by passing through DOWEX 50WX8 Na^+ resin column. Then, a solution of compound 54b in MeOH (3 mL) 0.1M NaOH (4 eq), triethylamine (8 eq), 10 eq of SO_3.Py was added at an interval of 1 h for 6 times by maintain 9-10 pH of reaction mixture and kept it on stirring for room temperature. The progress of reaction was monitored using TLC plate (ethyl acetate/pyridine/water/acetic acid 8/5/3/1 v/v/v/v). After completion, reaction mixture solvent was evaporated and purified using reverse phase C-18 column chromatography using

(MeOH/H_2O 2/3 v/v), followed by passing through DOWEX 50WX8 Na^+ resin column to obtain compound **40** in 57 % yield. ^{1}H NMR (400 MHz, Deuterium Oxide) δ 7.55 – 7.50 (m, 2H), 7.49 – 7.35 (m, 8H), 5.74 (d, J = 3.3 Hz, 1H), 5.12 (s, 2H), 5.01 (d, J = 10.2 Hz, 1H), 4.90 (d, J = 10.3 Hz, 1H), 4.49 (d, J = 7.9 Hz, 1H), 4.40 (dd, J = 10.6, 8.2 Hz, 1H), 3.93 (s, 3H), 3.85 – 3.76 (m, 2H), 3.76 (d, J = 0.9 Hz, 1H), 3.72 – 3.61 (m, 3H), 3.48 (t, J = 8.2 Hz, 1H), 3.40 (dd, J = 10.6, 3.3 Hz, 1H), 3.25 – 3.24 (m, 2H), 1.81 (p, J = 6.2 Hz, 2H). ^{13}C NMR (151 MHz, D_2O) δ 174.4, 158.4, 137.4, 136.6, 129.0, 128.8, 128.6, 128.3, 128.3, 127.6, 102.1, 96.4, 83.7, 78.4, 74.3, 73.7, 72.2, 68.7, 67.8, 66.8, 60.3, 56.6, 55.4, 37.4, 28.7, 27.5, 19.1. HR-ESI-MS *(m/z)*: $[M]^{2-}$ calcd for $C_{30}H_{38}N_2O_{19}S_2^{2-}$, 397.0760; found, 397.0751.

3-aminopropyl-*O*-[(2-acetamido-6-*O*-sulfonato-*α*-D-glucopyranosyl)]-(1→4)-*O*-β-D-glucopyranosiduronate (G-2)

Compound **25** was dissolved in water, and $Pd(OH)_2$ was added, followed by stirring under an H_2 atmosphere for 48 hours. The mixture was then filtered, concentrated, and eluted through a Bond Elute C-18 column using H_2O as the eluent. The combined water fractions were pooled and subjected to lyophilization, resulting in the fully deprotected GlA-NAc-disaccharide **50**. ^{1}H NMR (600 MHz, Deuterium Oxide) δ 5.43 (d, *J* = 3.8 Hz, 1H), 4.48 (dd, *J* = 8.0, 1.3 Hz, 1H), 4.37 – 4.30 (m, 1H), 4.18 (dt, *J* = 11.1, 1.8 Hz, 1H), 4.01 – 3.95 (m, 1H), 3.94 – 3.82 (m, 3H), 3.81 – 3.66 (m, 4H), 3.58 (dd, *J* = 10.4, 9.1, 1.3 Hz, 1H), 3.37 – 3.32 (m, 1H), 3.16 (q, *J* = 6.7 Hz, 2H), 2.05 (s, 3H), 1.99 (p, *J* = 6.4 Hz, 2H). ^{13}C NMR (151 MHz, D_2O) δ 175.0, 174.3, 102.2, 96.8, 76.6, 76.5, 75.5, 73.4, 70.6, 70.0, 69.0, 67.9, 66.3, 53.5, 37.6, 26.7, 21.9. HR-ESI-MS *(m/z)*: $[M]^-$ calcd for $C_{17}H_{29}N_2O_{15}S^-$, 533.1294; found, 533.1281.

3-aminopropyl-*O*-[(2-acetamido-*α*-D-glucopyranosyl)]-(1→4)-*O*-β-D-glucopyranosiduronate (G-1)

Compound **26** was dissolved in water, and $Pd(OH)_2$ was added, followed by stirring under an H_2 atmosphere for 48 hours. The mixture was then filtered, concentrated, and eluted through a Bond Elute C-18 column using H_2O as the eluent. The combined water fractions were pooled and subjected to lyophilization, resulting in the fully deprotected GlA-NAc-disaccharide **51**. ^{1}H NMR (400 MHz, Deuterium Oxide) δ 5.32 (d, J = 3.7 Hz, 1H), 4.38 (d, J = 8.0 Hz, 1H), 3.93 – 3.84 (m, 1H), 3.82 – 3.74 (m, 2H), 3.73 – 3.67 (m, 4H), 3.66 – 3.59 (m, 3H), 3.39 (dd, J = 10.1,

9.1 Hz, 1H), 3.28 – 3.19 (m, 1H), 2.01 – 1.84 (m, 5H). ^{13}C NMR (101 MHz, D_2O) δ 175.2, 174.4, 100.4, 94.4, 73.1, 71.9, 71.1, 69.6, 68.7, 68.4, 67.3, 66.4, 60.1, 53.5, 38.2, 26.4, 21.9. HR-ESI-MS *(m/z)*: $[M]^-$ calcd for $C_{17}H_{30}N_2O_{12}$, 454.1799; found, 454.1793.

3-aminopropyl-*O*-[(2-acetamido-3-*O*-sulfonato-*α*-D-glucopyranosyl)]-(1→4)-*O*-β-D-glucopyranosiduronate (G-3)

Compound **27** was dissolved in water, and $Pd(OH)_2$ was added, followed by stirring under an H2 atmosphere for 48 hours. The mixture was then filtered, concentrated, and eluted through a Bond Elute C-18 column using H2O as the eluent. The combined water fractions were pooled and subjected to lyophilization, resulting in the fully deprotected GlA-NAc-disaccharide **52**. ^{1}H NMR (400 MHz, Deuterium Oxide) δ 5.36 (d, J = 3.6 Hz, 1H), 4.52 – 4.36 (m, 2H), 4.04 (dd, J = 10.6, 3.6 Hz, 1H), 3.94 (dt, J = 11.2, 5.8 Hz, 1H), 3.88 (d, J = 8.4 Hz, 1H), 3.84 – 3.77 (m, 2H), 3.74 (dd, J = 8.0, 5.6 Hz, 3H), 3.64 (t, J = 9.3 Hz, 1H), 3.43 – 3.25 (m, 2H), 3.15 (t, J = 7.2 Hz, 2H), 2.05 – 1.91 (m, 5H). ^{13}C NMR (151 MHz, D_2O) δ 174.8, 174.3, 102.2, 102.2, 97.5, 79.6, 76.9, 76.8, 76.7, 76.2, 76.2, 73.3, 71.8, 68.2, 67.9, 67.7, 59.9, 52.3, 50.8, 42.5, 37.5, 26.7, 26.0, 22.1, 18.1, 18.1. HR-ESI-MS *(m/z)*: $[M]^-$ calcd for $C_{17}H_{29}N_2O_{15}S^-$, 533.1294; found, 533.1289.

3-aminopropyl-*O*-[(2-sulfonatomido-6-*O*-sulfonato-*α*-D-glucopyranosyl)]-(1→4)-*O*-1-β-D-glucopyranosiduronate (G-4)

A solution of compound **42** in H_2O and $Pd(OH)_2$ was added and stirred under H_2 atm. After 48 h, the mixture was filtered, concentrated and eluted through Bond Elute C-18 column using H_2O as eluent. The combined H_2O fraction was pooled and lyophilized to yield fully deprotected NS-disaccharide **53**. ^{1}H NMR (600 MHz, Deuterium Oxide) δ 5.61 – 5.54 (m, 1H), 4.45 (dd, J = 7.9, 2.1 Hz, 1H), 4.25 (d, J = 11.0 Hz, 1H), 4.09 (d, J = 11.0 Hz, 1H), 3.92 (dt, J = 9.3, 5.6 Hz, 1H), 3.76 (tt, J = 16.9, 9.6 Hz, 5H), 3.55 – 3.46 (m, 2H), 3.30 (t, J = 7.9 Hz, 1H), 3.22 – 3.16 (m, 1H), 3.08 (s, 2H), 1.94 – 1.88 (m, 2H). ^{13}C NMR (151 MHz, D_2O) δ 174.3, 102.2, 97.4, 76.2, 76.2, 76.1, 75.9, 72.6, 71.0, 69.9, 69.0, 68.0, 66.4, 57.8, 37.6, 26.7.

3-aminopropyl-*O*-[(2-sulfonatomido-3-*O*-sulfonato-*α*-D-glucopyranosyl)]-(1→4)-*O*-1-β-D-glucopyranosiduronate (G-5)

A solution of compound **43** in H_2O and $Pd(OH)_2$ was added and stirred under H_2 atm. After 48 h, the mixture was filtered, concentrated and eluted through Bond Elute C-18 column using H_2O as eluent. The combined H_2O fraction was pooled and lyophilized to yield fully deprotected NS-disaccharide **54**. ^{1}H NMR (400 MHz, Deuterium Oxide) δ 5.59 (d, J = 3.5 Hz, 1H), 4.53 (d, J = 8.0 Hz, 1H), 4.36 (dd, J = 10.5, 9.0 Hz, 1H), 4.01 (dt, J = 11.5, 5.8 Hz, 1H), 3.92 – 3.75 (m, 7H), 3.68 (t, J = 9.2 Hz, 1H), 3.43 – 3.34 (m, 2H), 3.17 (p, J = 7.1 Hz, 2H), 2.00 (p, J = 6.4 Hz, 2H). ^{13}C NMR (151 MHz, D_2O) δ 174.9, 102.2, 98.2, 78.6, 78.0, 76.6, 76.1, 72.5, 71.7, 68.3, 68.0, 59.9, 56.8, 37.6, 26.6.

1. Synthesis of Amino Acid Building Block

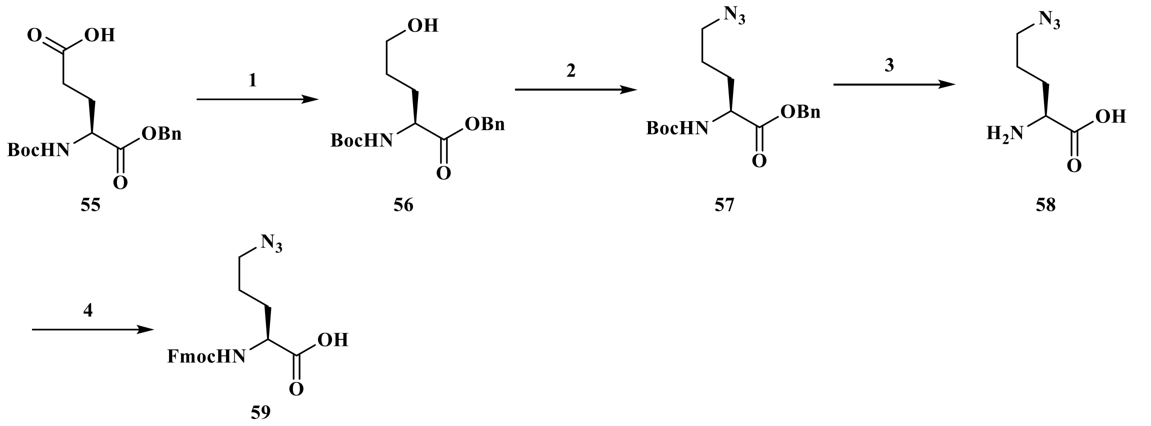

Reagent and Yield: (**1**) (**a**) NHS, DCC (**b**) $NaBH_4$, 70 %, (**2**) (**a**) MsCl, NEt_3, 90 %, (**b**) NaN_3, 81 %, (**3**) (**a**) $LiOH.H_2O$, 91 %, (**b**) 40 % TFA in DCM, 94 %, (**4**) Fmoc-Cl, Na_2CO_3, 62 %.

Benzyl (S)-2-((tert-butoxycarbonyl)amino)-5-hydroxypentanoate (56)

The commercially available Boc-NH-Glu (COOH)-O-tBu (4.54 g, 15 mmol) was dissolved in 30 mL of dry THF. The resulting mixture was cooled to 0 °C using a combination of salt and ice, and 2.06 mL (15 mmol) of triethylamine was added. After a 5-minute interval, N-hydroxysuccinamide (2 equivalents) and N,N′-Dicyclohexylcarbodiimide (5 equivalents) were introduced and stirred for 12 hours. Subsequently, $NaBH_4$ (3 equivalents) was added to the reaction mixture and stirred for an additional 30 minutes. The progress of the reaction was monitored using TLC. Upon completion of the reaction (approximately 30 minutes), the THF solvent was evaporated under reduced pressure, and any excess $NaBH_4$ was neutralized using a 10% HCl solution. The resulting mixture was then subjected to extraction with ethyl acetate (3 × 50 mL). The organic layer was washed with 10% HCl solution (3 × 50 mL), 10% Na2CO3 (3 ×

50 mL), brine solution (3 × 50 mL), and finally dried over anhydrous Na2SO4. The compound was purified using silica gel chromatography with ethyl acetate/hexane (1/3 v/v) to obtain compound **25**. ^{1}H NMR (400 MHz, Chloroform-d) δ 7.39 – 7.30 (m, 5H), 5.12 (s, 2H), 4.84 – 4.78 (m, 1H), 3.63 (t, J = 6.3 Hz, 2H), 3.59 – 3.50 (m, 1H), 2.52 – 2.42 (m, 2H), 1.95 – 1.87 (m, 1H), 1.83 – 1.75 (m, 1H), 1.43 (s, 9H). ^{13}C NMR (101 MHz, CHLOROFORM-D) δ 173.6, 156.3, 135.8, 128.7, 128.4, 128.4, 79.8, 77.4, 77.1, 76.8, 66.6, 65.3, 52.3, 34.0, 31.0, 28.4, 26.3.

Benzyl (S)-5-azido-2-((tert-butoxycarbonyl)amino)pentanoate (57)

A solution of compound **56** comprising 1 equivalent of compound in dry dichloromethane (DCM) underwent treatment with 2 equivalents of triethylamine at 0 °C. After 5 minutes of stirring, 3 equivalents of methanesulfonyl chloride were introduced and the mixture was stirred for 45 minutes. Quenching of the reaction was achieved using 1N aqueous ammonium chloride (NH_4Cl), and the resultant mixture was subjected to thrice washing with NH_4Cl before undergoing drying over anhydrous sodium sulfate (Na_2SO_4). Subsequent purification involved silica gel chromatography utilizing an ethyl acetate/hexane solvent system (1:3 v/v). Post-purification, the compound was dissolved in dry dimethylformamide (DMF), and 2 equivalents of sodium azide (NaN_3) were incorporated, followed by stirring for 12 hours at 45 °C. To this resultant reaction mixture, 50 mL of ethyl acetate was added, followed by thrice washing with brine and final drying over Na_2SO_4. The compound underwent another purification step using silica gel chromatography with an ethyl acetate/hexane solvent system (2:3 v/v) to obtain **57**. ^{1}H NMR (400 MHz, Chloroform-*d*) δ 7.41 – 7.28 (m, 5H), 5.13 (s, 2H), 4.62 (d, *J* = 8.1 Hz, 1H), 3.82 – 3.69 (m, 1H), 3.41 (qd, *J* = 12.3, 4.3 Hz, 2H), 2.45 (t, *J* = 7.4 Hz, 2H), 1.95 – 1.72 (m, 2H), 1.43 (s, 9H). ^{13}C NMR (101 MHz, $CDCl_3$) δ 172.9, 155.3, 135.8, 128.6, 128.3, 128.3, 79.9, 77.4, 77.0, 76.7, 66.5, 54.9, 50.0, 30.8, 28.3, 27.4.

(S)-2-((((9H-fluoren-9-yl)methoxy)carbonyl)amino)-5-azidopentanoic acid (59)

Compound **57** (1 equivalent) was dissolved in a mixture of MeOH:THF:H2O (1:1:1). Subsequently, 5 equivalents of $LiOH.H_2O$ were added, and the mixture was stirred for 2 hours at room temperature. After evaporation, ethyl acetate was added, followed by three washes with 1N HCl. The resulting solution was dried over anhydrous Na_2SO_4. Next, 40% TFA in DCM was added to the reaction mixture, and it was stirred for 2 hours at room temperature. After

evaporation and vacuum drying, Compound **58** (1 equivalent) was dissolved in 20 mL of H_2O and cooled to 0 °C. To this solution, 10 mL of 10% Na_2CO_3 was added, followed by Fmoc-Cl (2 equivalents), and the reaction mixture was stirred for approximately 4 hours. The progress of the reaction was monitored by TLC. Upon completion, H_2O was evaporated under reduced pressure, and the resulting mixture was acidified with a 1N HCl solution. Extraction with ethyl acetate (3 × 50 mL) followed, and the combined organic layer was washed with a brine solution (3 × 50 mL). After drying over anhydrous Na_2SO_4, the organic layer was evaporated under reduced pressure. The compound was then purified using silica gel chromatography with ethyl acetate/hexane (2/3 v/v) to obtain compound **59**. ^{1}H NMR (400 MHz, Chloroform-*d*) δ 7.76 (d, *J* = 7.5 Hz, 2H), 7.57 (dd, *J* = 18.3, 7.0 Hz, 2H), 7.40 (t, *J* = 7.5 Hz, 2H), 7.31 (t, *J* = 7.5 Hz, 2H), 5.38 (d, *J* = 8.1 Hz, 1H), 4.43 (t, *J* = 6.2 Hz, 2H), 4.22 (t, *J* = 6.6 Hz, 1H), 3.41 – 3.09 (m, 2H), 1.96 (s, 1H), 1.84 – 1.35 (m, 4H). ^{13}C NMR (101 MHz, CHLOROFORM-D) δ 176.4, 156.2, 143.9, 143.7, 141.4, 127.9, 127.2, 127.2, 125.1, 125.1, 120.1, 120.1, 77.5, 77.1, 76.8, 67.2, 53.3, 50.9, 47.2, 29.8, 29.7, 24.9.

5. Synthesis of Peptide Backbone

A 12mer peptide was manually synthesized via solid-phase peptide synthesis on rink amide resin at a 0.3 mmol scale, employing standard Fmoc conditions. The coupling reagents consisted of a combination of HBTU and HOBt, with DIPEA serving as the activator base. NMP was utilized as the coupling solvent, and 20% piperidine in DMF was applied for Fmoc group deprotection. To remove the peptides from the solid support, the resin underwent stirring for approximately 2 hours in a 10 mL cocktail containing trifluoroacetic acid (TFA)/triisopropylsilane/water/phenol (90:5:2.5:2.5). After filtration of the resin, the crude peptides in the filtrate were evaporated under reduced pressure, and the residue was triturated with 20 mL of cold diethyl ether. The resulting precipitate was separated through centrifugation. The crude peptides were subsequently dissolved in 10 mL of MeOH and subjected to purification on a C-18 column using reversed-phase HPLC. A MeOH and water gradient system containing 0.1% TFA at a 0.5 mL flow rate was employed for peptide purification. Confirmation of the purified peptides was accomplished through IR, NMR, and HR-ESI-MS.

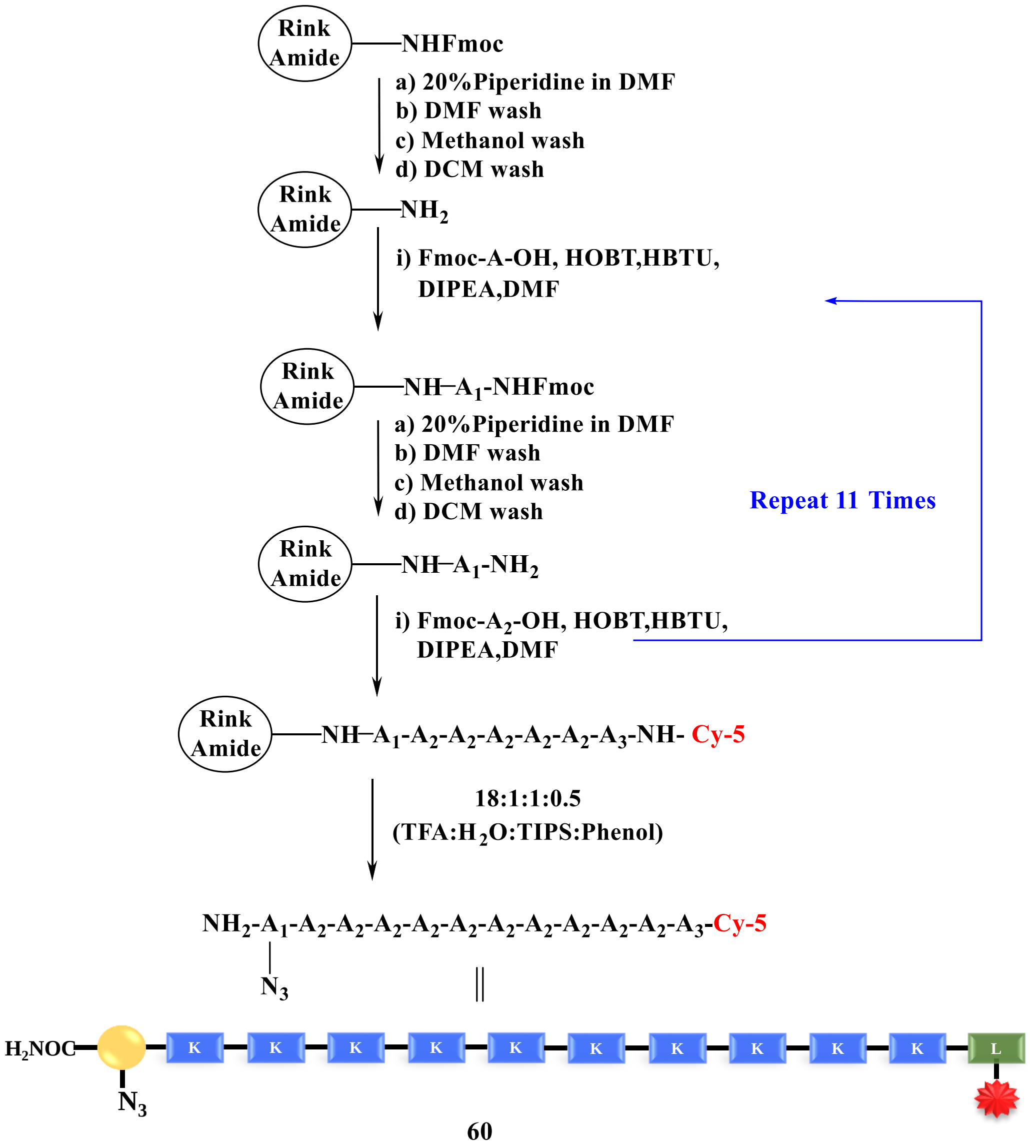

60

12mer Peptide Backbone

^{1}H NMR (600 MHz, Methanol-d_4) δ 8.13 (t, J = 11.3 Hz, 2H), 8.05 – 7.82 (m, 5H), 7.37 (dd, J = 7.6, 4.5 Hz, 2H), 7.30 (tt, J = 7.7, 1.9 Hz, 2H), 7.16 (dt, J = 14.6, 7.3 Hz, 4H), 6.55 – 6.46 (m, 1H), 6.16 (dd, J = 13.7, 4.0 Hz, 1H), 4.34 – 3.76 (m, 15H), 3.52 (s, 3H), 3.20 (dq, J = 4.4, 2.7, 2.2 Hz, 3H), 2.82 (h, J = 6.8 Hz, 23H), 2.23 (dt, J = 12.3, 6.1 Hz, 2H), 1.88 – 1.65 (m, 29H), 1.64 – 1.51 (m, 47H), 1.51 – 1.43 (m, 6H), 1.42 – 1.28 (m, 17H). ^{13}C NMR (151 MHz, MeOD) δ 176.4, 175.8, 175.7, 175.6, 175.2, 175.2, 175.1, 175.1, 175.0, 174.8, 174.6, 174.5, 174.1, 173.6, 173.4, 173.1, 161.3, 161.0, 158.0, 157.5, 157.2, 156.9, 154.2, 154.0, 142.8, 142.2, 141.2, 141.1, 128.6, 128.3, 125.2, 125.2, 124.9, 124.8, 122.0, 121.9, 119.6, 117.6, 117.5, 115.7, 115.7, 115.6,

114.3, 113.7, 111.8, 110.5, 110.5, 103.1, 102.8, 56.8, 56.7, 56.5, 56.3, 56.1, 55.4, 54.8, 54.7, 54.5, 53.8, 53.7, 53.4, 53.3, 53.1, 53.0, 52.8, 50.8, 50.7, 49.1, 49.1, 48.5, 48.1, 47.9, 47.8, 47.7, 47.5, 47.4, 47.2, 43.5, 39.2, 39.1, 38.9, 35.1, 30.2, 30.1, 29.9, 29.7, 29.5, 28.7, 27.5, 26.9, 26.7, 26.6, 26.4, 26.3, 25.4, 25.3, 25.1, 25.0, 23.1, 23.0, 22.9, 22.8, 22.7, 22.6, 22.3, 15.9. HR-ESI-MS Calcd for $C_{100}H_{173}N_{28}O_{13}^{+}$ $[M]^{+}$ is 1974.3731, found is 1974.3710.

6. Synthesis Protocol for Cholesterol and Azide Linker Conjugation

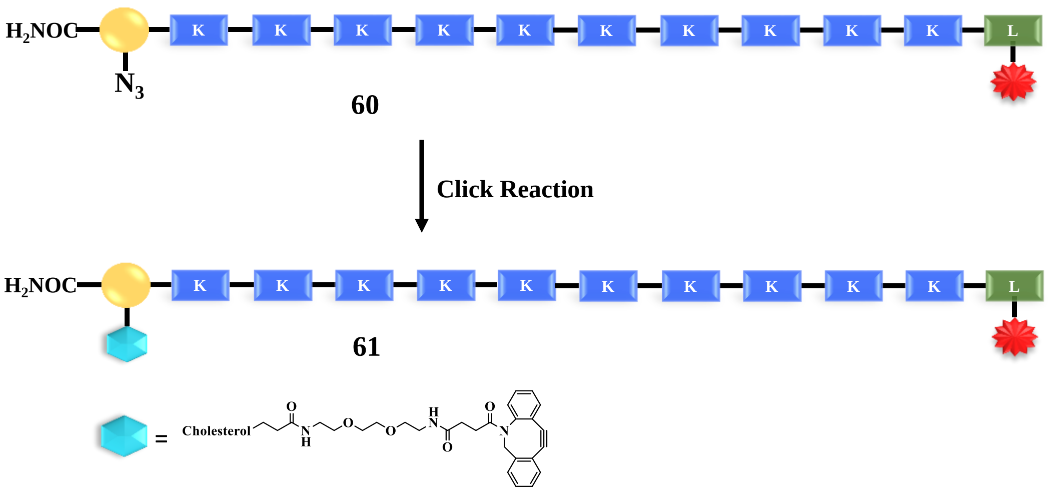

6a. Procedure for conjugation of Cholesterol-DBCO

A 12-mer peptide (1 equivalent) was dissolved in 1 mL of MeOH, while cholesterol-DBCO (1 equivalent) was dissolved in 1 mL of DCM. The subsequent step involved the gradual addition of the cholesterol-DBCO solution into the peptide solution, with continuous stirring for 12 hours at room temperature. The progress of the reaction was tracked through MALDI-TOF/TOF analysis. Upon completion of the reaction (approximately 12 hours), the solvent was evaporated under reduced pressure. The crude compound underwent purification via C-18 bond elute column chromatography in a MeOH/H_2O (6/10) solvent system, yielding the pure cholesterol-conjugated peptide compound. Confirmation was accomplished through IR, NMR, and HR-ESI-MS analyses.

12mer Peptide with Cholesterol

^{1}H NMR (600 MHz, Methanol-d_4) δ 8.31 – 8.19 (m, 2H), 7.94 (d, J = 77.2 Hz, 2H), 7.71 – 7.52 (m, 4H), 7.50 (dd, J = 7.6, 2.2 Hz, 3H), 7.45 – 7.36 (m, 5H), 7.33 – 7.25 (m, 6H), 6.63 (dd, J =

13.9, 11.1 Hz, 1H), 6.32 – 6.26 (m, 2H), 5.37 – 5.32 (m, 1H), 4.31 – 3.97 (m, 17H), 3.64 (s, 3H), 3.62 – 3.48 (m, 11H), 2.97 – 2.93 (m, 28H), 2.46 – 2.27 (m, 9H), 2.00 – 1.78 (m, 36H), 1.73 (d, *J* = 3.3 Hz, 17H), 1.62 – 1.67 (m, 28H), 1.63 – 1.36 (m, 48H), 1.29 (s, 3H), 1.21 – 1.01 (m, 10H), 1.00 (s, 3H), 0.94 (d, *J* = 6.5 Hz, 4H), 0.89 (d, *J* = 2.2 Hz, 4H), 0.88 (d, *J* = 2.2 Hz, 4H), 0.71 (s, 3H). ^{13}C NMR (151 MHz, MeOD) δ 174.2, 174.2, 174.2, 173.1, 172.9, 162.0, 161.8, 161.5, 161.3, 154.3, 154.0, 142.8, 142.2, 141.2, 141.1, 140.5, 131.1, 129.8, 129.8, 129.1, 128.4, 127.1, 126.8, 125.2, 125.0, 124.8, 122.1, 121.9, 121.4, 120.3, 119.7, 117.8, 115.9, 113.9, 110.5, 103.1, 102.7, 79.3, 69.9, 69.9, 69.8, 69.2, 69.2, 65.1, 63.7, 62.7, 56.8, 56.2, 50.3, 49.2, 49.1, 48.5, 48.1, 47.9, 47.8, 47.6, 47.5, 47.4, 47.2, 43.4, 42.1, 39.7, 39.3, 39.1, 39.0, 39.0, 38.8, 37.0, 36.6, 36.6, 36.0, 35.7, 35.1, 33.6, 30.4, 30.3, 30.2, 29.6, 29.4, 29.4, 29.3, 29.2, 29.1, 29.0, 28.8, 28.0, 27.9, 27.7, 26.9, 26.7, 26.6, 26.4, 26.3, 25.1, 24.6, 23.9, 23.5, 22.8, 22.7, 22.7, 22.6, 22.3, 21.8, 21.6, 20.8, 18.5, 17.9, 13.1, 10.9. HR-ESI-MS Calcd for $C_{155}H_{252}N_{31}O_{19}^{+}$ $[M]^{+}$ is 2851.9700, found is 2852.9751 (M+H).

6b. Procedure for Conjugation of 3-Azidopropanoate Linker Conjugation

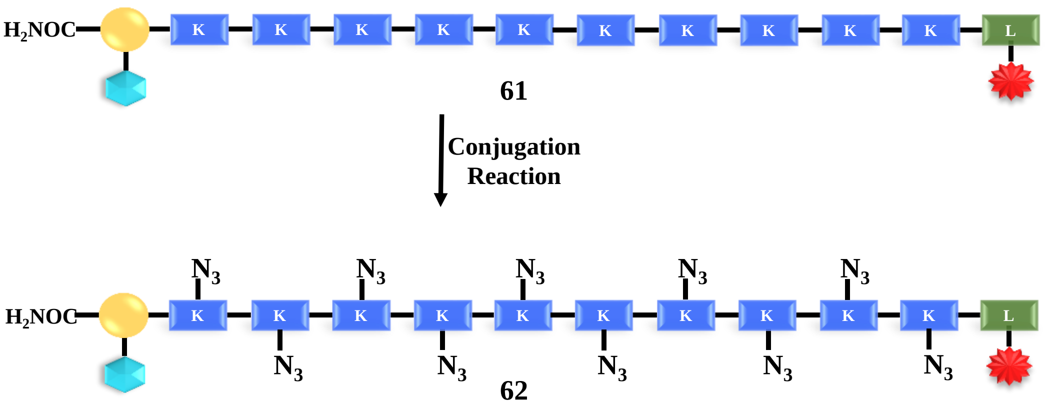

A 12-mer peptide, combined with cholesterol (1 equivalent), was dissolved in 1 mL of DMF. Subsequently, 40 µL of DIPEA was added to the solution, followed by the gradual addition of a solution containing 2,5-dioxopyrrolidin-1-yl 3-azidopropanoate (10 equivalents per NH_2) in 1 mL of DMF. The resulting peptide solution was stirred for 12 hours at room temperature. The progression of the reaction was monitored through MALDI-TOF/TOF analysis. After the reaction's completion (approximately 12 hours), the solvent was evaporated under reduced pressure. The crude compound underwent purification through LH – 20 size exclusion column

chromatography, utilizing MeOH as the solvent system. Additionally, it was passed through Amberlite® IR120, Na^+ form, to obtain the pure compound. Confirmation was done by IR, NMR, and MALDI-TOF/TOF analyses.

12mer Peptide with 3-Azidopropanoate Linker

^{1}H NMR (600 MHz, Methanol-d4) δ 7.71 (td, J = 14.7, 13.5, 8.4 Hz, 5H), 7.38 – 7.28 (m, 2H), 7.21 (d, J = 8.9 Hz, 1H), 7.14 (q, J = 7.4 Hz, 4H), 7.10 – 6.90 (m, 6H), 5.93 (dd, J = 21.7, 13.6 Hz, 1H), 5.05 (d, J = 5.0 Hz, 1H), 3.89 – 3.75 (m, 4H), 3.68 – 3.52 (m, 11H), 3.47 – 3.29 (m, 18H), 3.27 (dd, J = 11.4, 5.9 Hz, 28H), 2.99 – 2.79 (m, 24H), 2.62 – 2.47 (m, 7H), 2.21 – 2.12 (m, 23H), 1.69 – 1.48 (m, 25H), 1.45 (d, J = 3.6 Hz, 13H), 1.35 – 1.18 (m, 39H), 1.19 – 1.07 (m, 19H), 0.97 (s, 14H), 0.90 – 0.69 (m, 15H), 0.63 (d, J = 6.5 Hz, 4H), 0.57 (dd, J = 6.6, 2.6 Hz, 8H), 0.40 (s, 3H). ^{13}C NMR (151 MHz, MeOD) δ 175.8, 175.5, 175.4, 175.2, 174.5, 173.7, 173.2, 172.6, 171.0, 153.6, 153.4, 142.6, 142.0, 141.0, 140.8, 140.4, 131.2, 131.0, 129.4, 128.7, 127.9, 125.4, 125.1, 122.2, 122.1, 121.8, 110.8, 110.6, 103.1, 79.4, 77.7, 77.4, 77.2, 70.0, 69.8, 69.5, 65.2, 63.9, 63.0, 56.7, 56.1, 53.4, 51.5, 50.1, 49.3, 48.9, 48.7, 48.6, 48.4, 48.3, 48.1, 48.0, 47.4, 46.6, 44.0, 42.2, 39.7, 39.4, 39.3, 39.1, 38.9, 37.1, 36.9, 36.7, 36.1, 35.7, 35.3, 35.2, 34.0, 33.7, 32.2, 31.8, 31.8, 30.9, 29.6, 29.5, 29.5, 29.5, 29.3, 29.2, 29.1, 29.0, 28.9, 28.2, 28.1, 27.9, 27.6, 27.5, 27.3, 26.6, 25.4, 25.2, 24.8, 24.1, 23.7, 22.5, 22.5, 22.2, 21.0, 19.1, 18.4, 16.3, 13.7, 11.6. MALDI-TOF/TOF Calcd for $C_{185}H_{282}N_{61}O_{29}^+$ [M] is 3822.2461, found is 3822.2245.

7. General Procedure for Conjugation of Sugar Molecules

The sugar derivative was dissolved in 400 µL of dry DMF and stirred for 5 minutes. Next, NEt_3 (2 equivalents) was added, followed by dropwise addition of the solution of DBCO-NHS in 400 µL, while stirring at room temperature. After 12 hours, DMF was evaporated, and the crude product was purified using LH-20 size exclusion chromatography. Then, a compound with an azide linker (1 eq.) was dissolved in 500 µL of $CHCl_3$ and 500 µL of MeOH. Subsequently, a solution of sugar molecules (1.2 eq. per azide of peptide) in H_2O (1 mL) was added, followed by the addition of another 500 µL of MeOH. The resulting solution was stirred for 7 – 8 days at room temperature. The progress of the reaction was monitored by infrared spectroscopy (IR). After the completion of the reaction (approximately 7 - 8 days), the solvent was evaporated under reduced pressure. The crude product was then purified using a 10 kDa centrifuge filter,

followed by a C-18 column with reversed-phase high-performance liquid chromatography (HPLC). A gradient system of (95% MeOH+5% H_2O) and (95% water + 5% H_2O) at a flow rate of 1 mL/min was employed for compound purification. Confirmation of the product was achieved through IR, NMR, and HRMS-ESI. When the reaction got completed, we can observe disappearance of azide peak at 2210 nm^{-1} in IR spectrum.

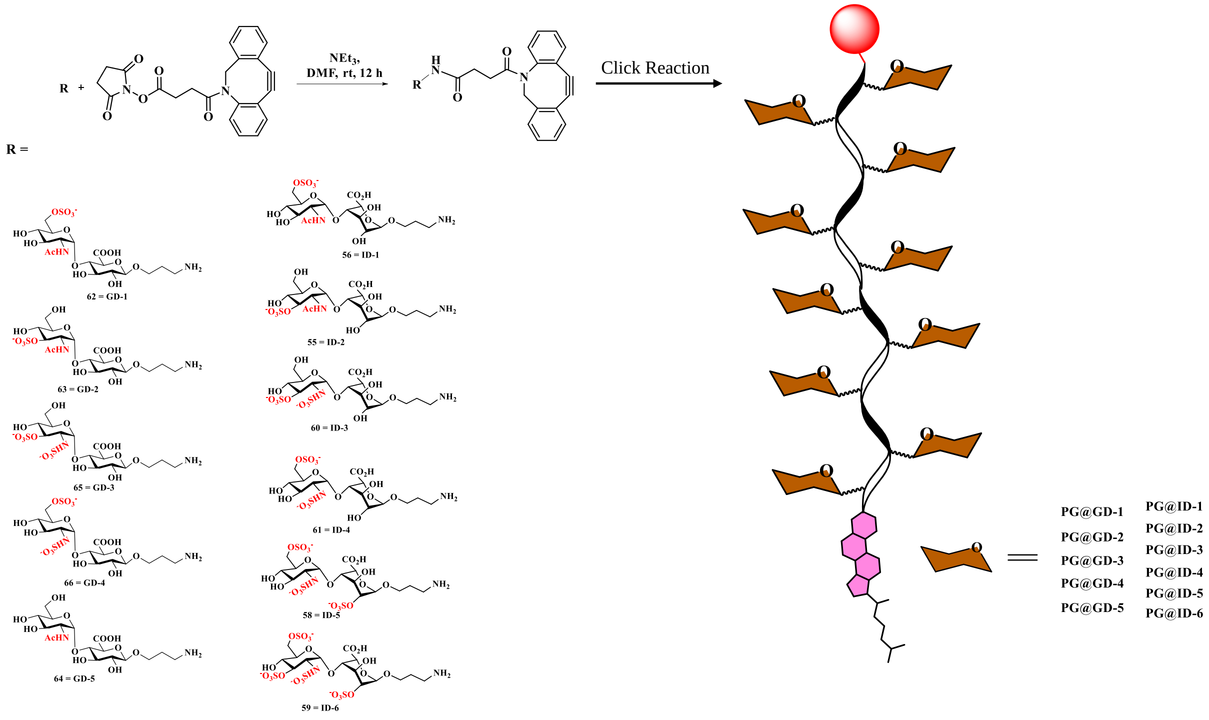

PG@ID-1

The compound was characterized using ^{1}H NMR, IR and HR-ESI-MS *(m/z)*: $[M]^{9-}$, calcd is 1336.6012; found, 1336.0099. The purity of compound was checked using reverse-phase high-performance liquid chromatography (HPLC) the elution time is ~ 19.5 min.

PG@ID-2

The compound was characterized using ^{1}H NMR, IR and HR-ESI-MS *(m/z)*: $[M]^{9-}$, calcd is 1336.6012; found 1336.5524. The purity of compound was checked using reverse-phase high-performance liquid chromatography (HPLC) the elution time is ~ 19.5 min.

PG@ID-3

The compound was characterized using ^{1}H NMR, IR and HR-ESI-MS *(m/z)*: $[M]^{19-}$ calcd is 652.3311; found, 652.2312. The purity of compound was checked using reverse-phase high-performance liquid chromatography (HPLC) the elution time is ~ 17.5 min.

PG@ID-4

The compound was characterized using ^{1}H NMR, IR and HR-ESI-MS *(m/z)*: $[M]^{19-}$ calcd is 652.3311; found, 652.2242. The purity of compound was checked using reverse-phase high-performance liquid chromatography (HPLC) the elution time is ~ 17.4 min.

PG@ID-5

The compound was characterized using ^{1}H NMR, IR and HR-ESI-MS *(m/z)*: $[M]^{29-}$ calcd is 454.1048; found, 454.0741. found, 1336.0099. The purity of compound was checked using reverse-phase high-performance liquid chromatography (HPLC) the elution time is ~ 16.1 min.

PG@ID-6

The compound was characterized using ^{1}H NMR, IR and HR-ESI-MS *(m/z)*: $[M]^{39-}$ calcd is 356.5128; found, $[M]^{39-}$ + Na + 5H is 384.1110. The purity of compound was checked using reverse-phase high-performance liquid chromatography (HPLC) the elution time is ~ 15 min.

PG@GD-1

The compound was characterized using ^{1}H NMR, IR and MALDI-TOF/TOF. The purity of compound was checked using reverse-phase high-performance liquid chromatography (HPLC) the elution time is ~ 22.5 min.

PG@GD-2

The compound was characterized using ^{1}H NMR, IR and HR-ESI-MS *(m/z)*: $[M]^{19-}$ calcd is 1336.1012; found, $[M]^{19-}$ + 6H is 1342.3002. The purity of compound was checked using reverse-phase high-performance liquid chromatography (HPLC) the elution time is ~ 19.5 min.

PG@GD-3

The compound was characterized using ^{1}H NMR, IR and HR-ESI-MS *(m/z)*: $[M]^{19-}$ calcd is 1336.1012; found, 1336.2484. The purity of compound was checked using reverse-phase high-performance liquid chromatography (HPLC) the elution time is ~ 19.5 min.

PG@GD-4

The compound was characterized using ^{1}H NMR, IR and HR-ESI-MS *(m/z)*: $[M]^{29-}$ calcd is 652.3311; found, 652.2471. The purity of compound was checked using reverse-phase high-performance liquid chromatography (HPLC) the elution time is ~ 17.3 min.

PG@GD-5

The compound was characterized using ^{1}H NMR, IR and HR-ESI-MS *(m/z)*: $[M]^{29-}$ calcd for 652.3311; found, 652.1973. The purity of compound was checked using reverse-phase high-performance liquid chromatography (HPLC) the elution time is ~ 17.3 min.

8. Physical Characterization

PG@ID-6 (NeoMProbe)

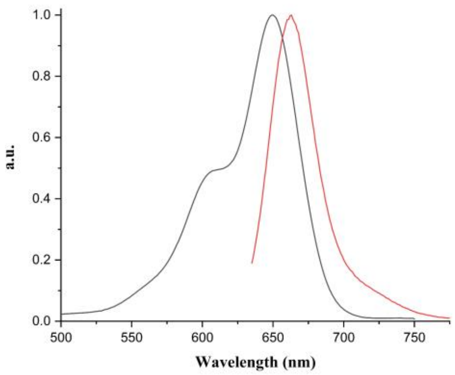

PG@GD-1

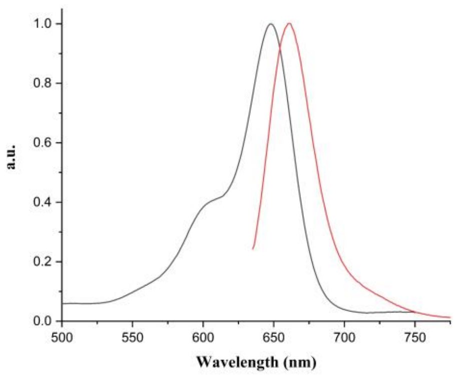

Standard compound used for calculation of quantum yield is Atto 655.

Table S1: Absorbance, emission and quantum yield of L-Iduronic acid-based molecules

S.No.	Compound	Absorbance (nm)	Emission (nm)	Quantum Yield
1.	PG@ID-1	648	660	22.8
2.	PG@ID-2	649	661	21.9
3.	PG@ID-3	649	662	22.3
4.	PG@ID-4	650	663	22.8
5.	PG@ID-5	648	662	22.2
6.	PG@ID-6	650	665	22.8

Table S2: Absorbance, emission and quantum yield of D-Glucuronic acid-based molecules

S.No.	Compound	Absorbance (nm)	Emission (nm)	Quantum Yield

1.	PG@GD-1	649	658	21.8
2.	PG@GD-2	648	660	22.0
3.	PG@GD-3	649	662	22.3
4.	PG@GD-4	650	664	22.1
5.	PG@GD-5	651	663	22.5

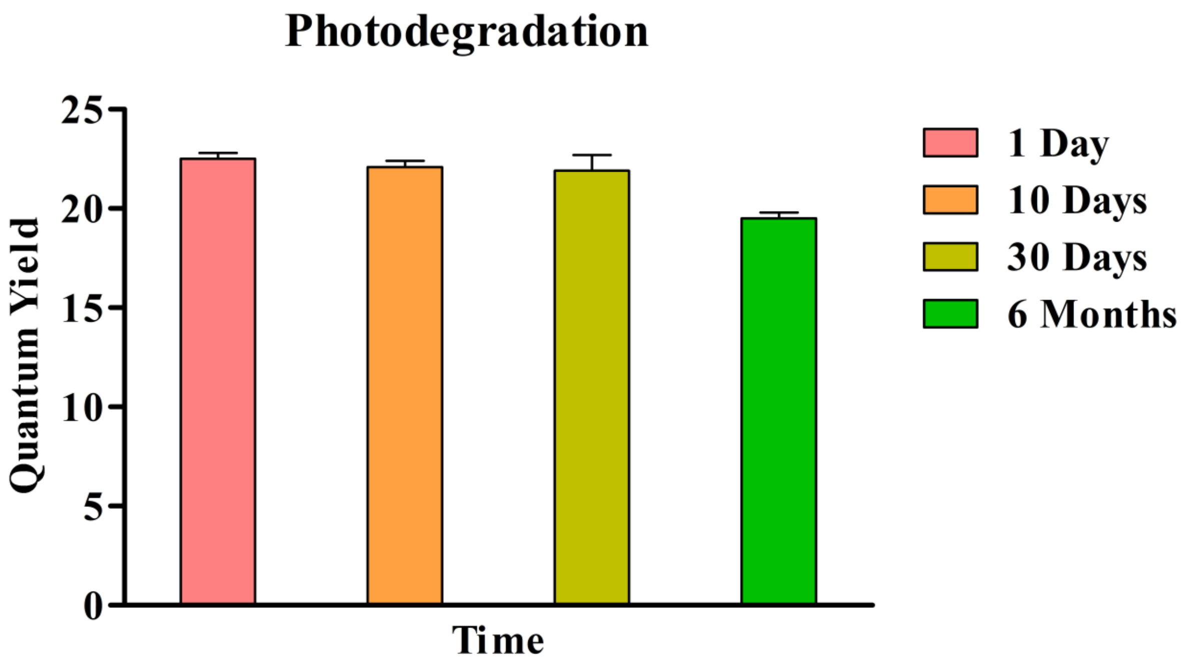

Figure S1. Quantum yield of the NeoMProbe at different time intervals.

9. Cell Lines

MDA-MB-468 and NIH3T3 cells were cultured at 37 °C in a 5% CO_2 atmosphere in DMEM media supplemented with 10% fetal bovine serum (FBS) and 0.1% streptomycin.

MTT Assay. MTT assay was used to check toxicity of the molecules. MDA-MB-468 and NIH-3T3 cells (5×10^3 cells per well) were plated in 96 well plate in DMEM medium and incubated overnight followed by treatment different concentration of neoPGs and further incubation for 48 h. Afterward 10 µl of MTT (5 mg/ml) reagent was added to each well and incubated plates further for 4h at 37 ºC. Purple precipitate formed was dissolved by adding 100 µl of DMSO and plate was read at 570 nm.

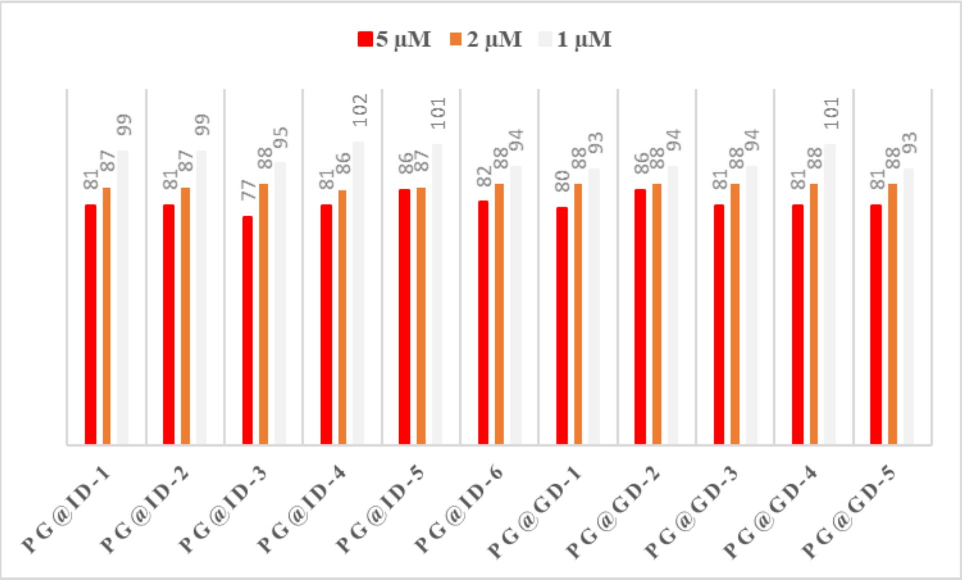

Fig S2a. MTT assay of NeoPGs in NIH-3T3 cells after 48 hours of incubation. The experiment represents the average of three independent experiments

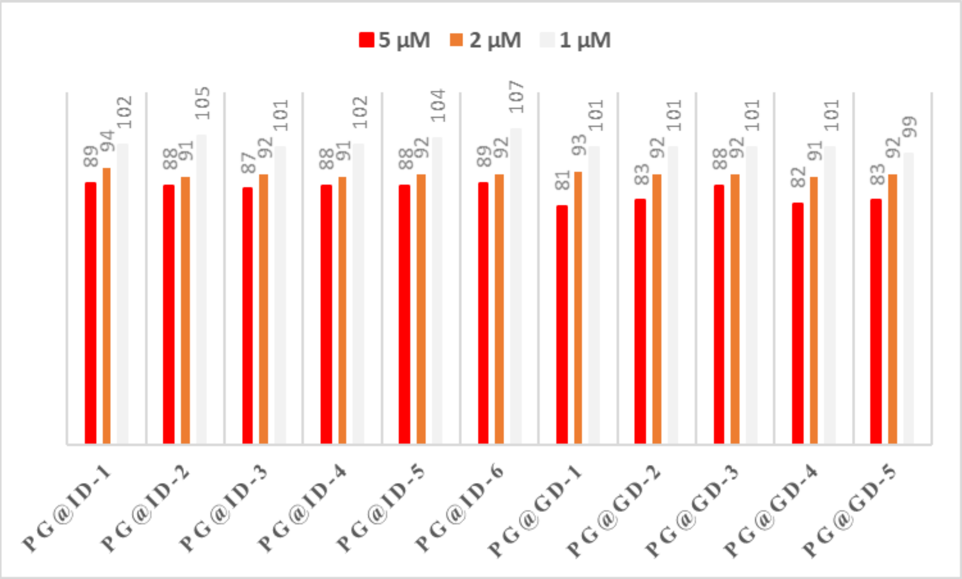

Fig S2b. MTT assay of NeoPGs in MDA-MB-468 cells after 48 hours of incubation. The experiment represents the average of three independent experiments

10. Confocal Imaging Study

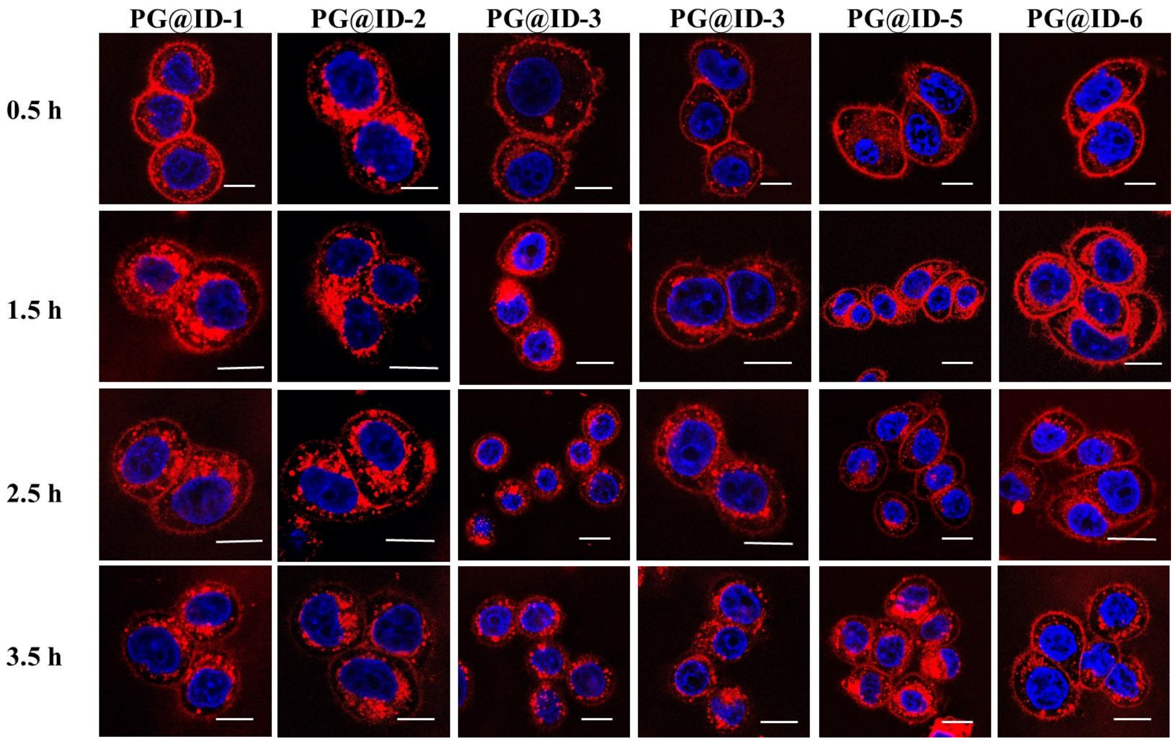

Fig. S3: Time dependent confocal images of MDA-MB-468 treated with 2 μM of PG@ID's.

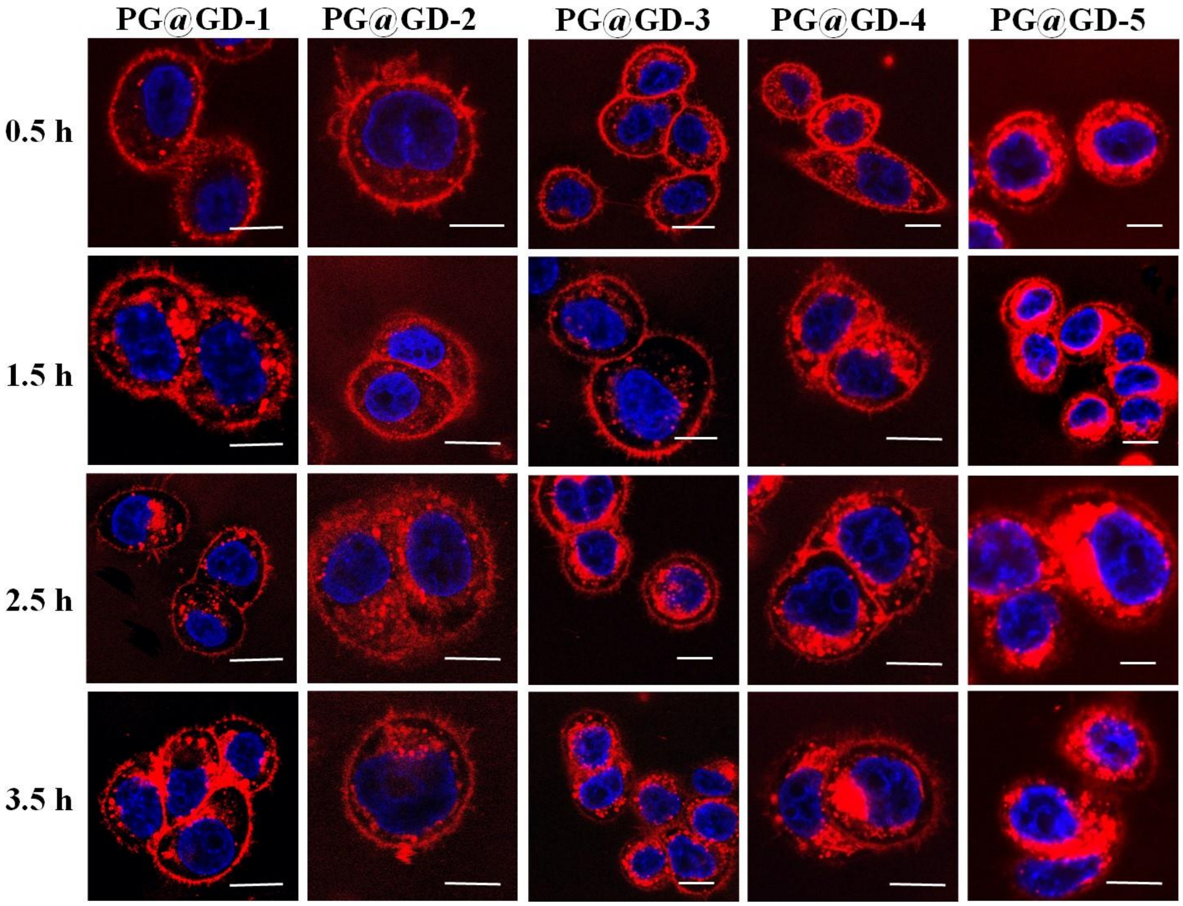

Fig. S4: Time dependent confocal images of MDA-MB-468 using 2 μM of PG@GD's library.

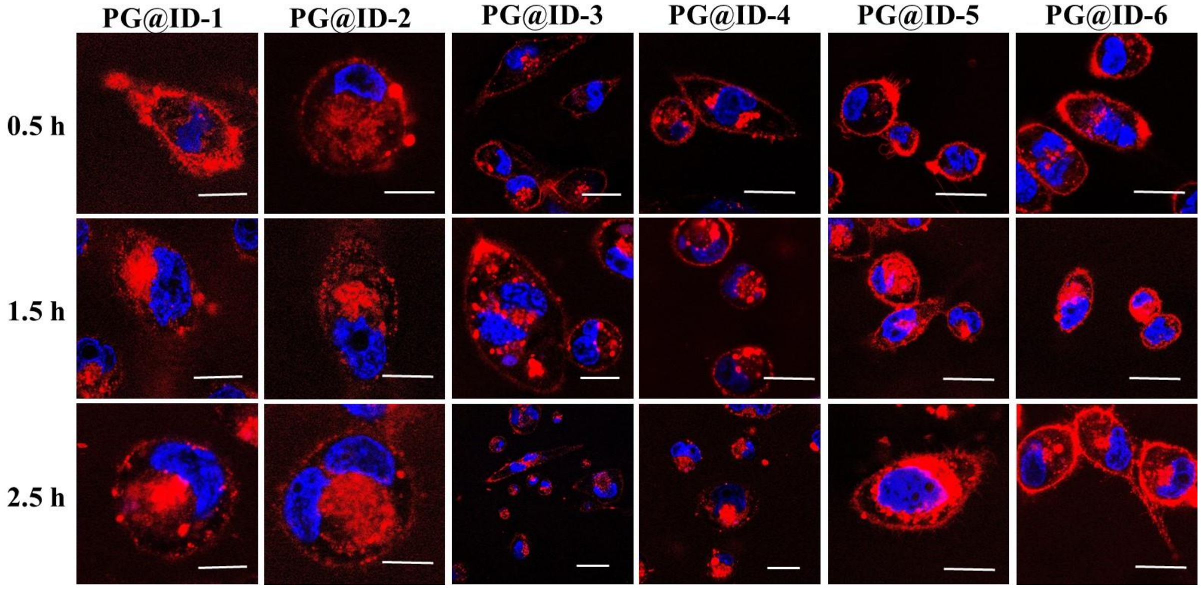

Fig. S5: Time dependent confocal images of NIH3T3 treated with 2 μM of PG@ID's library.

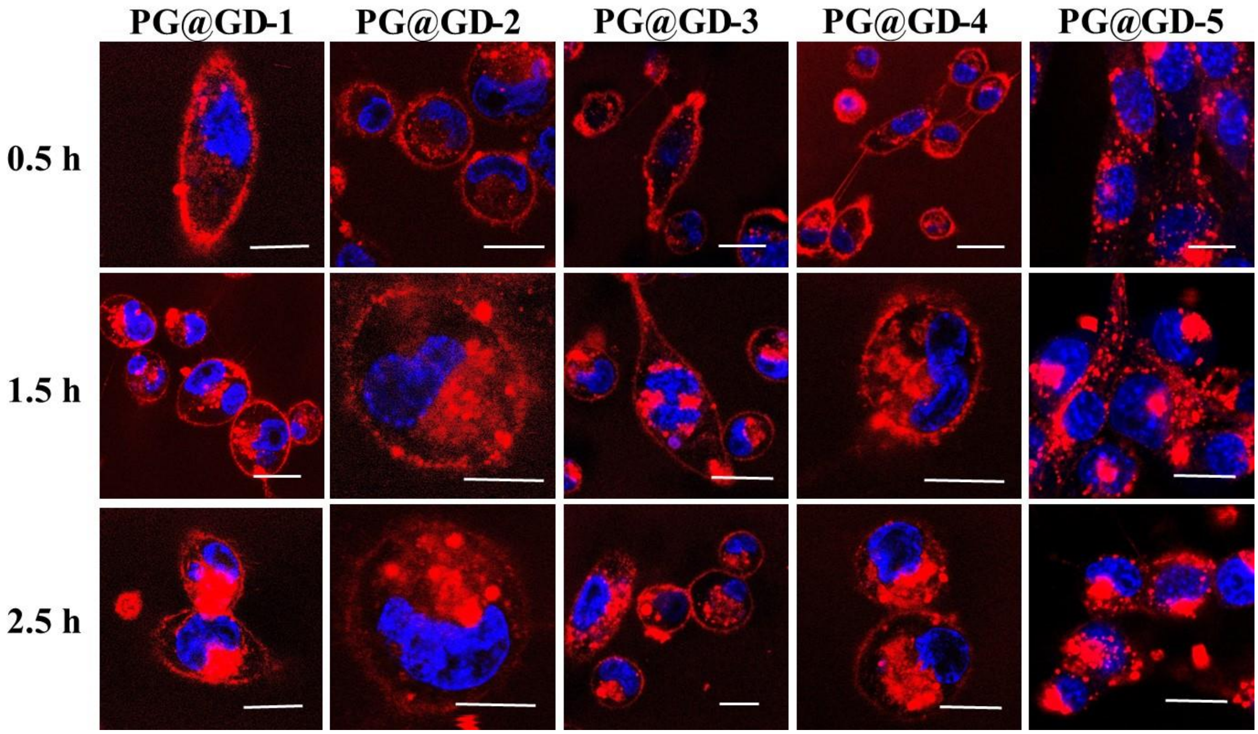

Fig. S6: Time dependent confocal images of NIH3T3 treated with 2 μM of PG@GD's library.

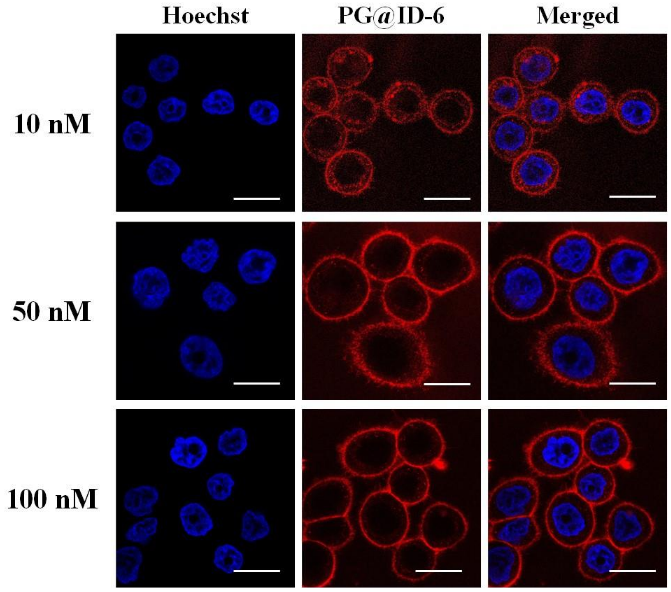

Fig. S7: Confocal images of MDA-MB-468 treated with PG@ID- 6 from 10 nM to 100 nM concentration after 30 min of incubation.

11. Co-Localization Assay in Cellular Level

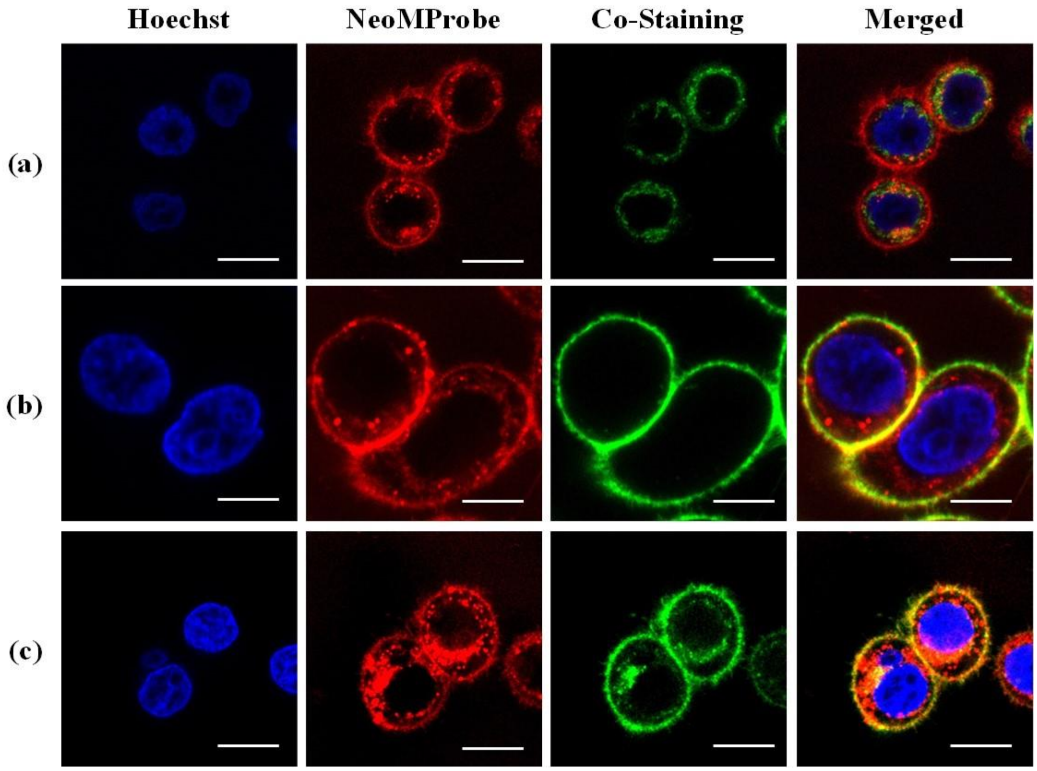

Fig. S8: Co-staining confocal images of MDA-MB-468 treated with NeoMProbe (PG@ID- 6) and **(a)** DiO **(b)** FITC-CD44 antibody and **(c)** FITC-E-cadherin antibody

12.Heparinase and sialidase Treated Assay

To investigate the glycocalyx anionic interaction modulating the activity, we first treated the cell with heparinase (0.03 IU/ml) for 1h at 37 °C, followed by sialidase (0.03 IU/ml) for 30 min at 4 °C respectively. Next cells incubated with **PG@ID-6 (NeoMProbe)** at 2 μM concentration. Our results showed significant amount of molecules internalized after 1 h and 2.5 h respectively.

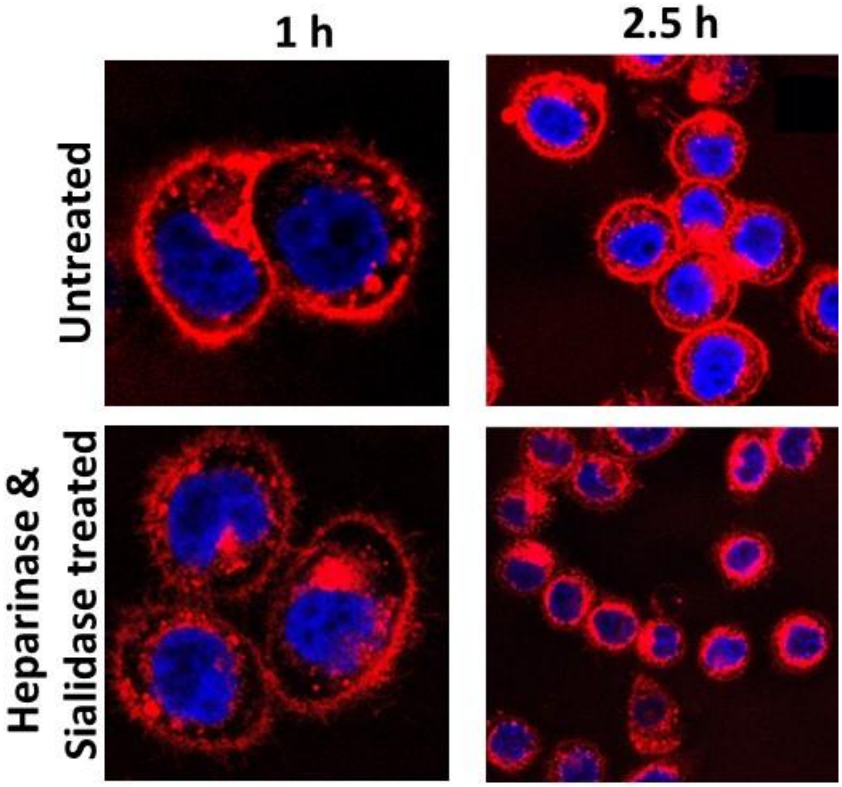

Fig. S9: NeoMProb plasma memebrane stability in the presence and absence of Heparinase and sialidase.

13. Anti-EGFR treated assay.

To investigate the EGFR receptor stabilizing the PG@ID-6. We first treated the cells with mice IgG anti-EGFR antiboby for (0.05 IU/ml) for 1 h and later inclubated with PG@ID-6 for 1 h and imaged. Our results showed significant decrease in the plasma membrane decoration of PG@ID-6, indicating that the molecules bind to cell surface receprot and stabilize.

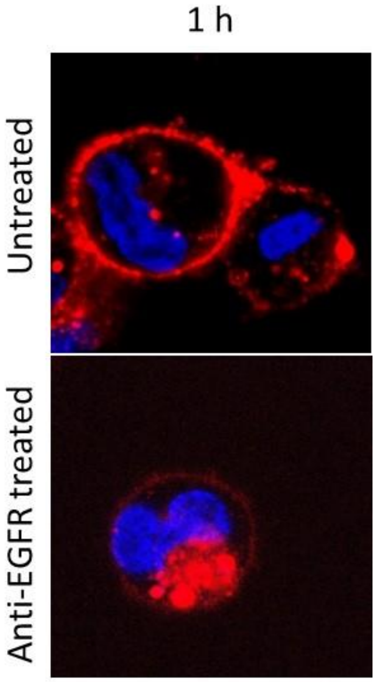

Fig S10. NeoMProb plasma memebrane stability in the presence and absence of anti-EGFR antibody.

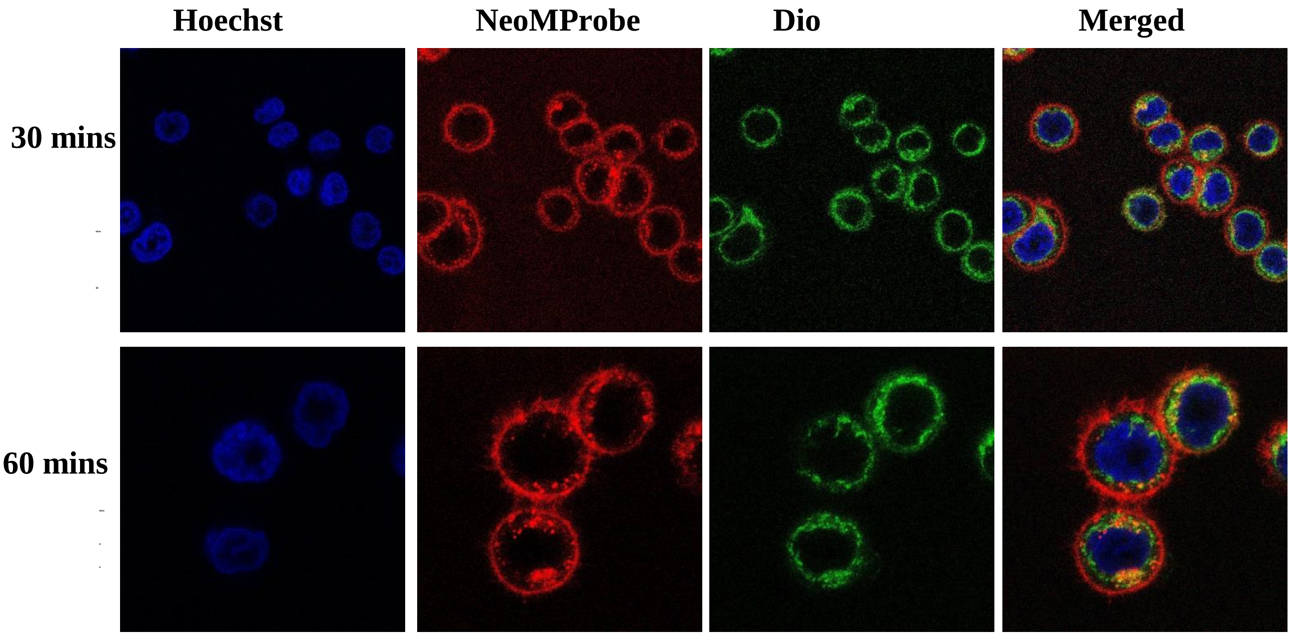

Figure S11. Costaining images of Dio and NeoMProbe of MDA-MB-468 after 30 and 60 mins.

12. FACS Analysis

Cells (5 × 10^5 cells per well) were seeded into a 24-well plate and incubated overnight. Subsequently, the cells were subjected to treatment with PG's mimetics for 1 hour. Followed by washing 3 times and re-suspended in FACS buffer for analysis.

13. Tissue Section Images

20a. *Ex vivo* tissue Section

To illustrate the potential of the PG's mimetics as a tissue membrane stanning, we stained the mouse tissue sections. For dewaxing of paraffin-embedded mouse tissue sections: Slides were initially heated at 60 °C for 15 minutes, then placed into glass slide holders and subjected to dewaxing as follows: Twice in 100% xylene wash for 5 minutes each, followed by immersion in 100% isopropanol with brief agitation (10–20 seconds) for 3 minutes, and finally twice in H_2O with brief agitation. For rehydration, the glass slides were immersed in PBS for 30 minutes. Subsequently, tissue sections were treated with a 2 µM and 500 nM of **PG@ID-6** for 1 hour at rt, followed by PBS washing for 3 minutes. Finally, sections were treated with Hoechst for 20 minutes at rt, followed by PBS washing 2 times for 3 minutes, and then subjected to confocal imaging.

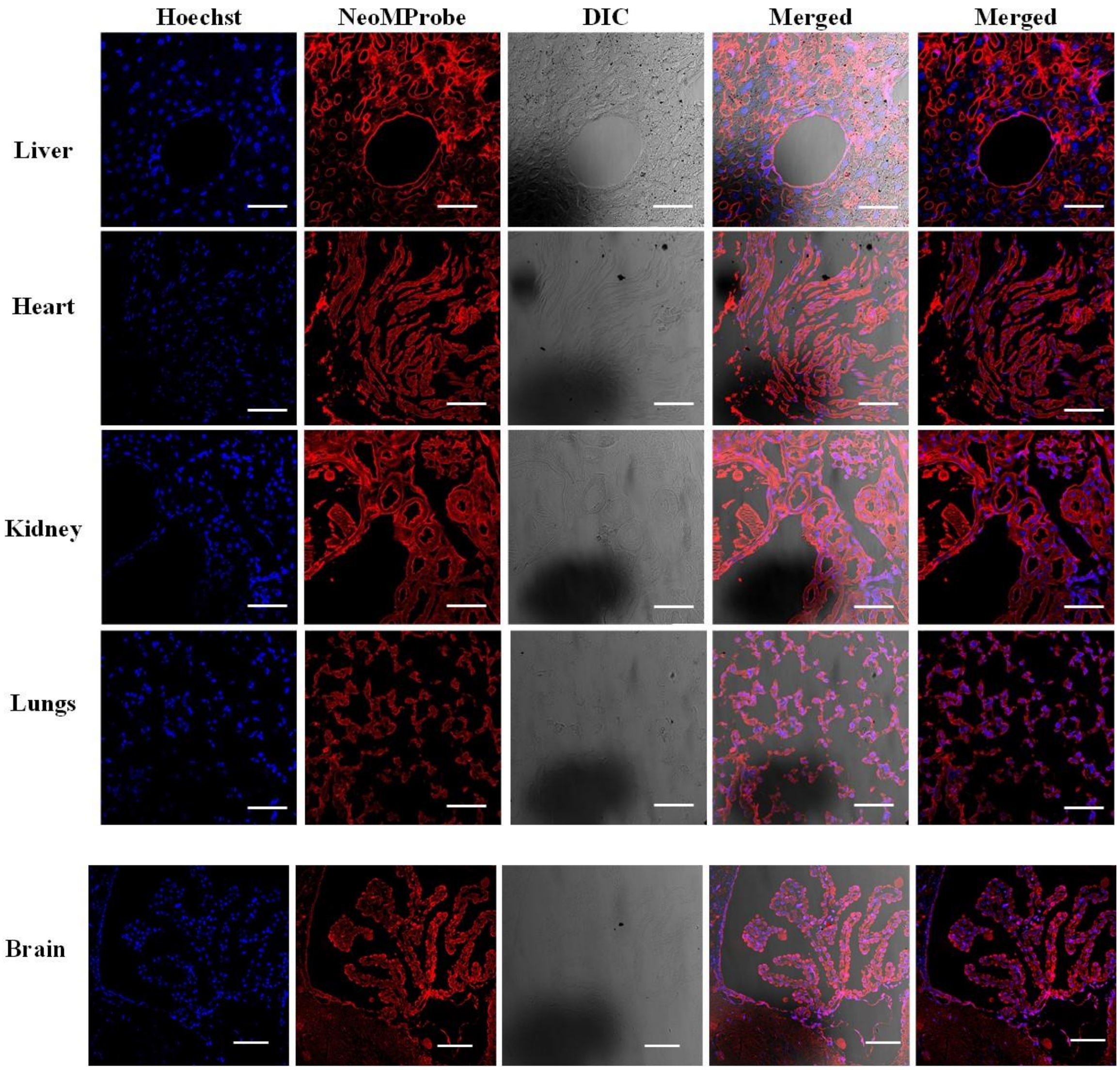

Fig. S12: Confocal images of tissue sections using NeoMProbe@500 nM concentration after 1 h of treatment

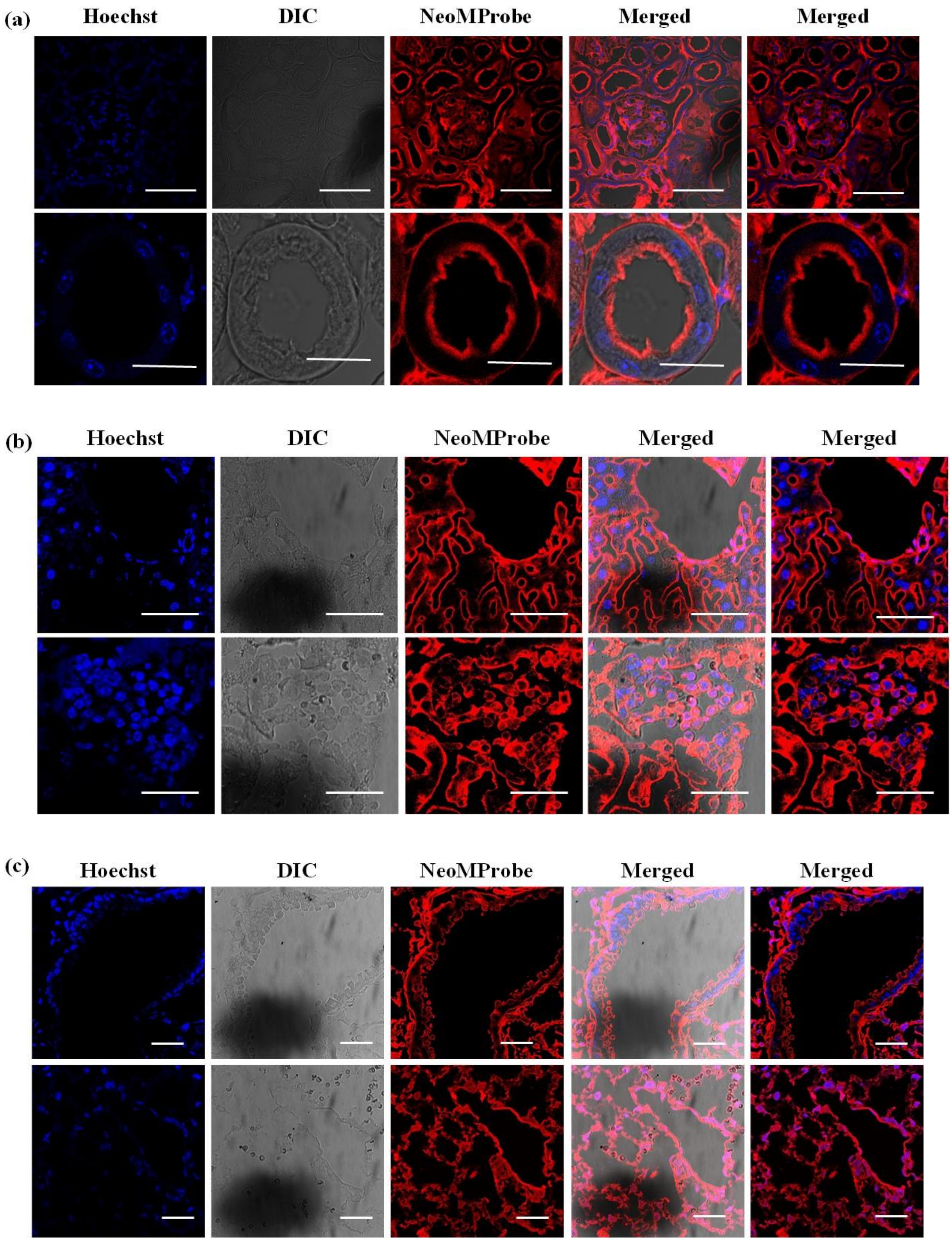
(a)
Hoechst
DIC
NeoMProbe
Merged
Merged
(b)
Hoechst
DIC
NeoMProbe
Merged
Merged
(c)
Hoechst
DIC
NeoMProbe
Merged
Merged

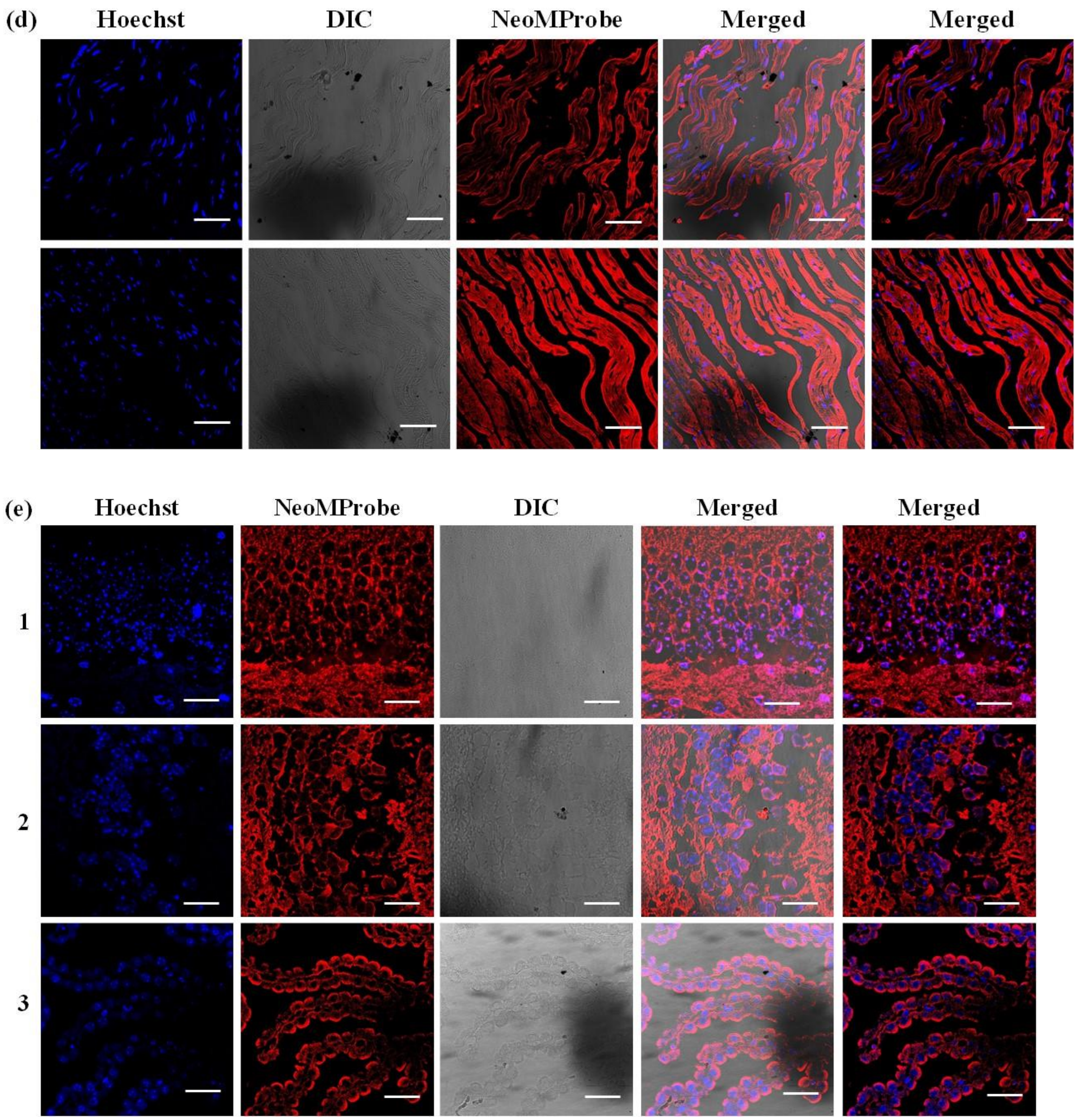

Fig. S13: Confocal images of tissue sections using NeoMProbe@2 µM concentration after 1 h of treatment **(a)** Kidney Section **(b)** Liver Section **(c)** Lungs Section **(d)** Heart Section **(e)** Brain section **(1.** Hippocampus region **2.** Cerebellum region **3.** Third ventricle region (V3)**)**

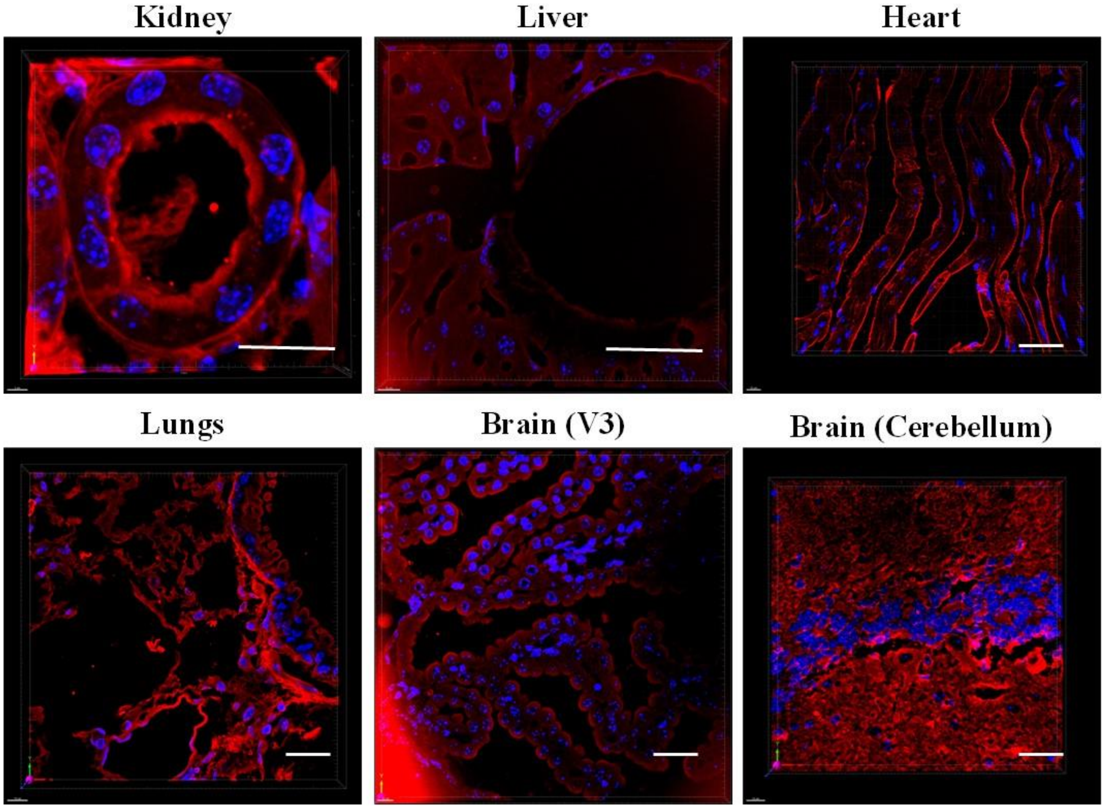

Fig. S14: Z stack images of tissue section using NeoMProbe@2 µM concentration

14. *Ex vivo* tissue Section Co-Staining

To illustrate the potential of the PG's mimetics as a tissue membrane stanning, we stained the mouse tissue sections. For dewaxing of paraffin-embedded mouse tissue sections: Slides were initially heated at 60 °C for 15 minutes, then placed into glass slide holders and subjected to dewaxing as follows: Twice in 100% xylene wash for 5 minutes each, followed by immersion in 100% isopropanol with brief agitation (10–20 seconds) for 3 minutes, and finally twice in H_2O with brief agitation. For rehydration, the glass side were immersed in PBS for 30 minutes:

(a) Subsequently, tissue sections were treated with a 2 µM of **PG@ID-6** and DiO ((3,3´ -dioctadecyloxacarbocyanine, perchlorate) for 30 minutes at rt, followed by PBS washing for 3 minutes. Finally, sections were treated with Hoechst for 20 minutes at rt, followed by PBS washing 2 times for 3 minutes, and then subjected to confocal imaging.

(b) Subsequently, it was treated with 0.1 % trypsin for 1 h followed by washing with PBS for 5 min 2 times. After that permeabilization was done using mixture of PBS and 0.25 % of triton X100 followed by washing with PBS for 5 min 2 times. Then, using 1% CD-44 anti-body in PBS

stanning was done for 2 h at rt followed by washing with PBS for 5 min 2 times. Finally, tissue sections were treated with a 2 µM of **PG@ID-6** and Hoechst for 30 minutes at rt, followed by PBS washing for 3 minutes 2 times, and then subjected to confocal imaging.

(c) Subsequently, it was treated with 0.1 % trypsin for 1 h followed by washing with PBS for 5 min 2 times. After that permeabilization was done using mixture of PBS, gelatin 1:1 and 0.25 % of triton X100 followed by washing with PBS for 5 min 2 times. Then, using 1% E-cadherin anti-body in PBS stanning was done for 3 h at rt followed by washing with PBS for 5 min 2 times. Finally, tissue sections were treated with a 2 µM of **PG@ID-6** and Hoechst for 30 minutes at rt, followed by PBS washing for 3 minutes 2 times, and then subjected to confocal imaging.

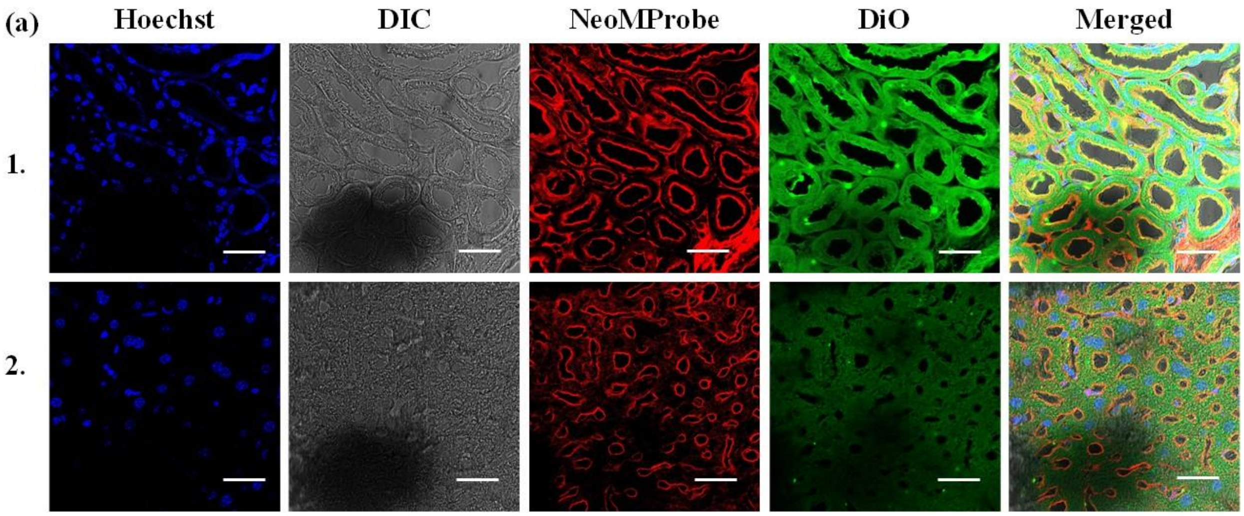

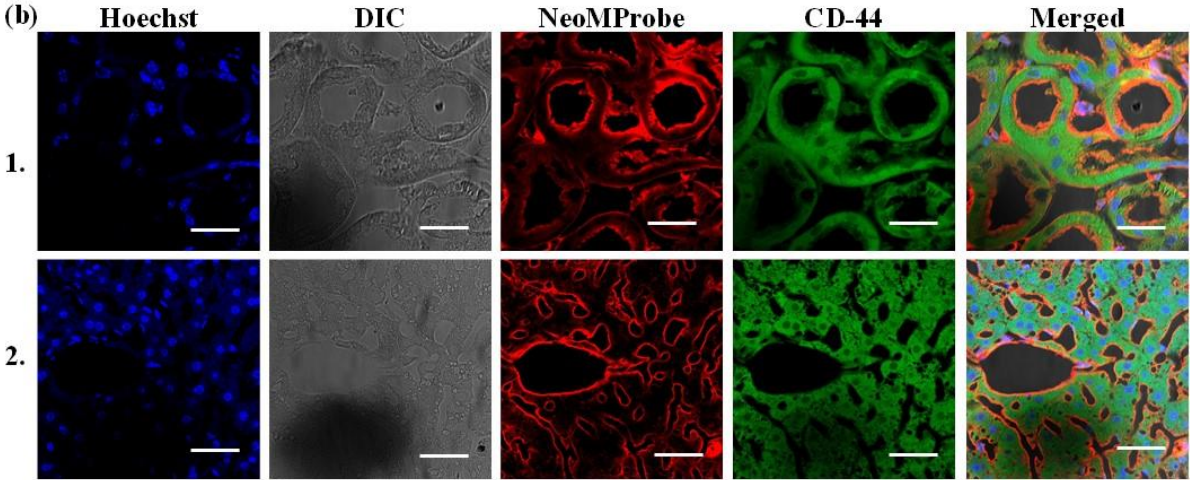

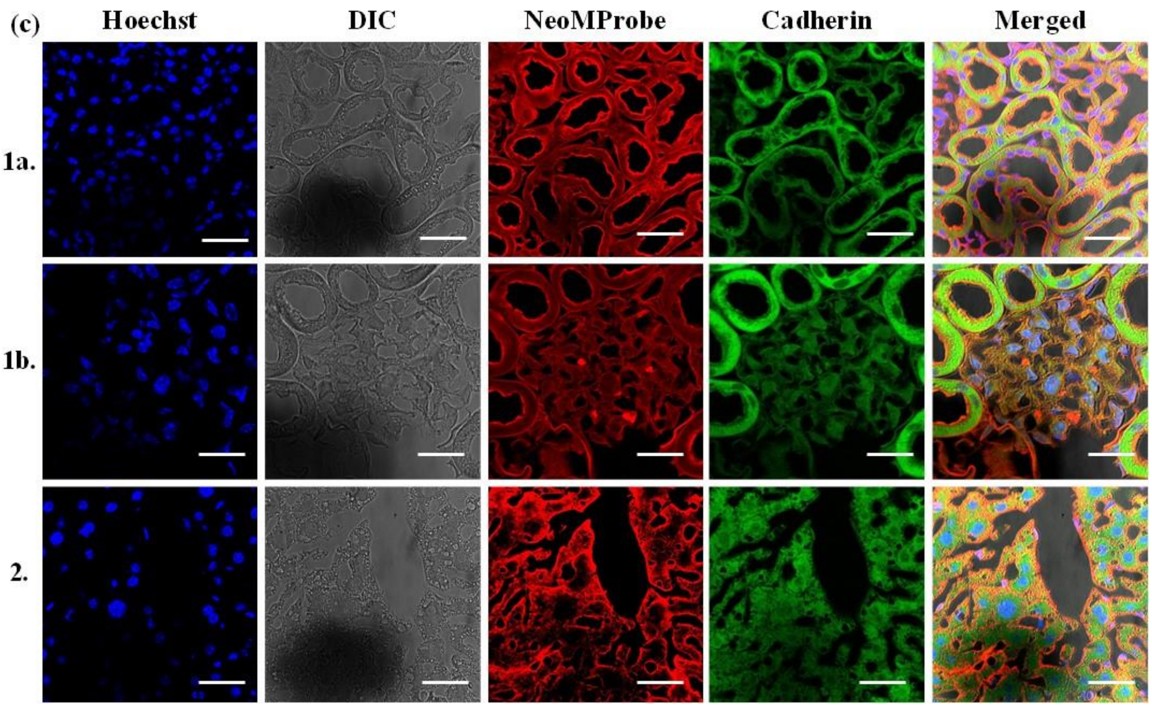

Fig. S15: Co-stanning confocal images of **1** is kidney and **2** is liver using **(a)** DiO **(b)** CD-44 **(c)** E-Cadherin

15. Animal Studies

15a. *In vivo* Studies

BALB/c 6-8 weeks old were taken from the National Facility for Gene Function in Health and Disease of IISER, Pune. Prior to experiments the mice were maintained in an animal house, with the proper amount of food and water. All experiments were performed in accordance with the relevant guidelines and regulations of the Institutional Animal Ethical Committee, set up by CPCSEA, Govt. of India. The mice (n = 3) were anesthetized by ketamine. After that **PG@ID-6 (NeoMProbe)** 1.8 mg/Kg were injected intraperitoneal. Then *in vivo* imaging was done using Quantum GX2 Micro-CT system (*excitation = 640 nm and emission = 680 nm*). First, before injection image was taken marked at 0 h, after injection at different time interval imaging was done upto 24 h. One mouse was sacrificed after 3 h. Subsequently, major organs including kidney and liver were collected and fixed with 4% paraformaldehyde. Organs were embedded in paraffin and 1 mm sections were cut using a microtome. The tissue sections were fixed on a poly- L -lysine-coated glass plates.

15b. Post Injection *in vivo* Tissue Section

To illustrate the potential of the PG's mimetics as an *in vivo* tissue membrane stain, we stained the mouse tissue sections. For dewaxing of paraffin-embedded mouse tissue sections: Slides were initially heated at 60 °C for 15 minutes, then placed into glass slide holders and subjected to dewaxing as follows: Twice in 100% xylene wash for 5 minutes each, followed by immersion in 100% isopropanol with brief agitation (10–20 seconds) for 3 minutes, and finally twice in H_2O with brief agitation. For rehydration, the glass slides were immersed in PBS for 30 minutes. Subsequently, tissue was treated with Hoechst for 20 minutes at rt, followed by PBS washing 2 times for 3 minutes, and then subjected to confocal imaging.

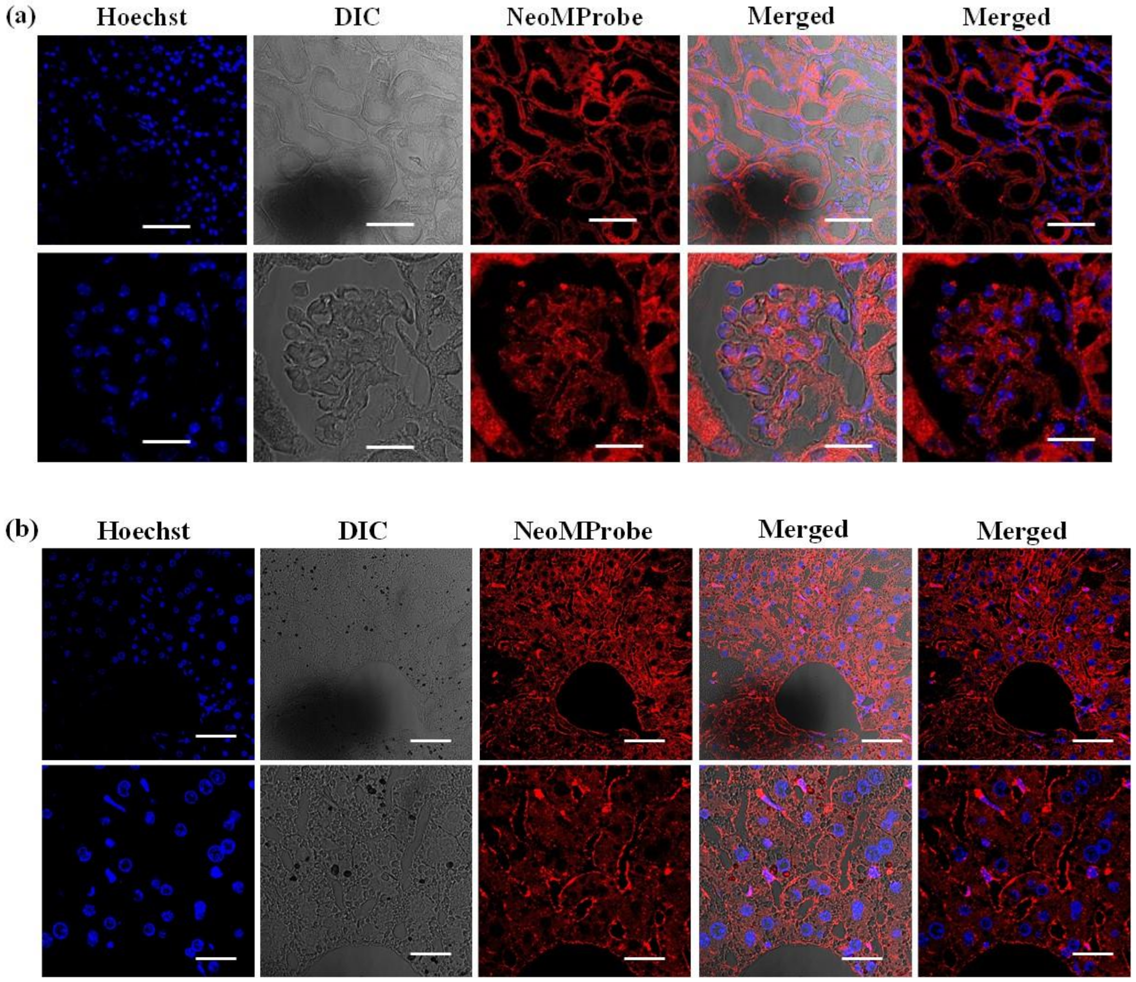

Fig. S16: Confocal images of tissue sections using NeoMProbe post injection **(a)** Kidney Section **(b)** Liver Section

15c. Post Injection *in vivo* Tissue Section Co-Staining

To illustrate the potential of the PG's mimetics as a tissue membrane stanning, we stained the mouse tissue sections. For dewaxing of paraffin-embedded mouse tissue sections: Slides were initially heated at 60 °C for 15 minutes, then placed into glass slide holders and subjected to dewaxing as follows: Twice in 100% xylene wash for 5 minutes each, followed by immersion in 100% isopropanol with brief agitation (10–20 seconds) for 3 minutes, and finally twice in H_2O with brief agitation. For rehydration, the glass slides were immersed in PBS for 30 minutes. Subsequently, it was treated with 0.1 % trypsin for 1 h followed by washing with PBS for 5 min 2 times. After that permeabilization was done using mixture of PBS, gelatin 1:1 and 0.25 % of triton X100 followed by washing with PBS for 5 min 2 times. Then, using 1% E-cadherin anti-body in PBS stanning was done for 3 h at rt followed by washing with PBS for 5 min 2 times. Finally, tissue sections were treated with a 2 µM of **PG@ID-6** and Hoechst for 30 minutes at rt, followed by PBS washing for 3 minutes 2 times, and then subjected to confocal imaging.

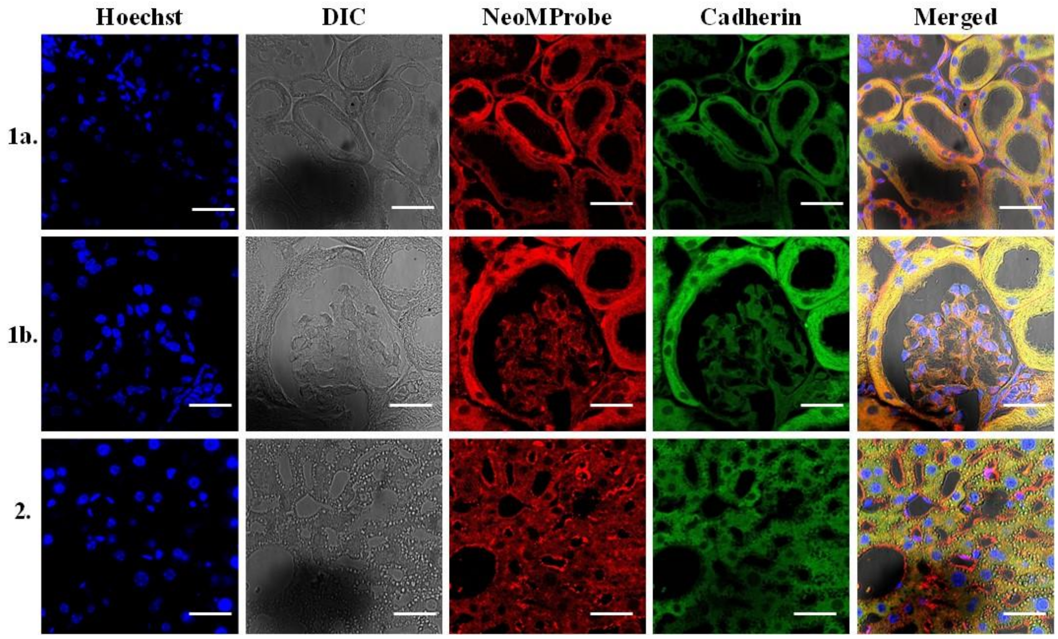

Fig. S17: Co-stanning confocal images of **1a** and **1b** is kidney and **2** is liver using E-Cadherin

18. NMR and other Characterization Technique

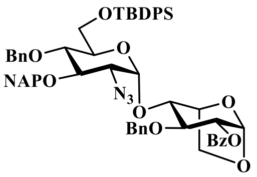

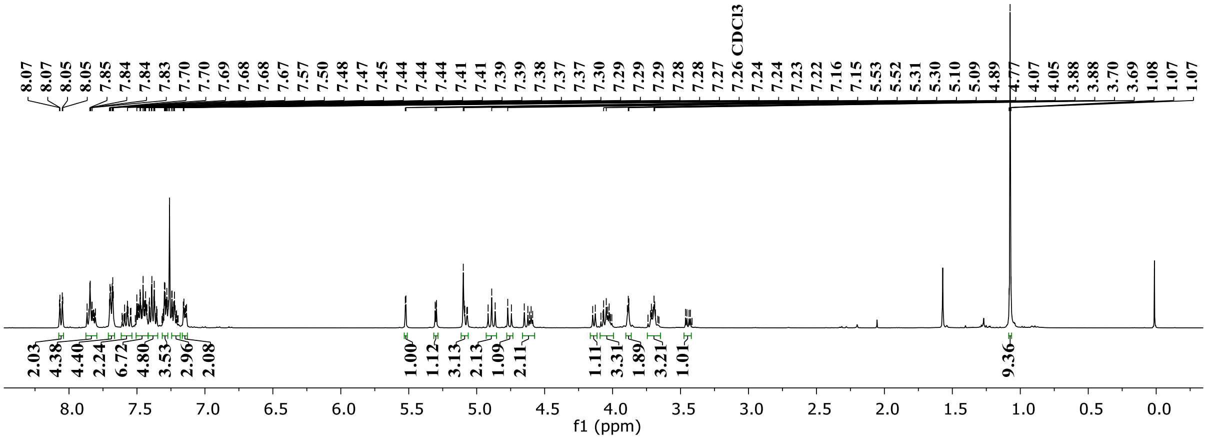

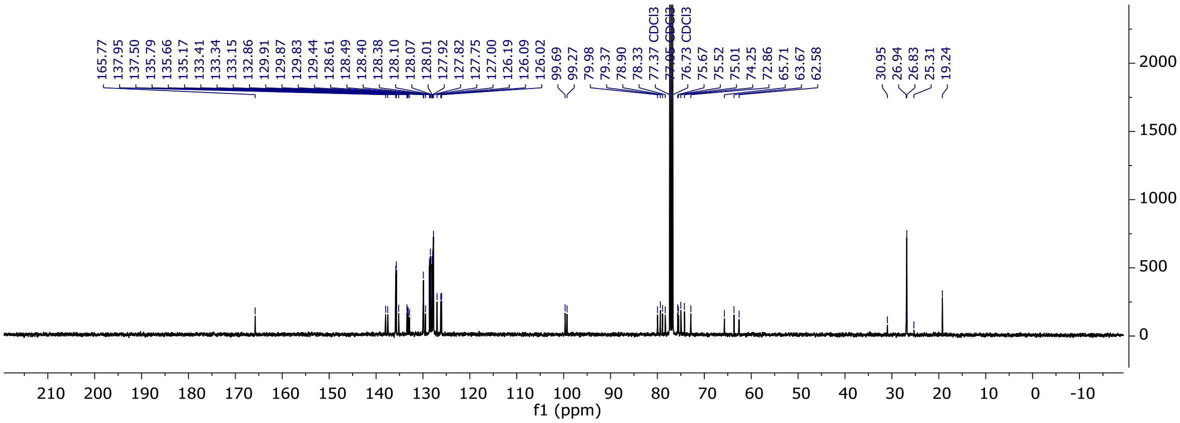

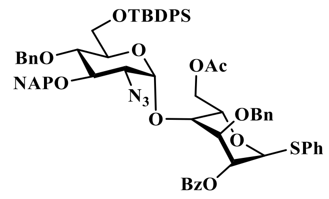
OTBDPS
BnO
O
NAPO
N_3
O
OAc
OBn
O
SPh
BzO

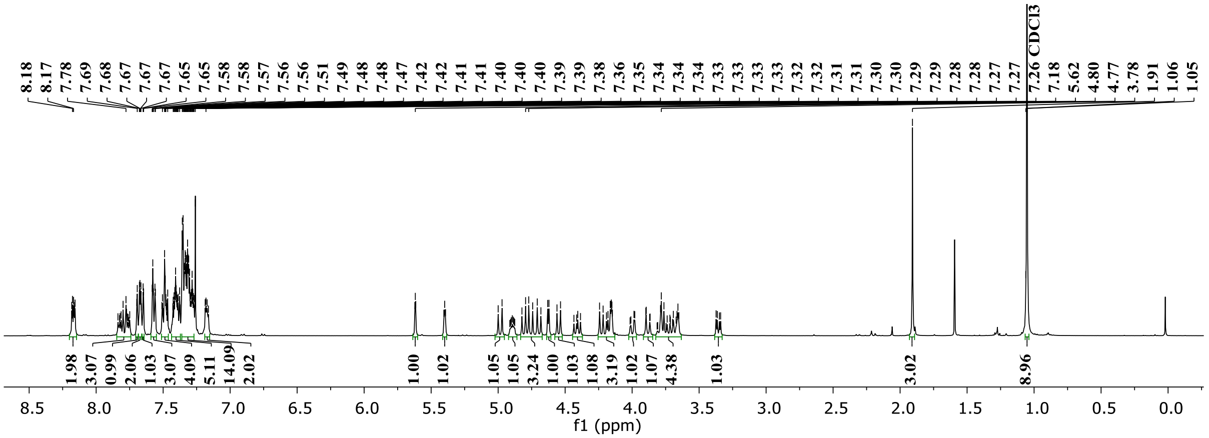
8.18 8.17 7.78 7.69 7.68 7.67 7.67 7.67 7.65 7.65 7.58 7.58 7.57 7.56 7.56 7.51 7.49 7.48 7.48 7.47 7.42 7.42 7.41 7.41 7.40 7.40 7.40 7.39 7.39 7.38 7.36 7.35 7.34 7.34 7.34 7.33 7.33 7.33 7.33 7.32 7.32 7.31 7.31 7.30 7.30 7.29 7.29 7.28 7.28 7.27 7.27 7.26 CDCl3 7.18 5.62 4.80 4.77 3.78 1.91 1.06 1.05
1.98 3.07 0.99 2.06 1.03 3.07 4.09 5.11 14.09 2.02 1.00 1.02 1.05 1.05 3.24 1.00 1.03 1.08 3.19 1.02 1.07 4.38 1.03 3.02 8.96
8.5 8.0 7.5 7.0 6.5 6.0 5.5 5.0 4.5 4.0 3.5 3.0 2.5 2.0 1.5 1.0 0.5 0.0
f1 (ppm)

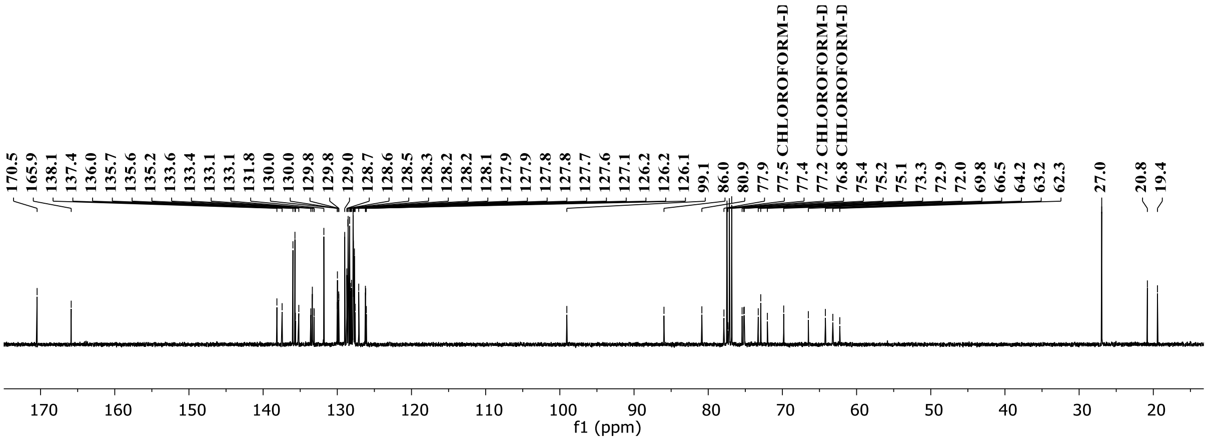
170.5 165.9 138.1 137.4 136.0 135.7 135.6 135.2 133.6 133.4 133.1 133.1 131.8 130.0 130.0 129.8 129.8 129.0 128.7 128.6 128.5 128.3 128.2 128.2 128.1 127.9 127.9 127.8 127.8 127.7 127.6 127.1 126.2 126.1 99.1 86.0 80.9 77.9 77.5 CHLOROFORM-D 77.4 77.2 CHLOROFORM-D 76.8 CHLOROFORM-D 75.4 75.2 75.1 73.3 72.9 72.0 69.8 66.5 64.2 63.2 62.3 27.0 20.8 19.4
170 160 150 140 130 120 110 100 90 80 70 60 50 40 30 20
f1 (ppm)

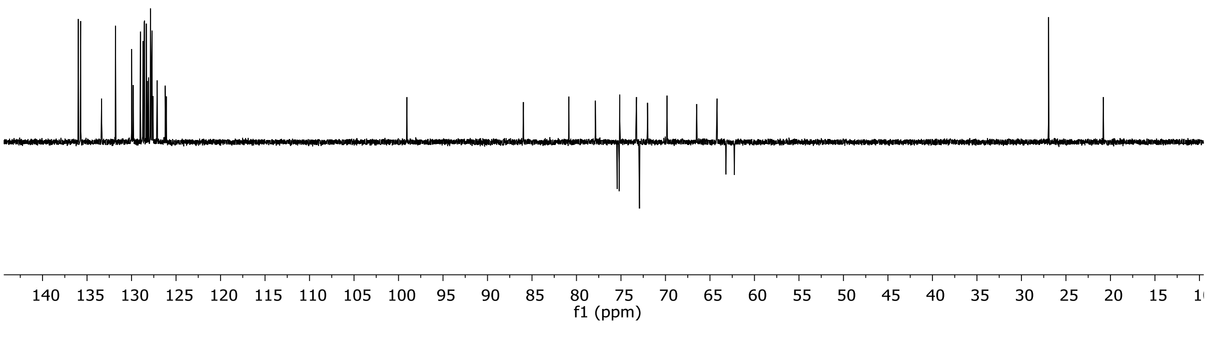
140 135 130 125 120 115 110 105 100 95 90 85 80 75 70 65 60 55 50 45 40 35 30 25 20 15
f1 (ppm)

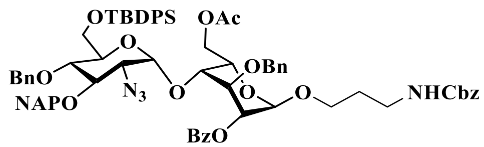
OTBDPS
OAc
BnO
OBn
NAPO
N_3
O
NHCbz
BzO

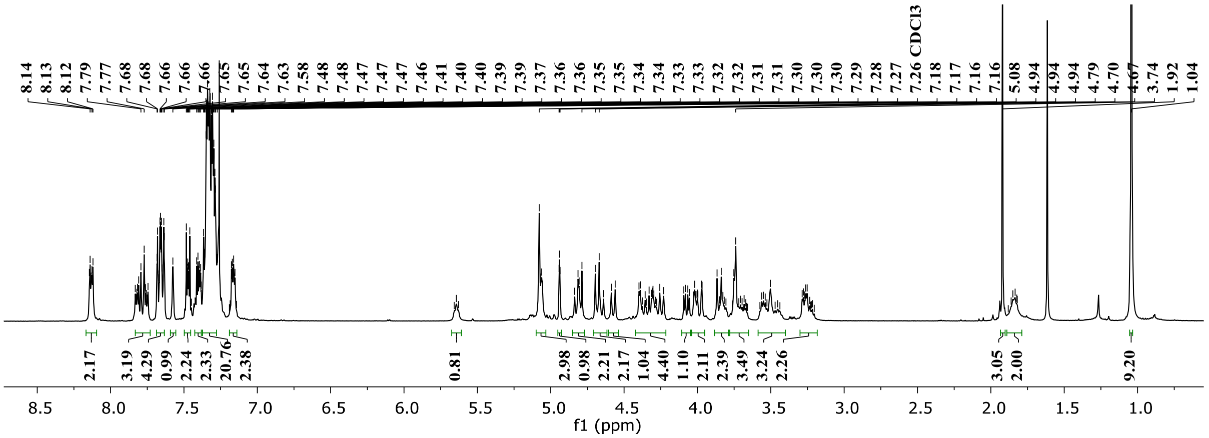
8.14 8.13 8.12 7.79 7.77 7.68 7.68 7.66 7.66 7.66 7.65 7.65 7.64 7.63 7.58 7.48 7.48 7.47 7.47 7.47 7.46 7.41 7.40 7.40 7.39 7.39 7.37 7.36 7.36 7.35 7.35 7.34 7.34 7.33 7.33 7.32 7.32 7.31 7.31 7.30 7.30 7.30 7.29 7.28 7.27 7.26 CDCl3 7.18 7.17 7.16 7.16 5.08 4.94 4.94 4.94 4.79 4.70 4.67 3.74 1.92 1.04
2.17 3.19 4.29 0.99 2.24 2.33 20.76 2.38 0.81 2.98 0.98 2.21 2.17 1.04 4.40 1.10 2.11 2.39 3.49 3.24 2.26 3.05 2.00 9.20
8.5 8.0 7.5 7.0 6.5 6.0 5.5 5.0 4.5 4.0 3.5 3.0 2.5 2.0 1.5 1.0
f1 (ppm)

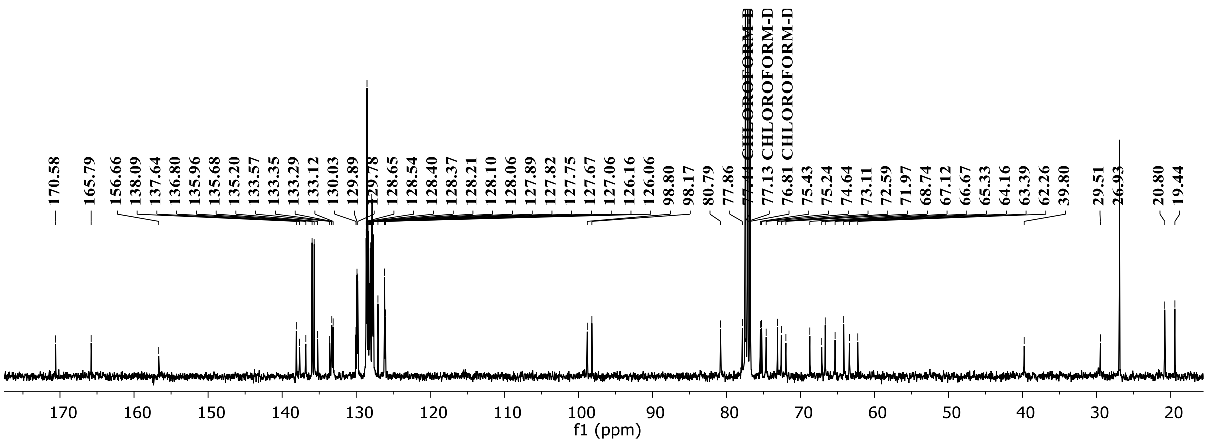
170.58 165.79 156.66 138.09 137.64 136.80 135.96 135.68 135.20 133.57 133.35 133.29 133.12 130.03 129.89 129.78 128.65 128.54 128.40 128.37 128.21 128.10 128.06 127.89 127.82 127.75 127.67 127.06 126.16 126.06 98.80 98.17 80.79 77.86 77.44 CHLOROFORM-D 77.13 CHLOROFORM-D 76.81 CHLOROFORM-D 75.43 75.24 74.64 73.11 72.59 71.97 68.74 67.12 66.67 65.33 64.16 63.39 62.26 39.80 29.51 26.93 20.80 19.44
170 160 150 140 130 120 110 100 90 80 70 60 50 40 30 20
f1 (ppm)

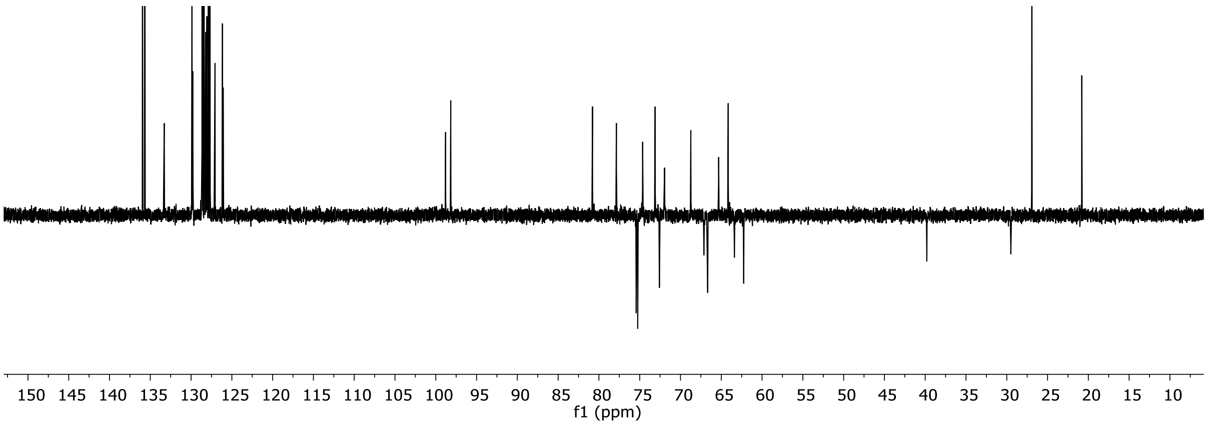
150 145 140 135 130 125 120 115 110 105 100 95 90 85 80 75 70 65 60 55 50 45 40 35 30 25 20 15 10
f1 (ppm)

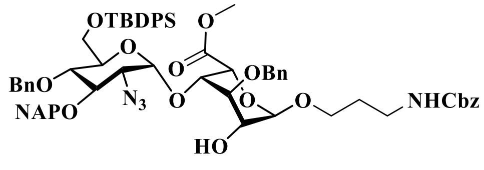
OTBDPS
O
O
O
OBn
BnO
NAPO
N_3
O
O
O
NHCbz
HO

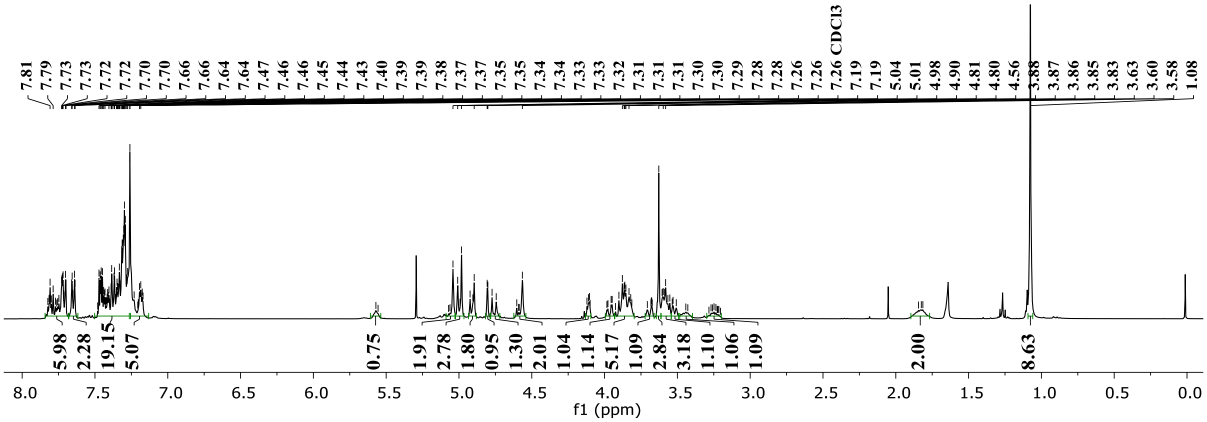
7.81
7.79
7.73
7.73
7.72
7.72
7.70
7.70
7.66
7.66
7.64
7.64
7.47
7.46
7.46
7.45
7.44
7.43
7.40
7.39
7.39
7.38
7.37
7.37
7.35
7.35
7.34
7.34
7.33
7.33
7.32
7.31
7.31
7.31
7.30
7.30
7.29
7.28
7.28
7.26
7.26
7.26 CDCl3
7.19
7.19
5.04
5.01
4.98
4.90
4.81
4.80
4.56
3.88
3.87
3.86
3.85
3.83
3.63
3.60
3.58
1.08
5.98
2.28
19.15
5.07
0.75
1.91
2.78
1.80
0.95
1.30
2.01
1.04
1.14
5.17
1.09
2.84
3.18
1.10
1.06
1.09
2.00
8.63
8.0 7.5 7.0 6.5 6.0 5.5 5.0 4.5 4.0 3.5 3.0 2.5 2.0 1.5 1.0 0.5 0.0
f1 (ppm)

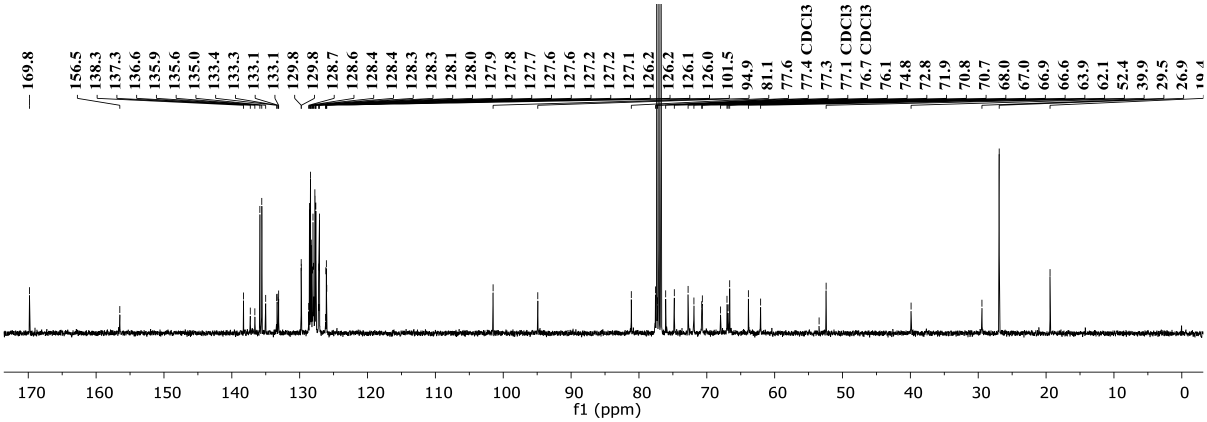
169.8
156.5
138.3
137.3
136.6
135.9
135.6
135.0
133.4
133.3
133.1
133.1
129.8
129.8
128.7
128.6
128.4
128.4
128.3
128.3
128.1
128.0
127.9
127.8
127.7
127.6
127.6
127.2
127.2
127.1
126.2
126.2
126.1
126.0
101.5
94.9
81.1
77.6
77.4 CDCl3
77.3
77.1 CDCl3
76.7 CDCl3
76.1
74.8
72.8
71.9
70.8
70.7
68.0
67.0
66.9
66.6
63.9
62.1
52.4
39.9
29.5
26.9
170 160 150 140 130 120 110 100 90 80 70 60 50 40 30 20 10 0
f1 (ppm)

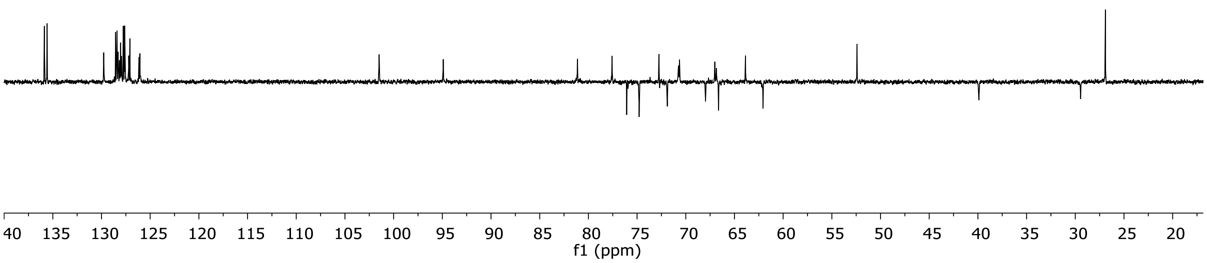
40 135 130 125 120 115 110 105 100 95 90 85 80 75 70 65 60 55 50 45 40 35 30 25 20
f1 (ppm)

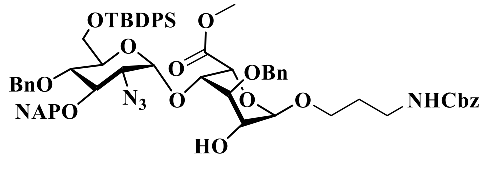
OTBDPS
O
BnO
NAPO
N3
O
O
OBn
O
O
NHCbz
HO

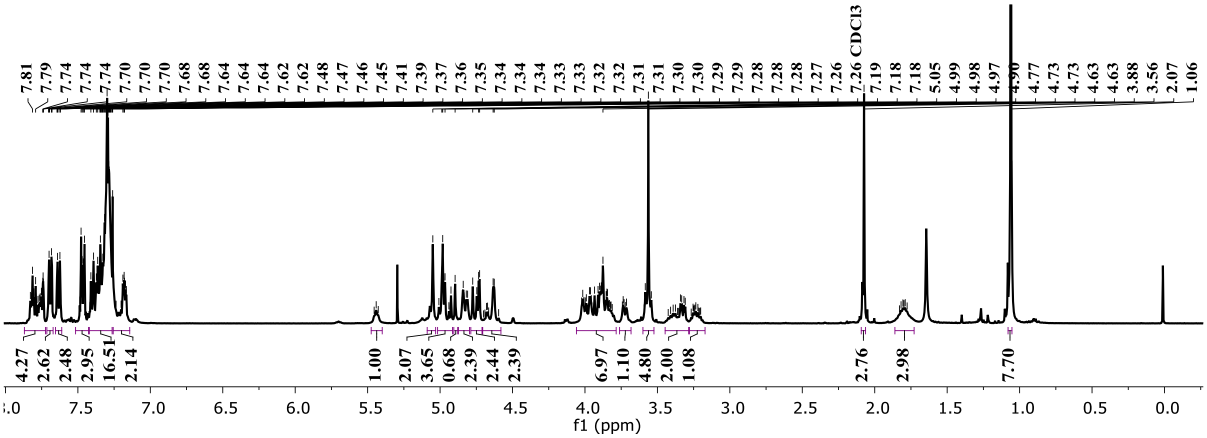
7.81
7.79
7.74
7.74
7.74
7.70
7.70
7.68
7.68
7.64
7.64
7.64
7.62
7.62
7.48
7.47
7.46
7.45
7.41
7.39
7.37
7.36
7.35
7.34
7.34
7.34
7.33
7.33
7.32
7.32
7.31
7.31
7.30
7.30
7.29
7.29
7.28
7.28
7.28
7.27
7.26
7.26 CDCl3
7.19
7.18
7.18
5.05
4.99
4.98
4.97
4.90
4.77
4.73
4.73
4.63
4.63
3.88
3.56
2.07
1.06
4.27
2.62
2.48
2.95
16.51
2.14
1.00
2.07
3.65
0.68
2.39
2.44
2.39
6.97
1.10
4.80
2.00
1.08
2.76
2.98
7.70
7.5
7.0
6.5
6.0
5.5
5.0
4.5
4.0
3.5
3.0
2.5
2.0
1.5
1.0
0.5
0.0
f1 (ppm)

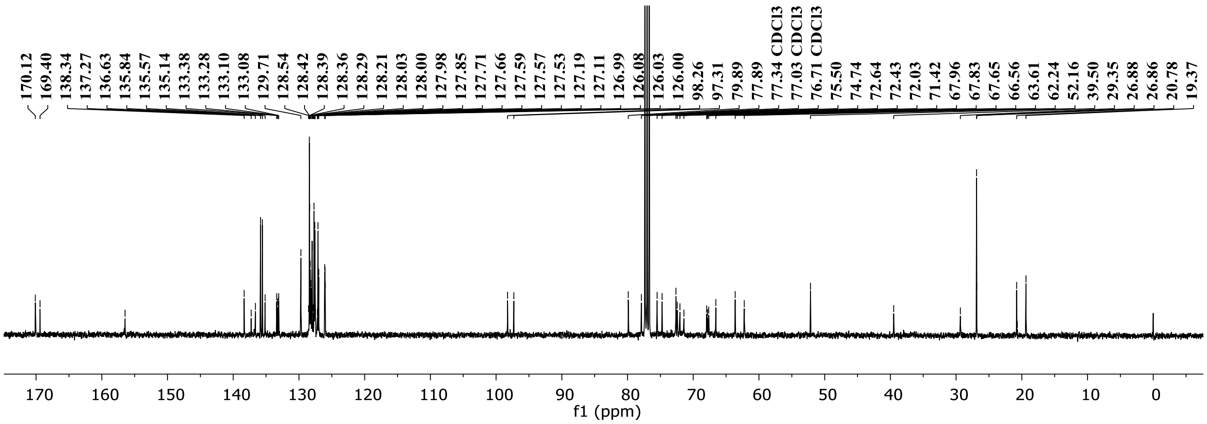
170.12
169.40
138.34
137.27
136.63
135.84
135.57
135.14
133.38
133.28
133.10
133.08
129.71
128.54
128.42
128.39
128.36
128.29
128.21
128.03
128.00
127.98
127.85
127.71
127.66
127.59
127.57
127.53
127.19
127.11
126.99
126.08
126.03
126.00
98.26
97.31
79.89
77.89
77.34 CDCl3
77.03 CDCl3
76.71 CDCl3
75.50
74.74
72.64
72.43
72.03
71.42
67.96
67.83
67.65
66.56
63.61
62.24
52.16
39.50
29.35
26.88
26.86
20.78
19.37
170
160
150
140
130
120
110
100
90
80
70
60
50
40
30
20
10
0
f1 (ppm)

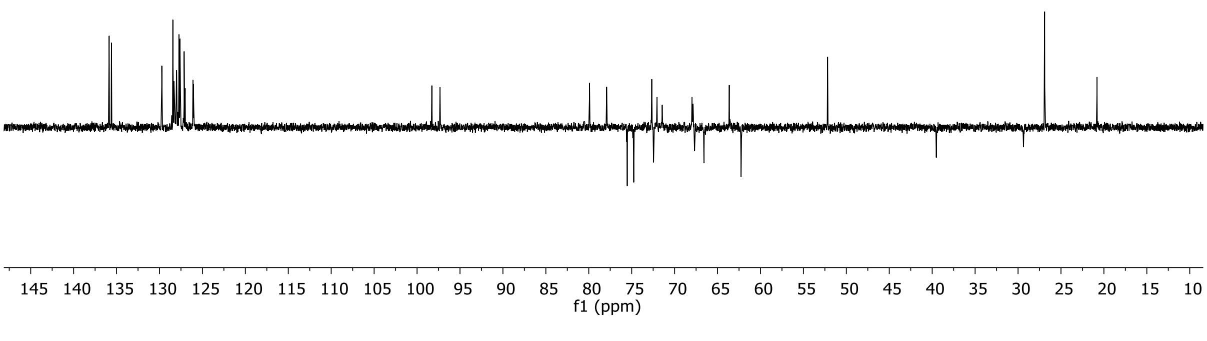
145
140
135
130
125
120
115
110
105
100
95
90
85
80
75
70
65
60
55
50
45
40
35
30
25
20
15
10
f1 (ppm)

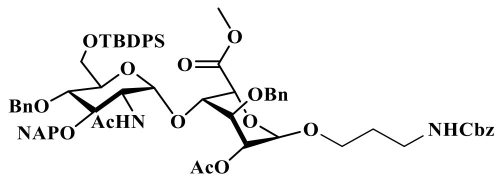
OTBDPS
O
BnO
NAPO
AcHN
O
O
OBn
O
O
NHCbz
AcO

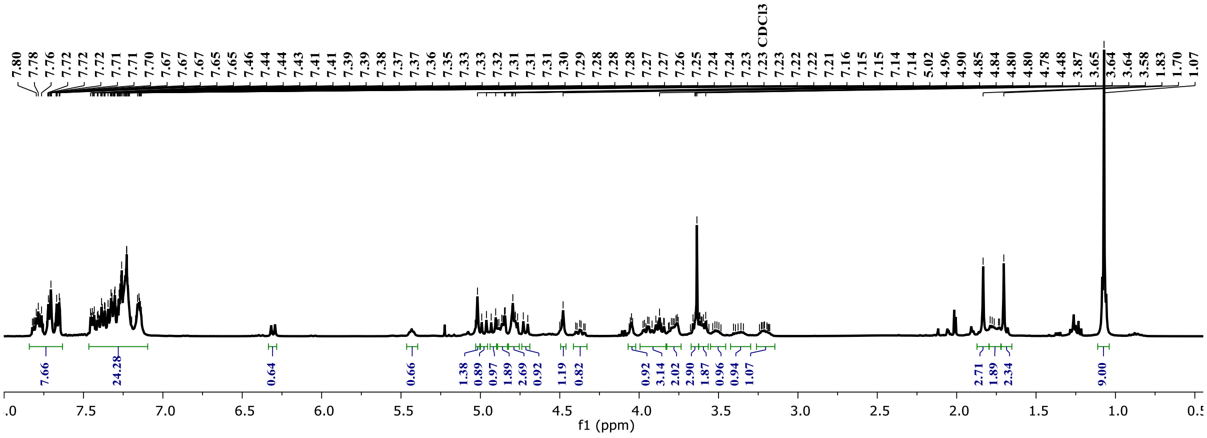
7.80 7.78 7.76 7.72 7.72 7.72 7.71 7.71 7.70 7.67 7.67 7.67 7.65 7.65 7.46 7.44 7.44 7.43 7.41 7.41 7.39 7.39 7.38 7.37 7.37 7.36 7.35 7.33 7.33 7.32 7.31 7.31 7.31 7.30 7.29 7.28 7.28 7.28 7.27 7.27 7.26 7.25 7.24 7.24 7.23 7.23 7.23 7.22 7.22 7.21 7.16 7.15 7.15 7.14 7.14 5.02 4.96 4.90 4.85 4.84 4.80 4.80 4.78 4.48 3.87 3.65 3.64 3.64 3.58 1.83 1.70 1.07
CDCl3
7.66 24.28 0.64 0.66 1.38 0.89 0.97 1.89 2.69 0.92 1.19 0.82 0.92 3.14 2.02 2.90 1.87 0.96 0.94 1.07 2.71 1.89 2.34 9.00
.0 7.5 7.0 6.5 6.0 5.5 5.0 4.5 4.0 3.5 3.0 2.5 2.0 1.5 1.0 0.5
f1 (ppm)

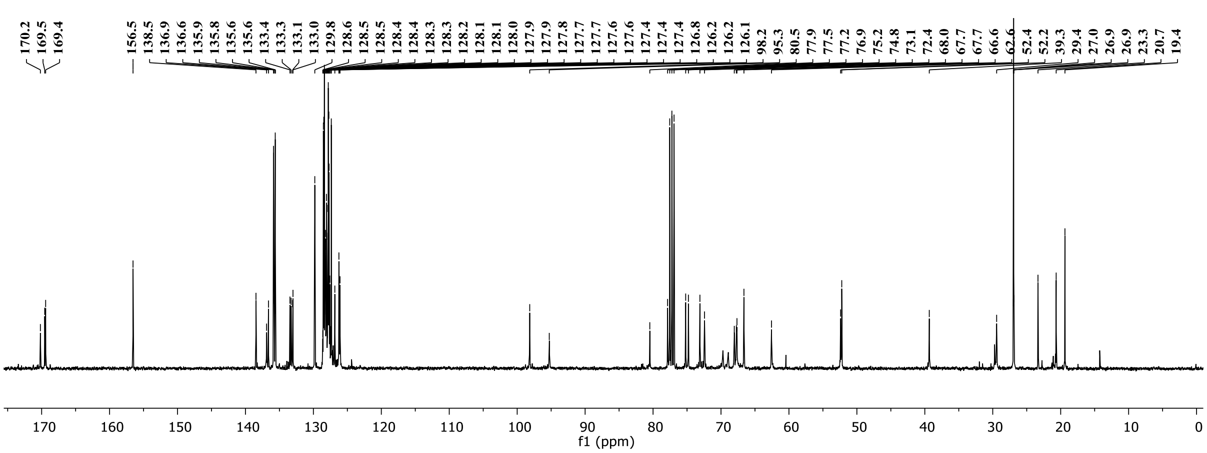
170.2 169.5 169.4 156.5 138.5 136.9 136.6 135.9 135.8 135.6 135.6 133.4 133.3 133.1 133.0 129.8 128.6 128.5 128.5 128.4 128.4 128.3 128.3 128.2 128.1 128.1 128.0 127.9 127.9 127.8 127.7 127.7 127.6 127.4 127.4 127.4 126.8 126.2 126.2 126.1 98.2 95.3 80.5 77.9 77.5 77.2 76.9 75.2 74.8 73.1 72.4 68.0 67.7 67.7 66.6 62.6 52.4 52.2 39.3 29.4 27.0 26.9 26.9 23.3 20.7 19.4
170 160 150 140 130 120 110 100 90 80 70 60 50 40 30 20 10 0
f1 (ppm)

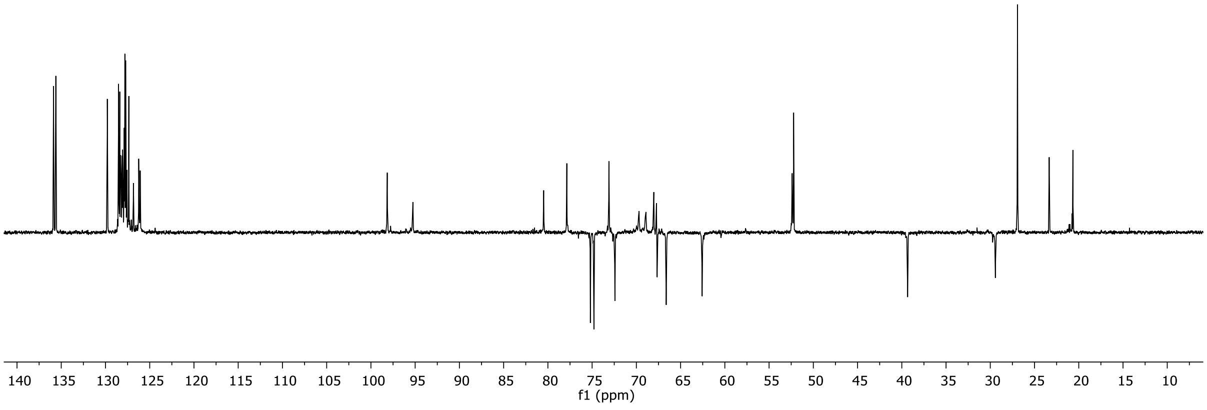
140 135 130 125 120 115 110 105 100 95 90 85 80 75 70 65 60 55 50 45 40 35 30 25 20 15 10
f1 (ppm)

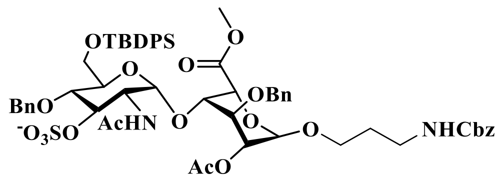
OTBDPS
BnO
^-O_3SO
AcHN
O
O
OBn
O
AcO
NHCbz

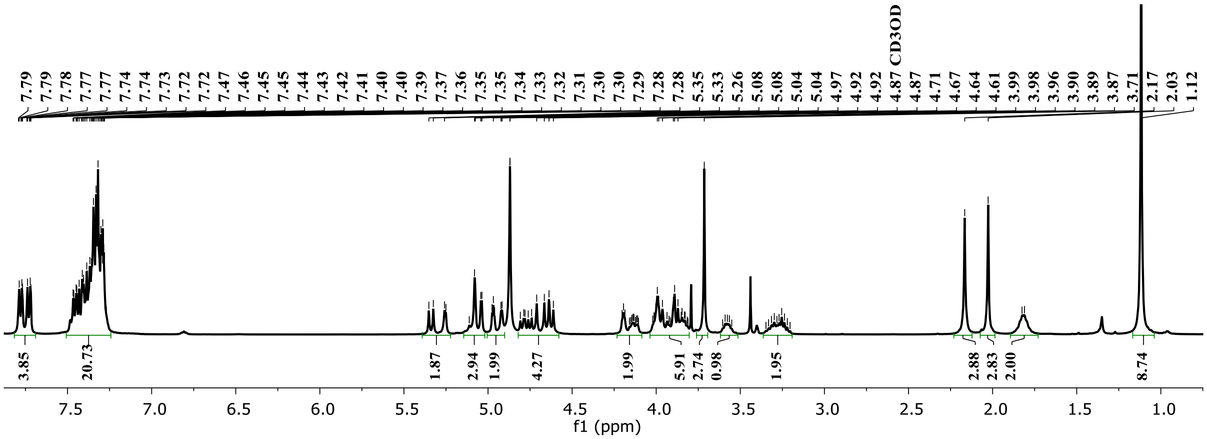
7.79 7.79 7.78 7.77 7.77 7.74 7.74 7.73 7.72 7.72 7.47 7.46 7.45 7.45 7.44 7.43 7.42 7.41 7.40 7.40 7.39 7.37 7.36 7.35 7.35 7.34 7.33 7.32 7.31 7.30 7.30 7.29 7.28 7.28 5.35 5.33 5.26 5.08 5.08 5.04 5.04 4.97 4.92 4.92 4.87 CD3OD 4.87 4.71 4.67 4.64 4.61 3.99 3.98 3.96 3.90 3.89 3.87 3.71 2.17 2.03 1.12
3.85 20.73 1.87 2.94 1.99 4.27 1.99 5.91 2.74 0.98 1.95 2.88 2.83 2.00 8.74
7.5 7.0 6.5 6.0 5.5 5.0 4.5 4.0 3.5 3.0 2.5 2.0 1.5 1.0
f1 (ppm)

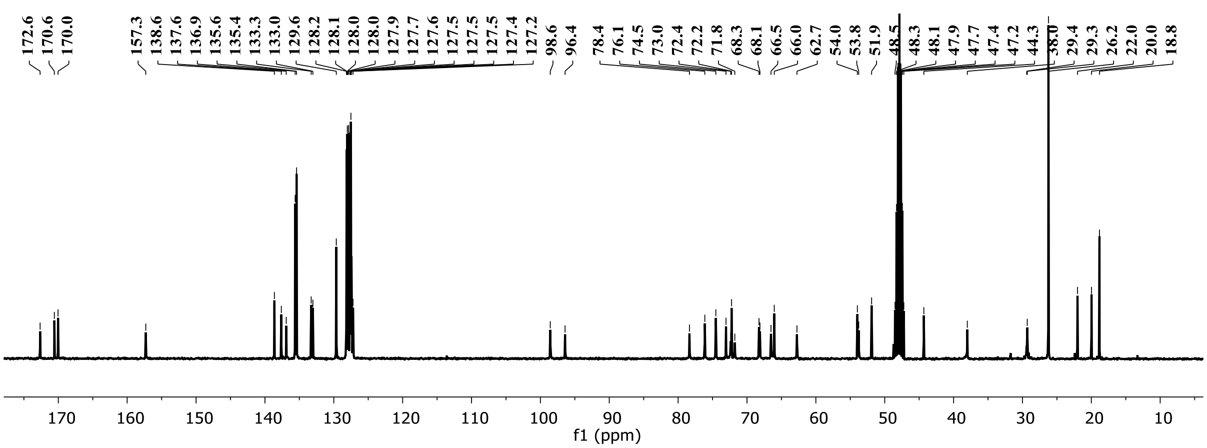
172.6 170.6 170.0 157.3 138.6 137.6 136.9 135.6 135.4 133.3 133.0 129.6 128.2 128.1 128.0 128.0 127.9 127.7 127.6 127.5 127.5 127.4 127.2 98.6 96.4 78.4 76.1 74.5 73.0 72.4 72.2 71.8 68.3 68.1 66.5 66.0 62.7 54.0 53.8 51.9 48.5 48.3 48.1 47.9 47.7 47.4 47.2 44.3 38.0 29.4 29.3 26.2 22.0 20.0 18.8
170 160 150 140 130 120 110 100 90 80 70 60 50 40 30 20 10
f1 (ppm)

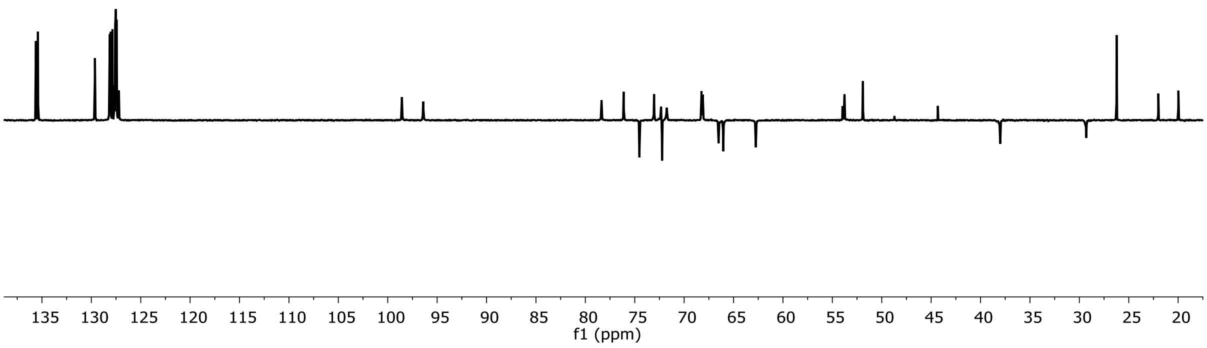
135 130 125 120 115 110 105 100 95 90 85 80 75 70 65 60 55 50 45 40 35 30 25 20
f1 (ppm)

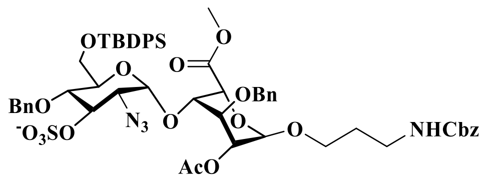
OTBDPS
O
O
BnO
$^{-}O_3SO$
N_3
O
OBn
O
O
NHCbz
AcO

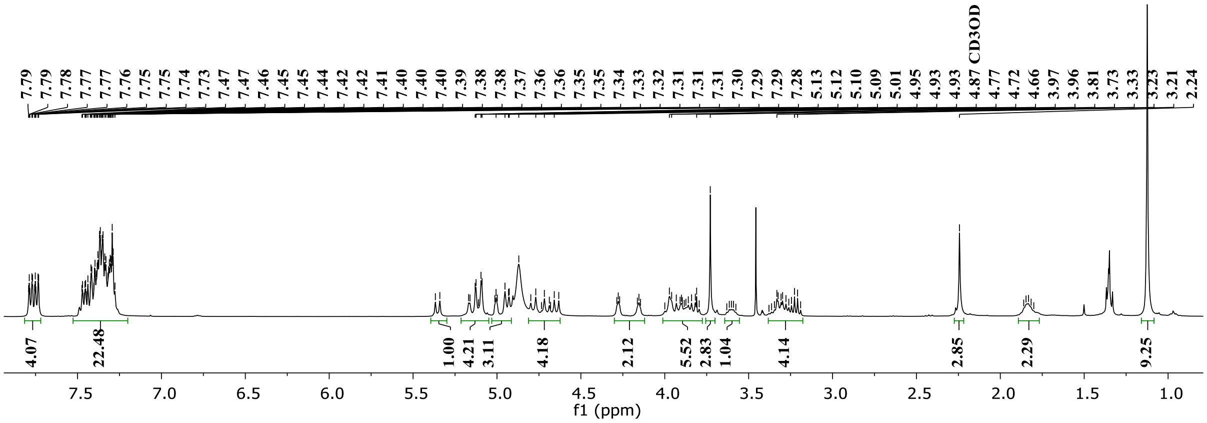
7.79 7.79 7.78 7.77 7.77 7.77 7.76 7.75 7.75 7.74 7.73 7.47 7.47 7.46 7.45 7.45 7.44 7.42 7.42 7.41 7.40 7.40 7.40 7.39 7.38 7.38 7.37 7.36 7.36 7.35 7.35 7.34 7.33 7.32 7.31 7.31 7.31 7.30 7.29 7.29 7.28 5.13 5.12 5.10 5.09 5.01 4.95 4.93 4.93 4.87 CD3OD 4.77 4.72 4.66 3.97 3.96 3.81 3.73 3.33 3.23 3.21 2.24
4.07
22.48
1.00
4.21
3.11
4.18
2.12
5.52
2.83
1.04
4.14
2.85
2.29
9.25
7.5 7.0 6.5 6.0 5.5 5.0 4.5 4.0 3.5 3.0 2.5 2.0 1.5 1.0
f1 (ppm)

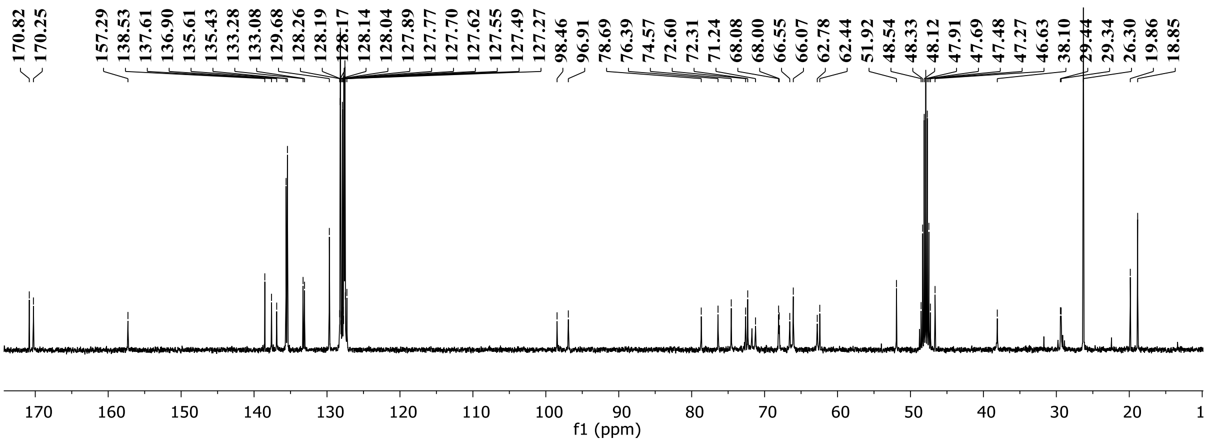
170.82 170.25
157.29 138.53 137.61 136.90 135.61 135.43 133.28 133.08 129.68 128.26 128.19 128.17 128.14 128.04 127.89 127.77 127.70 127.62 127.55 127.49 127.27
98.46 96.91
78.69 76.39 74.57 72.60 72.31 71.24 68.08 68.00 66.55 66.07 62.78 62.44
51.92 48.54 48.33 48.12 47.91 47.69 47.48 47.27 46.63 38.10 29.44 29.34 26.30 19.86 18.85
170 160 150 140 130 120 110 100 90 80 70 60 50 40 30 20 1
f1 (ppm)

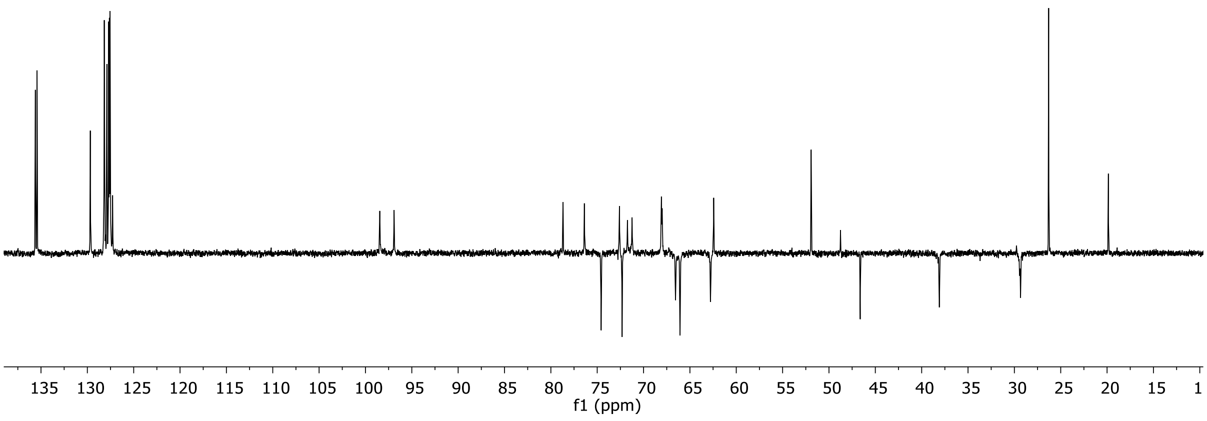
135 130 125 120 115 110 105 100 95 90 85 80 75 70 65 60 55 50 45 40 35 30 25 20 15 1
f1 (ppm)

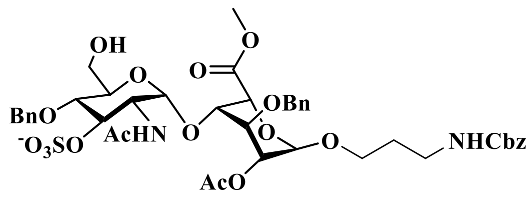

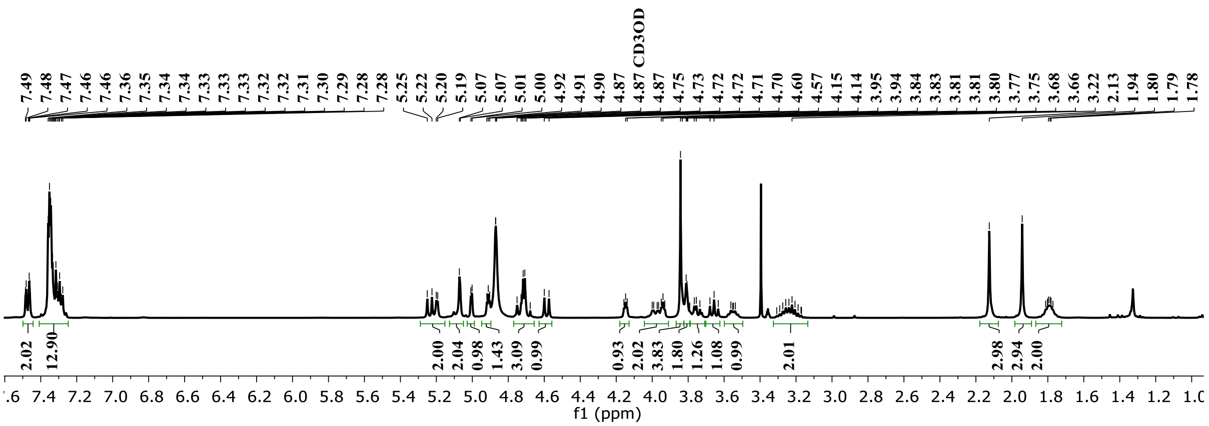

CD3OD
f1 (ppm)

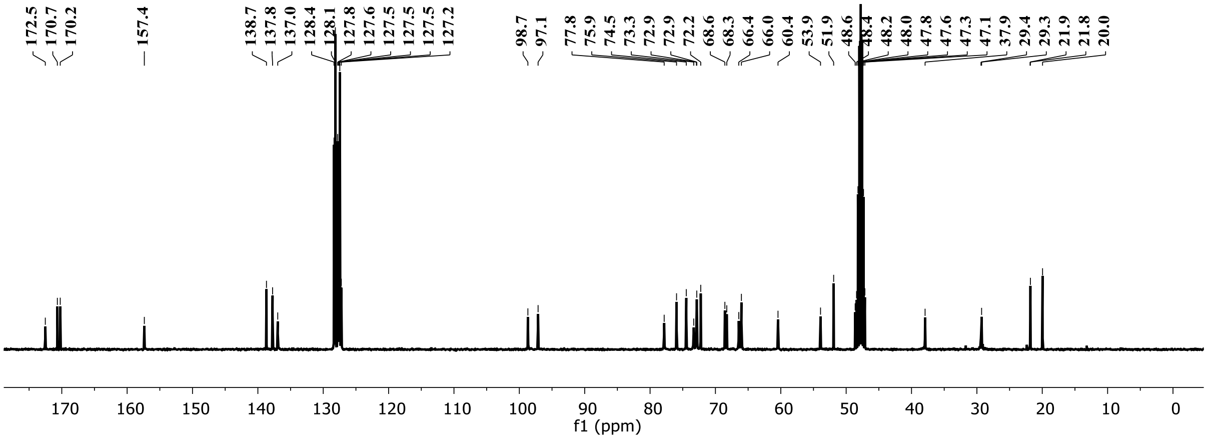

f1 (ppm)

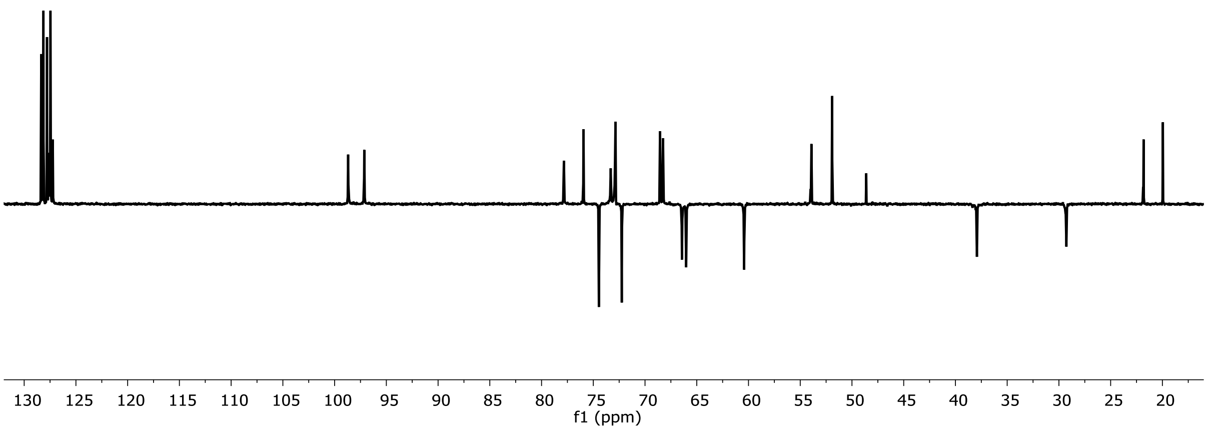

f1 (ppm)

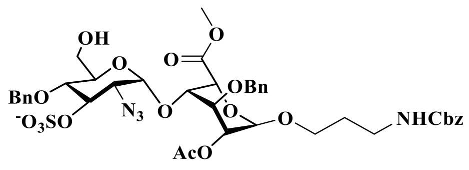
OH
BnO
$^{-}O_3SO$
N_3
O
O
OBn
NHCbz
AcO

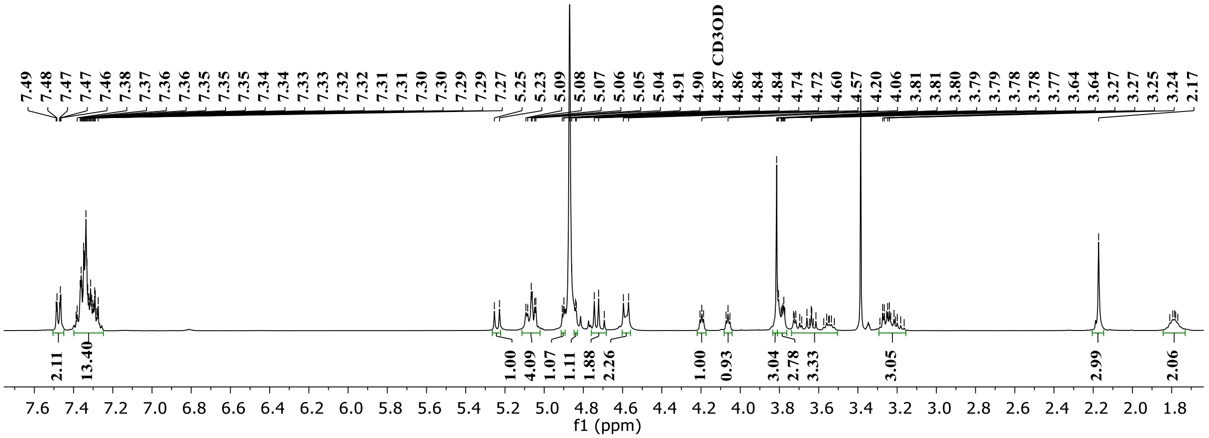
7.49 7.48 7.47 7.47 7.46 7.38 7.37 7.36 7.36 7.35 7.35 7.34 7.34 7.33 7.33 7.32 7.32 7.31 7.31 7.30 7.30 7.29 7.29 7.27 5.25 5.23 5.09 5.08 5.07 5.06 5.05 5.04 4.91 4.90 4.87 CD3OD 4.86 4.84 4.84 4.74 4.72 4.60 4.57 4.20 4.06 3.81 3.81 3.80 3.79 3.79 3.78 3.78 3.77 3.64 3.64 3.27 3.27 3.25 3.24 2.17
2.11 13.40 1.00 4.09 1.07 1.11 1.88 2.26 1.00 0.93 3.04 2.78 3.33 3.05 2.99 2.06
7.6 7.4 7.2 7.0 6.8 6.6 6.4 6.2 6.0 5.8 5.6 5.4 5.2 5.0 4.8 4.6 4.4 4.2 4.0 3.8 3.6 3.4 3.2 3.0 2.8 2.6 2.4 2.2 2.0 1.8
f1 (ppm)

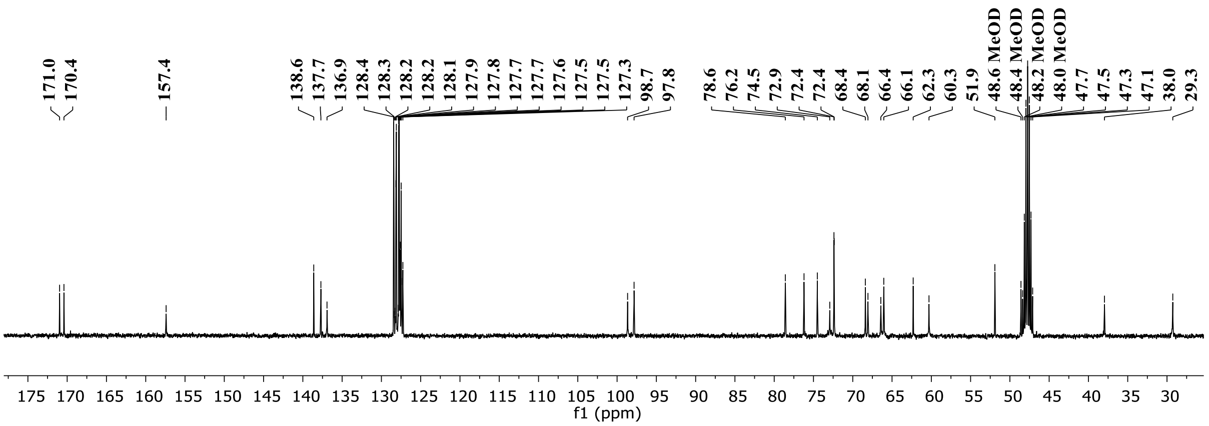
171.0 170.4 157.4 138.6 137.7 136.9 128.4 128.3 128.2 128.2 128.1 127.9 127.8 127.7 127.7 127.6 127.5 127.5 127.3 98.7 97.8 78.6 76.2 74.5 72.9 72.4 72.4 68.4 68.1 66.4 66.1 62.3 60.3 51.9 48.6 MeOD 48.4 MeOD 48.2 MeOD 48.0 MeOD 47.7 47.5 47.3 47.1 38.0 29.3
175 170 165 160 155 150 145 140 135 130 125 120 115 110 105 100 95 90 85 80 75 70 65 60 55 50 45 40 35 30
f1 (ppm)

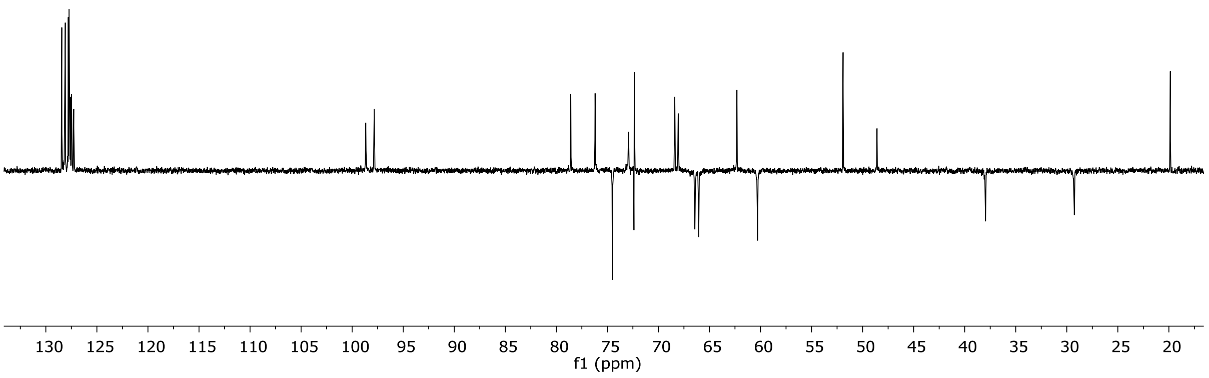
130 125 120 115 110 105 100 95 90 85 80 75 70 65 60 55 50 45 40 35 30 25 20
f1 (ppm)

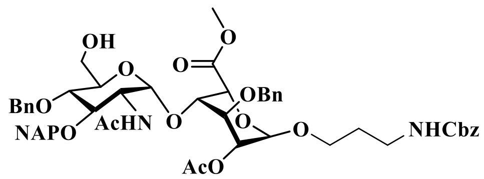
OH
O
BnO
NAPO
AcHN
O
OBn
NHCbz
AcO

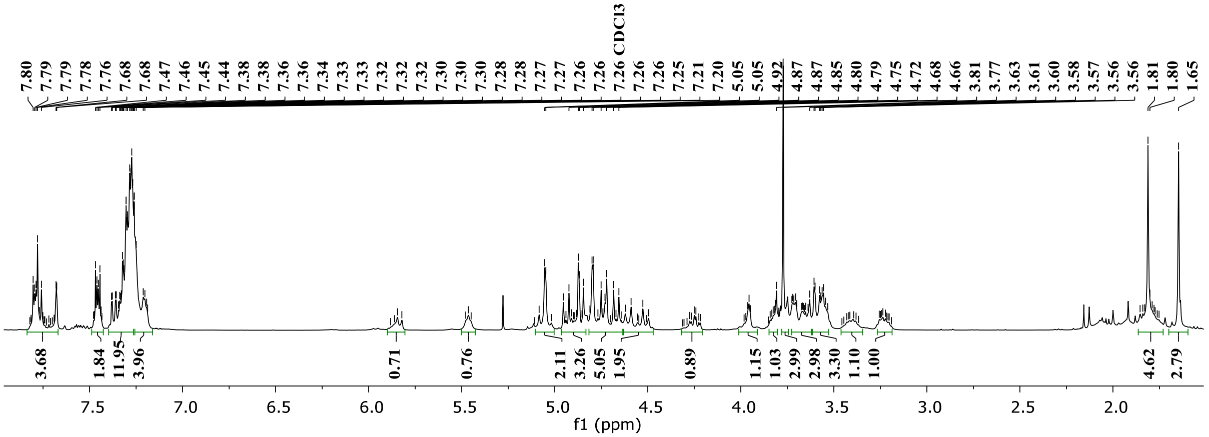
CDCl3
7.80 7.79 7.79 7.78 7.76 7.68 7.68 7.47 7.46 7.45 7.44 7.38 7.38 7.36 7.36 7.34 7.33 7.33 7.32 7.32 7.32 7.30 7.30 7.28 7.28 7.27 7.27 7.26 7.26 7.26 7.26 7.26 7.25 7.21 7.20 5.05 5.05 4.92 4.87 4.87 4.85 4.80 4.79 4.75 4.72 4.68 4.66 3.81 3.77 3.63 3.61 3.60 3.58 3.57 3.56 3.56 1.81 1.80 1.65
3.68 1.84 11.95 3.96 0.71 0.76 2.11 3.26 5.05 1.95 0.89 1.15 1.03 2.99 2.98 3.30 1.10 1.00 4.62 2.79
7.5 7.0 6.5 6.0 5.5 5.0 4.5 4.0 3.5 3.0 2.5 2.0
f1 (ppm)

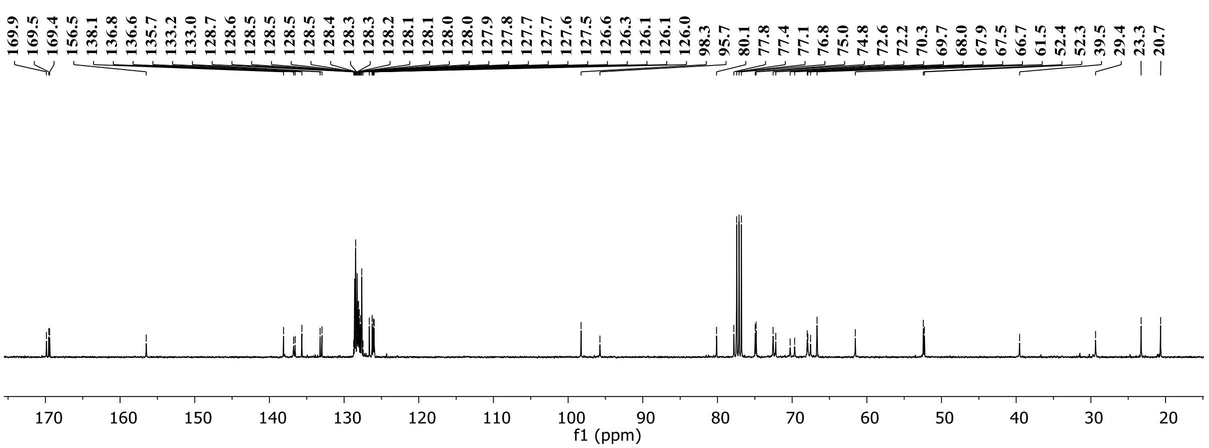
169.9 169.5 169.4 156.5 138.1 136.8 136.6 135.7 133.2 133.0 128.7 128.6 128.5 128.5 128.5 128.5 128.4 128.3 128.3 128.2 128.1 128.1 128.0 128.0 127.9 127.8 127.7 127.7 127.6 127.5 126.6 126.3 126.1 126.1 126.0 98.3 95.7 80.1 77.8 77.4 77.1 76.8 75.0 74.8 72.6 72.2 70.3 69.7 68.0 67.9 67.5 66.7 61.5 52.4 52.3 39.5 29.4 23.3 20.7
170 160 150 140 130 120 110 100 90 80 70 60 50 40 30 20
f1 (ppm)

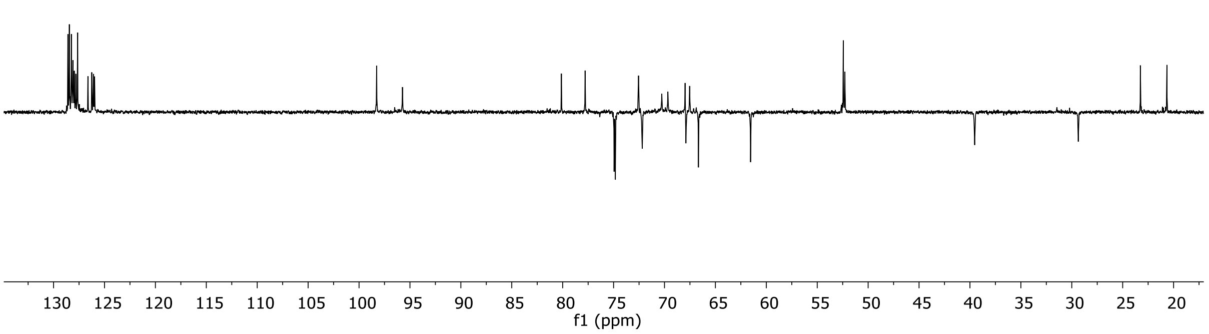
130 125 120 115 110 105 100 95 90 85 80 75 70 65 60 55 50 45 40 35 30 25 20
f1 (ppm)

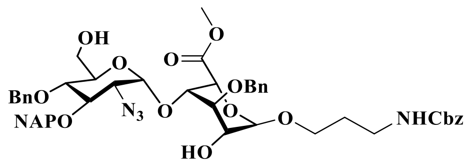

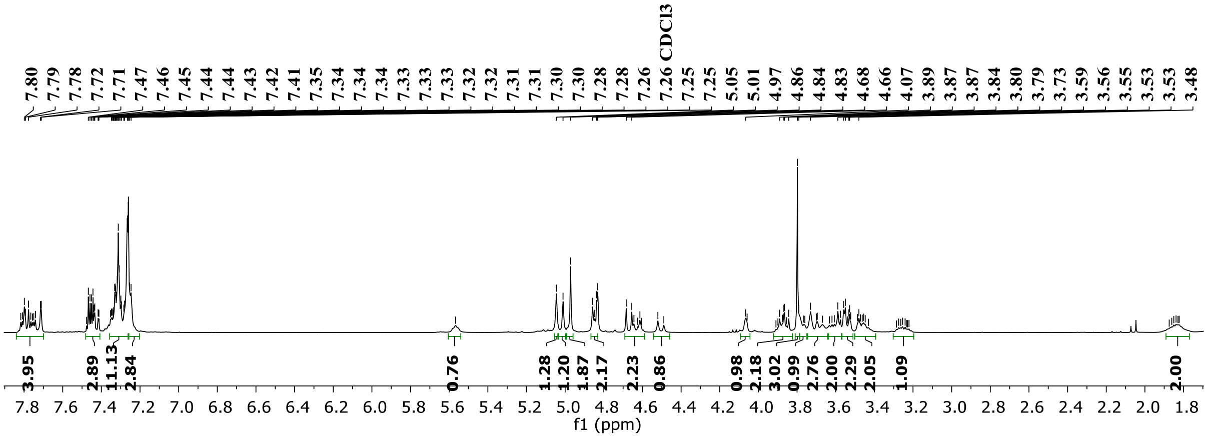
7.80 7.79 7.78 7.72 7.71 7.47 7.46 7.45 7.44 7.44 7.43 7.42 7.41 7.35 7.34 7.34 7.34 7.33 7.33 7.33 7.32 7.32 7.31 7.31 7.30 7.30 7.28 7.26 7.26 CDCl3 7.25 7.25 5.05 5.01 4.97 4.86 4.84 4.83 4.68 4.66 4.07 3.89 3.87 3.84 3.80 3.79 3.73 3.59 3.56 3.55 3.53 3.53 3.48
3.95 2.89 11.13 2.84 0.76 1.28 1.20 1.87 2.17 2.23 0.86 0.98 2.18 3.02 0.99 2.76 2.00 2.29 2.05 1.09 2.00
7.8 7.6 7.4 7.2 7.0 6.8 6.6 6.4 6.2 6.0 5.8 5.6 5.4 5.2 5.0 4.8 4.6 4.4 4.2 4.0 3.8 3.6 3.4 3.2 3.0 2.8 2.6 2.4 2.2 2.0 1.8
f1 (ppm)

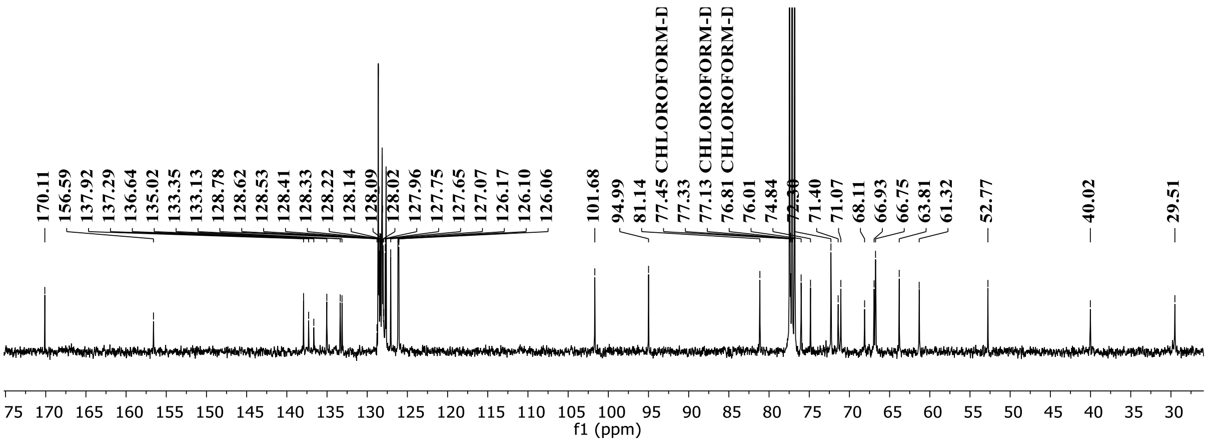
170.11 156.59 137.92 137.29 136.64 135.02 133.35 133.13 128.78 128.62 128.53 128.41 128.33 128.22 128.14 128.09 128.02 127.96 127.75 127.65 127.07 126.17 126.10 126.06 101.68 94.99 81.14 77.45 CHLOROFORM-D 77.33 77.13 CHLOROFORM-D 76.81 CHLOROFORM-D 76.01 74.84 72.30 71.40 71.07 68.11 66.93 66.75 63.81 61.32 52.77 40.02 29.51
75 170 165 160 155 150 145 140 135 130 125 120 115 110 105 100 95 90 85 80 75 70 65 60 55 50 45 40 35 30
f1 (ppm)

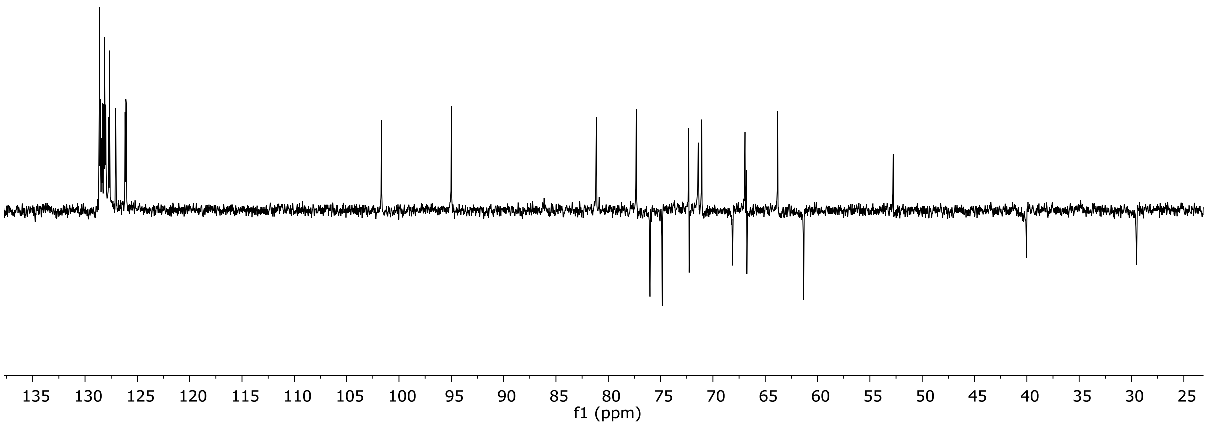
135 130 125 120 115 110 105 100 95 90 85 80 75 70 65 60 55 50 45 40 35 30 25
f1 (ppm)

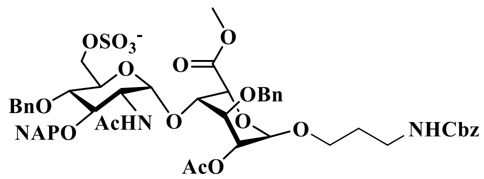
OSO3-
O
O
BnO
NAPO
AcHN
O
OBn
O
O
NHCbz
AcO

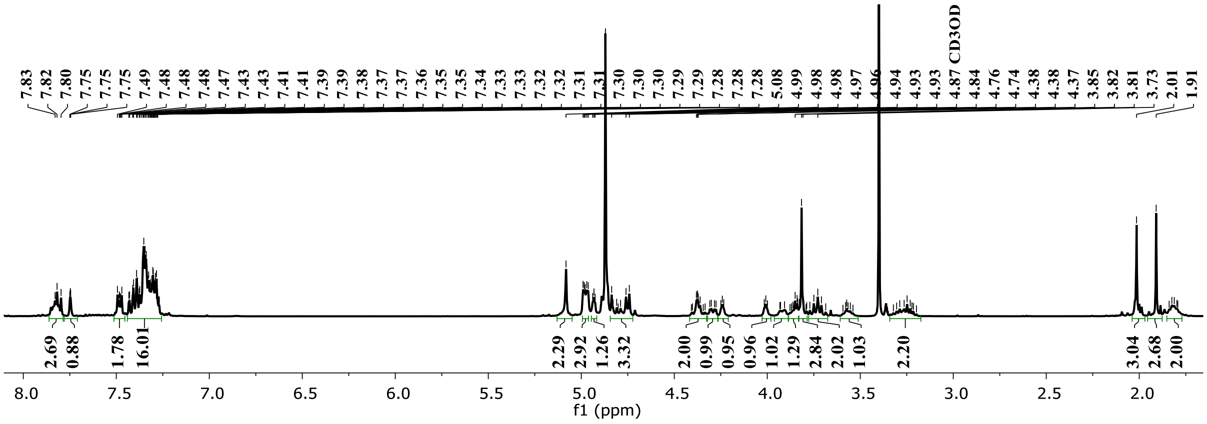
CD3OD
f1 (ppm)

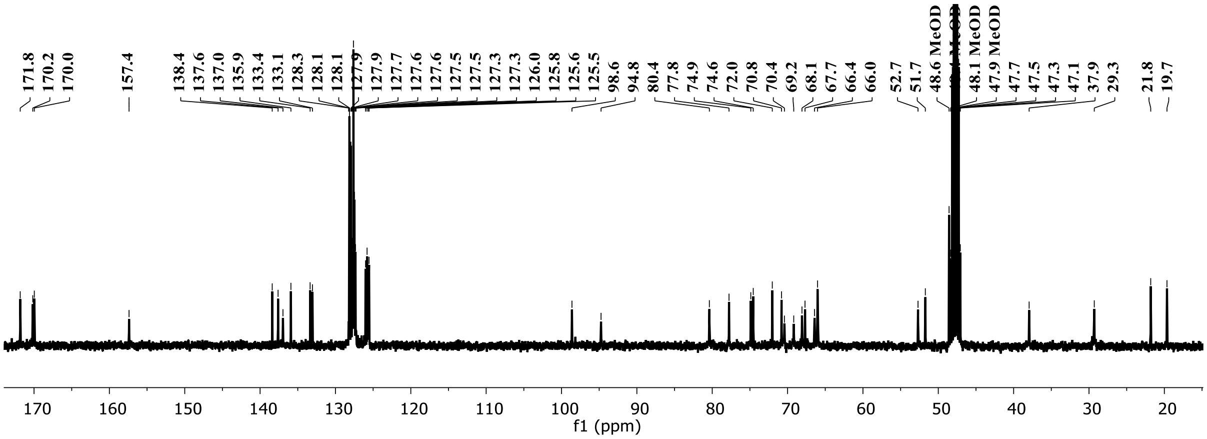
MeOD
f1 (ppm)

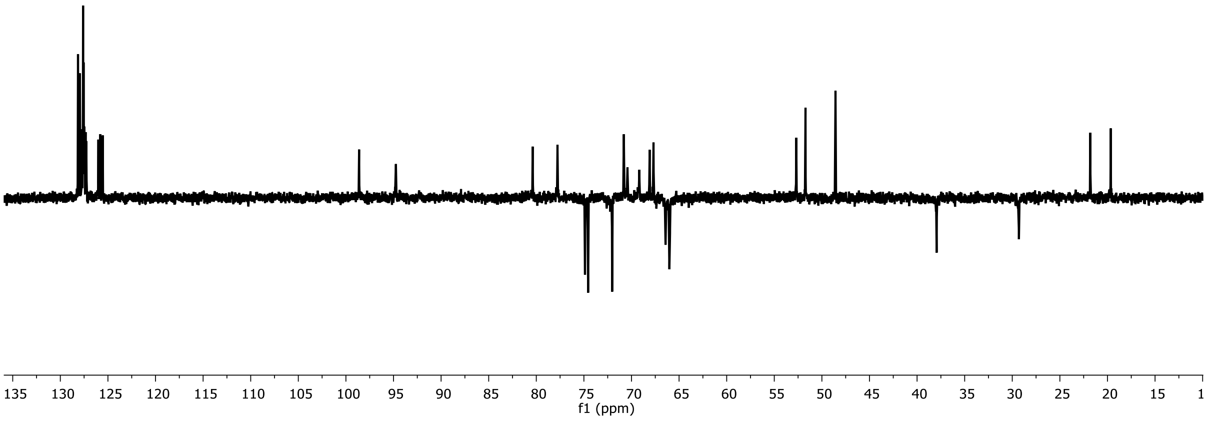
f1 (ppm)

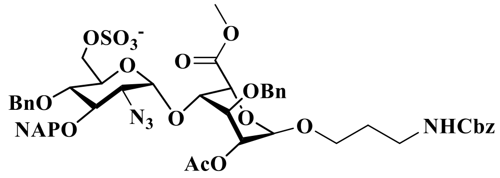
OSO3-
O
BnO
NAPO
N3
O
O
OBn
O
O
NHCbz
AcO

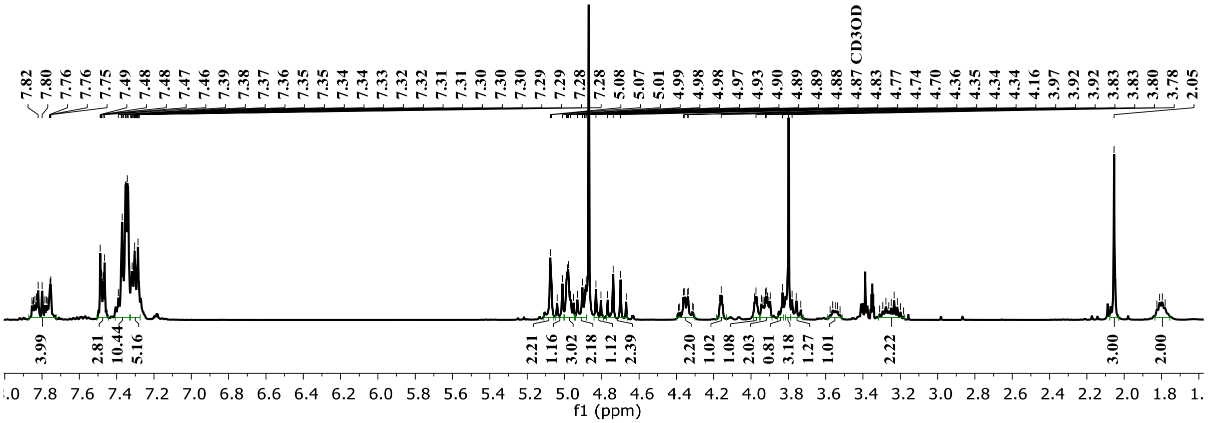
7.82 7.80 7.76 7.76 7.75 7.49 7.48 7.48 7.47 7.46 7.39 7.38 7.37 7.36 7.35 7.35 7.34 7.34 7.33 7.32 7.32 7.31 7.31 7.30 7.30 7.29 7.29 7.28 7.28 5.08 5.07 5.01 4.99 4.98 4.98 4.97 4.93 4.90 4.89 4.89 4.88 4.87 CD3OD 4.83 4.77 4.74 4.70 4.36 4.35 4.34 4.34 4.16 3.97 3.92 3.92 3.83 3.83 3.80 3.78 2.05
3.99 2.81 10.44 5.16 2.21 1.16 3.02 2.18 1.12 2.39 2.20 1.02 1.08 2.03 0.81 3.18 1.27 1.01 2.22 3.00 2.00
f1 (ppm)

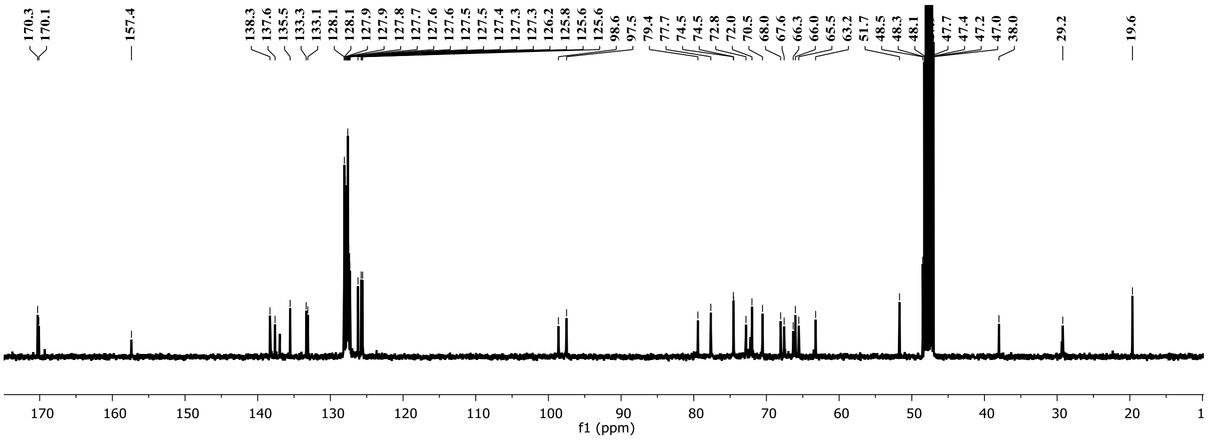
170.3 170.1 157.4 138.3 137.6 135.5 133.3 133.1 128.1 128.1 127.9 127.9 127.8 127.7 127.6 127.6 127.5 127.5 127.4 127.3 127.3 126.2 125.8 125.6 125.6 98.6 97.5 79.4 77.7 74.5 74.5 72.8 72.0 70.5 68.0 67.6 66.3 66.0 65.5 63.2 51.7 48.5 48.3 48.1 47.7 47.4 47.2 47.0 38.0 29.2 19.6
f1 (ppm)

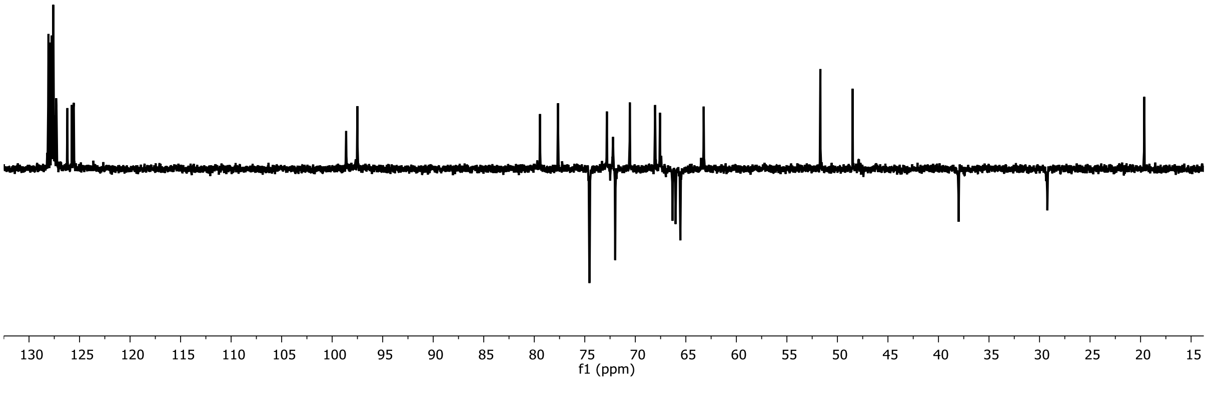
f1 (ppm)

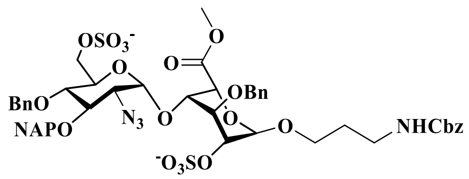
OSO3-
BnO
NAPO
N3
O
OBn
NHCbz
-O3SO

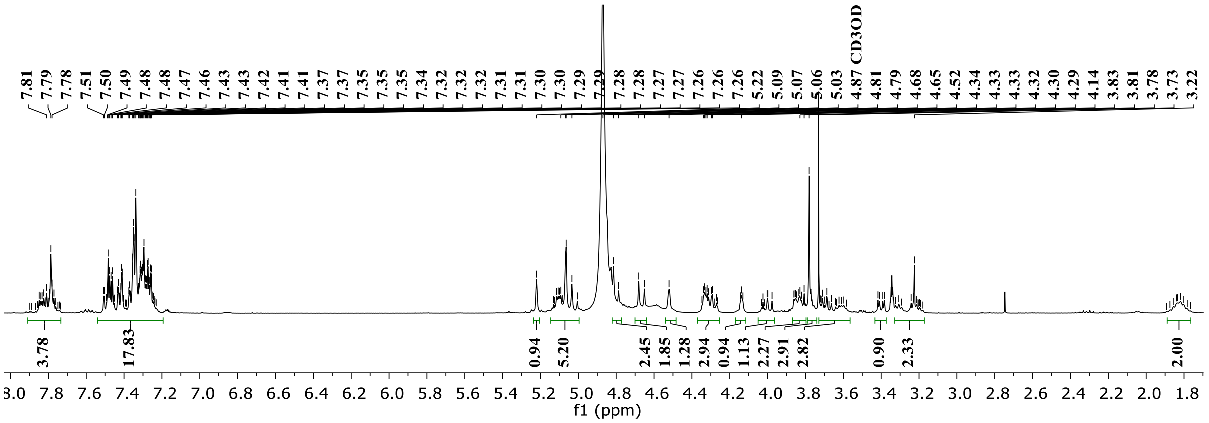
CD3OD
f1 (ppm)

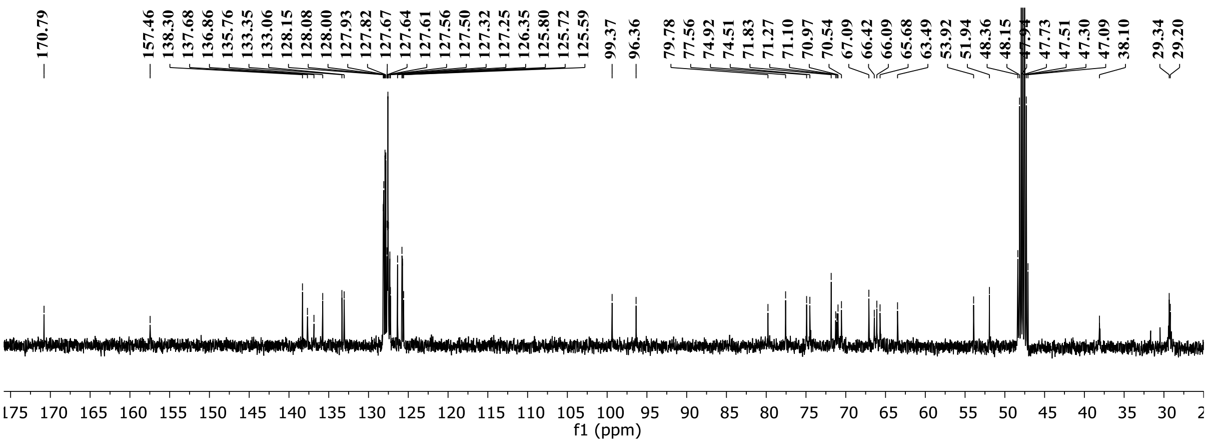
f1 (ppm)

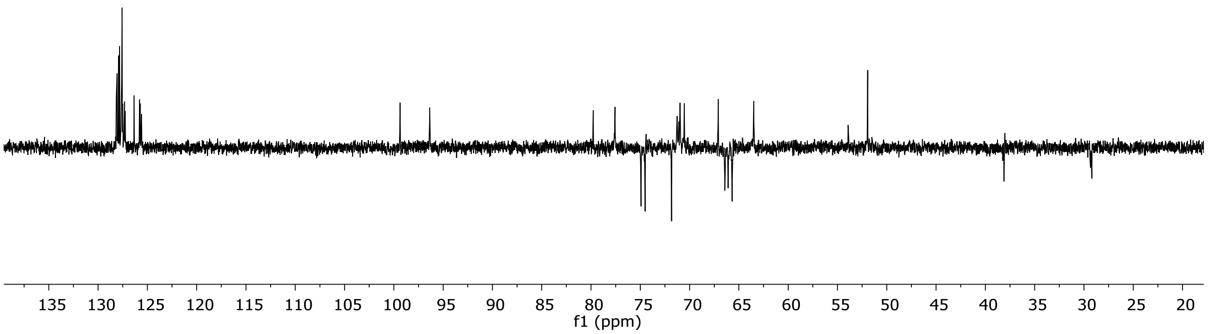
f1 (ppm)

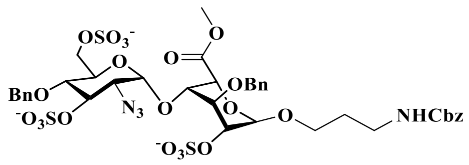
OSO3-
BnO
-O3SO
N3
O
OBn
O
NHCbz
-O3SO

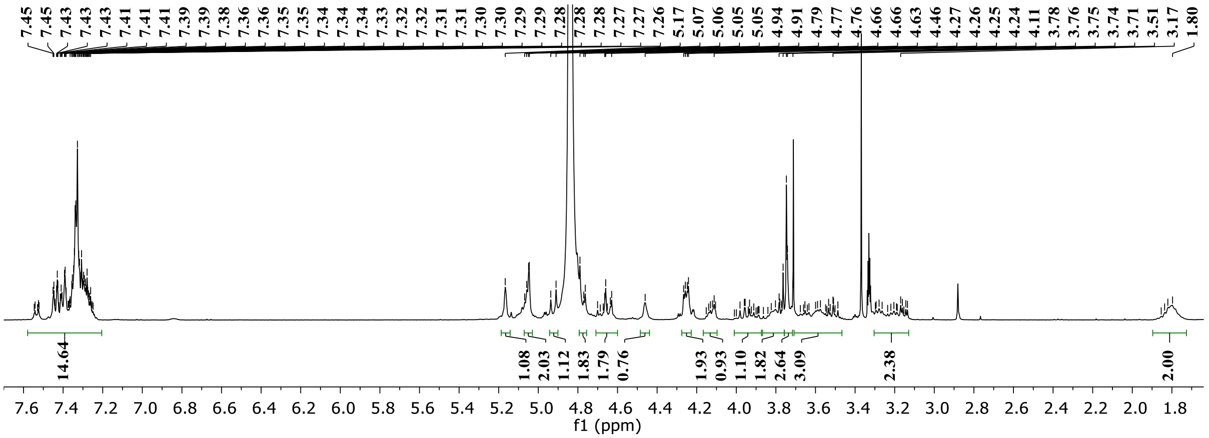
7.45 7.45 7.43 7.43 7.43 7.41 7.41 7.41 7.39 7.39 7.38 7.36 7.36 7.35 7.35 7.34 7.34 7.34 7.33 7.32 7.32 7.31 7.31 7.30 7.30 7.29 7.29 7.28 7.28 7.28 7.27 7.27 7.26 5.17 5.07 5.06 5.05 5.05 4.94 4.91 4.79 4.77 4.76 4.66 4.66 4.63 4.46 4.27 4.26 4.25 4.24 4.11 3.78 3.76 3.75 3.74 3.71 3.51 3.17 1.80
14.64
1.08 2.03 1.12 1.83 1.79 0.76
1.93 0.93 1.10 1.82 2.64 3.09
2.38
2.00
7.6 7.4 7.2 7.0 6.8 6.6 6.4 6.2 6.0 5.8 5.6 5.4 5.2 5.0 4.8 4.6 4.4 4.2 4.0 3.8 3.6 3.4 3.2 3.0 2.8 2.6 2.4 2.2 2.0 1.8
f1 (ppm)

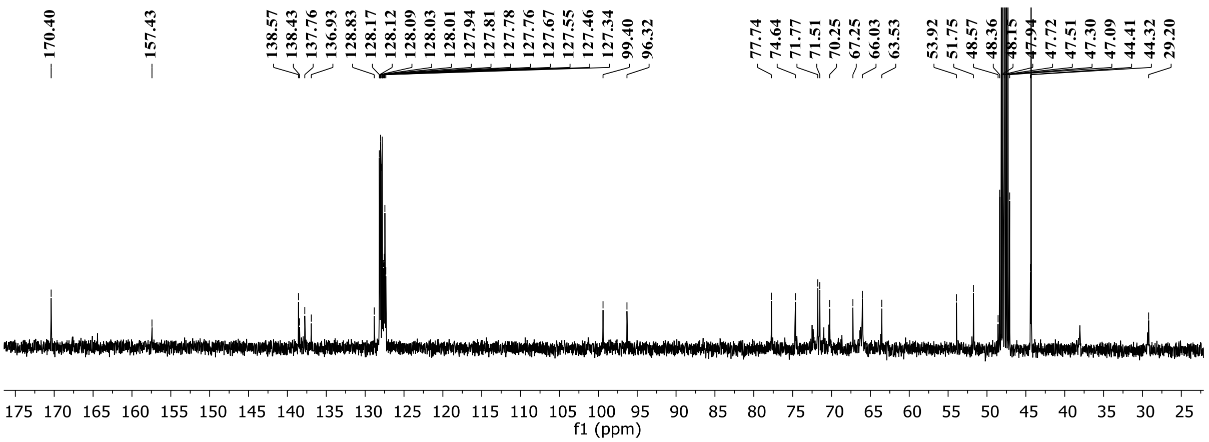
170.40
157.43
138.57 138.43 137.76 136.93 128.83 128.17 128.12 128.09 128.03 128.01 127.94 127.81 127.78 127.76 127.67 127.55 127.46 127.34 99.40 96.32
77.74 74.64 71.77 71.51 70.25 67.25 66.03 63.53
53.92 51.75 48.57 48.36 48.15 47.94 47.72 47.51 47.30 47.09 44.41 44.32 29.20
175 170 165 160 155 150 145 140 135 130 125 120 115 110 105 100 95 90 85 80 75 70 65 60 55 50 45 40 35 30 25
f1 (ppm)

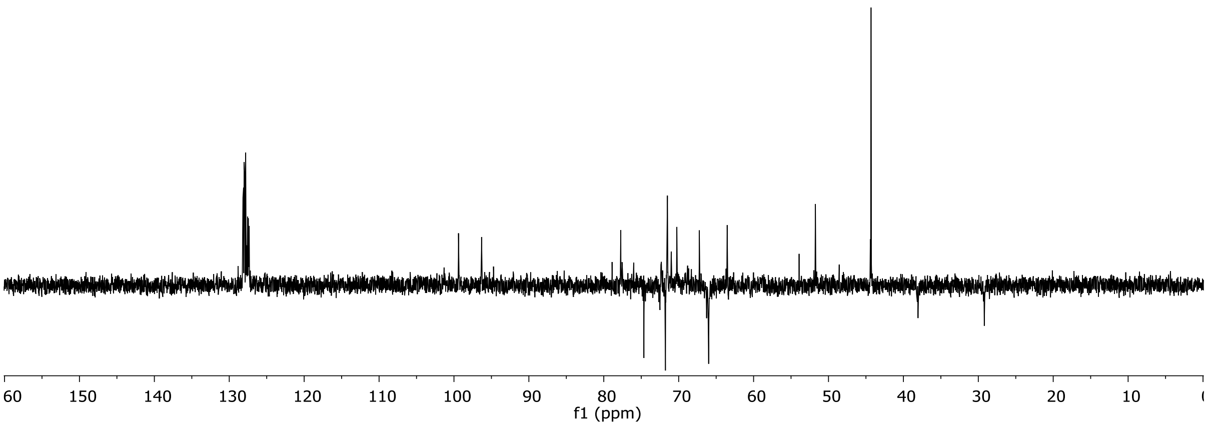
60 150 140 130 120 110 100 90 80 70 60 50 40 30 20 10
f1 (ppm)

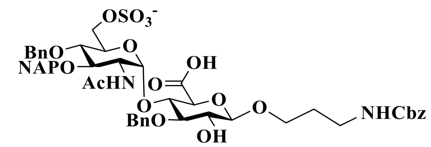
OSO3-
BnO
NAPO
AcHN
O
OH
O
BnO
OH
NHCbz

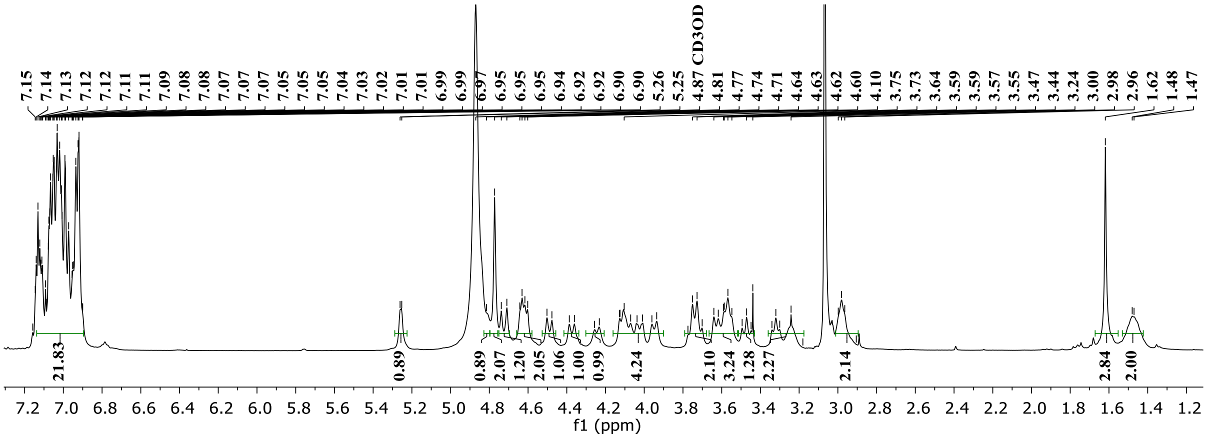
7.15 7.14 7.13 7.12 7.12 7.11 7.11 7.09 7.08 7.07 7.07 7.07 7.05 7.05 7.05 7.04 7.03 7.02 7.01 7.01 6.99 6.99 6.97 6.95 6.95 6.95 6.94 6.92 6.92 6.90 6.90 5.26 5.25 4.87 4.81 4.77 4.74 4.71 4.64 4.63 4.62 4.60 4.10 3.75 3.73 3.64 3.59 3.59 3.57 3.55 3.47 3.44 3.24 3.00 2.98 2.96 1.62 1.48 1.47
CD3OD
21.83 0.89 0.89 2.07 1.20 2.05 1.06 1.00 0.99 4.24 2.10 3.24 1.28 2.27 2.14 2.84 2.00
7.2 7.0 6.8 6.6 6.4 6.2 6.0 5.8 5.6 5.4 5.2 5.0 4.8 4.6 4.4 4.2 4.0 3.8 3.6 3.4 3.2 3.0 2.8 2.6 2.4 2.2 2.0 1.8 1.6 1.4 1.2
f1 (ppm)

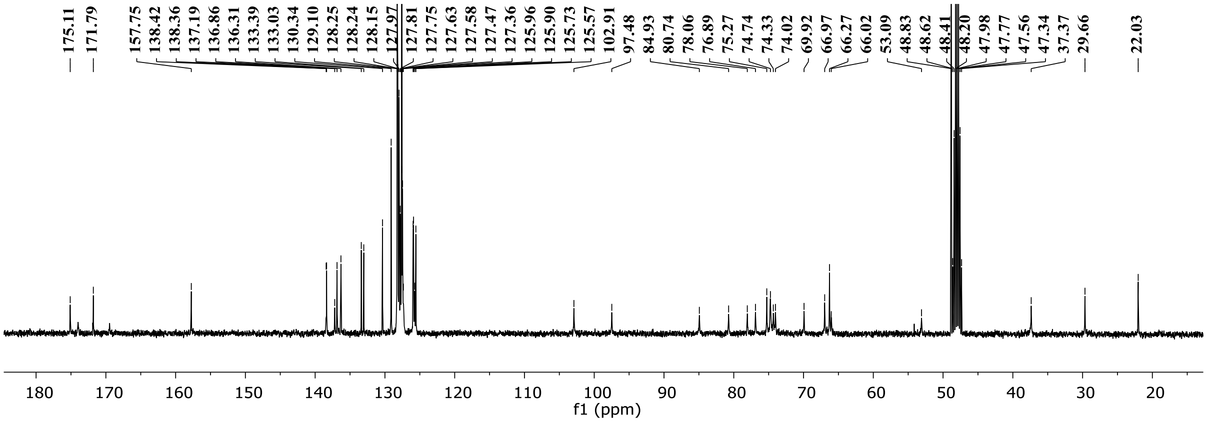
175.11 171.79 157.75 138.42 138.36 137.19 136.86 136.31 133.39 133.03 130.34 129.10 128.25 128.24 128.15 127.97 127.81 127.75 127.63 127.58 127.47 127.36 125.96 125.90 125.73 125.57 102.91 97.48 84.93 80.74 78.06 76.89 75.27 74.74 74.33 74.02 69.92 66.97 66.27 66.02 53.09 48.83 48.62 48.41 48.20 47.98 47.77 47.56 47.34 37.37 29.66 22.03
180 170 160 150 140 130 120 110 100 90 80 70 60 50 40 30 20
f1 (ppm)

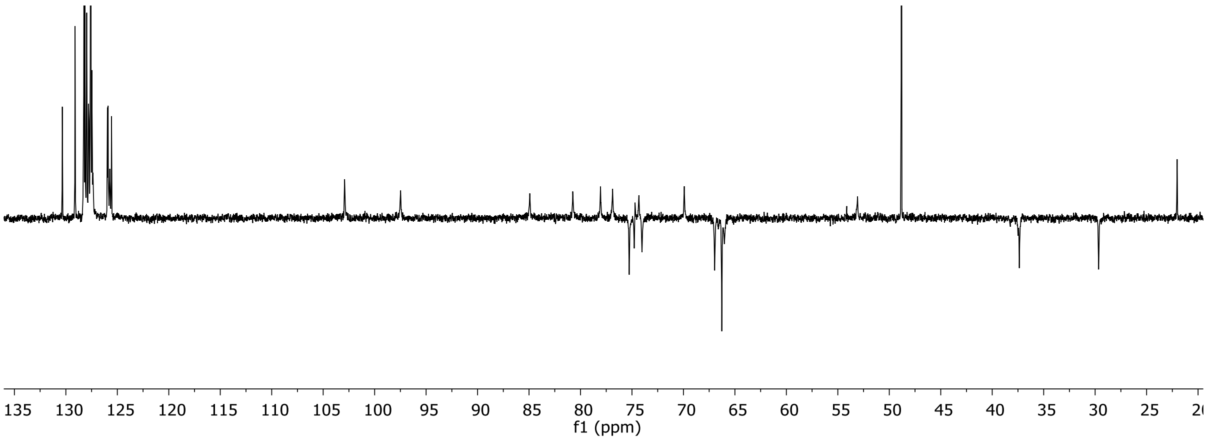
135 130 125 120 115 110 105 100 95 90 85 80 75 70 65 60 55 50 45 40 35 30 25
f1 (ppm)

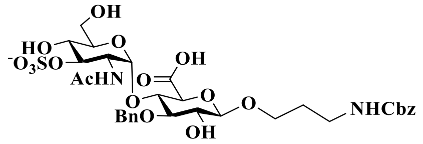
OH
HO
$^{-}O_3SO$
AcHN
O
OH
O
BnO
OH
O
NHCbz

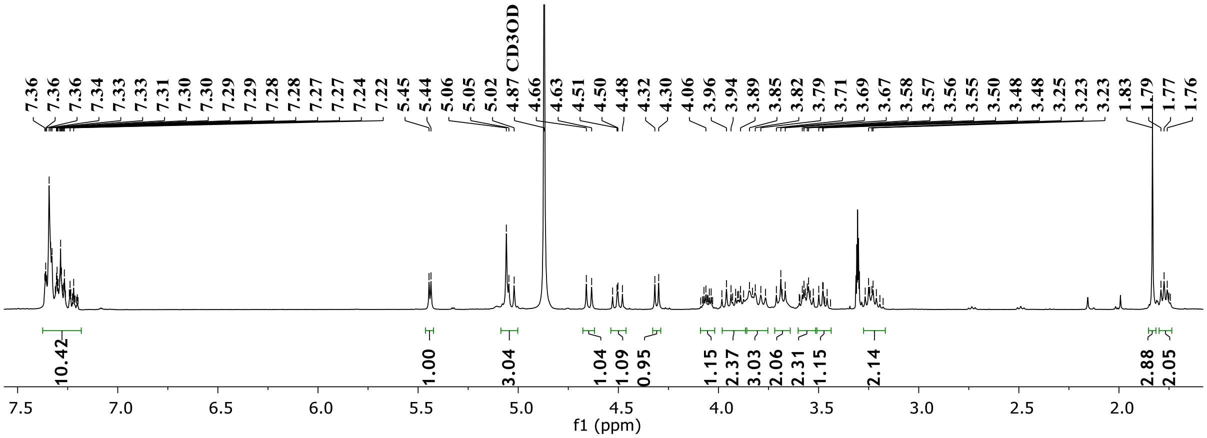
7.36 7.36 7.36 7.34 7.33 7.33 7.31 7.30 7.30 7.29 7.29 7.28 7.28 7.27 7.27 7.24 7.22 5.45 5.44 5.06 5.05 5.02 4.87 4.66 4.63 4.51 4.50 4.48 4.32 4.30 4.06 3.96 3.94 3.89 3.85 3.82 3.79 3.71 3.69 3.67 3.58 3.57 3.56 3.55 3.50 3.48 3.48 3.25 3.23 3.23 1.83 1.79 1.77 1.76
CD3OD
10.42 1.00 3.04 1.04 1.09 0.95 1.15 2.37 3.03 2.06 2.31 1.15 2.14 2.88 2.05
f1 (ppm)

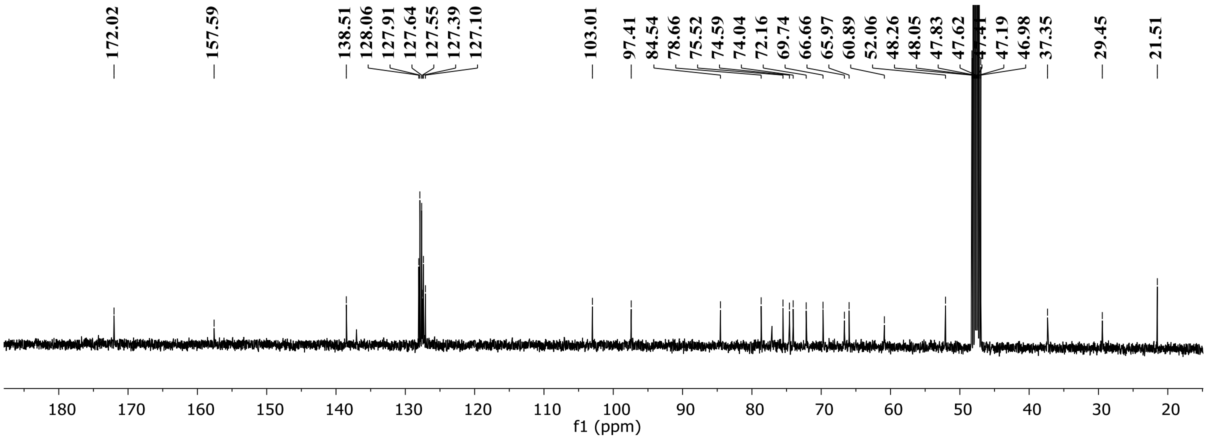
172.02 157.59 138.51 128.06 127.91 127.64 127.55 127.39 127.10 103.01 97.41 84.54 78.66 75.52 74.59 74.04 72.16 69.74 66.66 65.97 60.89 52.06 48.26 48.05 47.83 47.62 47.41 47.19 46.98 37.35 29.45 21.51
f1 (ppm)

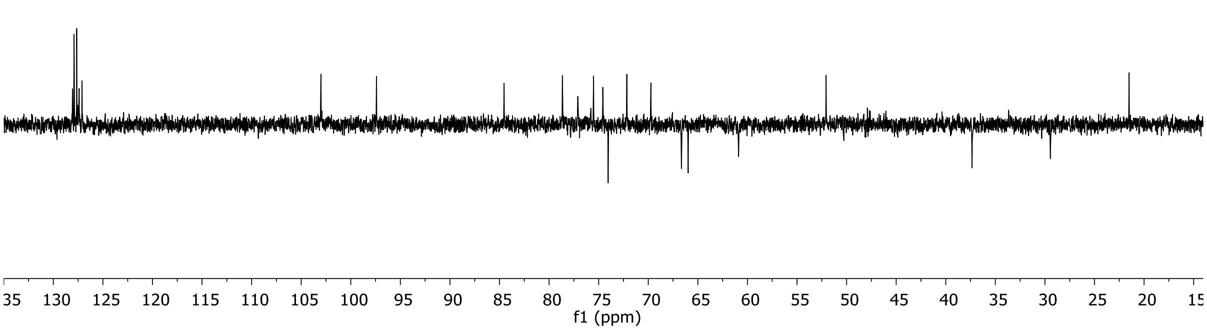
f1 (ppm)

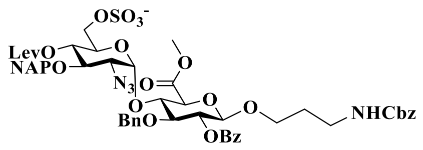
OSO3-
LevO
NAPO
N3
O
BnO
OBz
NHCbz

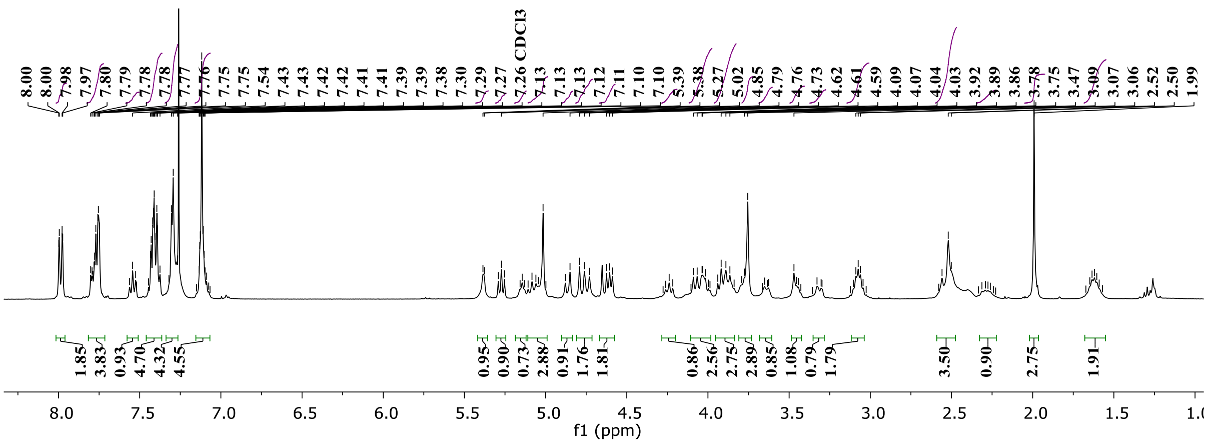
8.00 8.00 7.98 7.97 7.80 7.79 7.78 7.78 7.77 7.76 7.75 7.75 7.54 7.43 7.43 7.42 7.42 7.41 7.41 7.39 7.39 7.38 7.30 7.29 7.27 7.26 CDCl3 7.13 7.13 7.13 7.12 7.11 7.10 7.10 5.39 5.38 5.27 5.02 4.85 4.79 4.76 4.73 4.62 4.61 4.59 4.09 4.07 4.04 4.03 3.92 3.89 3.86 3.78 3.75 3.47 3.09 3.07 3.06 2.52 2.50 1.99
1.85 3.83 0.93 4.70 4.32 4.55 0.95 0.90 0.73 2.88 0.91 1.76 1.81 0.86 2.56 2.75 2.89 0.85 1.08 0.79 1.79 3.50 0.90 2.75 1.91
f1 (ppm)

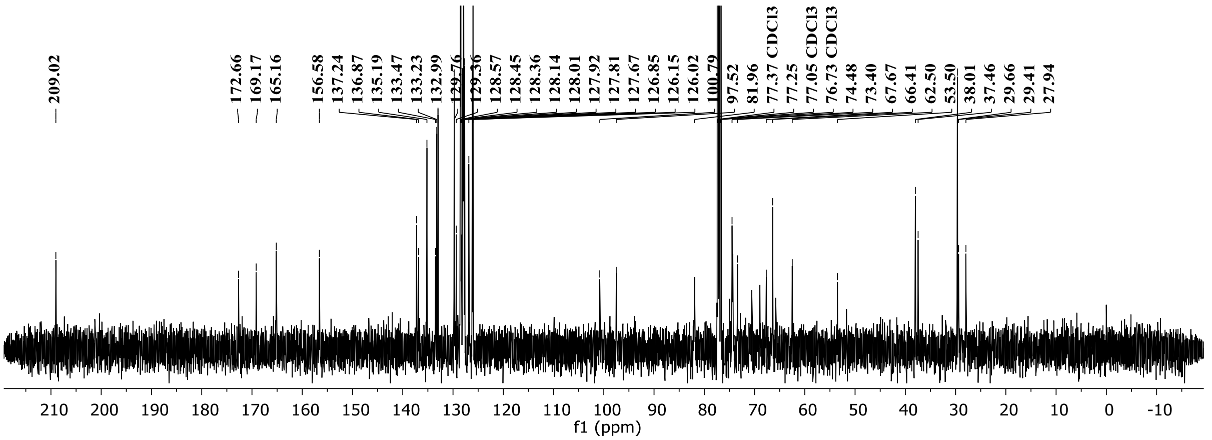
209.02 172.66 169.17 165.16 156.58 137.24 136.87 135.19 133.47 133.23 132.99 129.76 129.36 128.57 128.45 128.36 128.14 128.01 127.92 127.81 127.67 126.85 126.15 126.02 100.79 97.52 81.96 77.37 CDCl3 77.25 77.05 CDCl3 76.73 CDCl3 74.48 73.40 67.67 66.41 62.50 53.50 38.01 37.46 29.66 29.41 27.94
f1 (ppm)

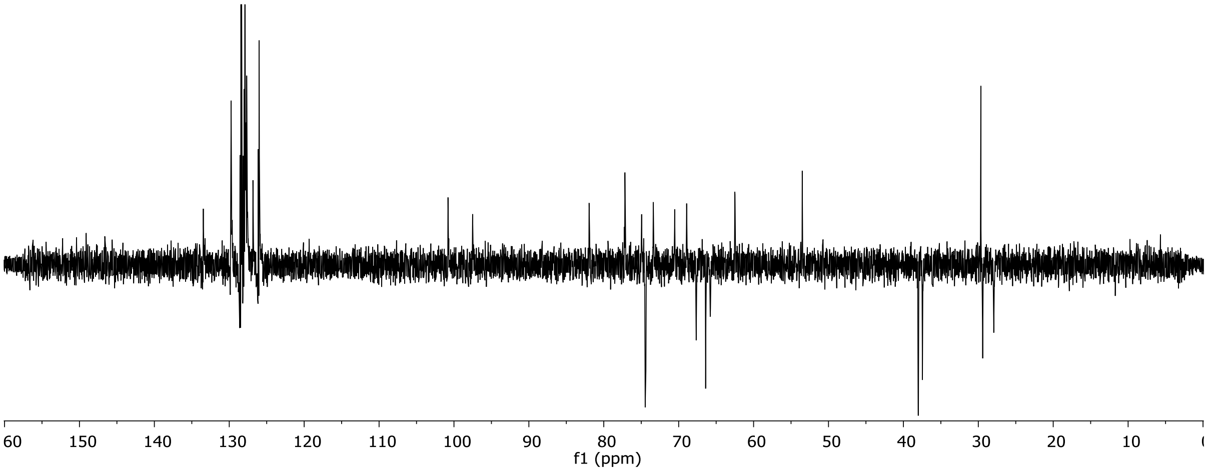
f1 (ppm)

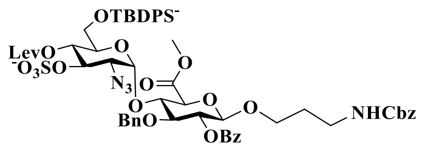
OTBDPS
LevO
^-O_3SO
N_3
O
BnO
OBz
NHCbz

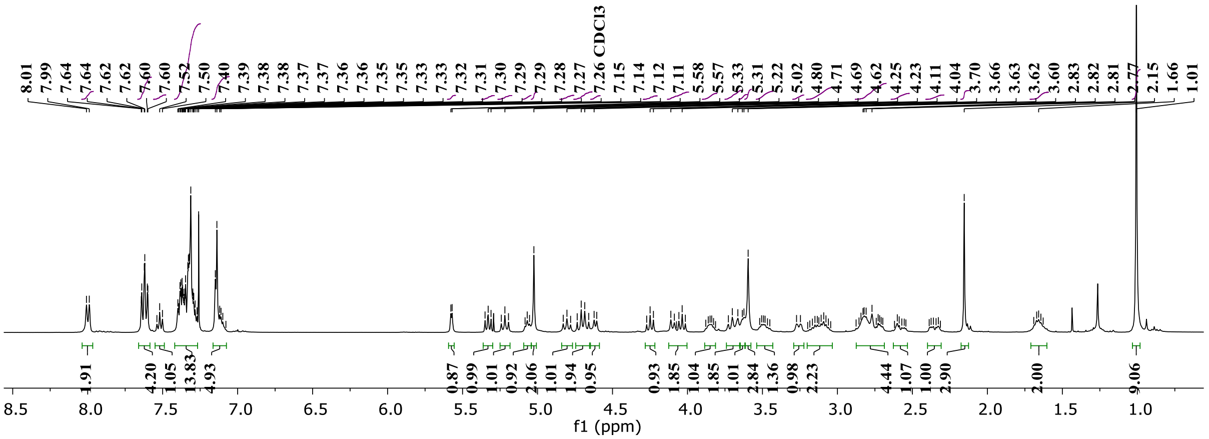
8.01 7.99 7.64 7.64 7.62 7.62 7.60 7.60 7.52 7.50 7.40 7.39 7.38 7.38 7.37 7.37 7.36 7.36 7.35 7.35 7.33 7.33 7.32 7.31 7.30 7.29 7.29 7.28 7.27 7.26 CDCl3 7.15 7.14 7.12 7.11 5.58 5.57 5.33 5.31 5.22 5.02 4.80 4.71 4.69 4.62 4.25 4.23 4.11 4.04 3.70 3.66 3.63 3.62 3.60 2.83 2.82 2.81 2.77 2.15 1.66 1.01
1.91 4.20 1.05 13.83 4.93 0.87 0.99 1.01 0.92 2.06 1.01 1.94 0.95 0.93 1.85 1.04 1.85 1.01 2.84 1.36 0.98 2.23 4.44 1.07 1.00 2.90 2.00 9.06
f1 (ppm)

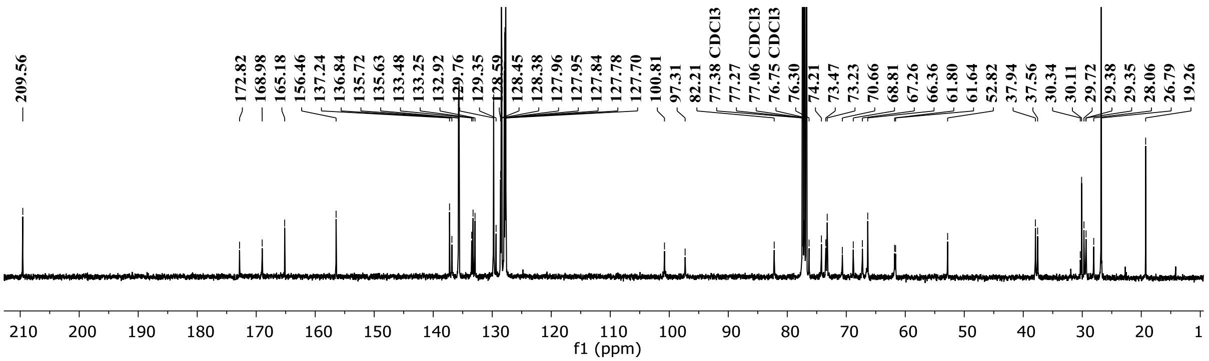
209.56 172.82 168.98 165.18 156.46 137.24 136.84 135.72 135.63 133.48 133.25 132.92 129.76 129.35 128.59 128.45 128.38 127.96 127.95 127.84 127.78 127.70 100.81 97.31 82.21 77.38 CDCl3 77.27 77.06 CDCl3 76.75 CDCl3 76.30 74.21 73.47 73.23 70.66 68.81 67.26 66.36 61.80 61.64 52.82 37.94 37.56 30.34 30.11 29.72 29.38 29.35 28.06 26.79 19.26
f1 (ppm)

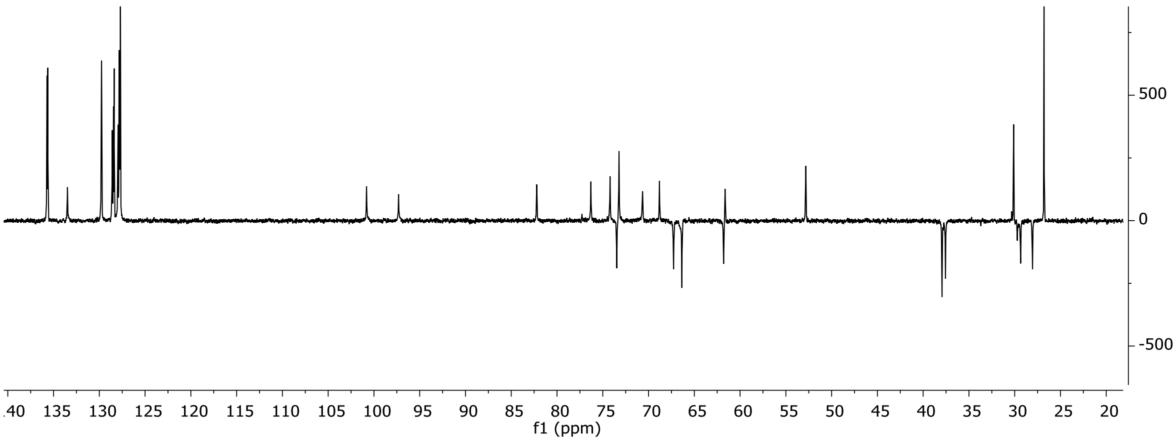
500
0
-500
f1 (ppm)

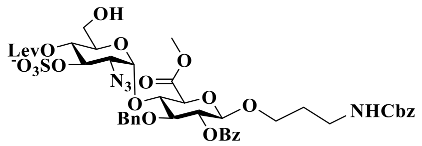

OH
LevO
-O3SO
N3
O
O
O
BnO
OBz
O
NHCbz

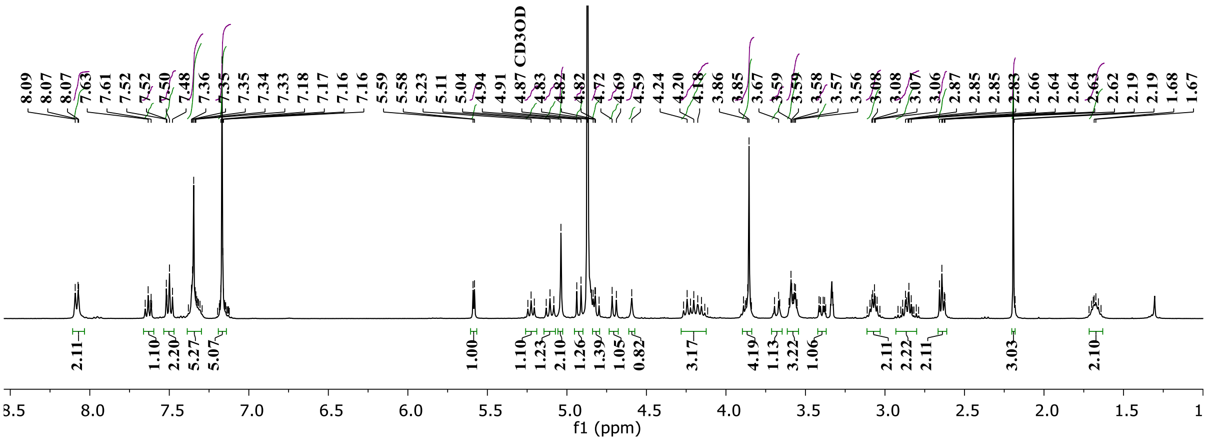

8.09
8.07
8.07
7.63
7.61
7.52
7.52
7.50
7.48
7.36
7.35
7.35
7.34
7.33
7.18
7.17
7.16
7.16
5.59
5.58
5.23
5.11
5.04
4.94
4.91
4.87 CD3OD
4.83
4.82
4.82
4.72
4.69
4.59
4.24
4.20
4.18
3.86
3.85
3.67
3.59
3.59
3.58
3.57
3.56
3.08
3.08
3.07
3.06
2.87
2.85
2.85
2.83
2.66
2.64
2.64
2.63
2.62
2.19
1.68
1.67
2.11
1.10
2.20
5.27
5.07
1.00
1.10
1.23
2.10
1.26
1.39
1.05
0.82
3.17
4.19
1.13
3.22
1.06
2.11
2.22
2.11
3.03
2.10
f1 (ppm)

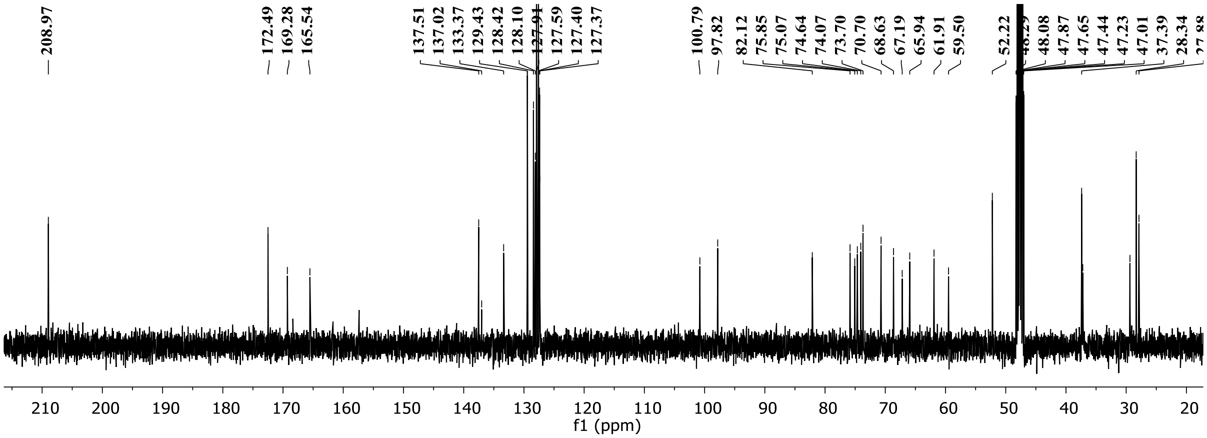

208.97
172.49
169.28
165.54
137.51
137.02
133.37
129.43
128.42
128.10
127.91
127.59
127.40
127.37
100.79
97.82
82.12
75.85
75.07
74.64
74.07
73.70
70.70
68.63
67.19
65.94
61.91
59.50
52.22
48.08
47.87
47.65
47.44
47.23
47.01
37.39
28.34
f1 (ppm)

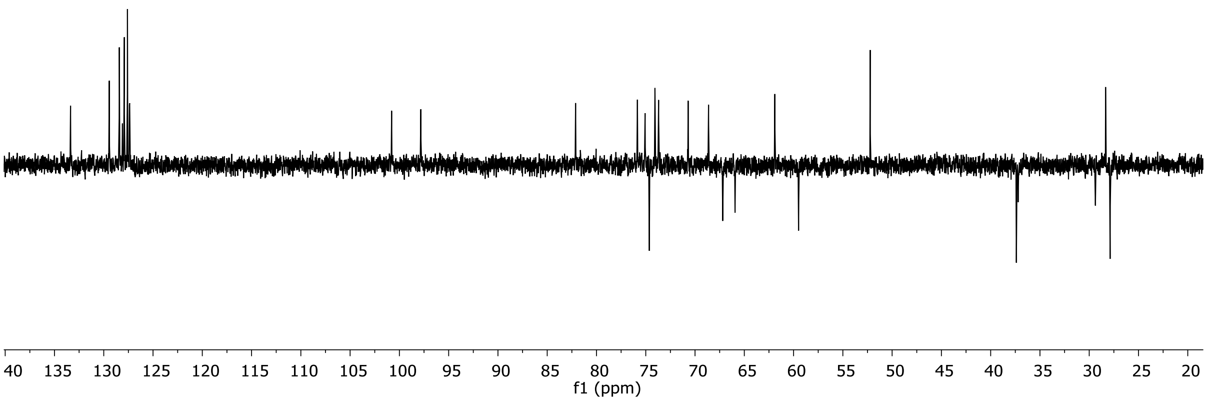

f1 (ppm)

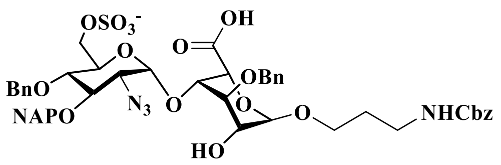
OSO3-
OH
O
O
OBn
BnO
NAPO
N3
O
O
O
NHCbz
HO

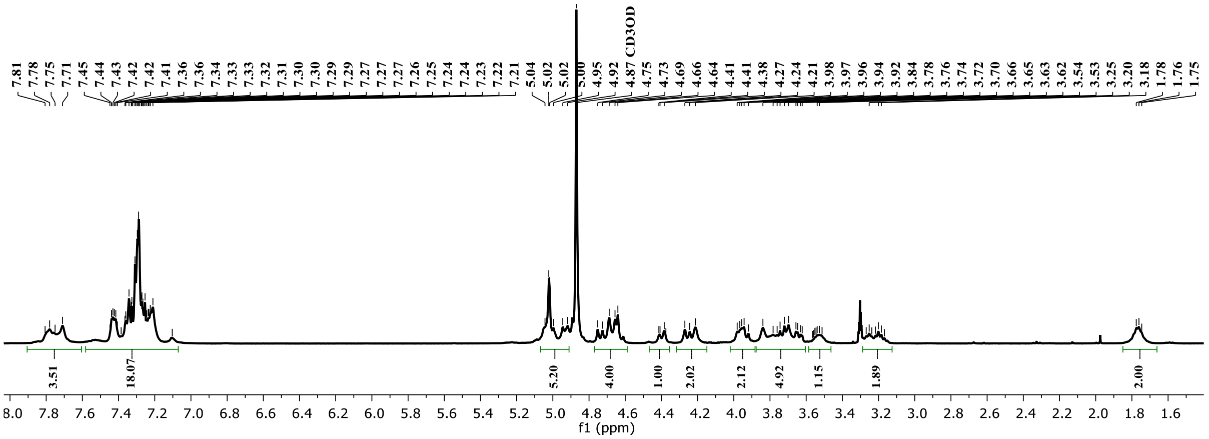
7.81 7.78 7.75 7.71 7.45 7.44 7.43 7.42 7.42 7.41 7.36 7.36 7.34 7.33 7.33 7.32 7.31 7.30 7.30 7.29 7.29 7.27 7.27 7.27 7.26 7.25 7.24 7.24 7.23 7.22 7.21 7.21 5.04 5.02 5.02 5.00 4.95 4.92 4.87 4.75 4.73 4.69 4.66 4.64 4.41 4.41 4.38 4.27 4.24 4.21 3.98 3.97 3.96 3.94 3.92 3.84 3.78 3.76 3.74 3.72 3.70 3.66 3.65 3.63 3.62 3.54 3.53 3.25 3.20 3.18 1.78 1.76 1.75
CD3OD
3.51
18.07
5.20
4.00
1.00
2.02
2.12
4.92
1.15
1.89
2.00
f1 (ppm)

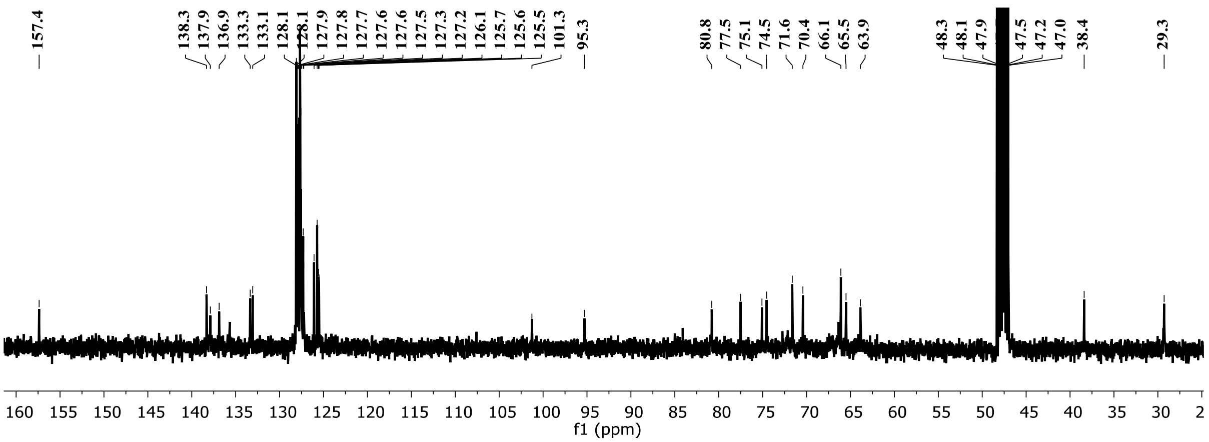
157.4 138.3 137.9 136.9 133.3 133.1 128.1 128.1 127.9 127.8 127.7 127.6 127.5 127.3 127.2 126.1 125.7 125.6 125.5 101.3 95.3 80.8 77.5 75.1 74.5 71.6 70.4 66.1 65.5 63.9 48.3 48.1 47.9 47.5 47.2 47.0 38.4 29.3
f1 (ppm)

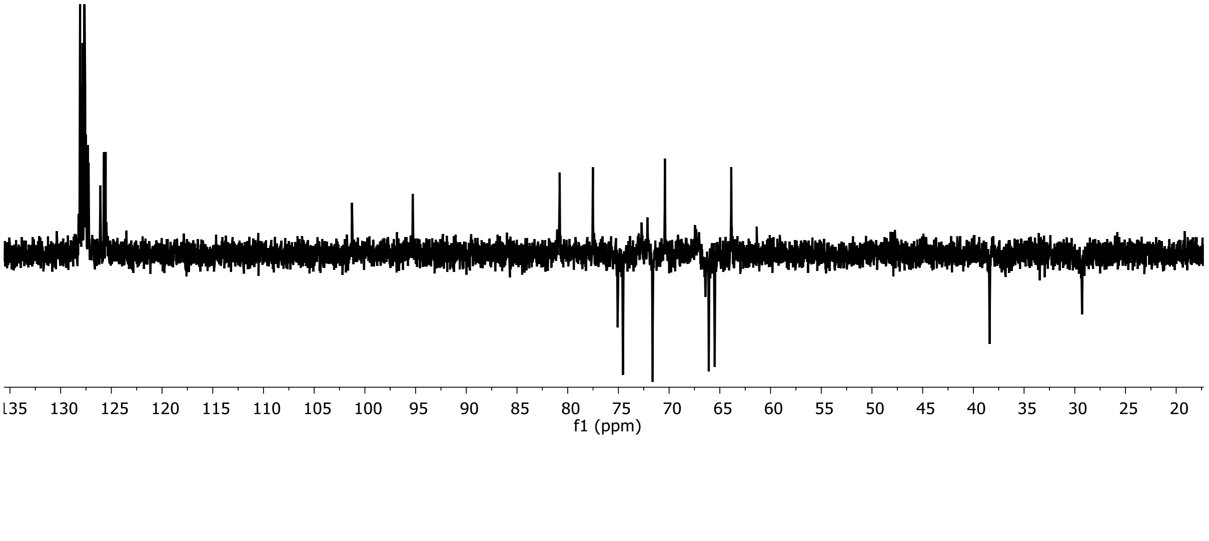
f1 (ppm)

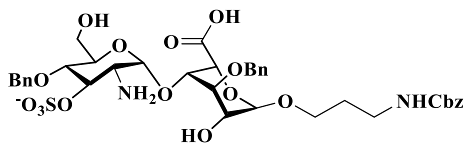
OH
OH
O
O
BnO
OBn
NH2O
-O3SO
O
O
NHCbz
HO

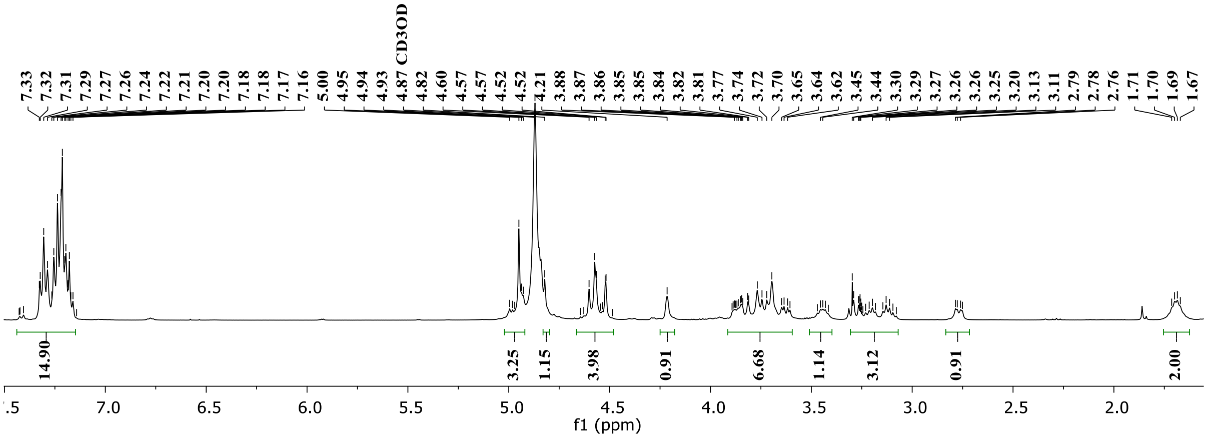
7.33 7.32 7.31 7.29 7.27 7.26 7.24 7.22 7.21 7.20 7.20 7.18 7.17 7.16 5.00 4.95 4.94 4.93 4.87 4.82 4.60 4.57 4.57 4.52 4.52 4.21 3.88 3.87 3.86 3.85 3.85 3.84 3.82 3.81 3.77 3.74 3.72 3.70 3.65 3.64 3.62 3.45 3.44 3.30 3.29 3.27 3.26 3.26 3.25 3.20 3.13 3.11 2.79 2.78 2.76 1.71 1.70 1.69 1.67
CD3OD
14.90
3.25
1.15
3.98
0.91
6.68
1.14
3.12
0.91
2.00
f1 (ppm)

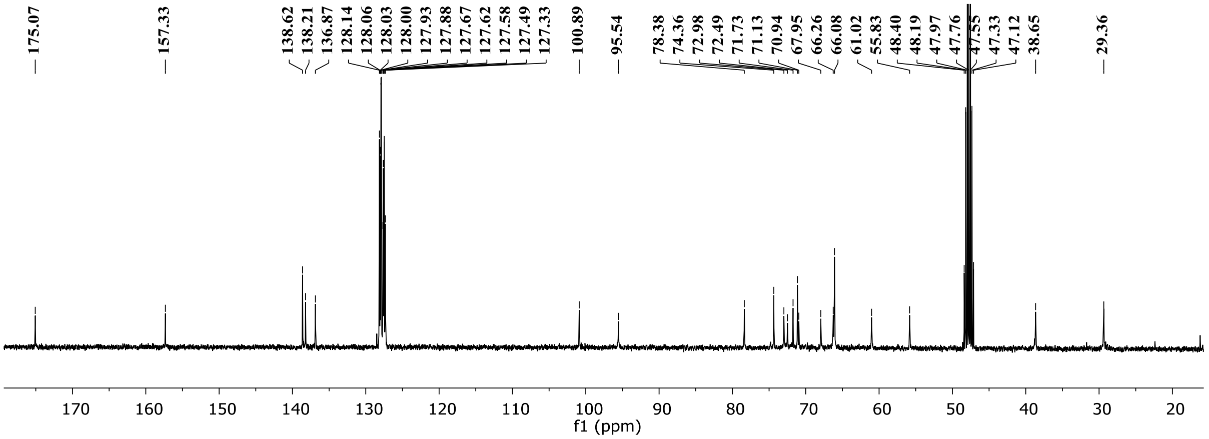
175.07
157.33
138.62 138.21 136.87 128.14 128.06 128.03 128.00 127.93 127.88 127.67 127.62 127.58 127.49 127.33
100.89
95.54
78.38 74.36 72.98 72.49 71.73 71.13 70.94 67.95 66.26 66.08 61.02 55.83 48.40 48.19 47.97 47.76 47.55 47.33 47.12 38.65
29.36
f1 (ppm)

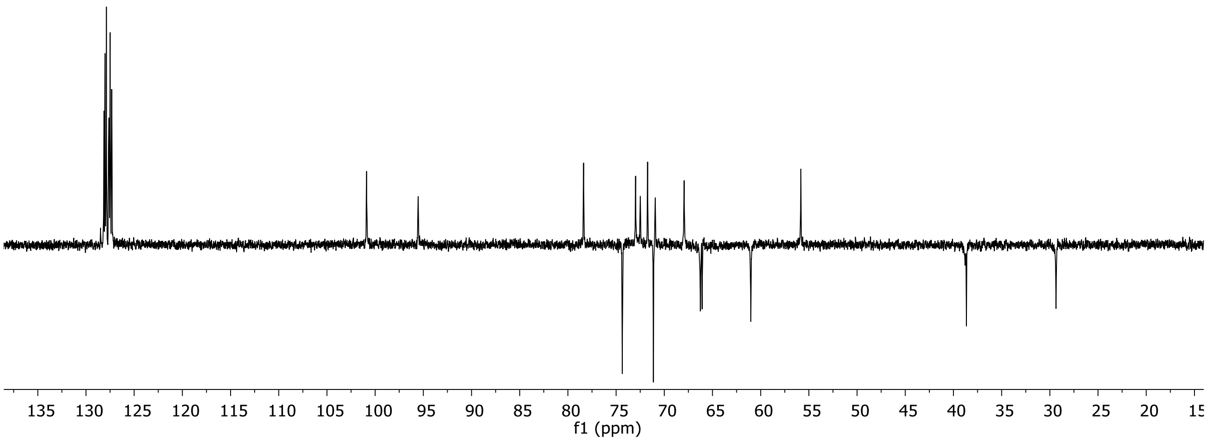
f1 (ppm)

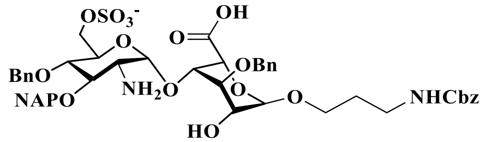
OSO3-
OH
O
O
BnO
OBn
NAPO
NH2O
O
O
NHCbz
HO

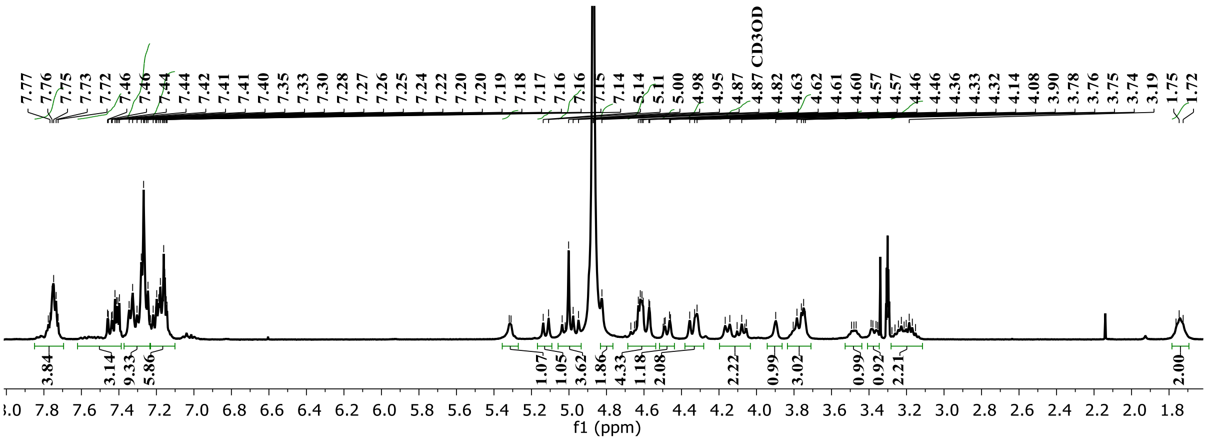
7.77 7.76 7.75 7.73 7.72 7.46 7.46 7.44 7.44 7.42 7.41 7.41 7.40 7.35 7.33 7.30 7.28 7.27 7.26 7.25 7.24 7.22 7.20 7.20 7.19 7.18 7.17 7.16 7.16 7.15 7.14 5.14 5.11 5.00 4.98 4.95 4.87 4.87 4.82 4.63 4.62 4.61 4.60 4.57 4.57 4.46 4.46 4.36 4.33 4.32 4.14 4.08 3.90 3.78 3.76 3.75 3.74 3.19 1.75 1.72
CD3OD
3.84 3.14 9.33 5.86 1.07 1.05 3.62 1.86 4.33 1.18 2.08 2.22 0.99 3.02 0.99 0.92 2.21 2.00
f1 (ppm)

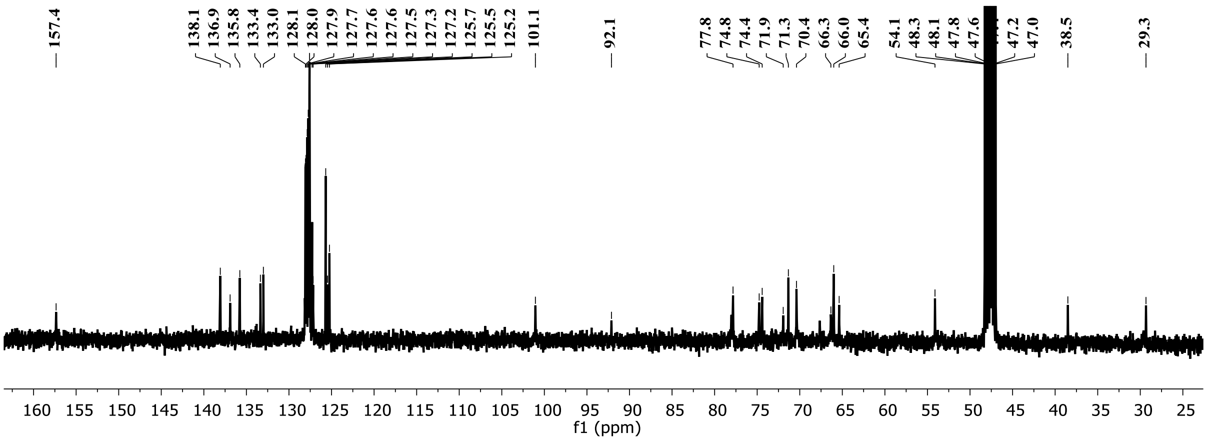
157.4 138.1 136.9 135.8 133.4 133.0 128.1 128.0 127.9 127.7 127.6 127.5 127.3 127.2 125.7 125.5 125.2 101.1 92.1 77.8 74.8 74.4 71.9 71.3 70.4 66.3 66.0 65.4 54.1 48.3 48.1 47.8 47.6 47.2 47.0 38.5 29.3
f1 (ppm)

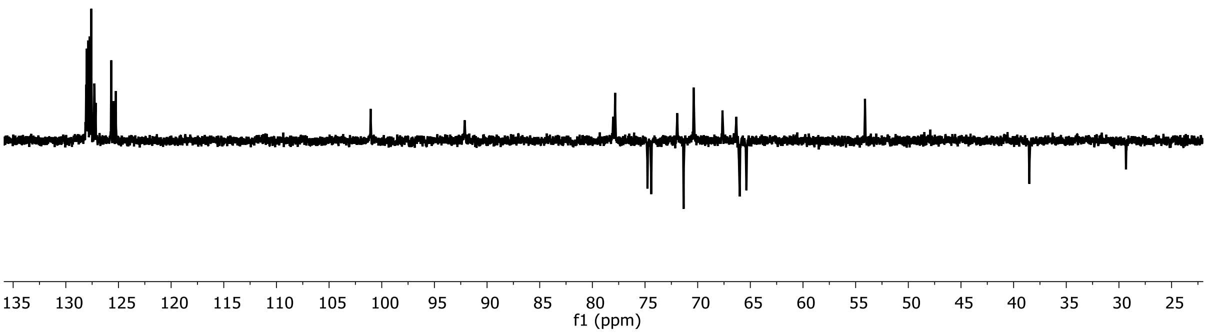
f1 (ppm)

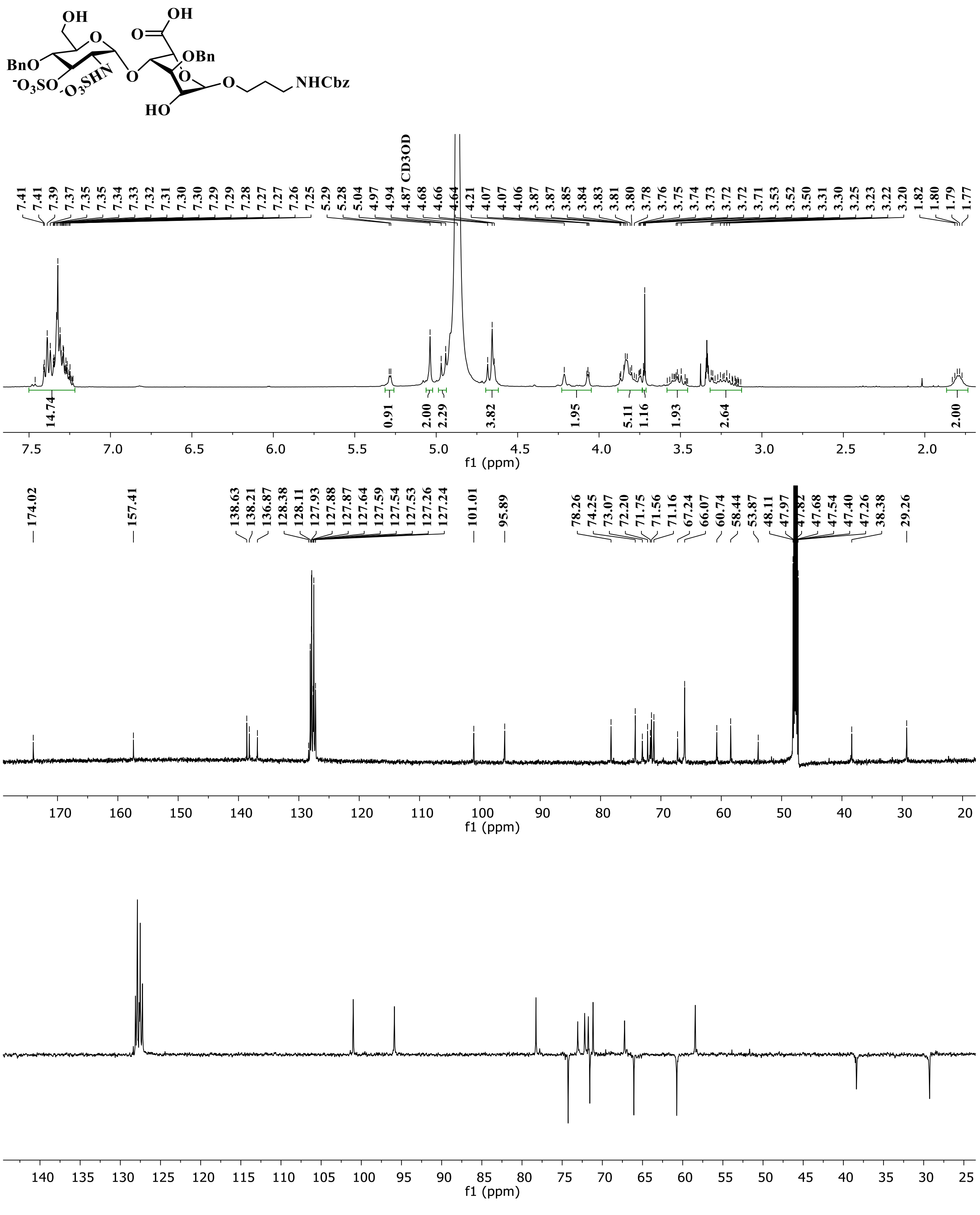
OH
O
OH
O
BnO
OBn
$^{-}O_3SO$
$^{-}O_3SHN$
O
O
O
NHCbz
HO
CD3OD
f1 (ppm)
f1 (ppm)
f1 (ppm)

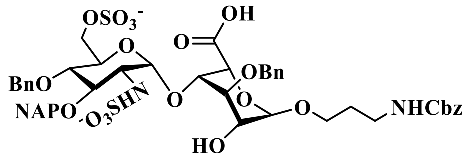
OSO3-
OH
O
O
OBn
BnO
NAPO
-O3SHN
O
O
O
NHCbz
HO

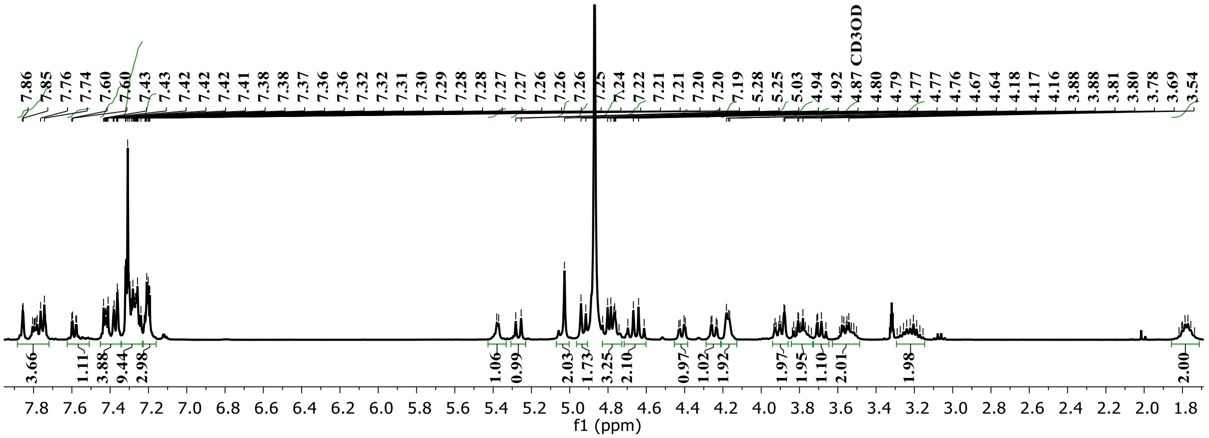
7.86 7.85 7.76 7.74 7.60 7.60 7.43 7.43 7.42 7.42 7.42 7.41 7.38 7.38 7.37 7.36 7.36 7.32 7.31 7.30 7.29 7.28 7.28 7.27 7.27 7.26 7.26 7.26 7.25 7.24 7.22 7.21 7.21 7.20 7.20 7.19 5.28 5.25 5.03 4.94 4.92 4.87 CD3OD 4.80 4.79 4.77 4.77 4.76 4.67 4.64 4.18 4.17 4.16 3.88 3.88 3.81 3.80 3.78 3.69 3.54
3.66 1.11 3.88 9.44 2.98 1.06 0.99 2.03 1.73 3.25 2.10 0.97 1.02 1.92 1.97 1.95 1.10 2.01 1.98 2.00
f1 (ppm)

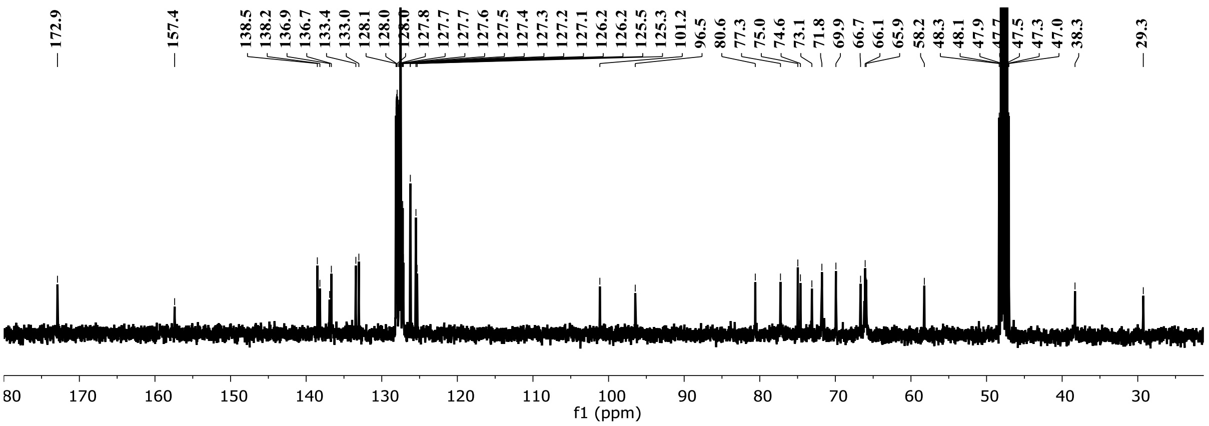
172.9 157.4 138.5 138.2 136.9 136.7 133.4 133.0 128.1 128.0 128.0 127.8 127.7 127.7 127.6 127.5 127.4 127.3 127.2 127.1 126.2 126.2 125.5 125.3 101.2 96.5 80.6 77.3 75.0 74.6 73.1 71.8 69.9 66.7 66.1 65.9 58.2 48.3 48.1 47.9 47.7 47.5 47.3 47.0 38.3 29.3
f1 (ppm)

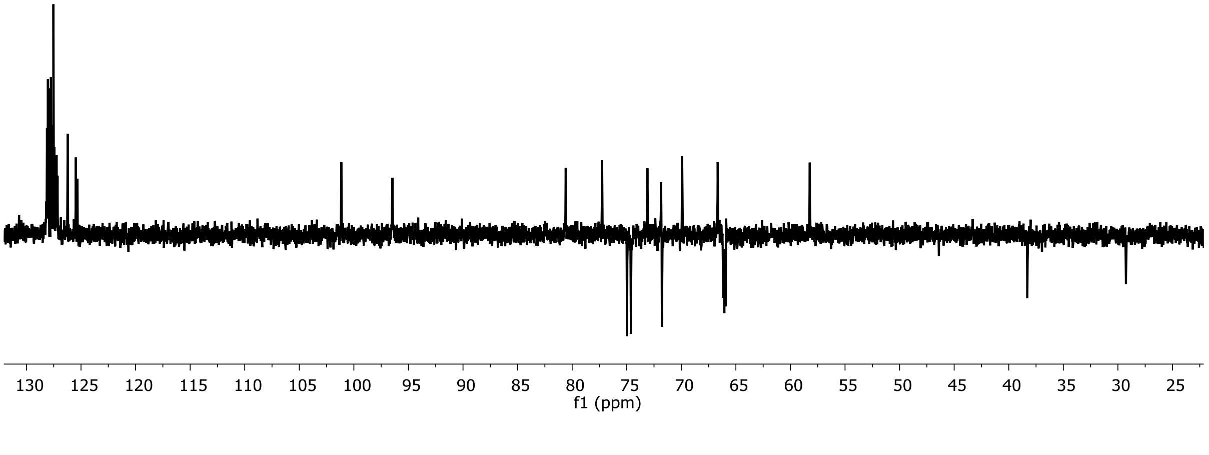
f1 (ppm)

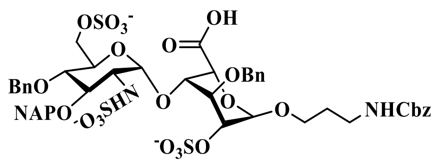
OSO3-
OH
O
BnO
NAPO
-O3SHN
OBn
O
NHCbz
-O3SO

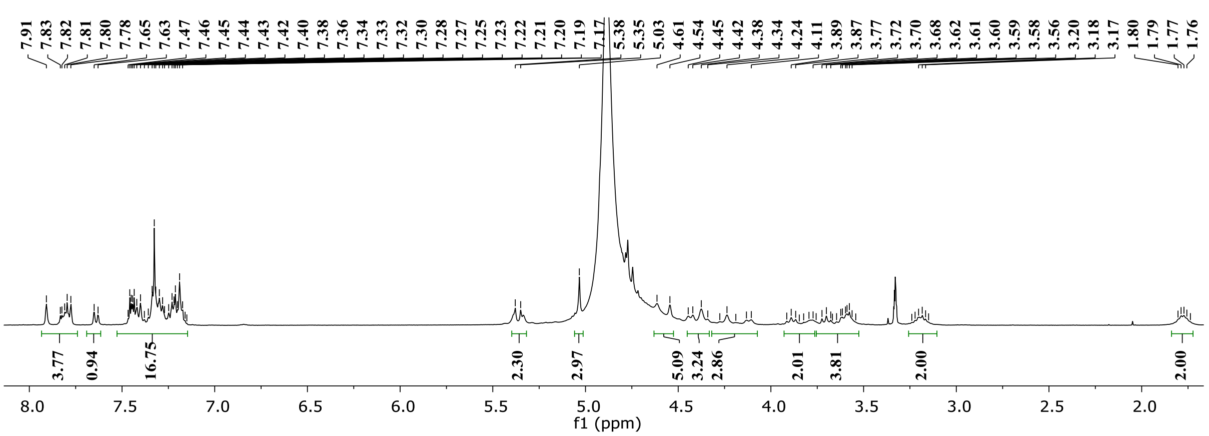
7.91 7.83 7.82 7.81 7.80 7.78 7.65 7.63 7.47 7.46 7.45 7.44 7.43 7.42 7.40 7.38 7.36 7.34 7.33 7.32 7.30 7.28 7.27 7.25 7.23 7.22 7.21 7.20 7.19 7.17 5.38 5.35 5.03 4.61 4.54 4.45 4.42 4.38 4.34 4.24 4.11 3.89 3.87 3.77 3.72 3.70 3.68 3.62 3.61 3.60 3.59 3.58 3.56 3.20 3.18 3.17 1.80 1.79 1.77 1.76
3.77
0.94
16.75
2.30
2.97
5.09
3.24
2.86
2.01
3.81
2.00
2.00
8.0 7.5 7.0 6.5 6.0 5.5 5.0 4.5 4.0 3.5 3.0 2.5 2.0
f1 (ppm)

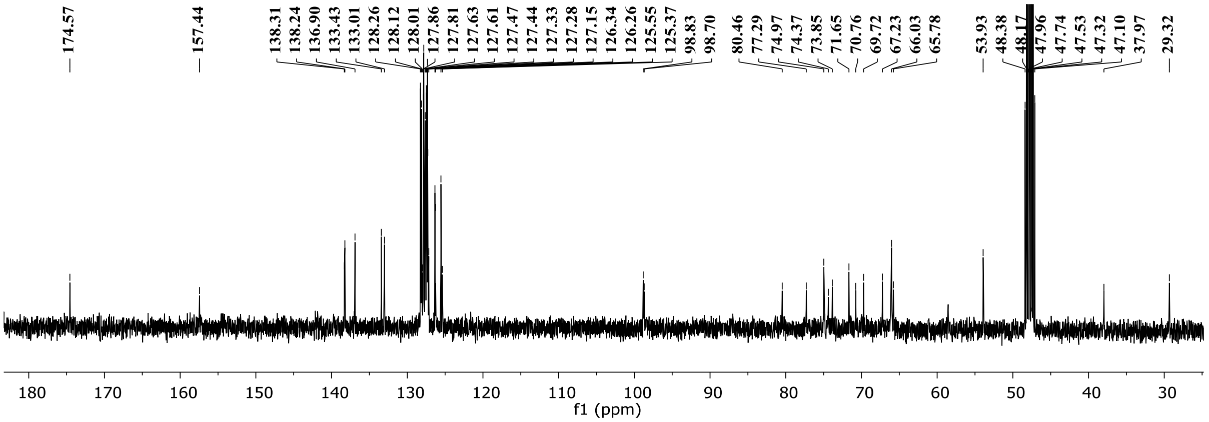
174.57
157.44
138.31 138.24 136.90 133.43 133.01 128.26 128.12 128.01 127.86 127.81 127.63 127.61 127.47 127.44 127.33 127.28 127.15 126.34 126.26 125.55 125.37 98.83 98.70
80.46 77.29 74.97 74.37 73.85 71.65 70.76 69.72 67.23 66.03 65.78
53.93 48.38 48.17 47.96 47.74 47.53 47.32 47.10 37.97 29.32
180 170 160 150 140 130 120 110 100 90 80 70 60 50 40 30
f1 (ppm)

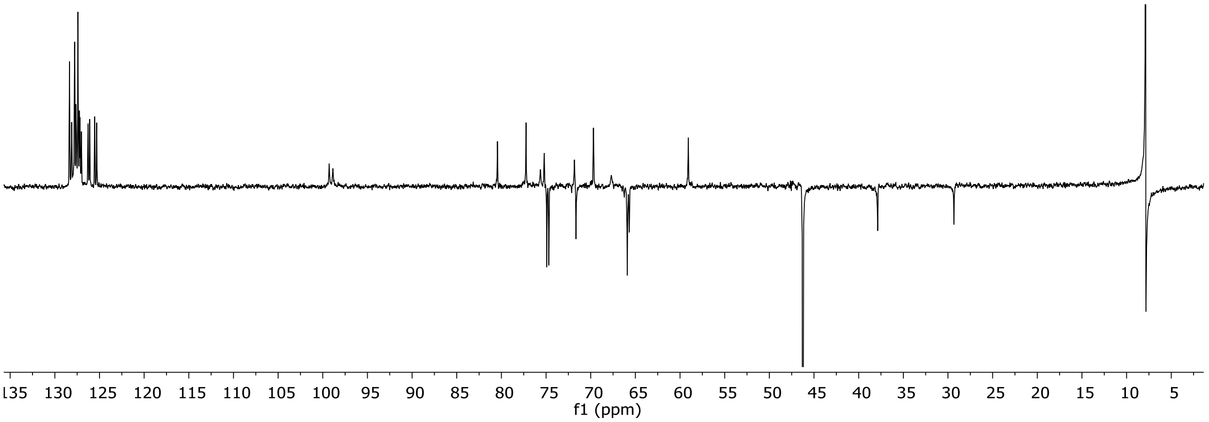
135 130 125 120 115 110 105 100 95 90 85 80 75 70 65 60 55 50 45 40 35 30 25 20 15 10 5
f1 (ppm)

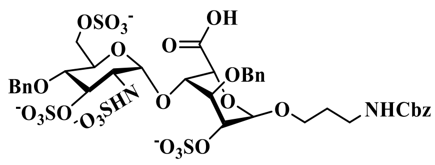

OSO3-
OH
O
OBn
BnO
-O3SO
-O3SHN
O
O
NHCbz
-O3SO

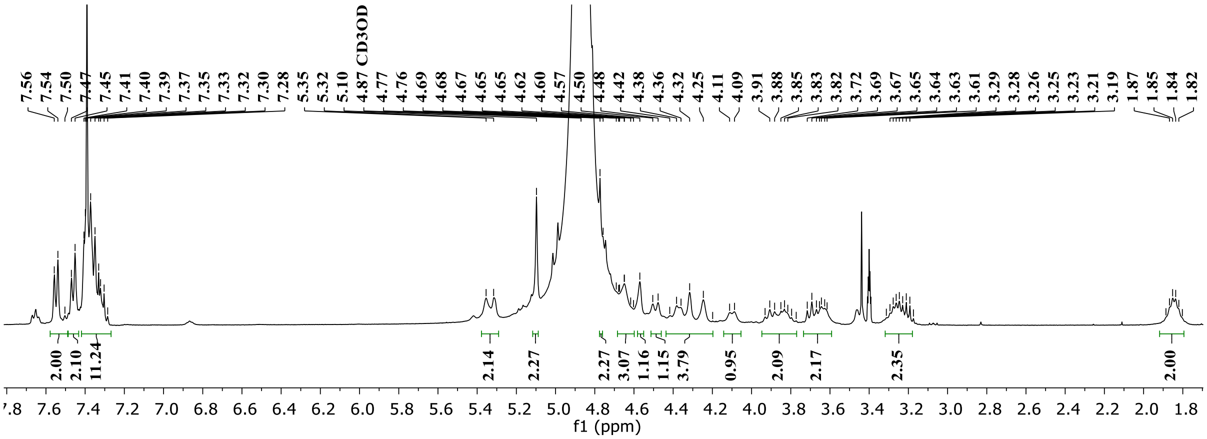

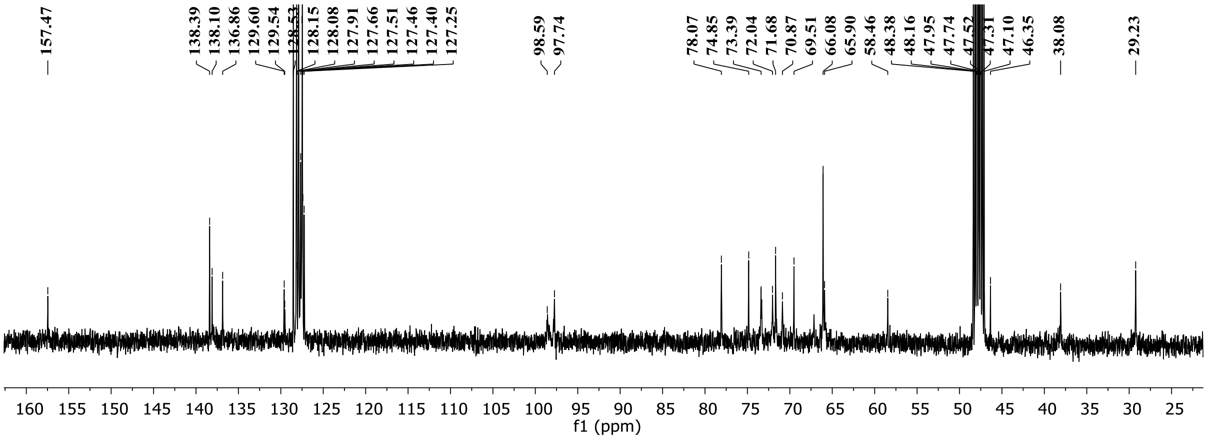

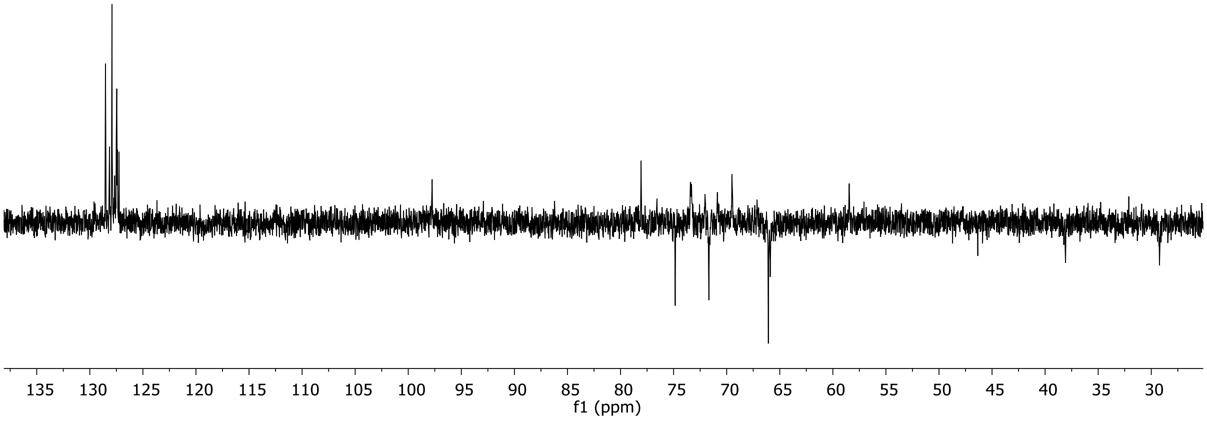

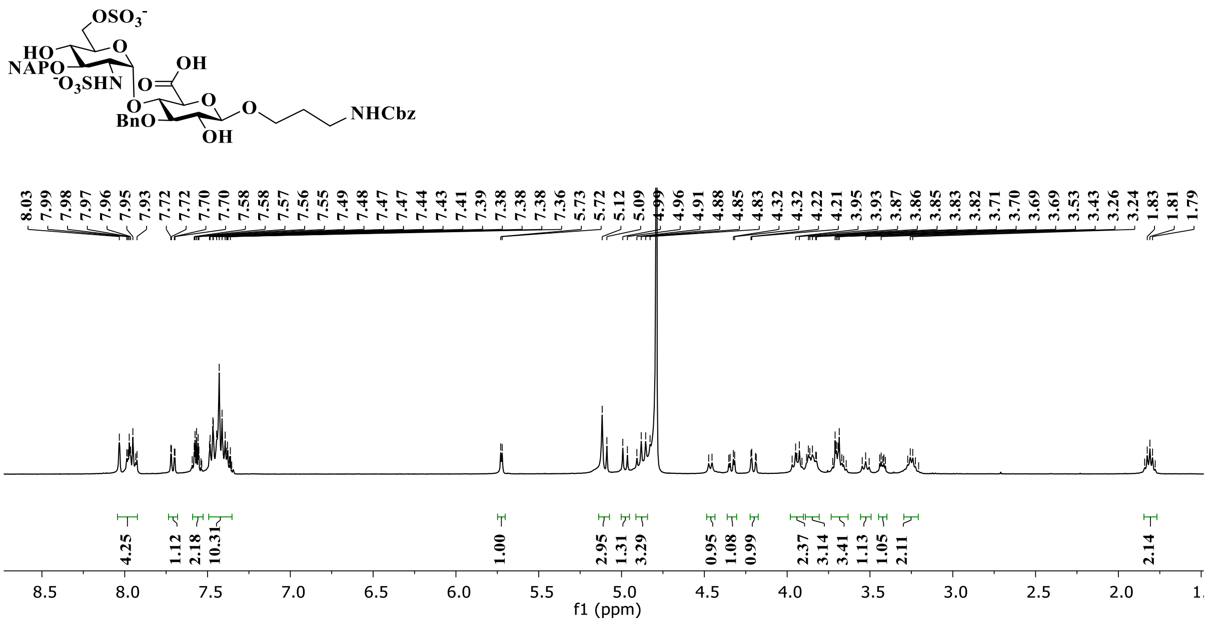
OSO3-
HO
NAPO
-O3SHN
O
OH
BnO
OH
NHCbz
8.03 7.99 7.98 7.97 7.96 7.95 7.93 7.72 7.72 7.70 7.70 7.58 7.58 7.57 7.56 7.55 7.49 7.48 7.47 7.47 7.44 7.43 7.41 7.39 7.38 7.38 7.38 7.36 5.73 5.72 5.12 5.09 4.99 4.96 4.91 4.88 4.85 4.83 4.32 4.32 4.22 4.21 3.95 3.93 3.87 3.86 3.85 3.83 3.82 3.71 3.70 3.69 3.53 3.43 3.26 3.24 1.83 1.81 1.79
4.25 1.12 2.18 10.31 1.00 2.95 1.31 3.29 0.95 1.08 0.99 2.37 3.14 3.41 1.13 1.05 2.11 2.14
f1 (ppm)

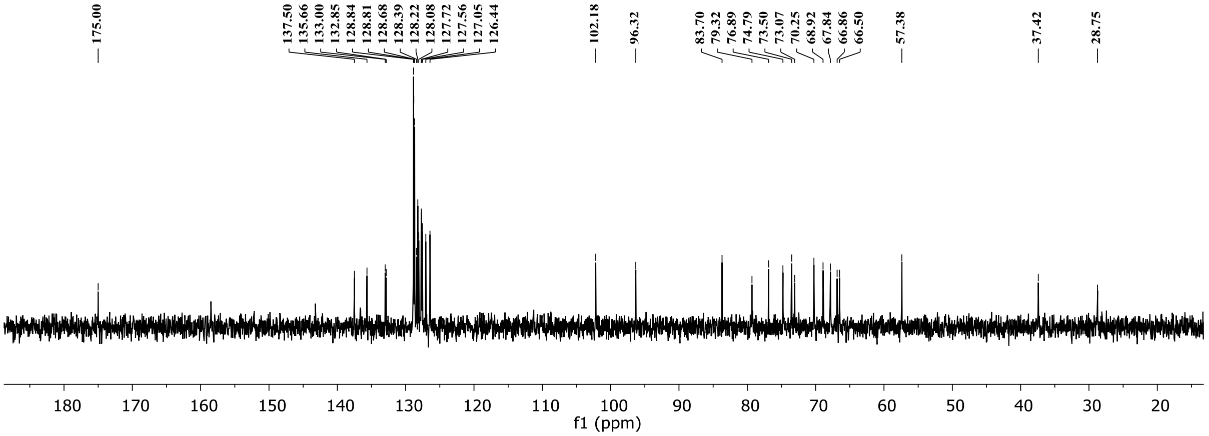
175.00 137.50 135.66 133.00 132.85 128.84 128.81 128.68 128.39 128.22 128.08 127.72 127.56 127.05 126.44 102.18 96.32 83.70 79.32 76.89 74.79 73.50 73.07 70.25 68.92 67.84 66.86 66.50 57.38 37.42 28.75
f1 (ppm)

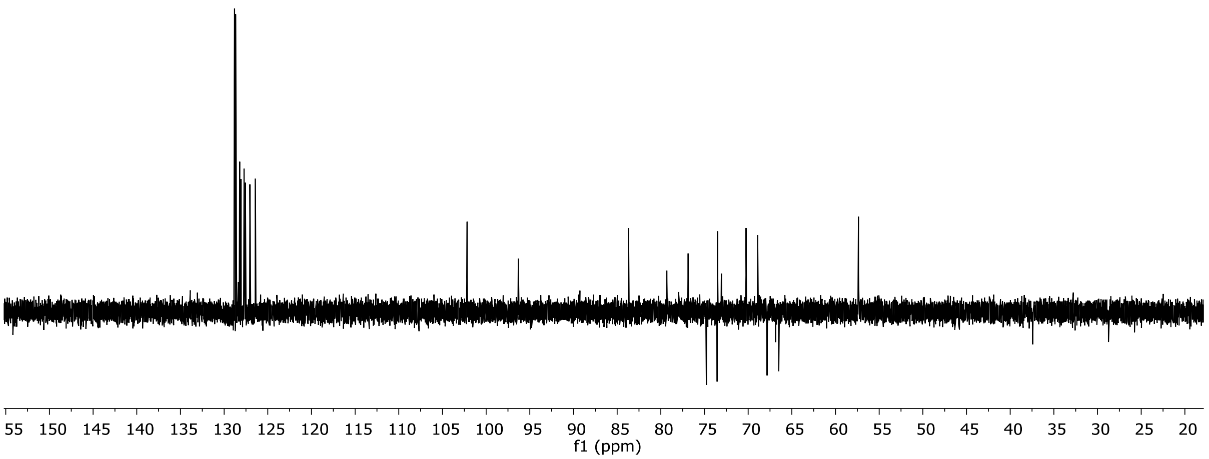
f1 (ppm)

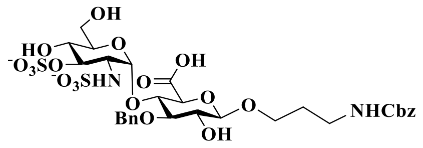
OH
HO
$^{-}O_3SO$
$^{-}O_3SHN$
O
OH
O
BnO
OH
NHCbz

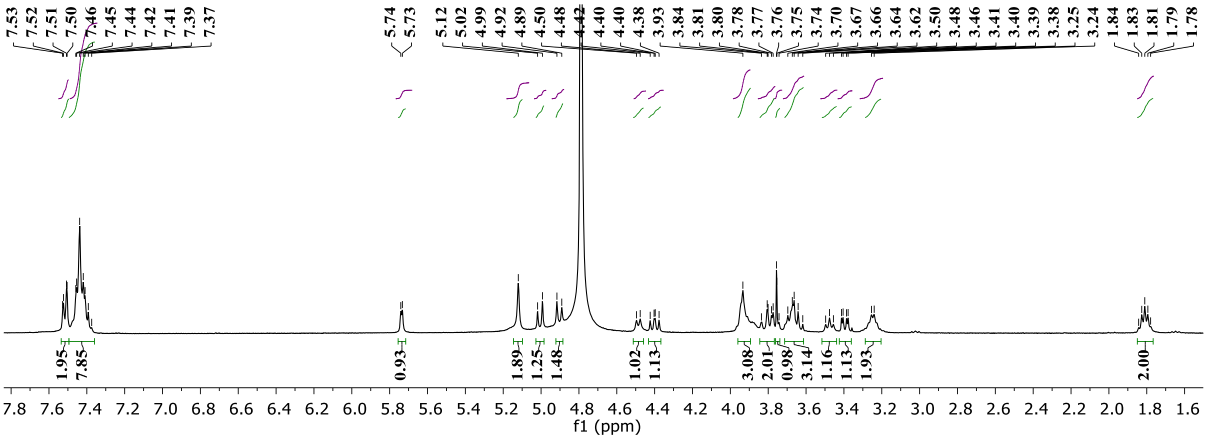
7.53 7.52 7.51 7.50 7.46 7.45 7.44 7.42 7.41 7.39 7.37
5.74 5.73
5.12 5.02 4.99 4.92 4.89 4.50 4.48 4.42 4.40 4.40 4.38 3.93 3.84 3.81 3.80 3.78 3.77 3.76 3.75 3.74 3.70 3.67 3.66 3.64 3.62 3.50 3.48 3.46 3.41 3.40 3.39 3.38 3.25 3.24
1.84 1.83 1.81 1.79 1.78
1.95 7.85 0.93 1.89 1.25 1.48 1.02 1.13 3.08 2.01 0.98 3.14 1.16 1.13 1.93 2.00
7.8 7.6 7.4 7.2 7.0 6.8 6.6 6.4 6.2 6.0 5.8 5.6 5.4 5.2 5.0 4.8 4.6 4.4 4.2 4.0 3.8 3.6 3.4 3.2 3.0 2.8 2.6 2.4 2.2 2.0 1.8 1.6
f1 (ppm)

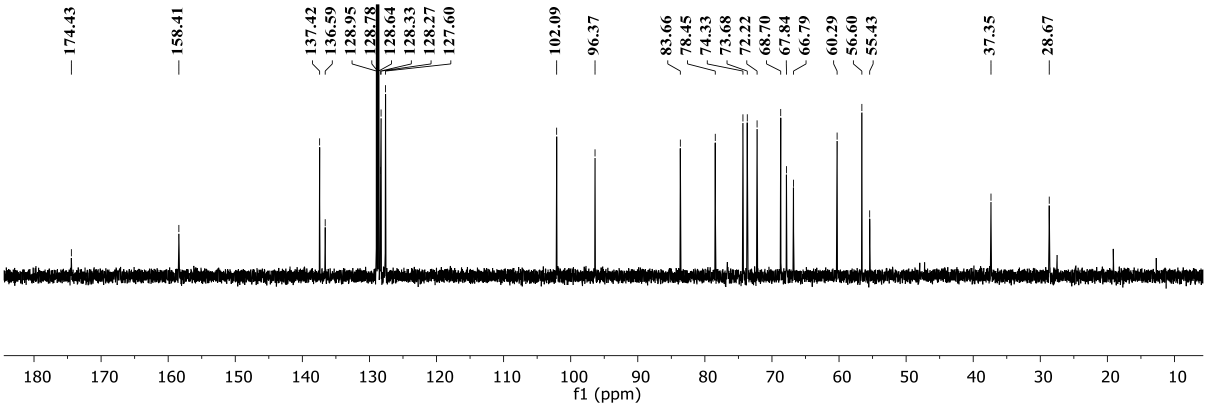
174.43
158.41
137.42 136.59 128.95 128.78 128.64 128.33 128.27 127.60
102.09
96.37
83.66 78.45 74.33 73.68 72.22 68.70 67.84 66.79
60.29 56.60 55.43
37.35
28.67
180 170 160 150 140 130 120 110 100 90 80 70 60 50 40 30 20 10
f1 (ppm)

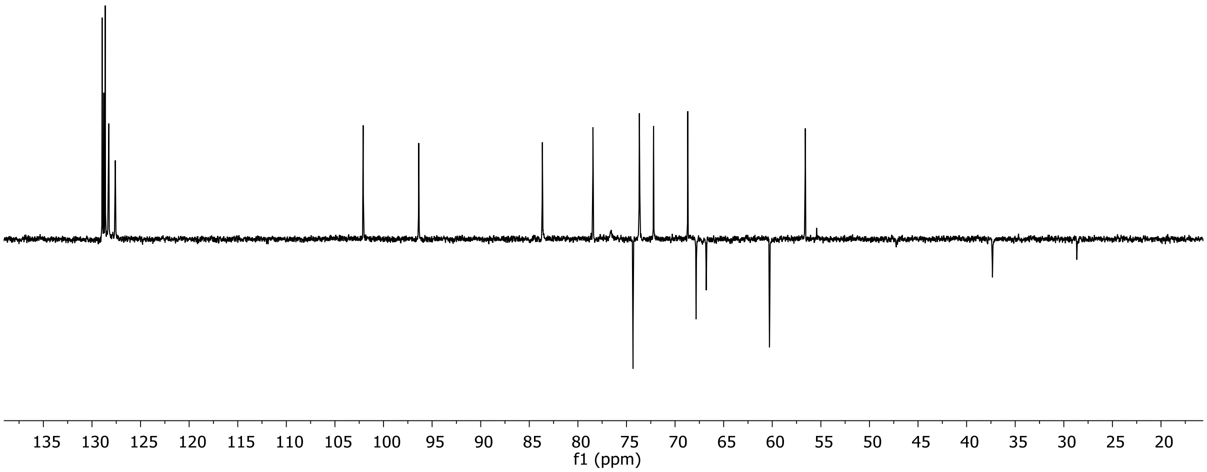
135 130 125 120 115 110 105 100 95 90 85 80 75 70 65 60 55 50 45 40 35 30 25 20
f1 (ppm)

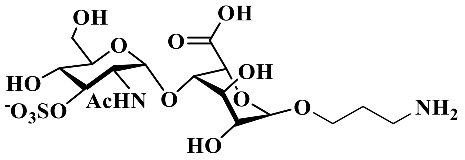
OH
O
OH
O
HO
OH
$^{-}O_3SO$
AcHN
O
O
O
NH_2
HO

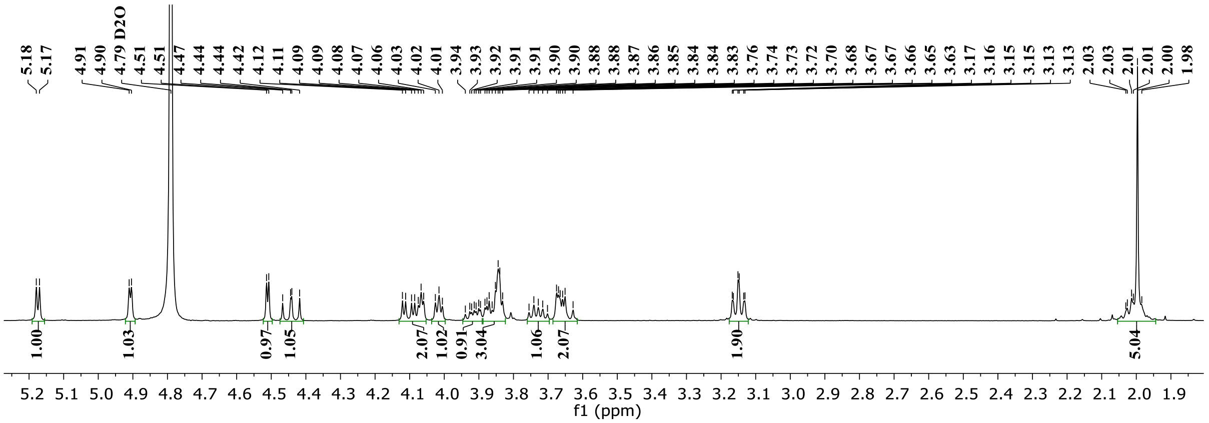
5.18
5.17
4.91
4.90
4.79 D2O
4.51
4.51
4.47
4.44
4.44
4.42
4.12
4.11
4.09
4.09
4.08
4.07
4.06
4.03
4.02
4.01
3.94
3.93
3.92
3.91
3.91
3.90
3.90
3.88
3.87
3.86
3.85
3.84
3.84
3.83
3.76
3.74
3.73
3.72
3.70
3.68
3.67
3.66
3.65
3.63
3.17
3.16
3.15
3.15
3.13
3.13
2.03
2.03
2.01
2.01
2.00
1.98
1.00
1.03
0.97
1.05
2.07
1.02
0.91
3.04
1.06
2.07
1.90
5.04
f1 (ppm)

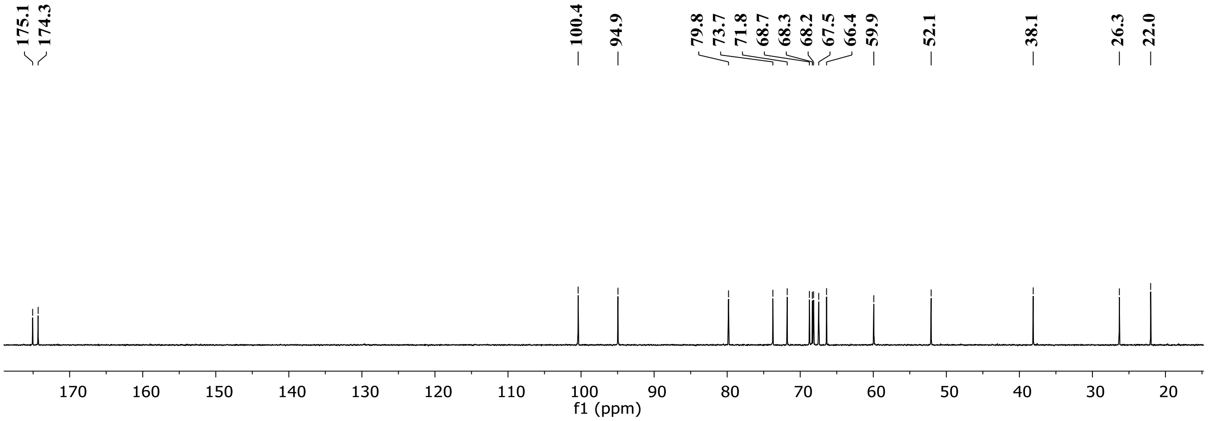
175.1
174.3
100.4
94.9
79.8
73.7
71.8
68.7
68.3
68.2
67.5
66.4
59.9
52.1
38.1
26.3
22.0
f1 (ppm)

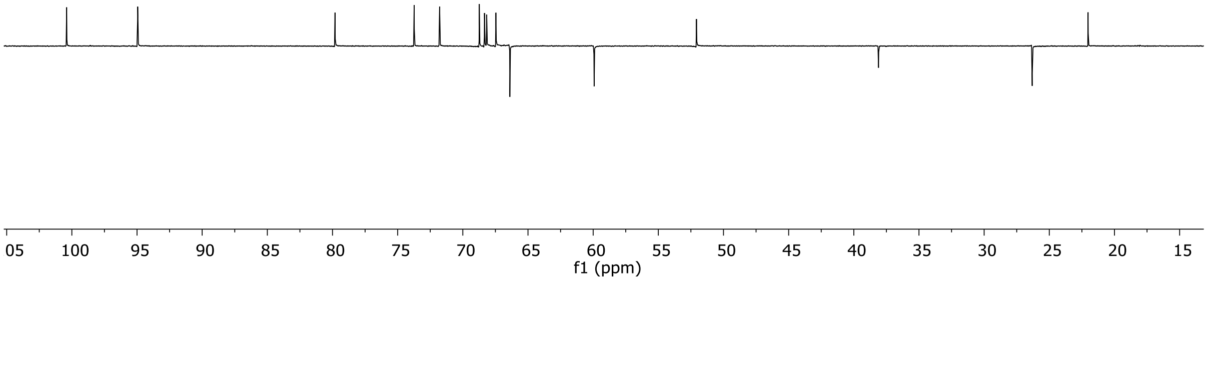
f1 (ppm)

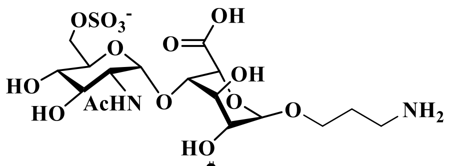

OSO3-
OH
O
HO
HO
AcHN
NH2

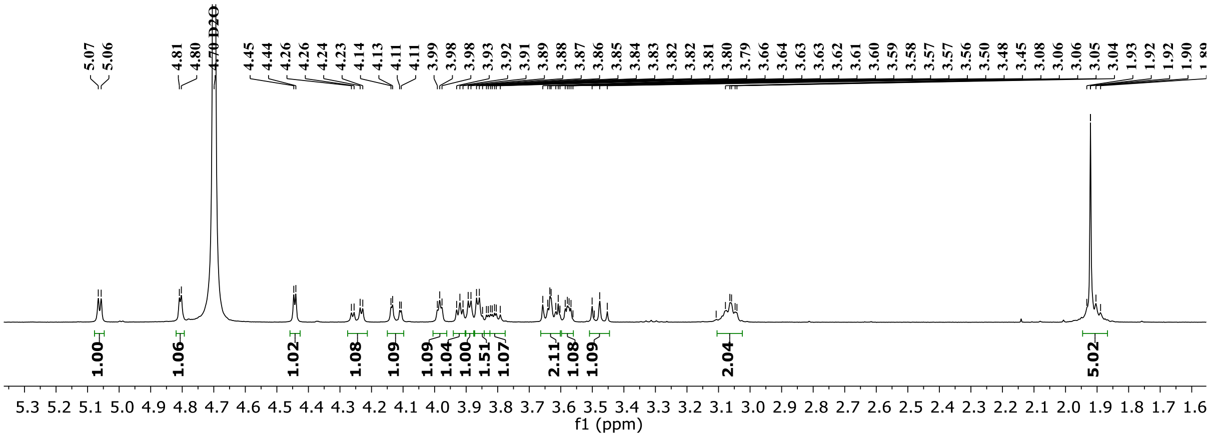

5.07
5.06
4.81
4.80
4.70 D2O
4.45
4.44
4.26
4.26
4.24
4.23
4.14
4.13
4.11
4.11
3.99
3.98
3.98
3.93
3.92
3.91
3.89
3.88
3.87
3.86
3.85
3.84
3.83
3.82
3.82
3.81
3.80
3.79
3.66
3.64
3.63
3.63
3.62
3.61
3.60
3.59
3.58
3.57
3.57
3.56
3.50
3.48
3.45
3.08
3.06
3.06
3.05
3.04
1.93
1.92
1.92
1.90
1.00
1.06
1.02
1.08
1.09
1.09
1.04
1.00
1.51
1.07
2.11
1.08
1.09
2.04
5.02
5.3 5.2 5.1 5.0 4.9 4.8 4.7 4.6 4.5 4.4 4.3 4.2 4.1 4.0 3.9 3.8 3.7 3.6 3.5 3.4 3.3 3.2 3.1 3.0 2.9 2.8 2.7 2.6 2.5 2.4 2.3 2.2 2.1 2.0 1.9 1.8 1.7 1.6
f1 (ppm)

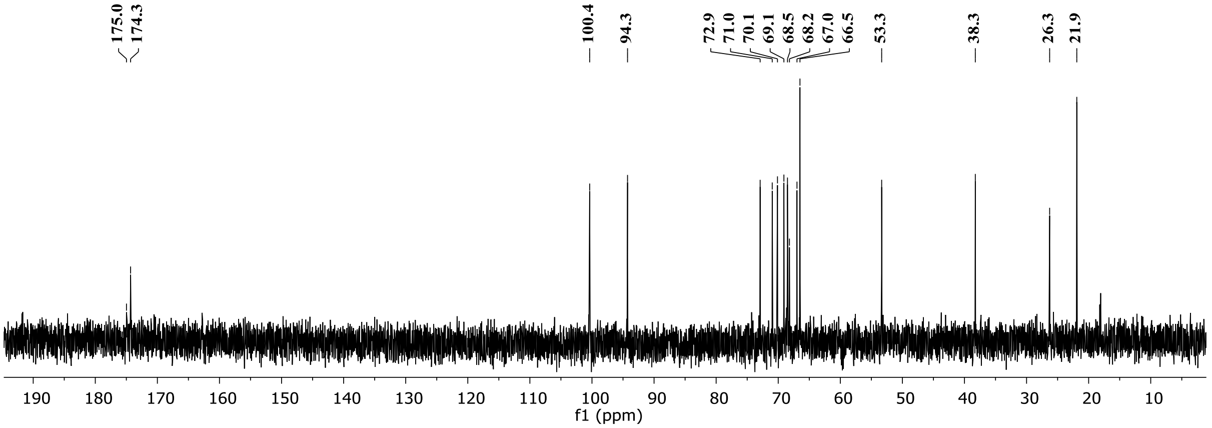

175.0
174.3
100.4
94.3
72.9
71.0
70.1
69.1
68.5
68.2
67.0
66.5
53.3
38.3
26.3
21.9
190 180 170 160 150 140 130 120 110 100 90 80 70 60 50 40 30 20 10
f1 (ppm)

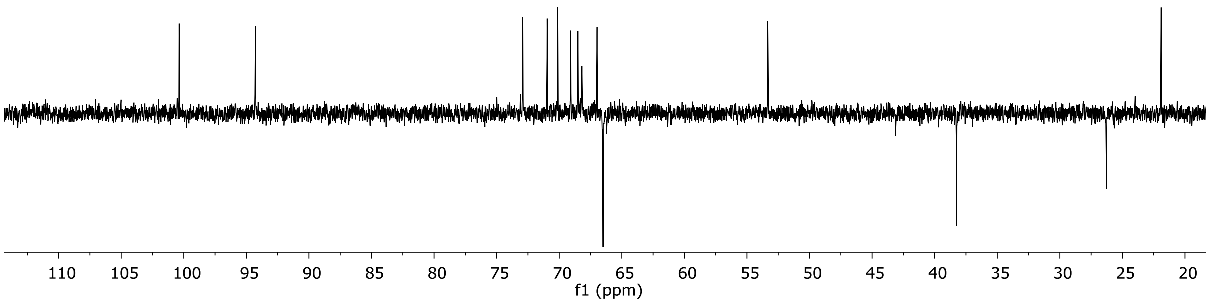

110 105 100 95 90 85 80 75 70 65 60 55 50 45 40 35 30 25 20
f1 (ppm)

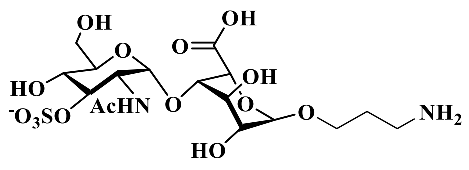
OH
O
HO
-O3SO
AcHN
O
OH
O
OH
O
O
NH2
HO

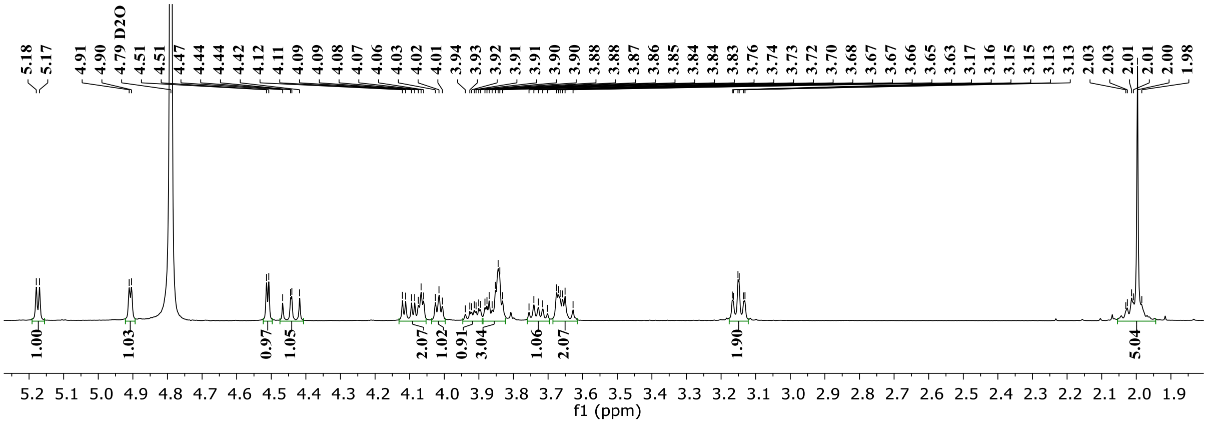
5.18
5.17
4.91
4.90
4.79 D2O
4.51
4.51
4.47
4.44
4.44
4.42
4.12
4.11
4.09
4.09
4.08
4.07
4.06
4.03
4.02
4.01
3.94
3.93
3.92
3.91
3.91
3.90
3.90
3.88
3.87
3.86
3.85
3.84
3.84
3.83
3.76
3.74
3.73
3.72
3.70
3.68
3.67
3.66
3.65
3.63
3.17
3.16
3.15
3.15
3.13
3.13
2.03
2.03
2.01
2.01
2.00
1.98
1.00
1.03
0.97
1.05
2.07
1.02
0.91
3.04
1.06
2.07
1.90
5.04
f1 (ppm)

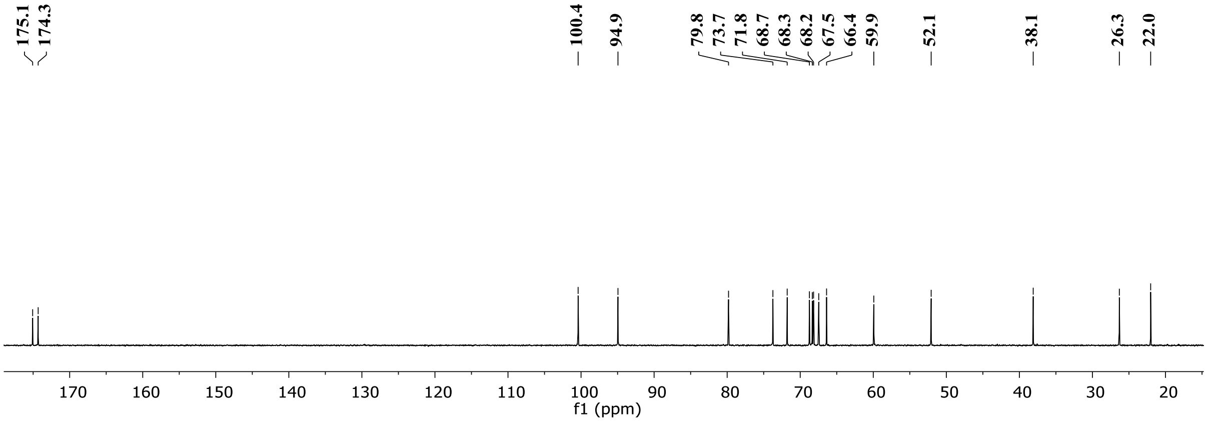
175.1
174.3
100.4
94.9
79.8
73.7
71.8
68.7
68.3
68.2
67.5
66.4
59.9
52.1
38.1
26.3
22.0
f1 (ppm)

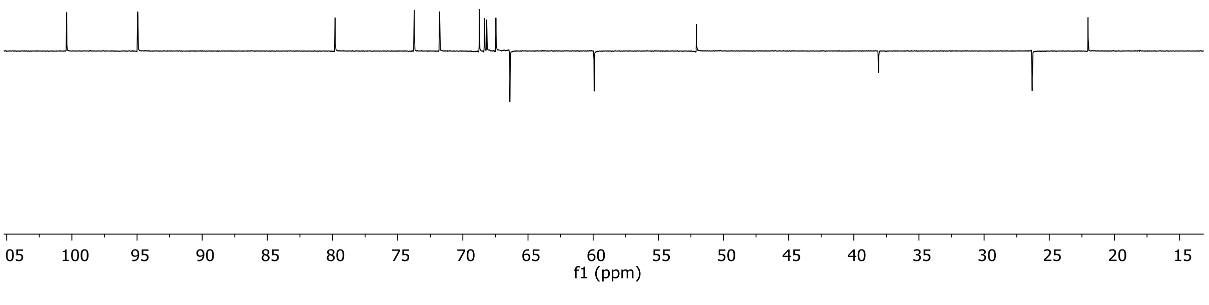
f1 (ppm)

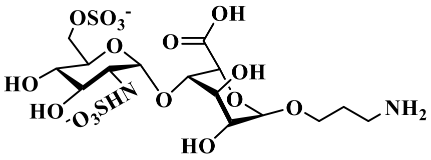
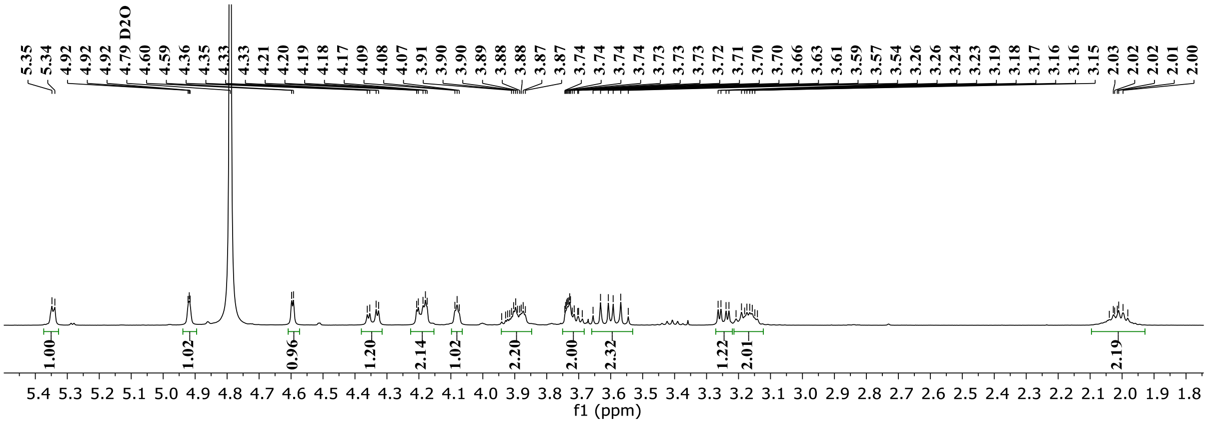
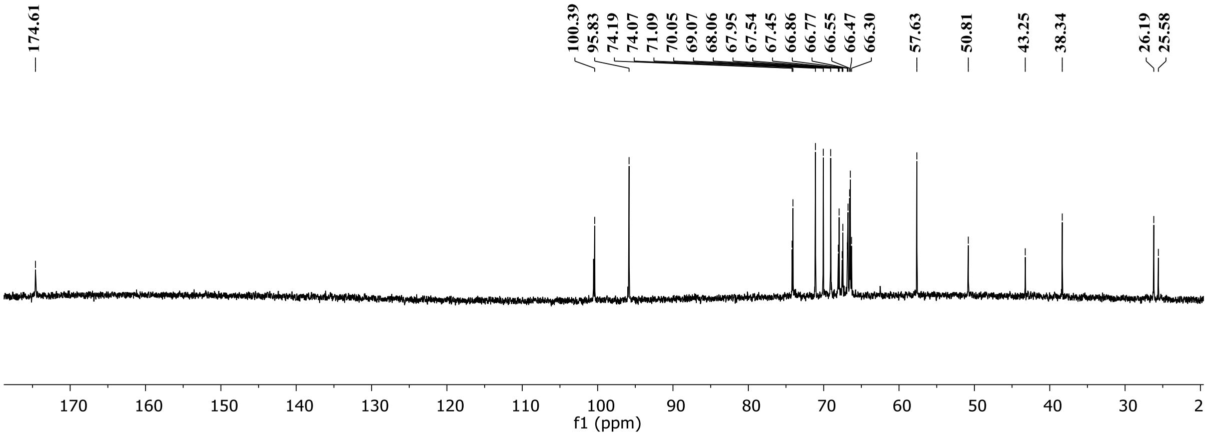

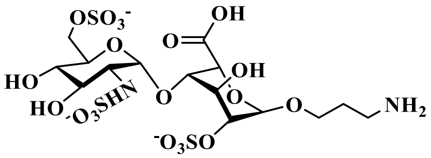
OSO3-
OH
O
O
OH
HO
O
HO
-O3SHN
O
O
NH2
-O3SO

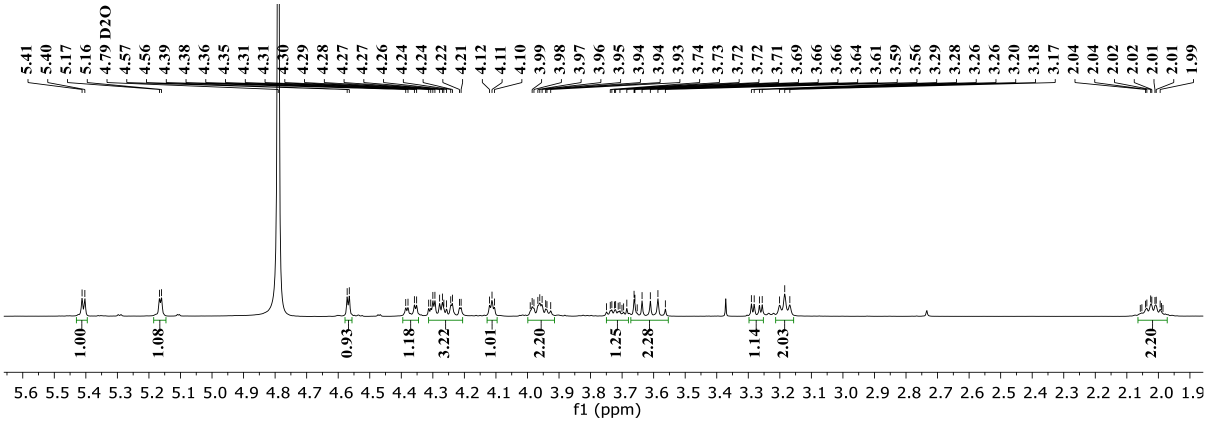
5.41 5.40 5.17 5.16 4.79 D2O 4.57 4.56 4.39 4.38 4.36 4.35 4.31 4.31 4.30 4.29 4.28 4.27 4.27 4.26 4.24 4.24 4.22 4.21 4.12 4.11 4.10 3.99 3.98 3.97 3.96 3.95 3.94 3.94 3.93 3.74 3.73 3.72 3.72 3.71 3.69 3.66 3.66 3.64 3.61 3.59 3.56 3.29 3.28 3.26 3.26 3.20 3.18 3.17 2.04 2.04 2.02 2.02 2.01 2.01 1.99
1.00 1.08 0.93 1.18 3.22 1.01 2.20 1.25 2.28 1.14 2.03 2.20
5.6 5.5 5.4 5.3 5.2 5.1 5.0 4.9 4.8 4.7 4.6 4.5 4.4 4.3 4.2 4.1 4.0 3.9 3.8 3.7 3.6 3.5 3.4 3.3 3.2 3.1 3.0 2.9 2.8 2.7 2.6 2.5 2.4 2.3 2.2 2.1 2.0 1.9
f1 (ppm)

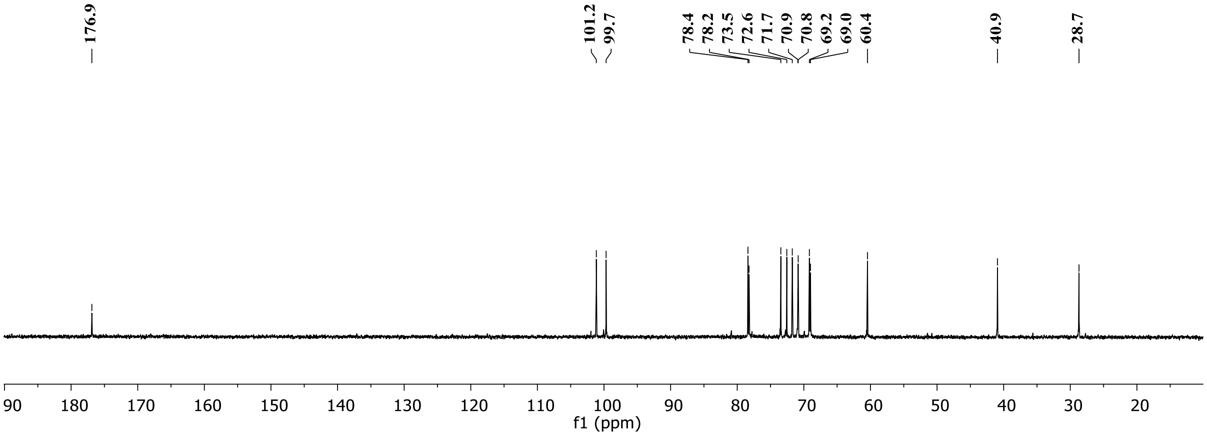
176.9
101.2 99.7
78.4 78.2 73.5 72.6 71.7 70.9 70.8 69.2 69.0 60.4
40.9
28.7
90 180 170 160 150 140 130 120 110 100 90 80 70 60 50 40 30 20
f1 (ppm)

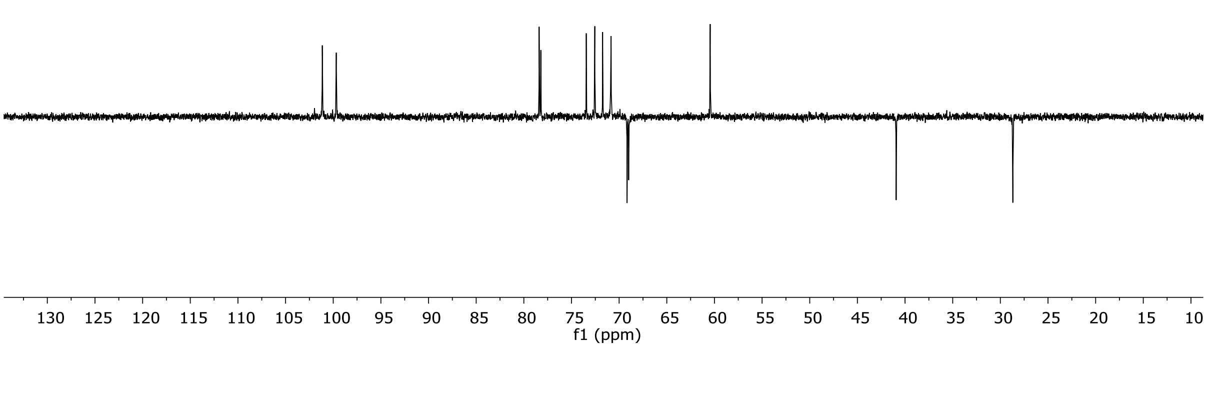
130 125 120 115 110 105 100 95 90 85 80 75 70 65 60 55 50 45 40 35 30 25 20 15 10
f1 (ppm)

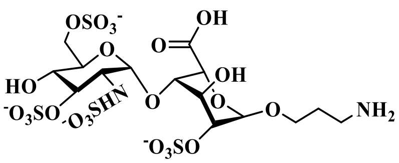
OSO3-
O
OH
O
HO
-O3SO
-O3SHN
O
OH
O
O
NH2
-O3SO

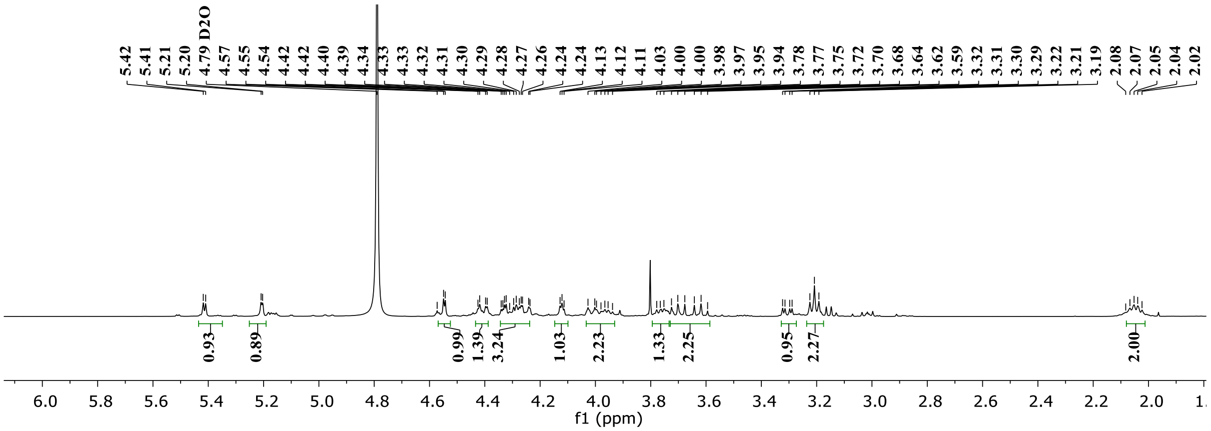
5.42
5.41
5.21
5.20
4.79 D2O
4.57
4.55
4.54
4.42
4.42
4.40
4.39
4.34
4.33
4.33
4.32
4.31
4.30
4.29
4.28
4.27
4.26
4.24
4.24
4.13
4.12
4.11
4.03
4.00
4.00
3.98
3.97
3.95
3.94
3.78
3.77
3.75
3.72
3.70
3.68
3.64
3.62
3.59
3.32
3.31
3.30
3.29
3.22
3.21
3.19
2.08
2.07
2.05
2.04
2.02
0.93
0.89
0.99
1.39
3.24
1.03
2.23
1.33
2.25
0.95
2.27
2.00
6.0 5.8 5.6 5.4 5.2 5.0 4.8 4.6 4.4 4.2 4.0 3.8 3.6 3.4 3.2 3.0 2.8 2.6 2.4 2.2 2.0
f1 (ppm)

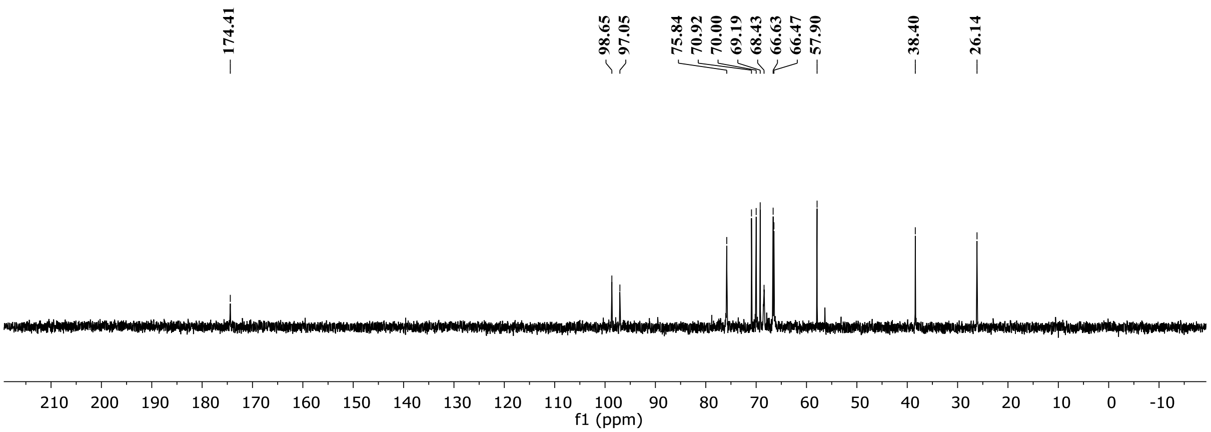
174.41
98.65
97.05
75.84
70.92
70.00
69.19
68.43
66.63
66.47
57.90
38.40
26.14
210 200 190 180 170 160 150 140 130 120 110 100 90 80 70 60 50 40 30 20 10 0 -10
f1 (ppm)

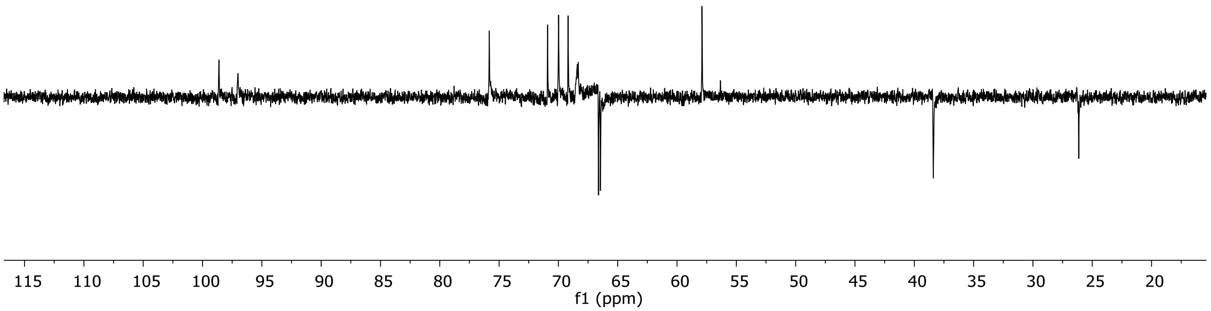
115 110 105 100 95 90 85 80 75 70 65 60 55 50 45 40 35 30 25 20
f1 (ppm)

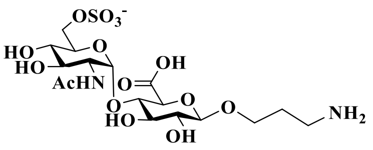

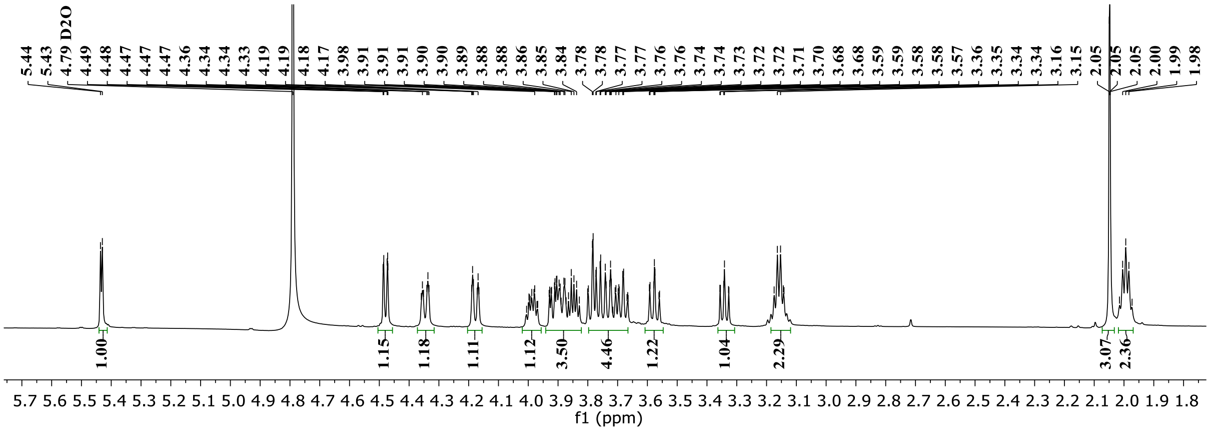
D2O
1.00
1.15
1.18
1.11
1.12
3.50
4.46
1.22
1.04
2.29
3.07
2.36
f1 (ppm)

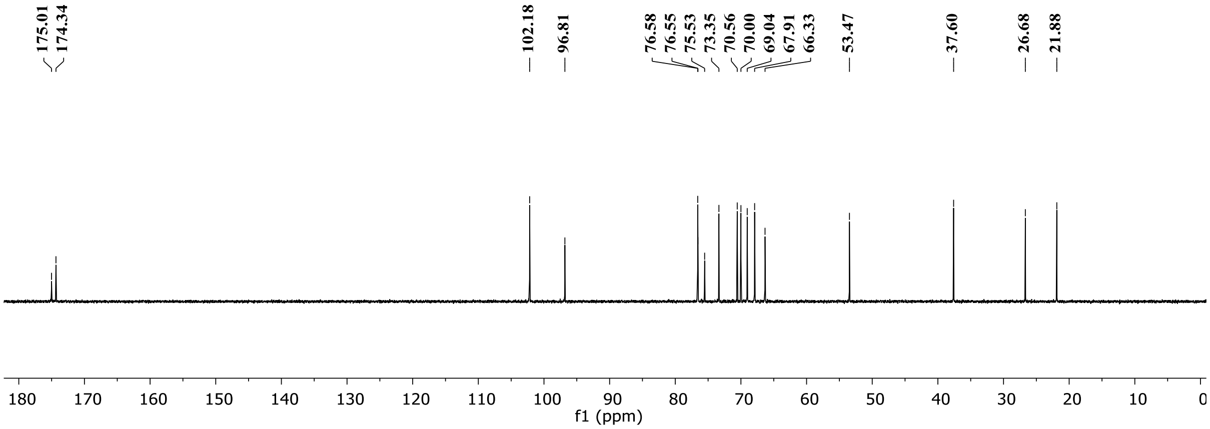
175.01
174.34
102.18
96.81
76.58
76.55
75.53
73.35
70.56
70.00
69.04
67.91
66.33
53.47
37.60
26.68
21.88
f1 (ppm)

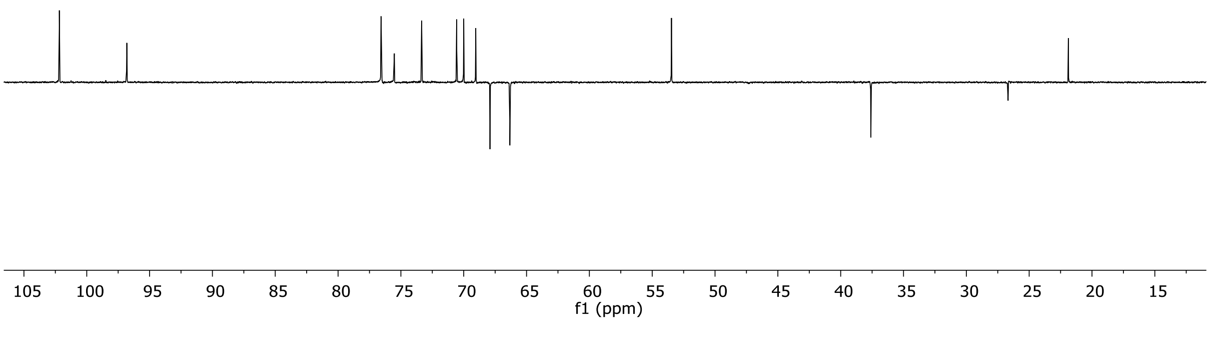
f1 (ppm)

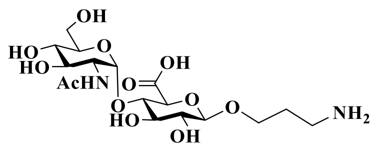
OH
HO
HO
AcHN
O
OH
O
HO
OH
O
NH2

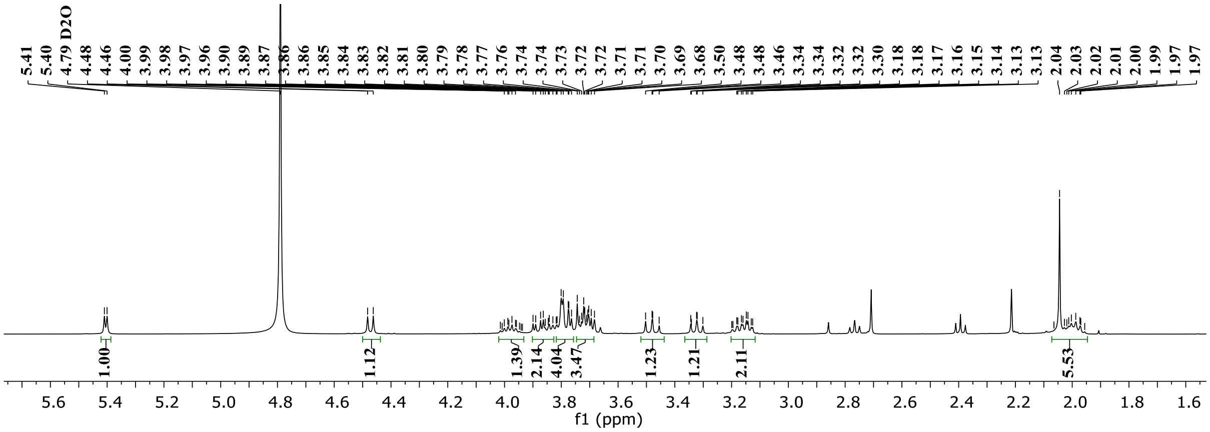
5.41
5.40
4.79 D2O
4.48
4.46
4.00
3.99
3.98
3.97
3.96
3.90
3.89
3.87
3.86
3.86
3.85
3.84
3.83
3.82
3.81
3.80
3.79
3.78
3.77
3.76
3.74
3.74
3.73
3.72
3.72
3.72
3.71
3.71
3.70
3.69
3.68
3.50
3.48
3.48
3.46
3.34
3.34
3.32
3.32
3.30
3.18
3.18
3.17
3.16
3.15
3.14
3.13
3.13
2.04
2.03
2.02
2.01
2.00
1.99
1.97
1.97
1.00
1.12
1.39
2.14
4.04
3.47
1.23
1.21
2.11
5.53
5.6
5.4
5.2
5.0
4.8
4.6
4.4
4.2
4.0
3.8
3.6
3.4
3.2
3.0
2.8
2.6
2.4
2.2
2.0
1.8
1.6
f1 (ppm)

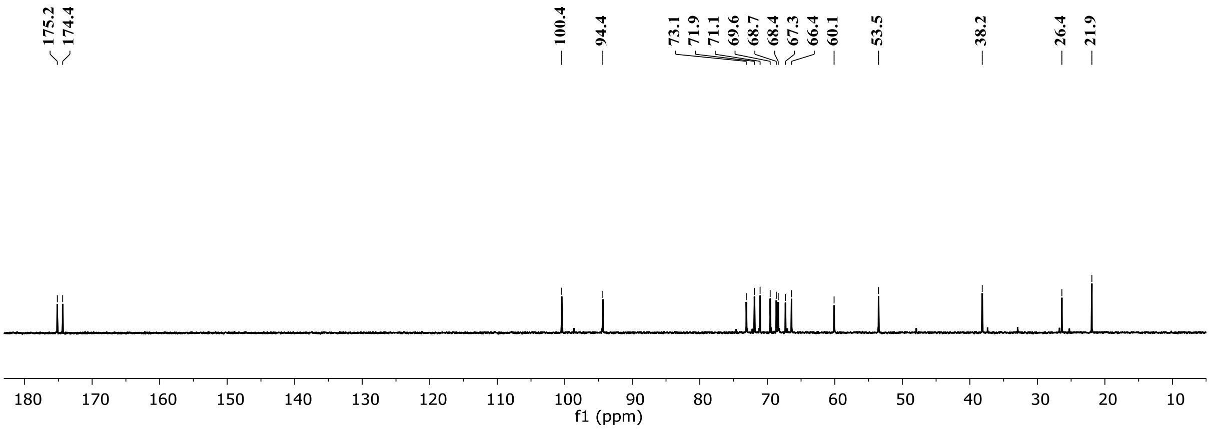
175.2
174.4
100.4
94.4
73.1
71.9
71.1
69.6
68.7
68.4
67.3
66.4
60.1
53.5
38.2
26.4
21.9
180
170
160
150
140
130
120
110
100
90
80
70
60
50
40
30
20
10
f1 (ppm)

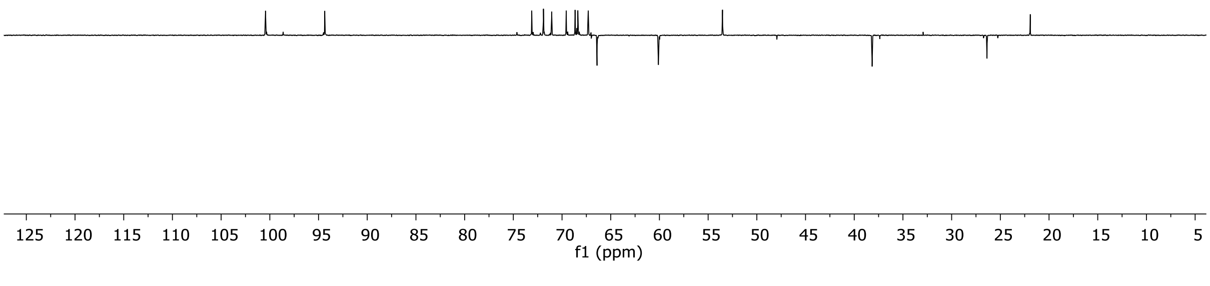
125
120
115
110
105
100
95
90
85
80
75
70
65
60
55
50
45
40
35
30
25
20
15
10
5
f1 (ppm)

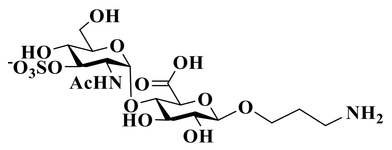

OH
HO
$^{-}O_3SO$
O
AcHN
O
O
OH
O
O
O
HO
OH
NH_2

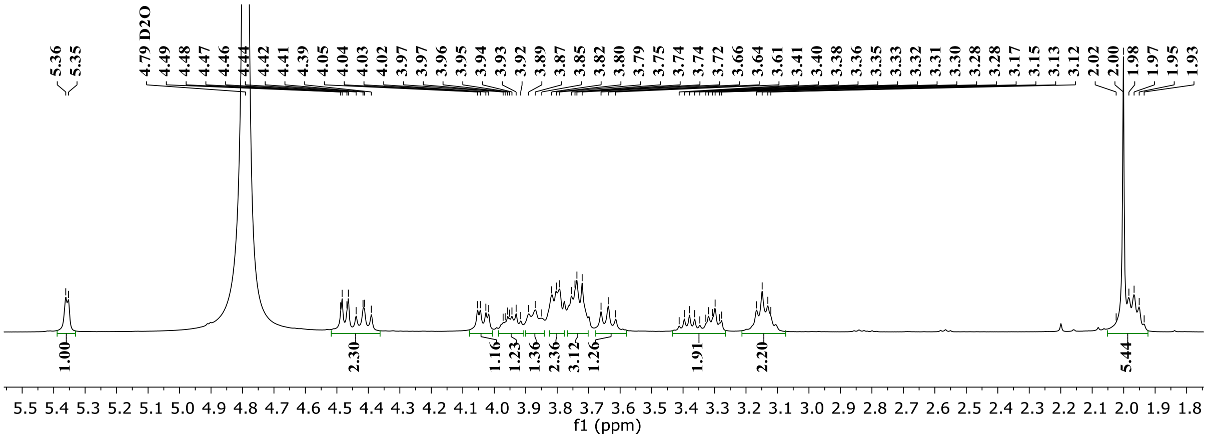

5.36
5.35
4.79 D2O
4.49
4.48
4.47
4.46
4.44
4.42
4.41
4.39
4.05
4.04
4.03
4.02
3.97
3.97
3.96
3.95
3.94
3.93
3.92
3.89
3.87
3.85
3.82
3.80
3.79
3.75
3.74
3.74
3.72
3.66
3.64
3.61
3.41
3.40
3.38
3.36
3.35
3.33
3.32
3.31
3.30
3.28
3.28
3.17
3.15
3.13
3.12
2.02
2.00
1.98
1.97
1.95
1.93
1.00
2.30
1.16
1.23
1.36
2.36
3.12
1.26
1.91
2.20
5.44
5.5 5.4 5.3 5.2 5.1 5.0 4.9 4.8 4.7 4.6 4.5 4.4 4.3 4.2 4.1 4.0 3.9 3.8 3.7 3.6 3.5 3.4 3.3 3.2 3.1 3.0 2.9 2.8 2.7 2.6 2.5 2.4 2.3 2.2 2.1 2.0 1.9 1.8
f1 (ppm)

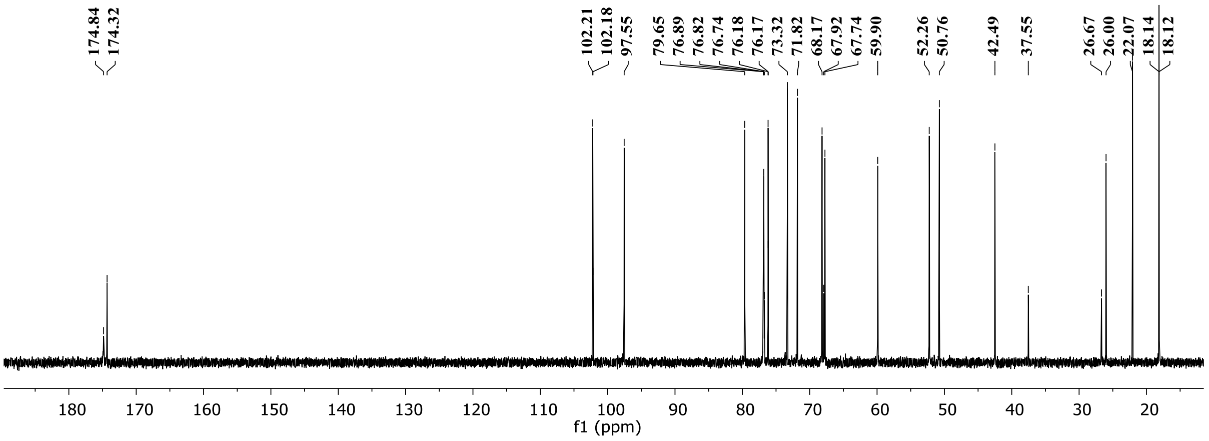

174.84
174.32
102.21
102.18
97.55
79.65
76.89
76.82
76.74
76.18
76.17
73.32
71.82
68.17
67.92
67.74
59.90
52.26
50.76
42.49
37.55
26.67
26.00
22.07
18.14
18.12
180 170 160 150 140 130 120 110 100 90 80 70 60 50 40 30 20
f1 (ppm)

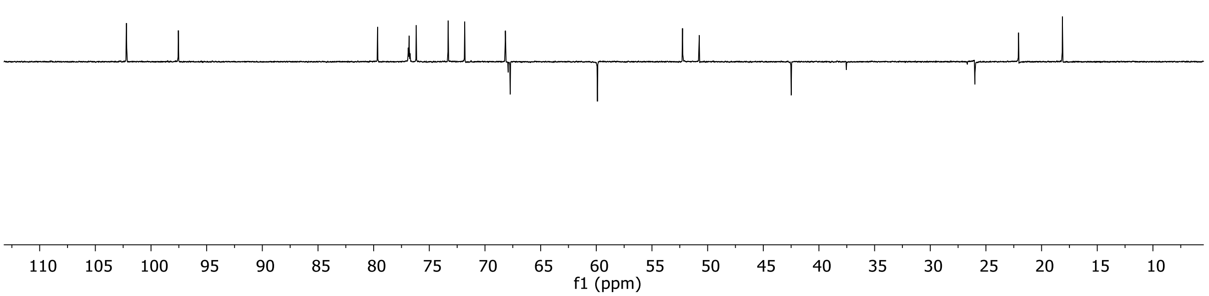

110 105 100 95 90 85 80 75 70 65 60 55 50 45 40 35 30 25 20 15 10
f1 (ppm)

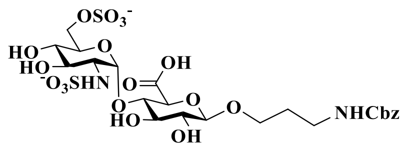
OSO3-
HO
HO
-O3SHN
O
OH
O
HO
OH
NHCbz

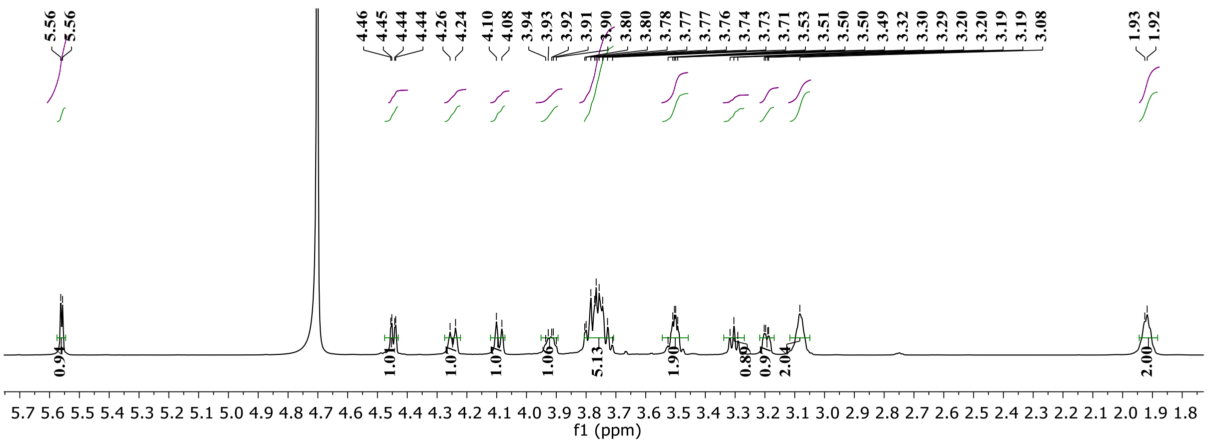
5.56 5.56
4.46 4.45 4.44 4.44 4.26 4.24 4.10 4.08 3.94 3.93 3.92 3.91 3.90 3.80 3.80 3.78 3.77 3.77 3.76 3.74 3.73 3.71 3.53 3.51 3.50 3.50 3.49 3.32 3.30 3.29 3.20 3.20 3.19 3.08
1.93 1.92
0.94 1.01 1.07 1.07 1.06 5.13 1.90 0.80 0.97 2.04 2.00
5.7 5.6 5.5 5.4 5.3 5.2 5.1 5.0 4.9 4.8 4.7 4.6 4.5 4.4 4.3 4.2 4.1 4.0 3.9 3.8 3.7 3.6 3.5 3.4 3.3 3.2 3.1 3.0 2.9 2.8 2.7 2.6 2.5 2.4 2.3 2.2 2.1 2.0 1.9 1.8
f1 (ppm)

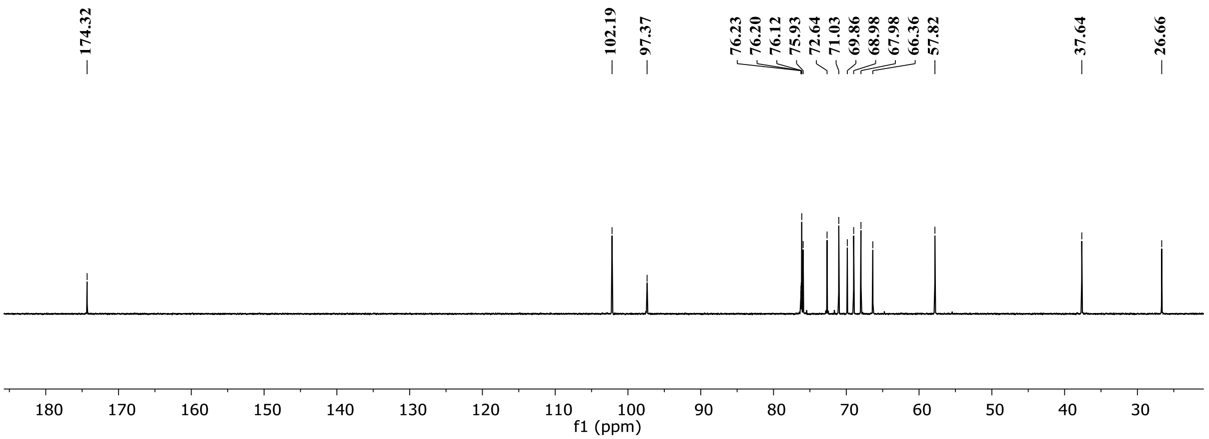
174.32
102.19
97.37
76.23 76.20 76.12 75.93 72.64 71.03 69.86 68.98 67.98 66.36 57.82
37.64
26.66
180 170 160 150 140 130 120 110 100 90 80 70 60 50 40 30
f1 (ppm)

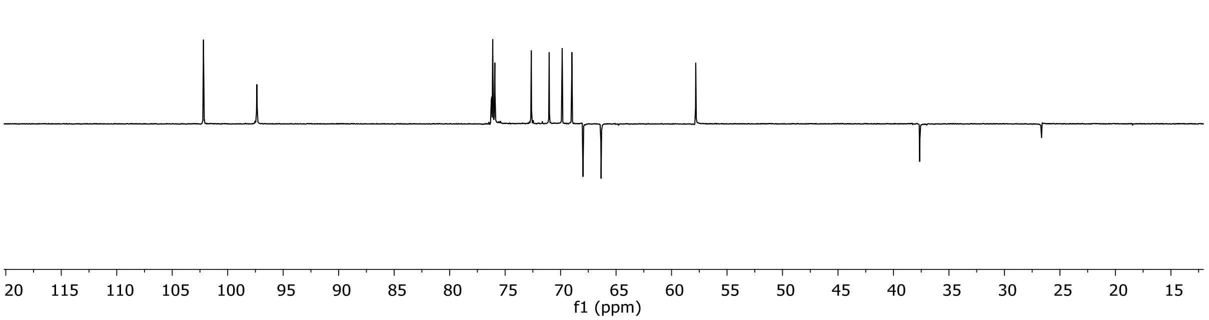
20 115 110 105 100 95 90 85 80 75 70 65 60 55 50 45 40 35 30 25 20 15
f1 (ppm)

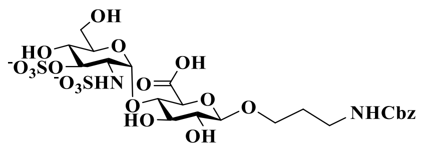
OH
HO
-O3SO
-O3SHN
O
OH
O
NHCbz
HO
OH

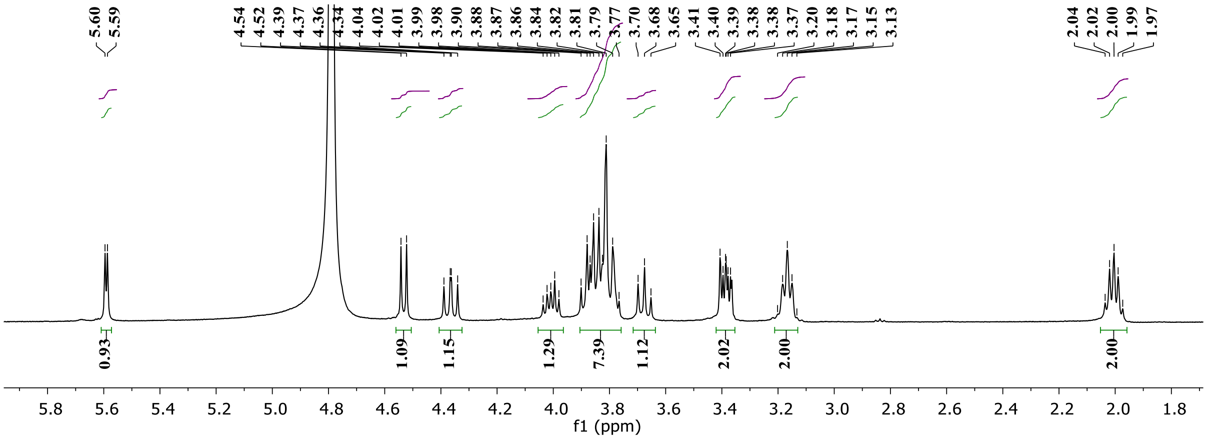
5.60
5.59
4.54
4.52
4.39
4.37
4.36
4.34
4.04
4.02
4.01
3.99
3.98
3.90
3.88
3.87
3.86
3.84
3.82
3.81
3.79
3.77
3.70
3.68
3.65
3.41
3.40
3.39
3.38
3.38
3.37
3.20
3.18
3.17
3.15
3.13
2.04
2.02
2.00
1.99
1.97
0.93
1.09
1.15
1.29
7.39
1.12
2.02
2.00
2.00
f1 (ppm)

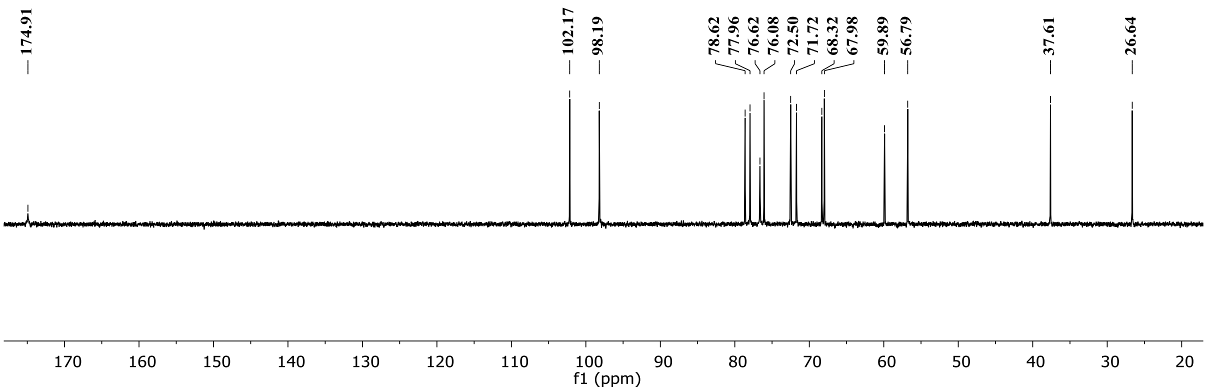
174.91
102.17
98.19
78.62
77.96
76.62
76.08
72.50
71.72
68.32
67.98
59.89
56.79
37.61
26.64
f1 (ppm)

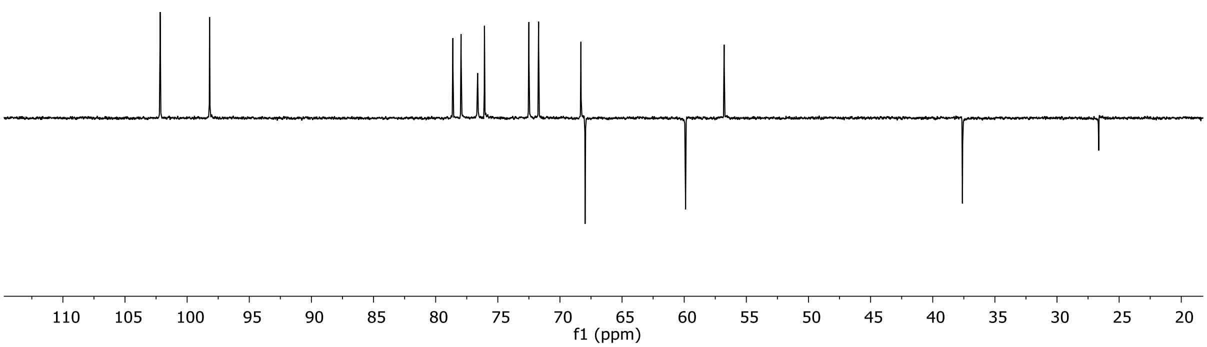
f1 (ppm)

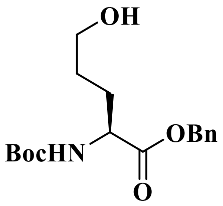
OH
BocHN
OBn
O

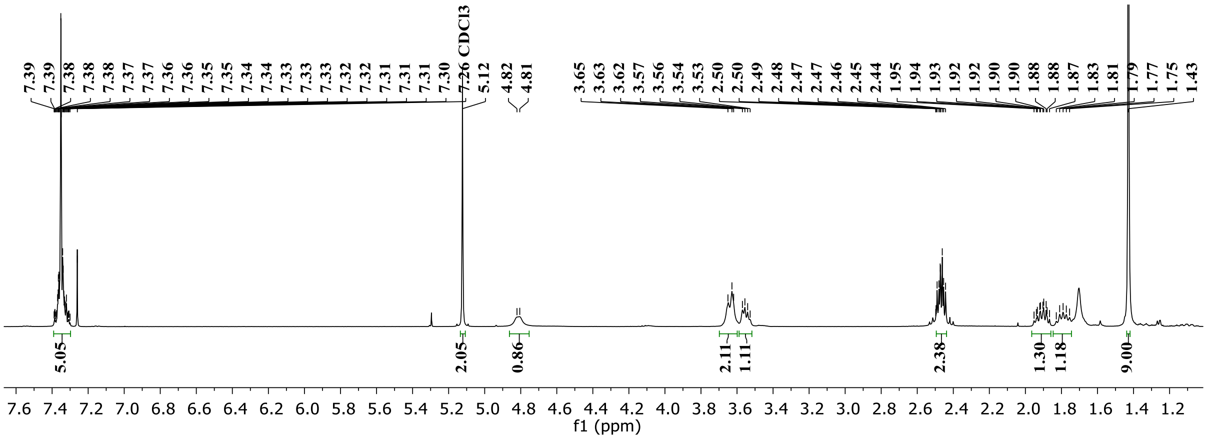
7.39
7.39
7.38
7.38
7.38
7.37
7.37
7.36
7.36
7.35
7.35
7.34
7.34
7.33
7.33
7.33
7.32
7.32
7.31
7.31
7.31
7.30
7.26 CDCl3
5.12
4.82
4.81
3.65
3.63
3.62
3.57
3.56
3.54
3.53
2.50
2.50
2.49
2.48
2.47
2.47
2.46
2.45
2.44
1.95
1.94
1.93
1.92
1.92
1.90
1.90
1.88
1.88
1.87
1.83
1.81
1.79
1.77
1.75
1.43
5.05
2.05
0.86
2.11
1.11
2.38
1.30
1.18
9.00
7.6 7.4 7.2 7.0 6.8 6.6 6.4 6.2 6.0 5.8 5.6 5.4 5.2 5.0 4.8 4.6 4.4 4.2 4.0 3.8 3.6 3.4 3.2 3.0 2.8 2.6 2.4 2.2 2.0 1.8 1.6 1.4 1.2
f1 (ppm)

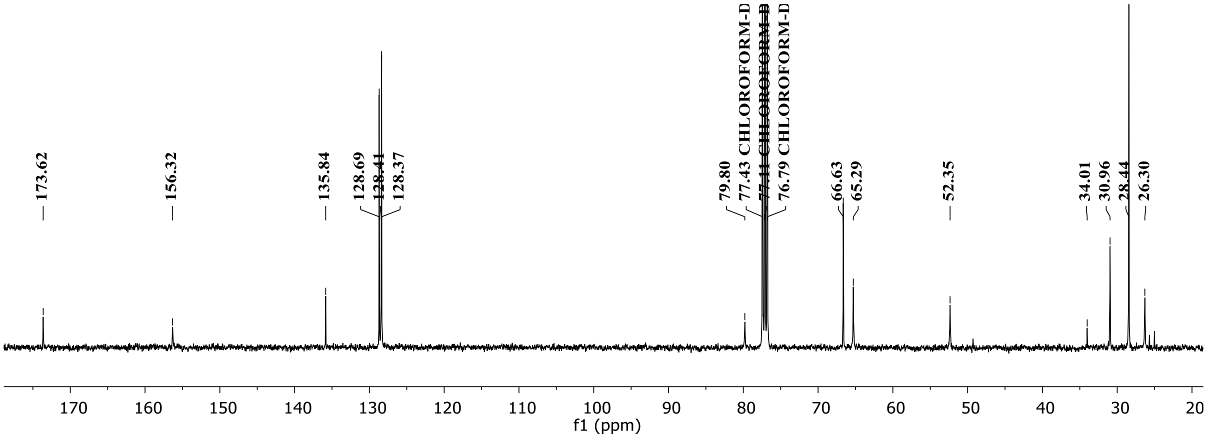
173.62
156.32
135.84
128.69
128.41
128.37
79.80
77.43 CHLOROFORM-D
77.11 CHLOROFORM-D
76.79 CHLOROFORM-D
66.63
65.29
52.35
34.01
30.96
28.44
26.30
170 160 150 140 130 120 110 100 90 80 70 60 50 40 30 20
f1 (ppm)

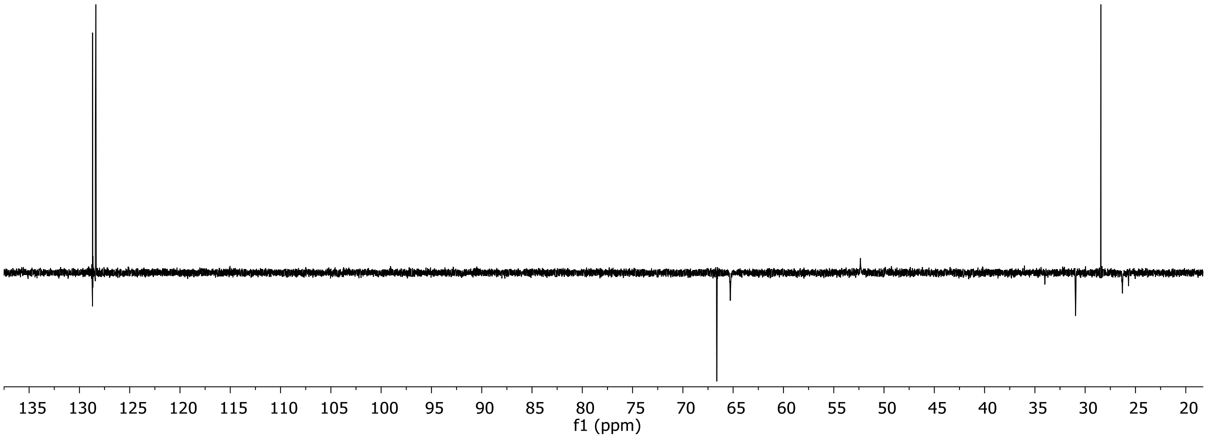
135 130 125 120 115 110 105 100 95 90 85 80 75 70 65 60 55 50 45 40 35 30 25 20
f1 (ppm)

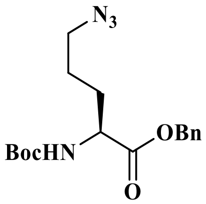
N3
OBn
BocHN
O

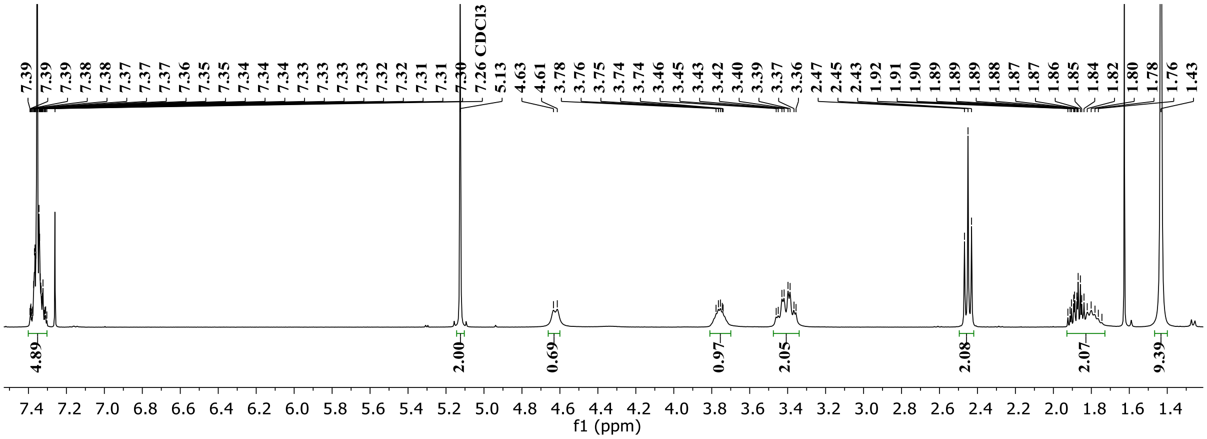
7.39 7.39 7.39 7.38 7.38 7.37 7.37 7.37 7.36 7.35 7.35 7.34 7.34 7.34 7.33 7.33 7.33 7.33 7.32 7.32 7.31 7.31 7.30 7.26 CDCl3 5.13 4.63 4.61 3.78 3.76 3.75 3.74 3.74 3.46 3.45 3.43 3.42 3.40 3.39 3.37 3.36 2.47 2.45 2.43 1.92 1.91 1.90 1.89 1.89 1.89 1.88 1.87 1.87 1.86 1.85 1.84 1.82 1.80 1.78 1.76 1.43
4.89
2.00
0.69
0.97
2.05
2.08
2.07
9.39
7.4 7.2 7.0 6.8 6.6 6.4 6.2 6.0 5.8 5.6 5.4 5.2 5.0 4.8 4.6 4.4 4.2 4.0 3.8 3.6 3.4 3.2 3.0 2.8 2.6 2.4 2.2 2.0 1.8 1.6 1.4
f1 (ppm)

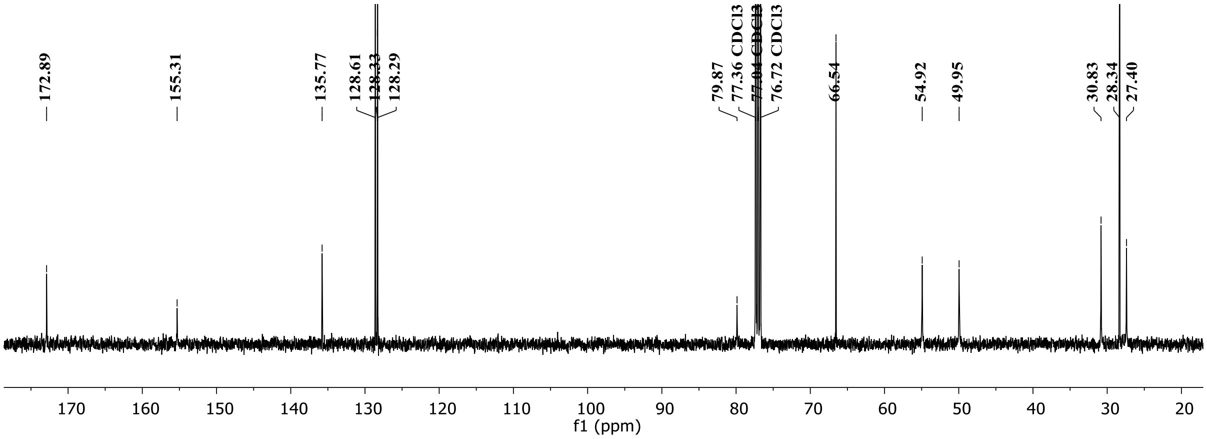
172.89
155.31
135.77
128.61 128.33 128.29
79.87 77.36 CDCl3 77.04 CDCl3 76.72 CDCl3
66.54
54.92
49.95
30.83 28.34 27.40
170 160 150 140 130 120 110 100 90 80 70 60 50 40 30 20
f1 (ppm)

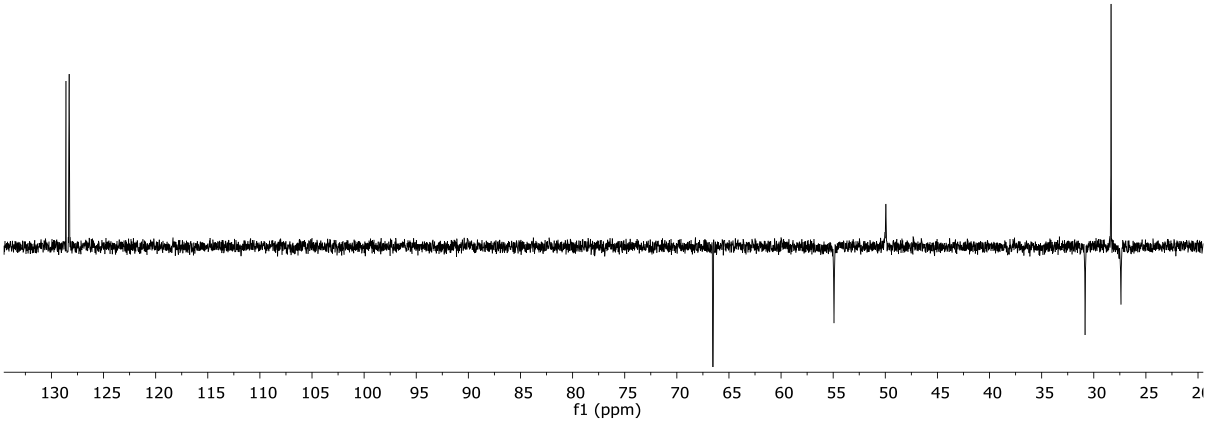
130 125 120 115 110 105 100 95 90 85 80 75 70 65 60 55 50 45 40 35 30 25
f1 (ppm)

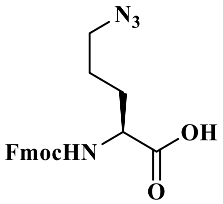
N3
OH
FmocHN
O

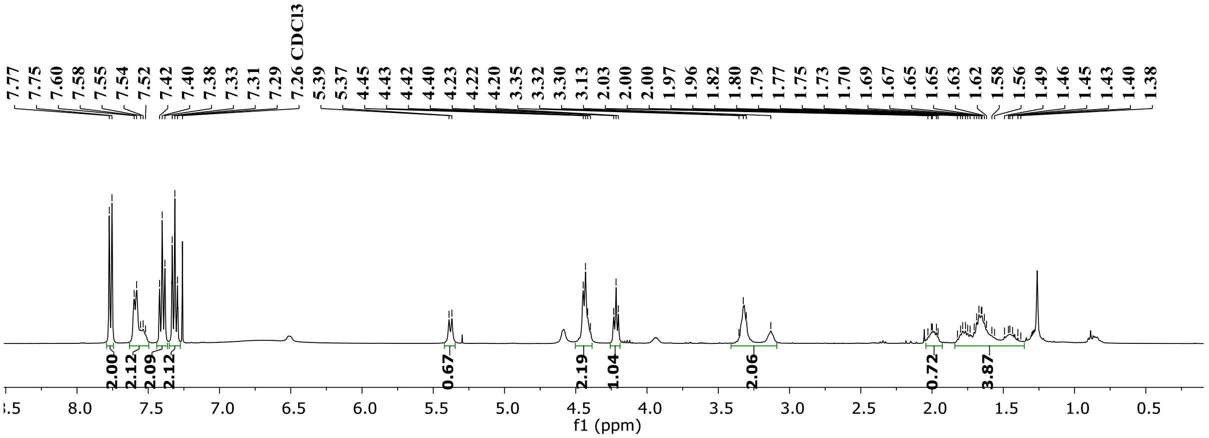

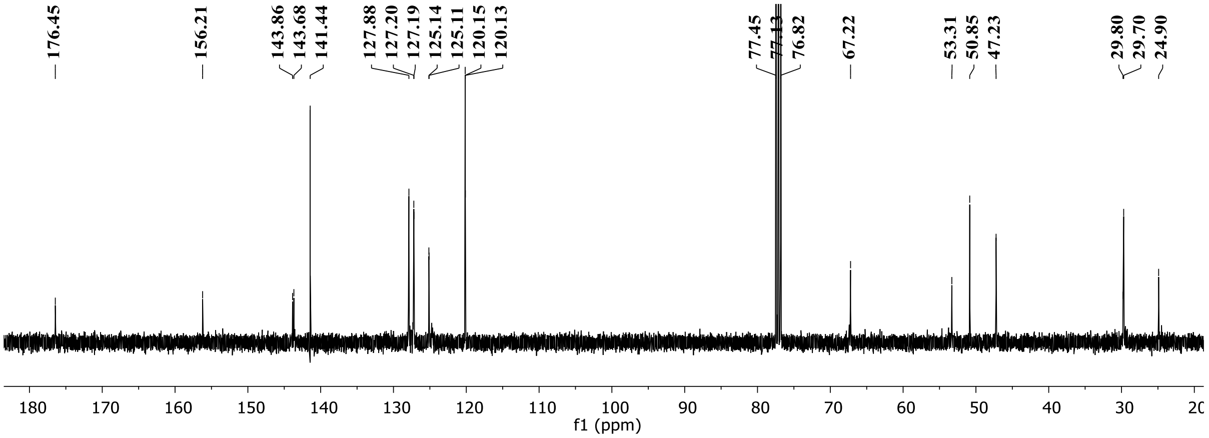

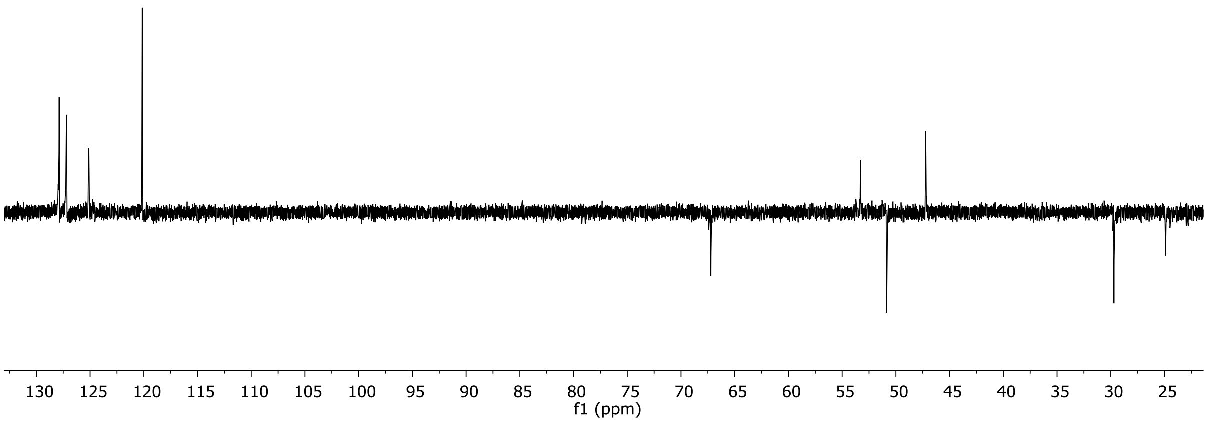

12mer Peptide NMR

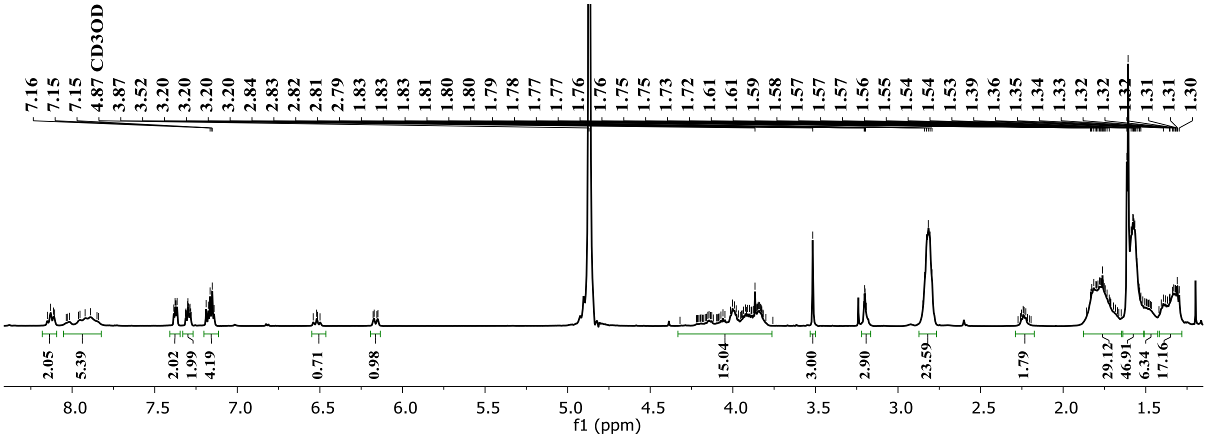

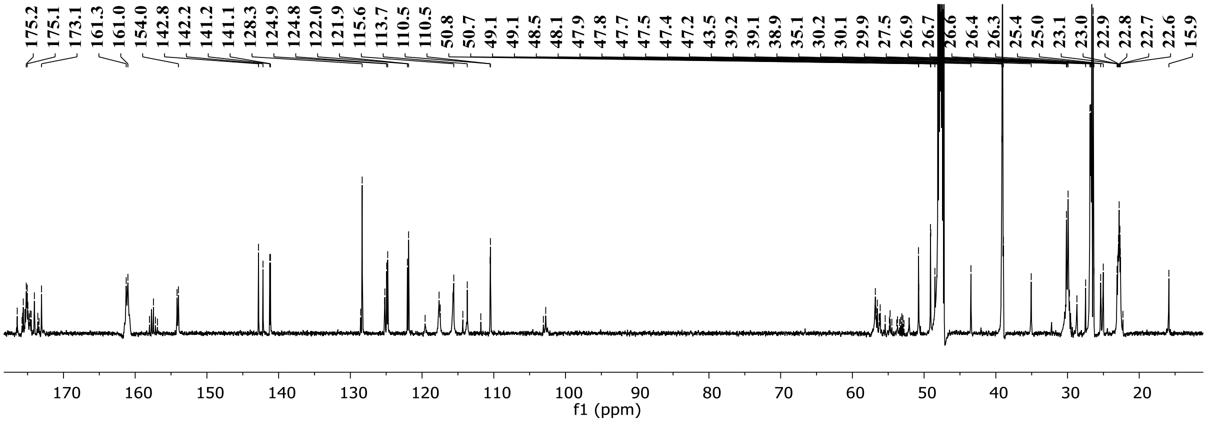

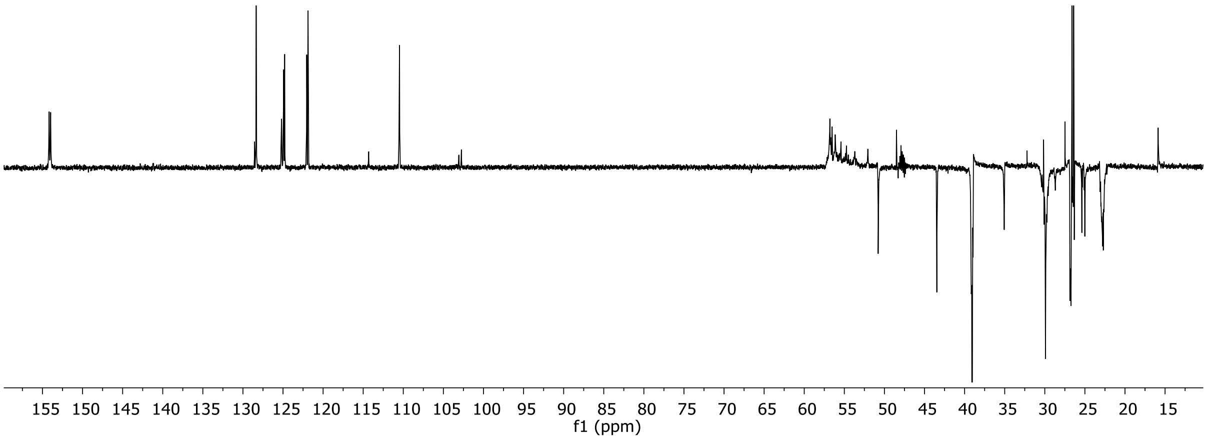

12mer Peptide with Cholesterol

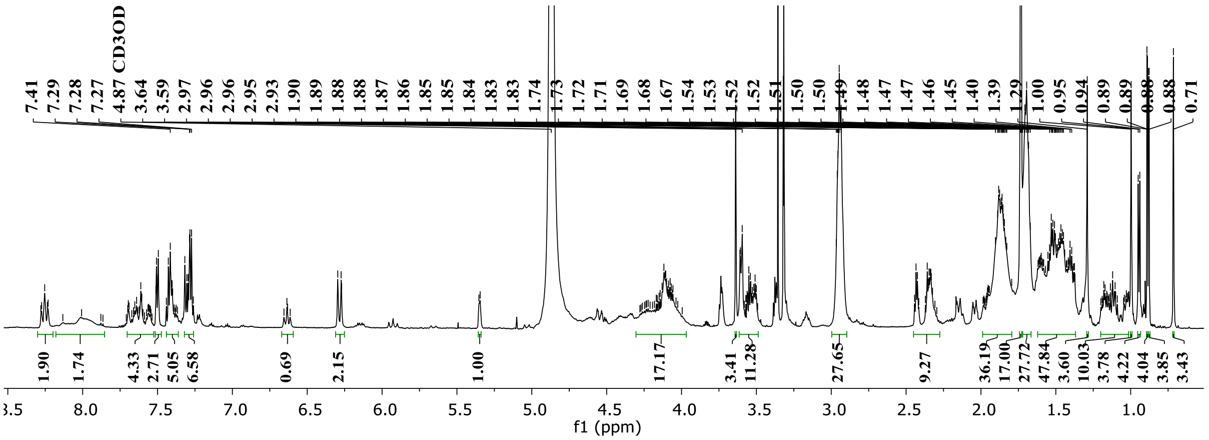

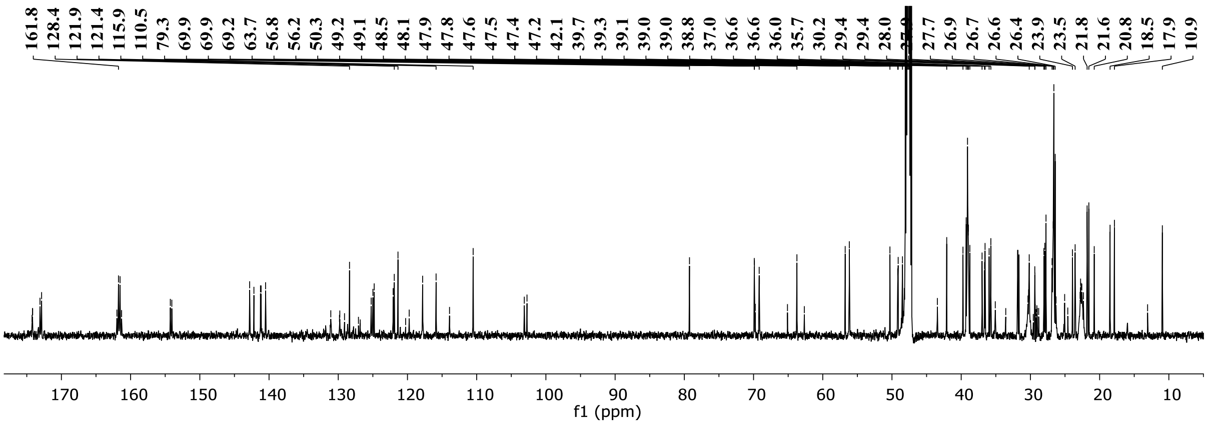

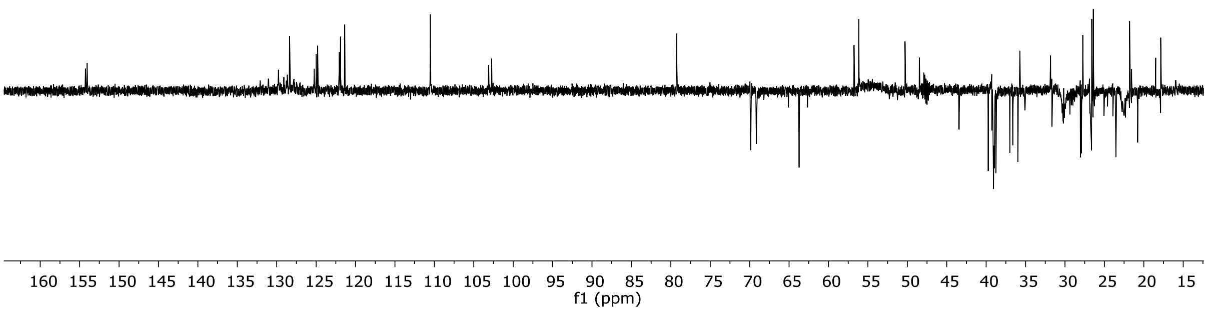

12mer Peptide with 3-Azidopropanoate Linker

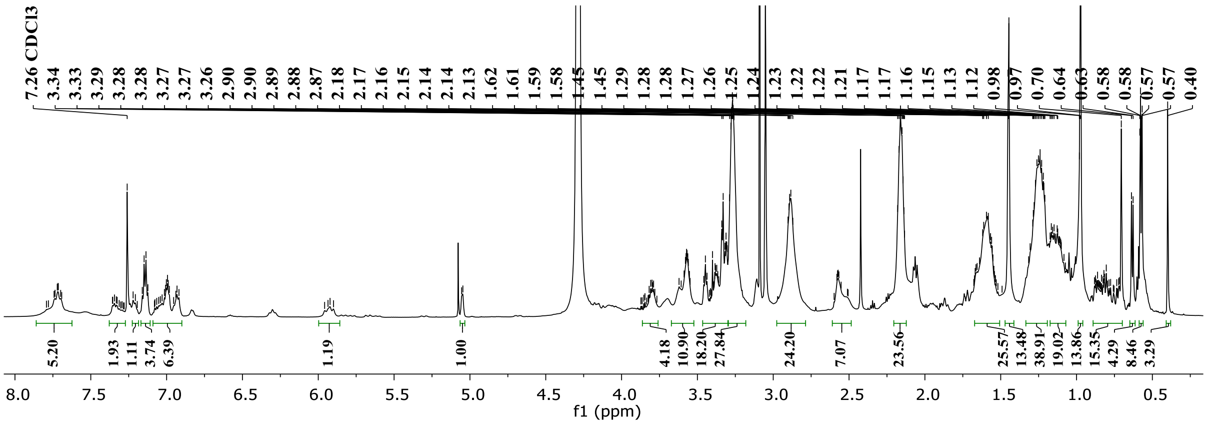

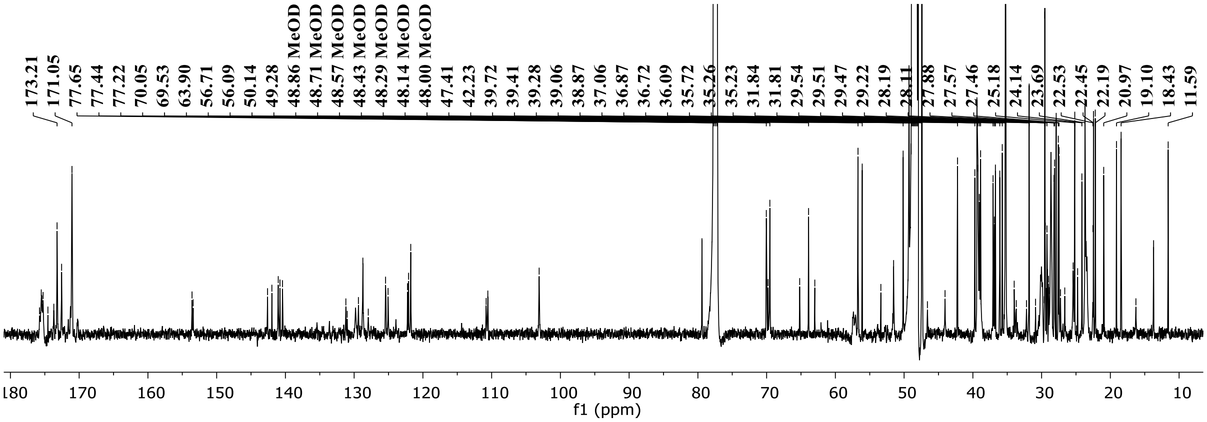

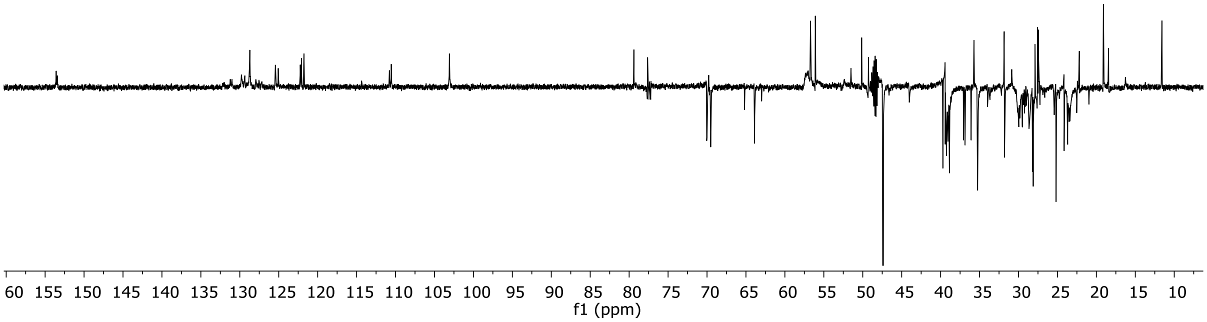

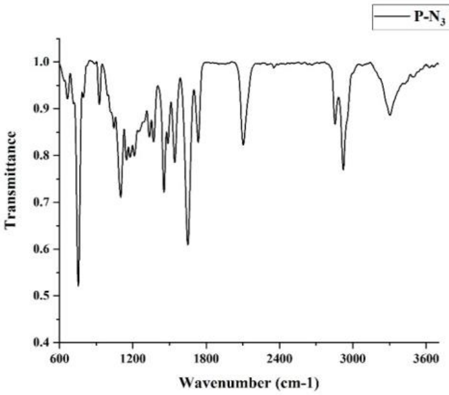
P-N3 Linker
Transmittance
Wavenumber (cm-1)
1.0
0.9
0.8
0.7
0.6
0.5
0.4
600
1200
1800
2400
3000
3600

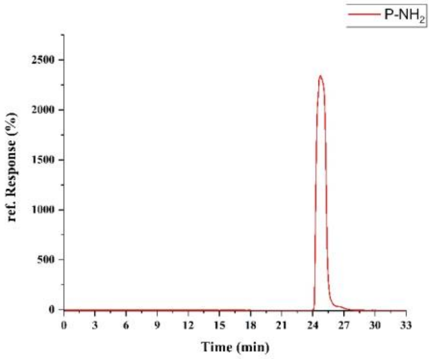
P-NH2
ref. Response (%)
Time (min)
2500
2000
1500
1000
500
0
0
3
6
9
12
15
18
21
24
27
30
33

PG@ID-1

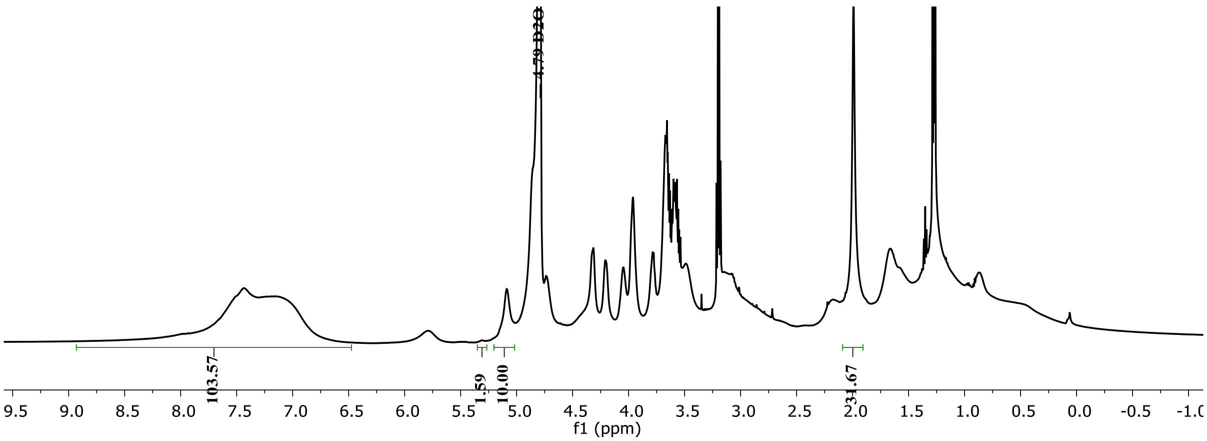

4.79 D2O
103.57
1.59
10.00
31.67
9.5 9.0 8.5 8.0 7.5 7.0 6.5 6.0 5.5 5.0 4.5 4.0 3.5 3.0 2.5 2.0 1.5 1.0 0.5 0.0 -0.5 -1.0
f1 (ppm)

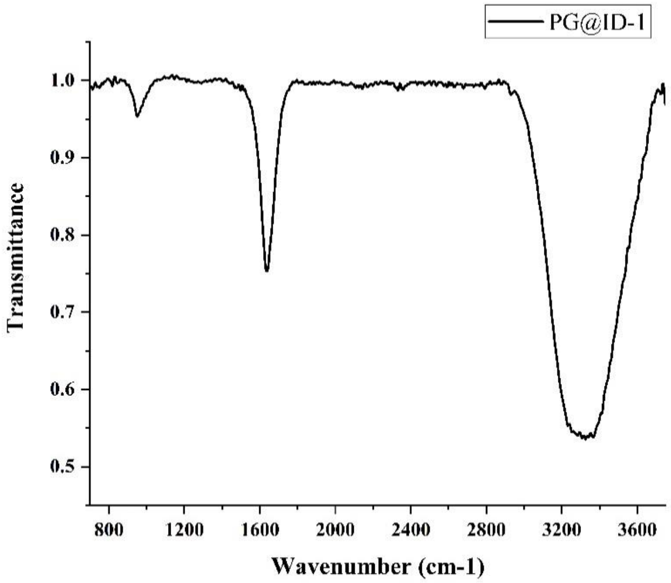

PG@ID-1
Transmittance
1.0
0.9
0.8
0.7
0.6
0.5
800 1200 1600 2000 2400 2800 3200 3600
Wavenumber (cm-1)

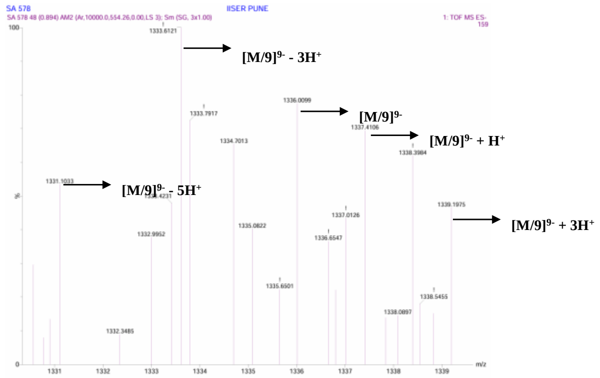

SA 578
IISER PUNE
1: TOF MS ES-
[M/9]9- - 3H+
[M/9]9-
[M/9]9- + H+
[M/9]9- - 5H+
[M/9]9- + 3H+
1333.6121
1336.0099
1331.1033
1339.1975

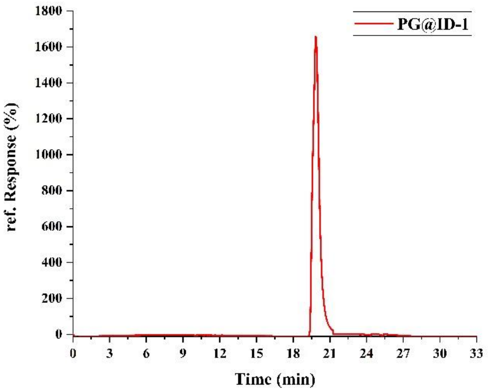

PG@ID-1
ref. Response (%)
Time (min)
0 3 6 9 12 15 18 21 24 27 30 33

PG@ID-2

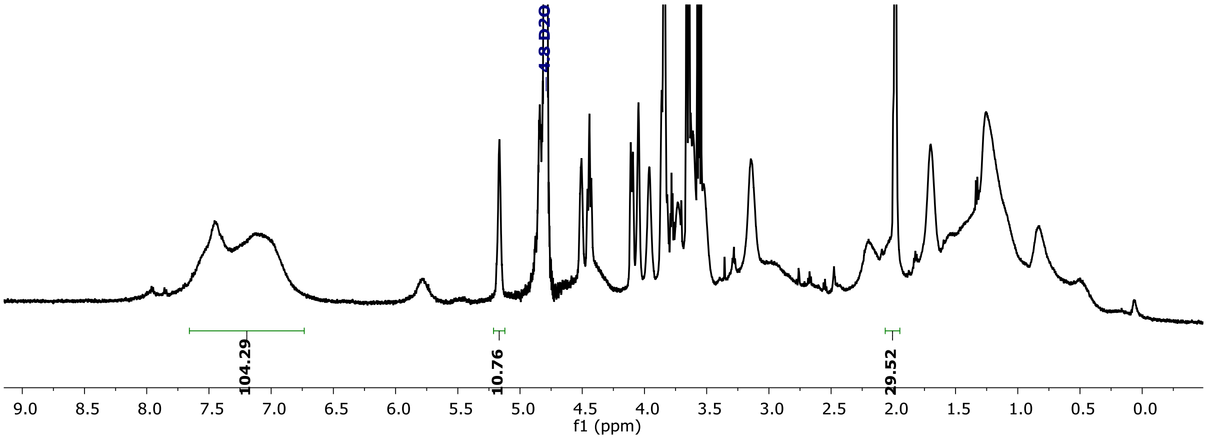

4.8 D2O
104.29
10.76
29.52
9.0 8.5 8.0 7.5 7.0 6.5 6.0 5.5 5.0 4.5 4.0 3.5 3.0 2.5 2.0 1.5 1.0 0.5 0.0
f1 (ppm)

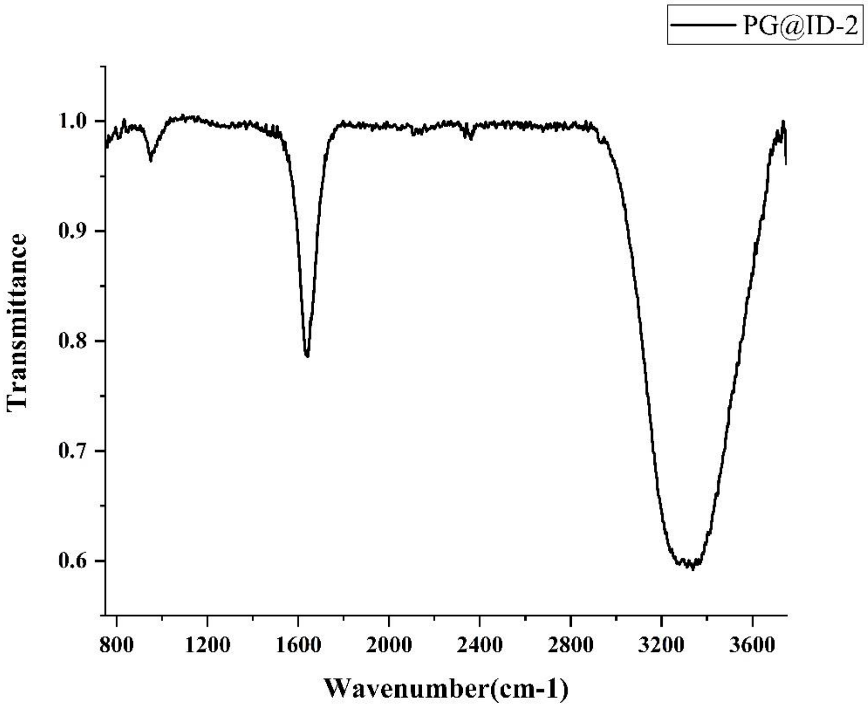

PG@ID-2
Transmittance
1.0
0.9
0.8
0.7
0.6
800 1200 1600 2000 2400 2800 3200 3600
Wavenumber(cm-1)

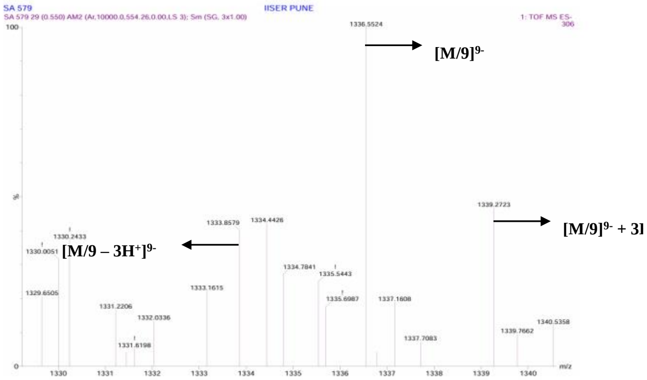
SA 579
IISER PUNE
SA 579 29 (0.550) AM2 (Ar,10000.0,554.26,0.00,LS 3); Sm (SG, 3x1.00)
1: TOF MS ES-
306
100
0
1336.5524
[M/9]9-
1339.2723
[M/9]9- + 3
1333.8579
1334.4426
1330.2433
1330.0051
[M/9 – 3H+]9-
1329.6505
1331.2206
1331.6198
1332.0336
1333.1615
1334.7841
1335.5443
1335.6987
1337.1608
1337.7083
1339.7662
1340.5358
1330
1331
1332
1333
1334
1335
1336
1337
1338
1339
1340
m/z

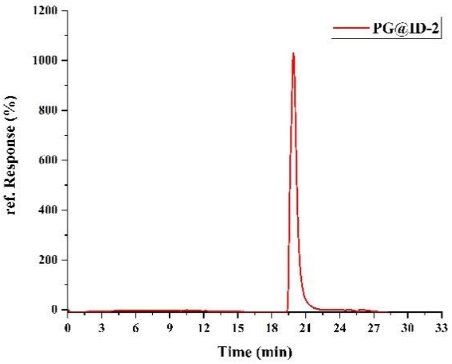
PG@1D-2
ref. Response (%)
Time (min)
0
200
400
600
800
1000
1200
0
3
6
9
12
15
18
21
24
27
30
33

PG@ID-3

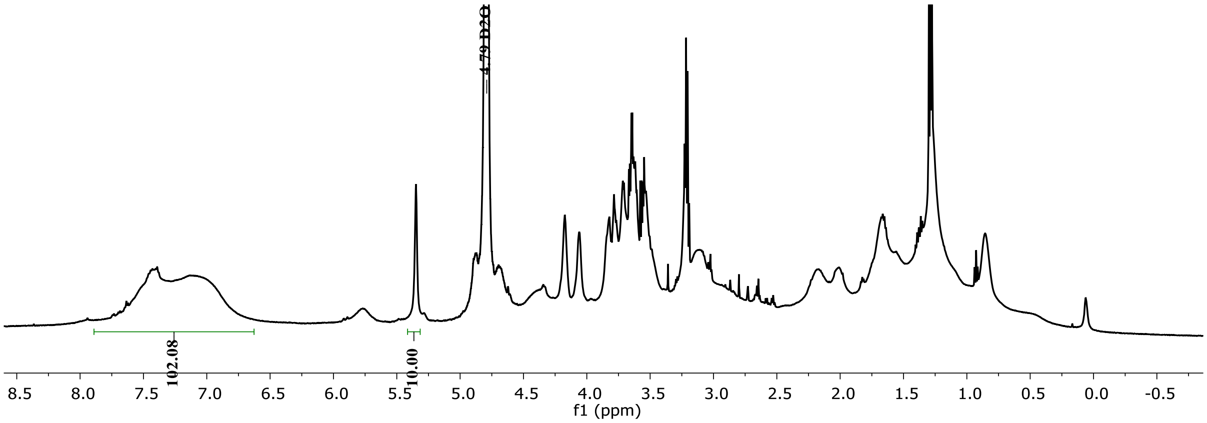

4.79 D2O
102.08
10.00
8.5
8.0
7.5
7.0
6.5
6.0
5.5
5.0
4.5
4.0
3.5
3.0
2.5
2.0
1.5
1.0
0.5
0.0
-0.5
f1 (ppm)

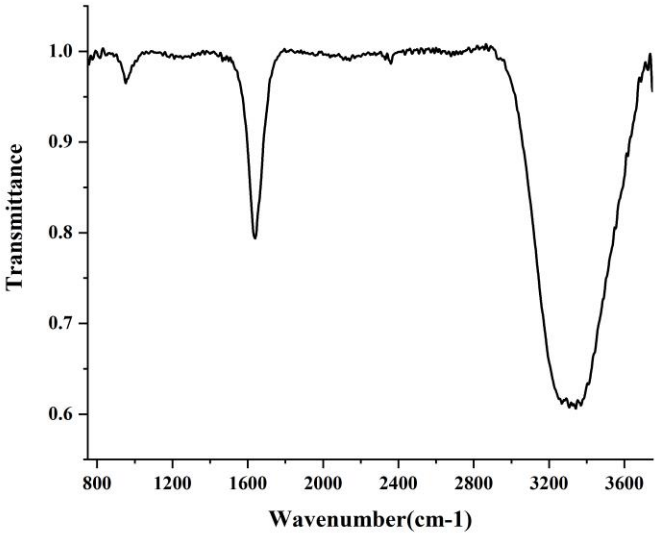

Transmittance
1.0
0.9
0.8
0.7
0.6
800
1200
1600
2000
2400
2800
3200
3600
Wavenumber(cm-1)

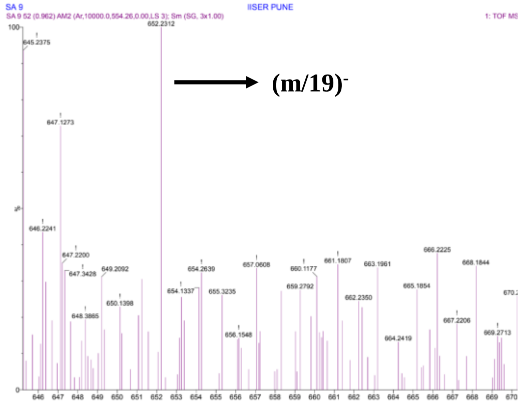
SA 9
IISER PUNE
1: TOF MS
652.2312
645.2375
647.1273
646.2241
647.2200
647.3428
648.3865
649.2092
650.1398
654.1337
654.2639
655.3235
656.1548
657.0608
659.2792
660.1177
661.1807
662.2350
663.1961
664.2419
665.1854
666.2225
667.2206
668.1844
669.2713
(m/19)-

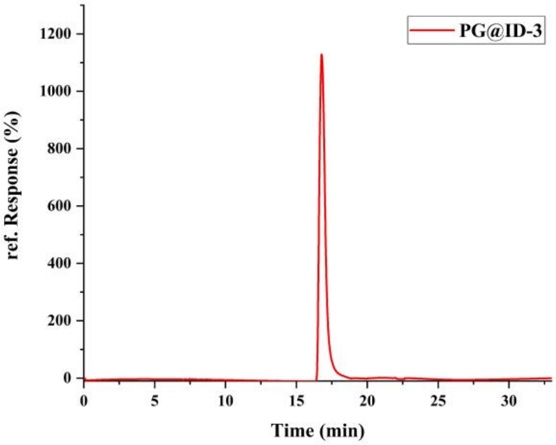
PG@ID-3
ref. Response (%)
Time (min)

PG@ID-4

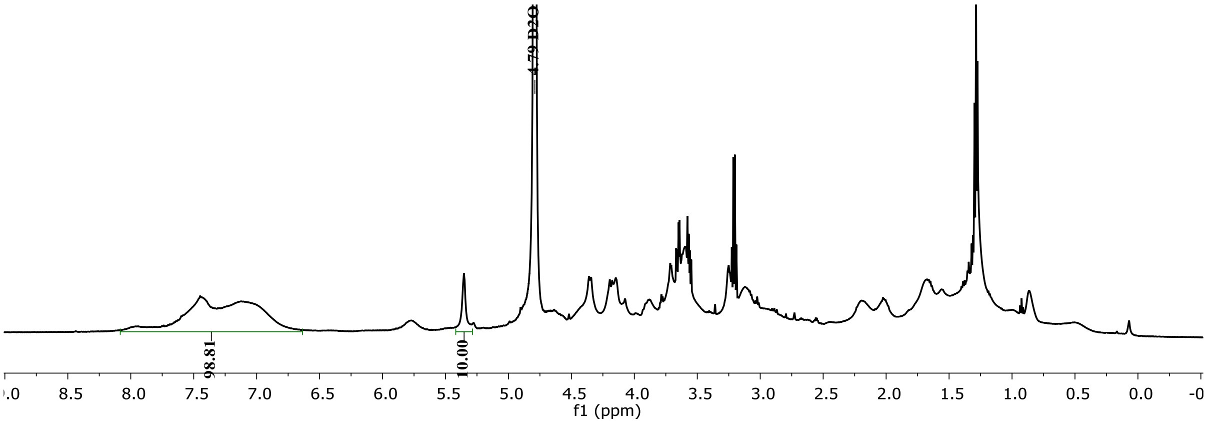

4.79 D2O
98.81
10.00
f1 (ppm)

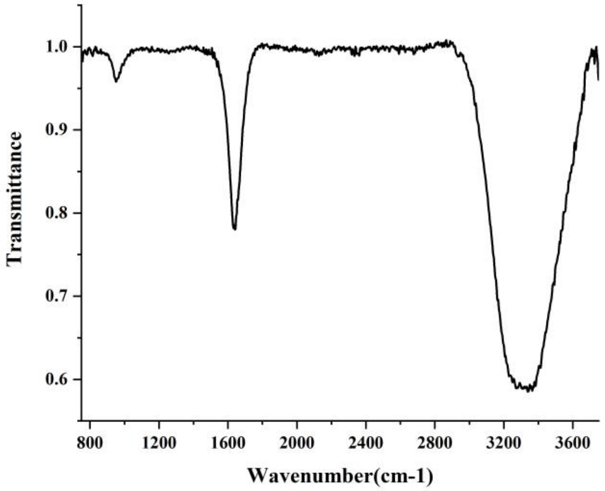

Transmittance
Wavenumber(cm-1)

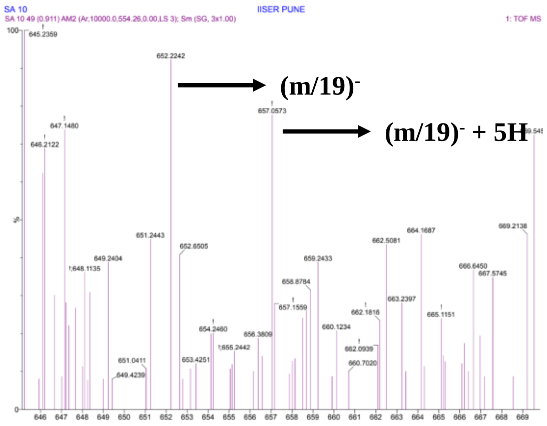

SA 10
IISER PUNE
1: TOF MS
645.2359
652.2242
657.0573
(m/19)-
(m/19)- + 5H
647.1480
646.2122
651.2443
652.6505
649.2404
648.1135
664.1687
662.5081
669.2138
666.6450
667.5745
659.2433
658.8784
657.1559
663.2397
662.1816
665.1151
660.1234
654.2460
656.3809
655.2442
662.0939
660.7020
653.4251
651.0411
649.4239

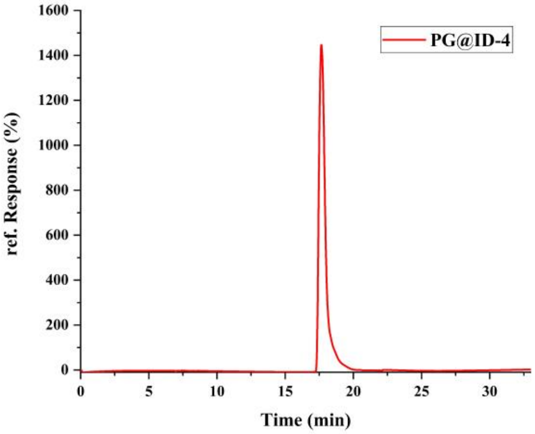

PG@ID-4
ref. Response (%)
Time (min)

PG@ID-5

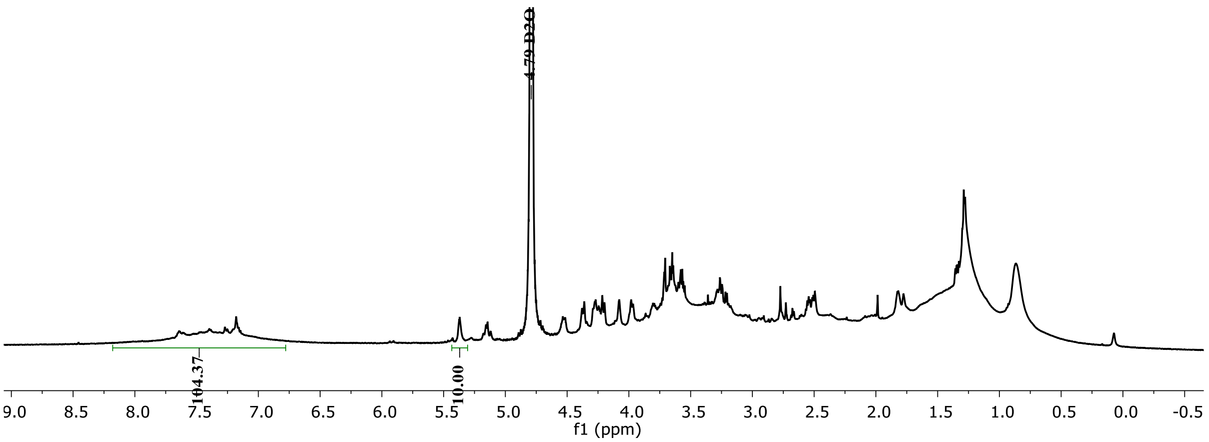
4.79 D2O
104.37
10.00
9.0
8.5
8.0
7.5
7.0
6.5
6.0
5.5
5.0
4.5
4.0
3.5
3.0
2.5
2.0
1.5
1.0
0.5
0.0
-0.5
f1 (ppm)

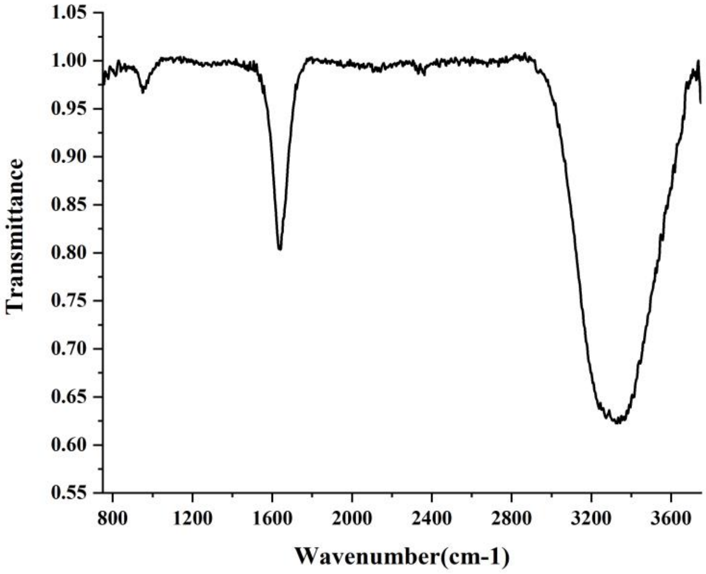
Transmittance
1.05
1.00
0.95
0.90
0.85
0.80
0.75
0.70
0.65
0.60
0.55
800
1200
1600
2000
2400
2800
3200
3600
Wavenumber(cm-1)

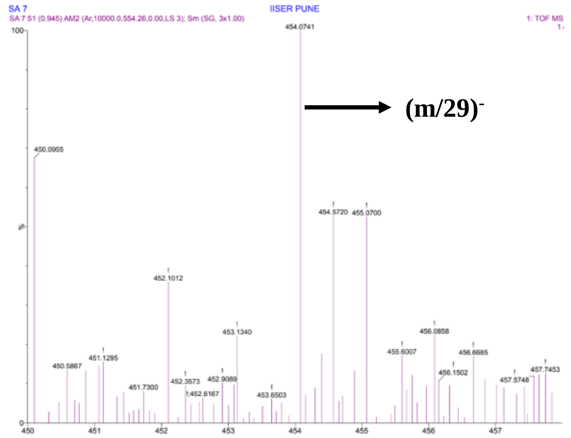
SA 7
SA 7 51 (0.945) AM2 (Ar,10000.0,554.26,0.00,LS 3); Sm (SG, 3x1.00)
IISER PUNE
1: TOF MS
454.0741
(m/29)-
450.0955
454.5720
455.0700
452.1012
453.1340
456.0858
455.6007
456.6685
451.1295
450.5867
456.1502
457.7453
457.5748
452.3573
452.9089
451.7300
452.6167
453.6503
100
%
0
450
451
452
453
454
455
456
457

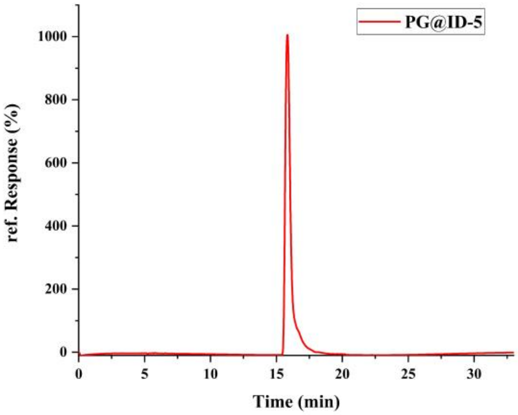
PG@ID-5
ref. Response (%)
Time (min)
0
200
400
600
800
1000
0
5
10
15
20
25
30

PG@ID-6

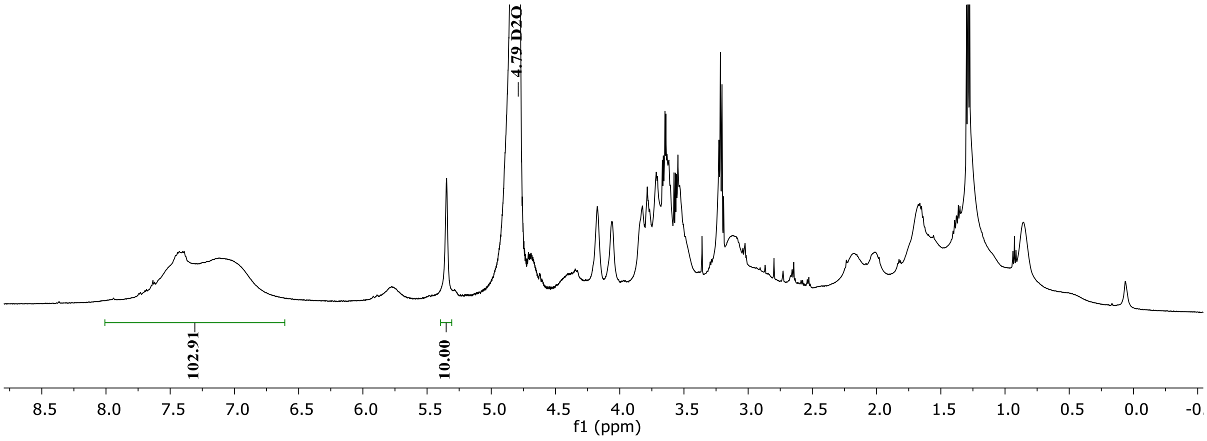

4.79 D2O
102.91
10.00
8.5
8.0
7.5
7.0
6.5
6.0
5.5
5.0
4.5
4.0
3.5
3.0
2.5
2.0
1.5
1.0
0.5
0.0
f1 (ppm)

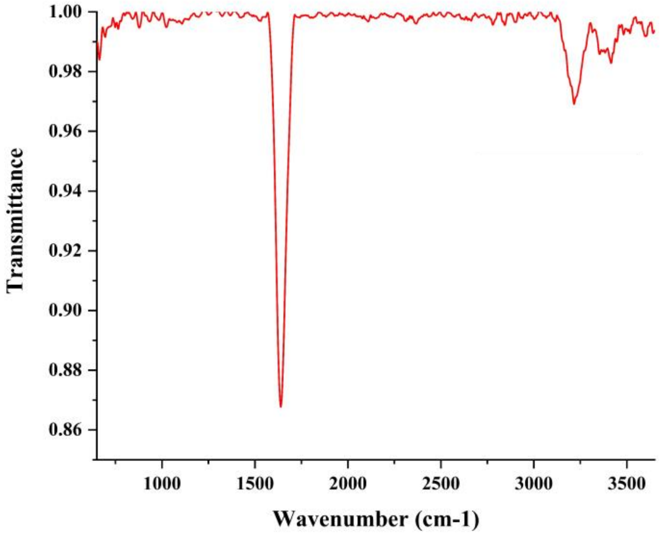

Transmittance
1.00
0.98
0.96
0.94
0.92
0.90
0.88
0.86
1000
1500
2000
2500
3000
3500
Wavenumber (cm-1)

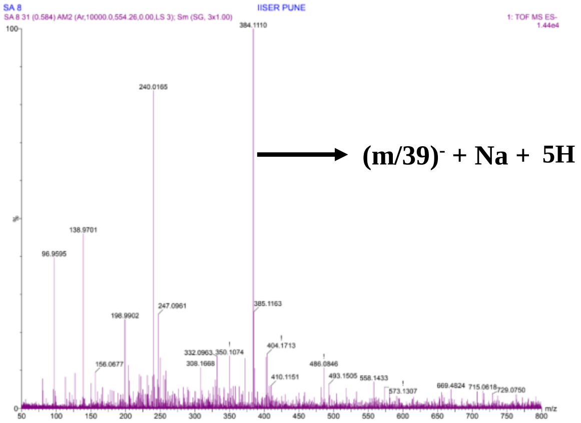
SA 8
IISER PUNE
SA 8 31 (0.584) AM2 (Ar,10000.0,554.26,0.00,LS 3); Sm (SG, 3x1.00)
1: TOF MS ES-
1.44e4
384.1110
240.0165
138.9701
96.9595
198.9902
247.0961
385.1163
156.0677
332.0963
350.1074
308.1668
404.1713
410.1151
486.0846
493.1505
558.1433
573.1307
669.4824
715.0618
729.0750
m/z
(m/39)- + Na + 5H

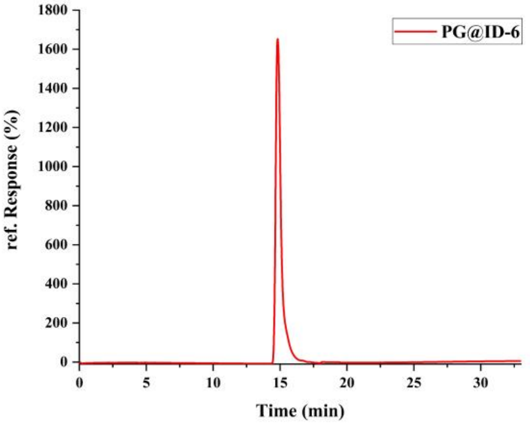
PG@ID-6
ref. Response (%)
Time (min)

PG@GD-1

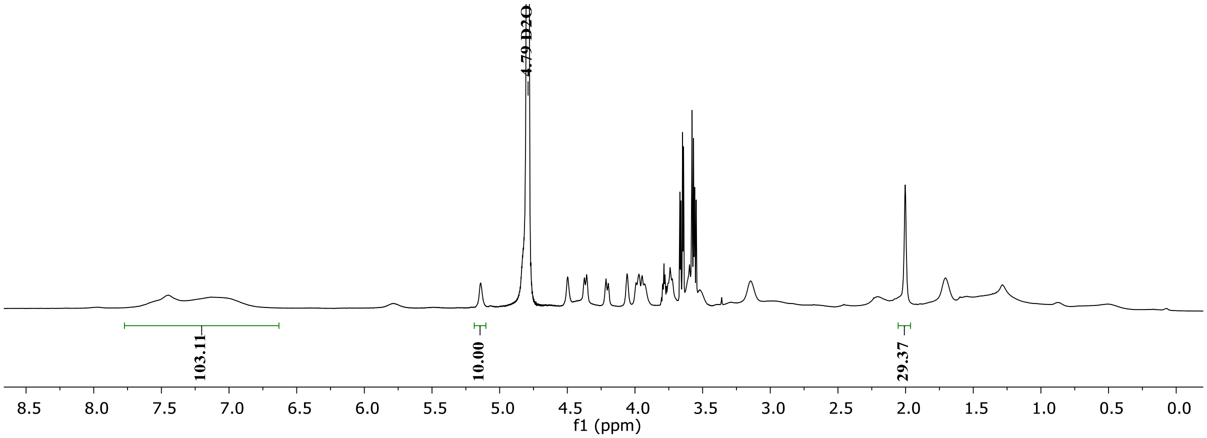
4.79 D2O
103.11
10.00
29.37
8.5
8.0
7.5
7.0
6.5
6.0
5.5
5.0
4.5
4.0
3.5
3.0
2.5
2.0
1.5
1.0
0.5
0.0
f1 (ppm)

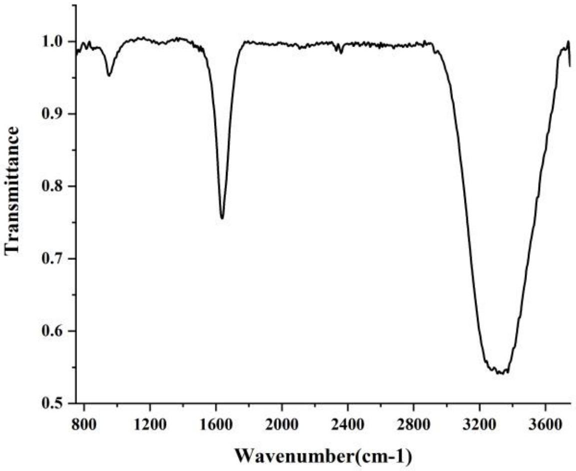
Transmittance
1.0
0.9
0.8
0.7
0.6
0.5
800
1200
1600
2000
2400
2800
3200
3600
Wavenumber(cm-1)

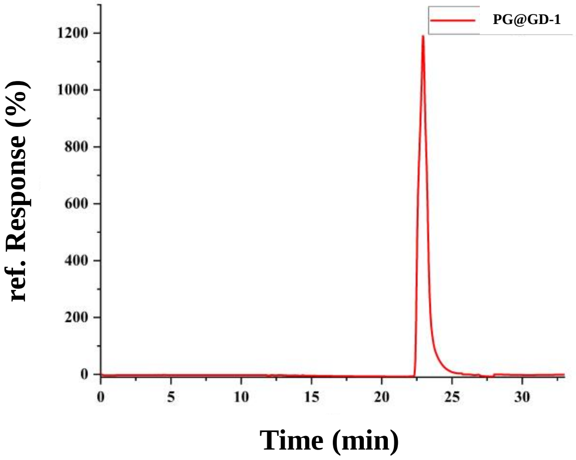
PG@GD-1
ref. Response (%)
1200
1000
800
600
400
200
0
0
5
10
15
20
25
30
Time (min)

PG@GD-2

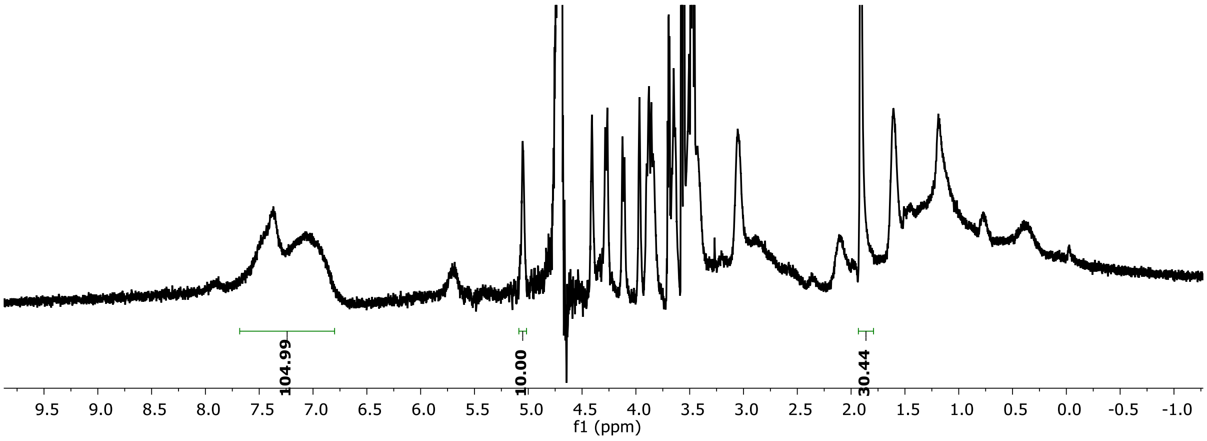
104.99
10.00
30.44
9.5 9.0 8.5 8.0 7.5 7.0 6.5 6.0 5.5 5.0 4.5 4.0 3.5 3.0 2.5 2.0 1.5 1.0 0.5 0.0 -0.5 -1.0
f1 (ppm)

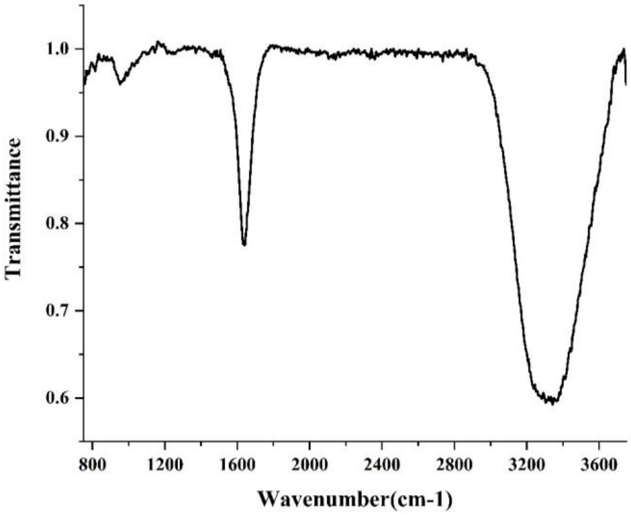
Transmittance
1.0
0.9
0.8
0.7
0.6
800 1200 1600 2000 2400 2800 3200 3600
Wavenumber(cm-1)

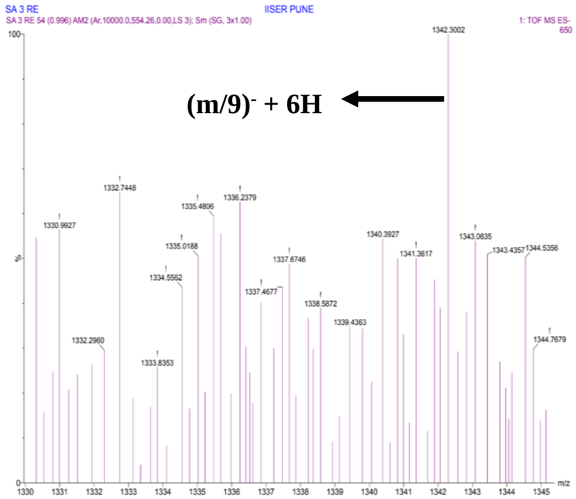

SA 3 RE
IISER PUNE
SA 3 RE 54 (0.996) AM2 (Ar,10000.0,554.26,0.00,LS 3); Sm (SG, 3x1.00)
1: TOF MS ES-
650
$(m/9)^{-} + 6H$
1342.3002
1332.7448
1330.9927
1336.2379
1335.4806
1335.0188
1334.5562
1337.6746
1337.4677
1338.5872
1339.4363
1340.3927
1341.3617
1343.0835
1343.4357
1344.5356
1344.7679
1332.2960
1333.8353
m/z

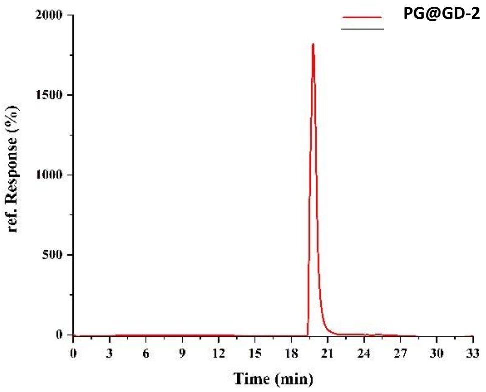

PG@GD-2
ref. Response (%)
Time (min)

PG@GD-3

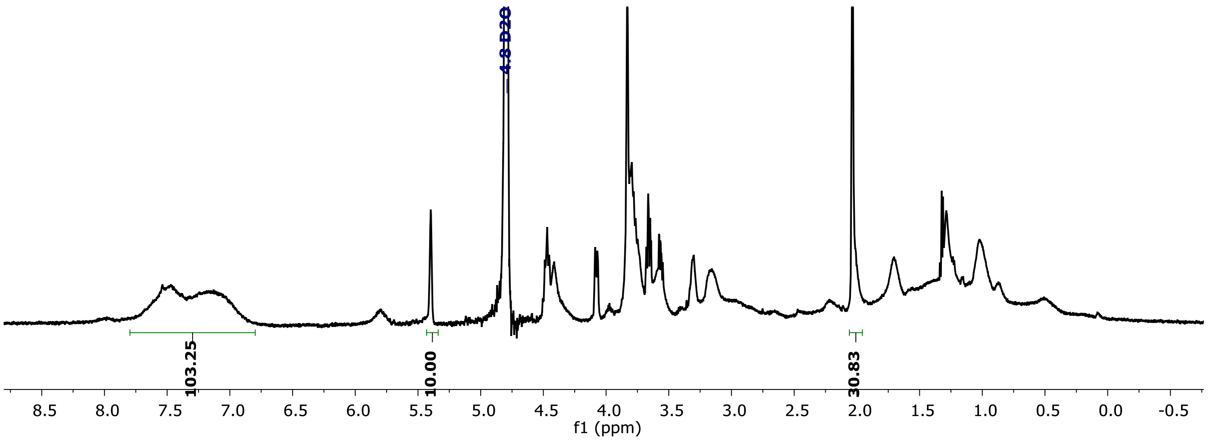

4.8 D2O
103.25
10.00
30.83
8.5 8.0 7.5 7.0 6.5 6.0 5.5 5.0 4.5 4.0 3.5 3.0 2.5 2.0 1.5 1.0 0.5 0.0 -0.5
f1 (ppm)

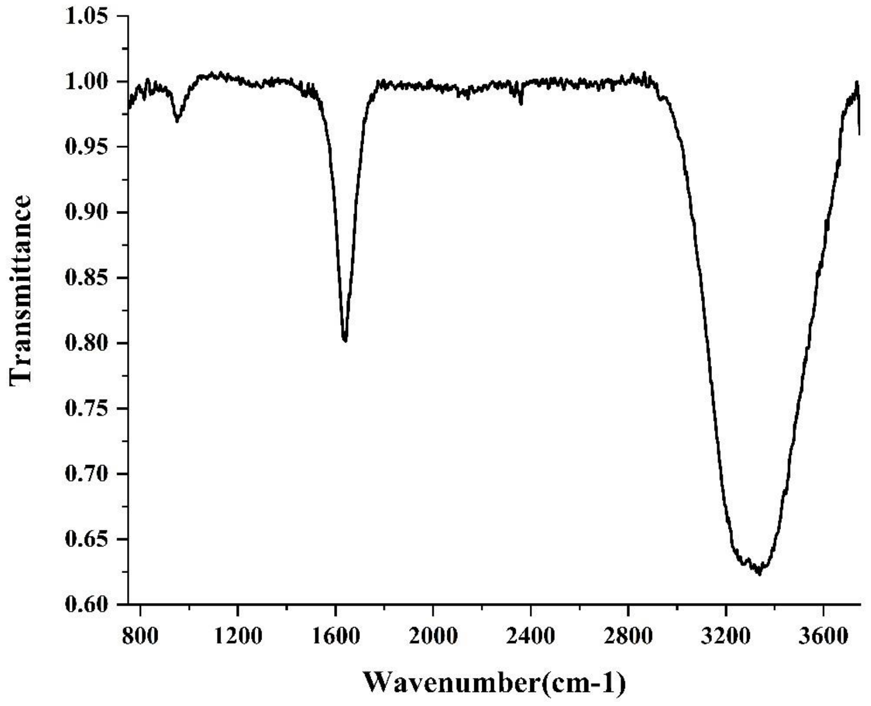

Transmittance
1.05
1.00
0.95
0.90
0.85
0.80
0.75
0.70
0.65
0.60
800 1200 1600 2000 2400 2800 3200 3600
Wavenumber(cm-1)

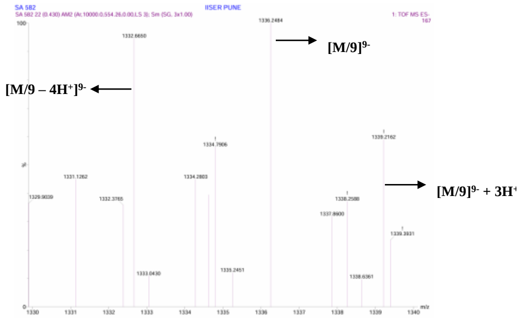

SA 582
SA 582 22 (0.430) AM2 (Ar,10000.0,554.26,0.00,LS 3); Sm (SG, 3x1.00)
IISER PUNE
1: TOF MS ES-
167
1336.2484
[M/9]9-
1332.6650
[M/9 – 4H+]9-
1334.7906
1339.2162
1331.1262
1334.2803
[M/9]9- + 3H+
1329.9039
1332.3765
1338.2588
1337.8600
1339.3931
1333.0430
1335.2451
1338.6361
%
m/z

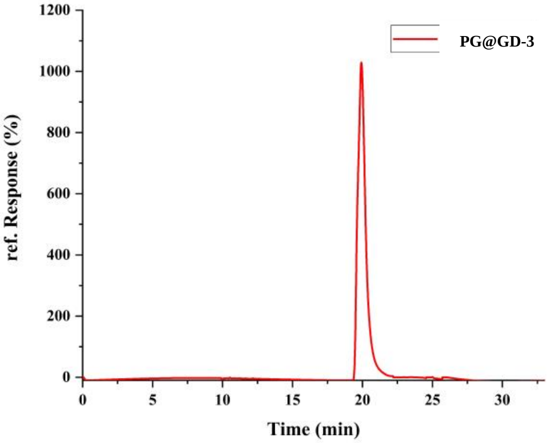

PG@GD-3
ref. Response (%)
Time (min)

PG@GD-4

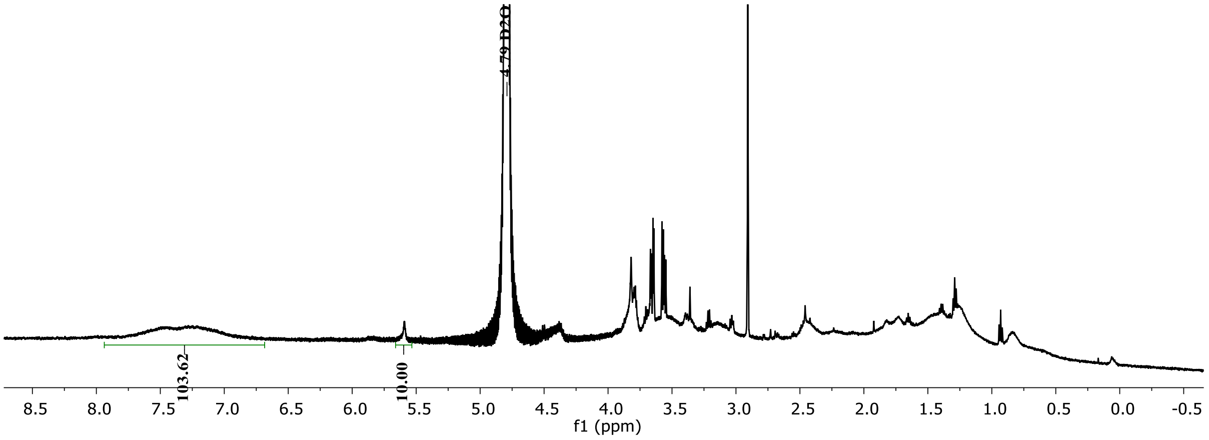

4.79 D2O
103.62
10.00
8.5 8.0 7.5 7.0 6.5 6.0 5.5 5.0 4.5 4.0 3.5 3.0 2.5 2.0 1.5 1.0 0.5 0.0 -0.5
f1 (ppm)

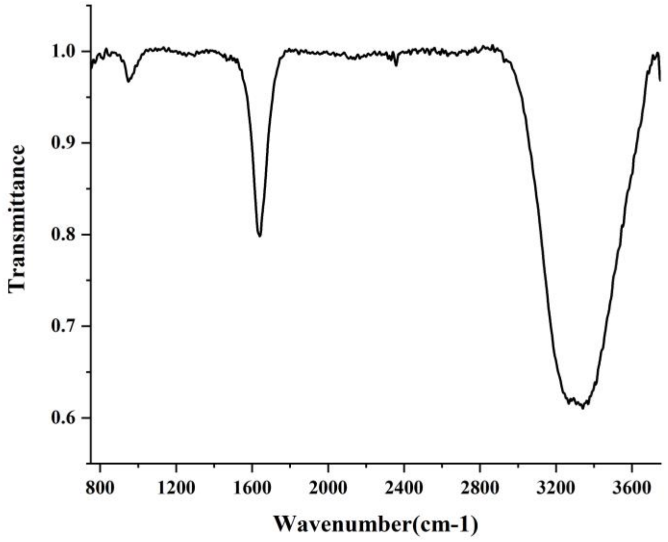

Transmittance
1.0
0.9
0.8
0.7
0.6
800 1200 1600 2000 2400 2800 3200 3600
Wavenumber(cm-1)

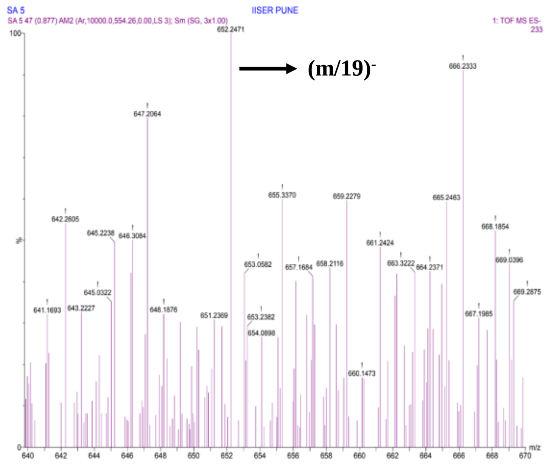

SA 5
SA 5 47 (0.877) AM2 (Ar,10000.0,554.26,0.00,LS 3); Sm (SG, 3x1.00)
IISER PUNE
1: TOF MS ES-
233
(m/19)-
652.2471
666.2333
647.2064
655.3370
659.2279
665.2463
642.2605
668.1854
645.2238
646.3084
661.2424
653.0582
657.1684
658.2116
663.3222
664.2371
669.0396
645.0322
641.1693
643.2227
648.1876
651.2369
653.2382
654.0898
667.1985
669.2875
660.1473
m/z

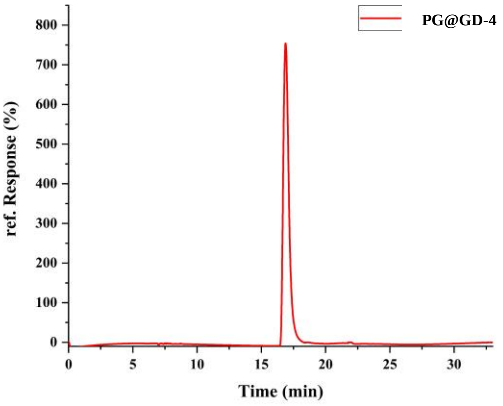

PG@GD-4
ref. Response (%)
800
700
600
500
400
300
200
100
0
0 5 10 15 20 25 30
Time (min)

PG@GD-5

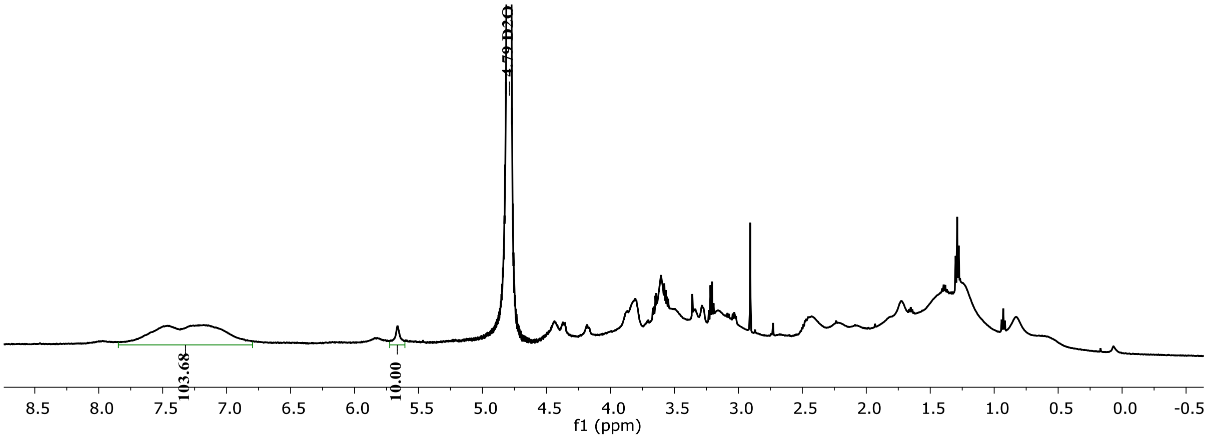

4.79 D2O
103.68
10.00
8.5
8.0
7.5
7.0
6.5
6.0
5.5
5.0
4.5
4.0
3.5
3.0
2.5
2.0
1.5
1.0
0.5
0.0
-0.5
f1 (ppm)

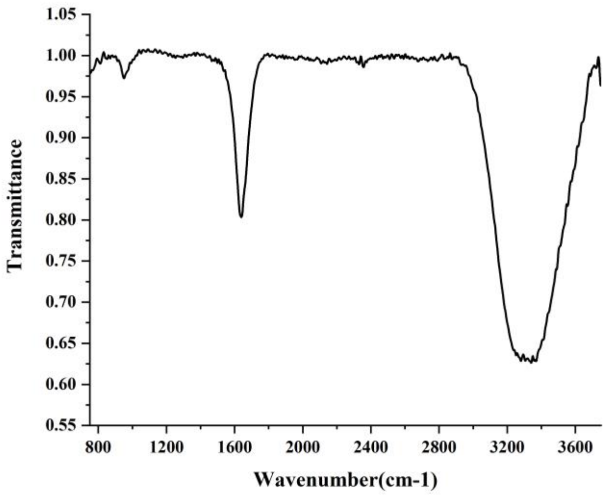

Transmittance
1.05
1.00
0.95
0.90
0.85
0.80
0.75
0.70
0.65
0.60
0.55
800
1200
1600
2000
2400
2800
3200
3600
Wavenumber(cm-1)

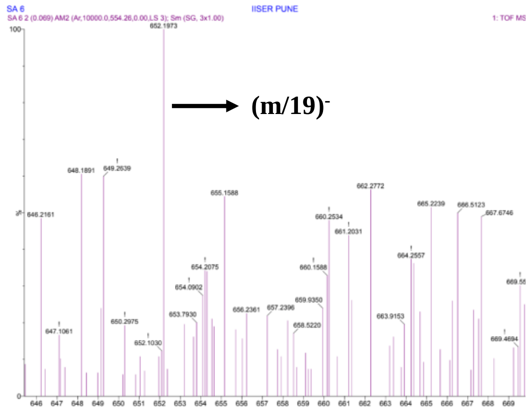
SA 6
SA 6 2 (0.069) AM2 (Ar,10000.0,554.26,0.00,LS 3); Sm (SG, 3x1.00)
IISER PUNE
1: TOF MS
652.1973
(m/19)-
646.2161
647.1061
648.1891
649.2639
650.2975
652.1030
653.7930
654.0902
654.2075
655.1588
656.2361
657.2396
658.5220
659.9350
660.1588
660.2534
661.2031
662.2772
663.9153
664.2557
665.2239
666.5123
667.6746
669.4694
100
0
646
647
648
649
650
651
652
653
654
655
656
657
658
659
660
661
662
663
664
665
666
667
668
669

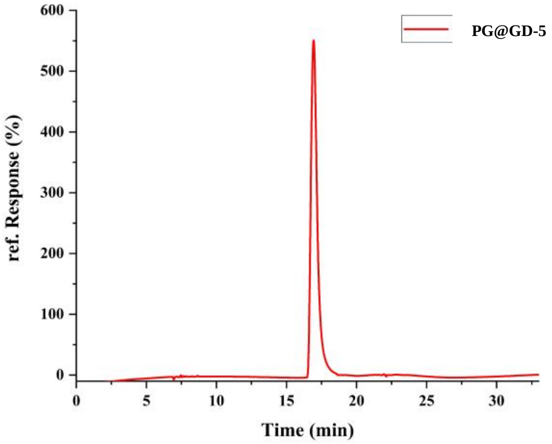
PG@GD-5
ref. Response (%)
Time (min)
0
100
200
300
400
500
600
5
10
15
20
25
30
